# Systematic analysis of somatic mutations impacting gene expression in 12 tumour types

**DOI:** 10.1038/ncomms9554

**Published:** 2015-10-05

**Authors:** Jiarui Ding, Melissa K. McConechy, Hugo M. Horlings, Gavin Ha, Fong Chun Chan, Tyler Funnell, Sarah C. Mullaly, Jüri Reimand, Ali Bashashati, Gary D. Bader, David Huntsman, Samuel Aparicio, Anne Condon, Sohrab P. Shah

**Affiliations:** 1 Department of Molecular Oncology, BC Cancer Agency, 675 West 10th Avenue, Vancouver, British Columbia, Canada V5Z 1L3; 2 Department of Computer Science, University of British Columbia, 2366 Main Mall, Vancouver, British Columbia, Canada V6T 1Z4; 3 Centre for the Translational and Applied Genomics, BC Cancer Agency, 600 West 10th Avenue, Vancouver, British Columbia, Canada V5Z 4E6; 4 Department of Pathology and Laboratory Medicine, University of British Columbia, 2211 Wesbrook Mall, Vancouver, British Columbia, Canada V6T 2B5; 5 The Donnelly Centre, University of Toronto, 160 College Street, Toronto, Ontario, Canada M5S 3E1; 6 Canada's Michael Smith Genome Sciences Centre, 570 West 7th Avenue, Vancouver, British Columbia, Canada V5Z 4S6

## Abstract

We present a novel hierarchical Bayes statistical model, xseq, to systematically quantify the impact of somatic mutations on expression profiles. We establish the theoretical framework and robust inference characteristics of the method using computational benchmarking. We then use xseq to analyse thousands of tumour data sets available through The Cancer Genome Atlas, to systematically quantify somatic mutations impacting expression profiles. We identify 30 novel *cis*-effect tumour suppressor gene candidates, enriched in loss-of-function mutations and biallelic inactivation. Analysis of *trans*-effects of mutations and copy number alterations with xseq identifies mutations in 150 genes impacting expression networks, with 89 novel predictions. We reveal two important novel characteristics of mutation impact on expression: (1) patients harbouring known driver mutations exhibit different downstream gene expression consequences; (2) expression patterns for some mutations are stable across tumour types. These results have critical implications for identification and interpretation of mutations with consequent impact on transcription in cancer.

## Abstract (web summary)

Assessing functional impact of mutations in cancer on gene expression can improve our understanding of cancer biology and may identify potential therapeutic targets. Here, Ding *et al*. describe a novel statistical model named xseq for a systematic survey of how mutations impact transcriptome landscapes across 12 different tumour types.

## Graphical abstract

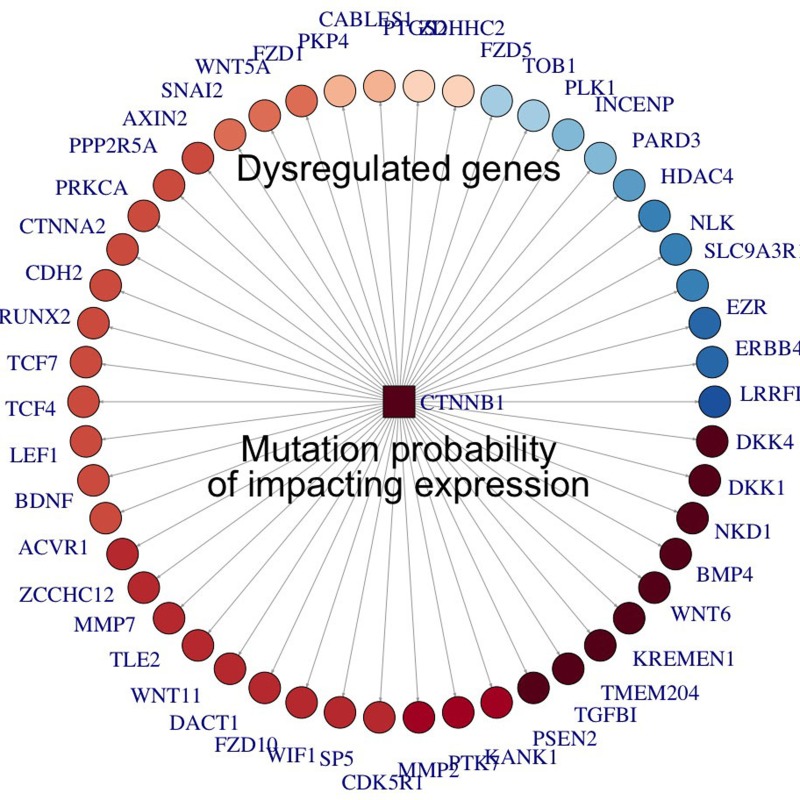

Human cancers acquire malignant properties following a stepwise accumulation of somatic genomic alterations^1^ and subsequent evolutionary selection on resultant phenotypic changes. Genomic mutations (loosely classified as single-nucleotide variants (SNVs), small insertions and deletions (indels), copy number alterations and genomic rearrangements) show widespread variation in their functional impacts on gene products, biochemical pathways and phenotypic properties. Consequently, the effect of a mutation is often difficult to predict. Previous computational approaches to predict functional effects of mutations include: evolutionary conservation of the mutation sites across species, the chemical properties of amino-acid substitutions^2^ and the frequency of mutations of a gene of interest relative to its expected background rate of mutations^3^. These approaches rely on interpretation of DNA sequences alone and do not consider other molecular measurements such as gene expression, methylation or proteome measurements that are co-acquired from the same tumour samples. Thus, histological or molecular context of mutations is often ignored in their interpretation. To address this deficiency, we propose that additional patterns representing functional consequences of mutations can be determined through simultaneous analysis of mutation and gene expression data.

We have assessed the impact of mutations on gene expression as a means of quantifying potential phenotypic effects, and for novel cancer gene discovery. This concept is motivated by biological hypotheses predicting that some functional mutations will exhibit a ‘transcriptional shadow', resulting from a mechanistic impact on the gene expression profile of a tumour. For example, loss-of-function mutations (nonsense mutations, frame-shifting indels, splice site mutations or homozygous copy number deletions) occurring in tumour suppressor genes such as *TP53* can cause loss of expression due to nonsense-mediated messenger RNA (mRNA) decay^4^ or gene dosage effects. In this context, we define a *cis*-effect as a genetic or epigenetic aberration that results in upregulation or downregulation of the gene itself. In contrast, some mutations can disrupt the expression of other genes in the same biochemical pathway (*trans*-effects^5^). This class of mutations tends to cast a long transcriptional shadow over many genes across the genome^5^. β-Catenin (*CTNNB1*) mutations, which drive constitutive activation of Wnt signalling in several cancer types, are a potent example of mutational impact on gene expression.

Large-scale data sets generated by international consortia provide opportunities to define the landscape of mutations impacting gene expression in thousands of tumours across the major cancer types. The Cancer Genome Atlas (TCGA) projects have generated genomic and transcriptomic data from multiple cancer types, providing a systematic characterization of somatic mutations^6^, copy number alterations^7^, oncogenic processes^8^, mutated sub-networks or pathways^9^, and genomic signature-defined tumour subtypes^10^.

There are few computational tools available^11^ to systematically identify mutations impacting gene expression (Supplementary Table 1 summarizes representative methods). CONEXIC^12^ is a probabilistic approach to detect driver copy number regulators and their target genes. EPoC^13^ derives driver copy number alterations and their target genes using differential equations to model the expression synthesis rate of a gene as a function of its copy number and the regulatory effects of other genes. MOCA^14^ detects differently expressed genes in the presence of mutations in a gene, and tests the significance of the correlation (between mutation and gene differential expression). PARADIGM^15^ integrates copy number and expression to identify disrupted pathways. DriverNet^16^ uses a combinatorial approach and a greedy algorithm to nominate cancer driver genes. However, none of these methods can identify individual mutations that correlate with dysregulated gene networks.

We present a novel statistical model, xseq, using a hierarchical Bayes approach and apply it to the analysis of thousands of tumour data sets available through TCGA, systematically examining the impact of somatic mutations on expression profiles across 12 tumour types. We demonstrate the robustness of xseq by conducting extensive computational benchmarking, and by testing xseq on an independent breast cancer data set. We identify 30 novel *cis*-effect tumour suppressor gene candidates, enriched in loss-of-function mutations and frequent biallelic inactivations. We identify 150 genes from *trans*-effect analysis impacting expression networks in the 12 cancer types, with 60 known cancer genes and 89 novel predictions. Notably, 29 of these newly predicted genes are known interacting partners of cancer driver genes. On the basis of the *trans*-analysis, we find two important characteristics of mutations impacting gene expression that could not be revealed with other methods: (1) a stratification of patients harbouring known driver mutations, but that exhibit different downstream gene expression consequences; (2) identification of mutations driving expression patterns that are stable across tumour types, thereby nominating important molecular targets for therapeutic intervention, transcending anatomic sites of origin.

## Results

### Data sets

We used somatic point mutation, copy number alteration and gene expression data in 12 cancer types, from the TCGA Pan-Cancer project^17^ (Table 1). A total of 2,786 patients with all three types of data were included in our analyses. For *trans*-analysis, focal copy number homozygous deletions and amplifications (with four or more copies) predicted by GISTIC^7^,^18^ were also encoded as inputs to xseq. We did not analyse the *cis*-effects of copy number alterations since the majority of them have *cis*-effects on gene expression^19^.

### Modelling the effects of mutations on expression with xseq

To address the central question of whether somatic mutations in a patient's tumour impact gene expression, we developed a generative probabilistic model, xseq. We present the conditional probability distributions and descriptions of the random variables of the model in Supplementary Table 2. The model specification, assumptions and inference algorithm are fully explained in the Supplementary Methods and the Methods sections. We briefly describe them here.

**Figure 1:**
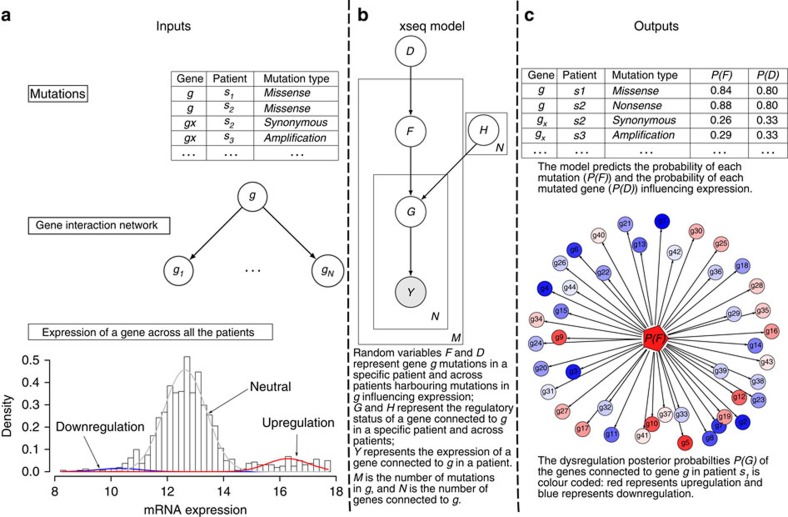
Overview of the xseq modelling framework. (**a**) The inputs to the xseq model: a mutation matrix typically from next-generation sequencing, a gene interaction network and a gene expression matrix. xseq models the expression of a gene across all the patients by mixture distributions. The three mixture components represent downregulation, neutral and upregulation, respectively. (**b**) The graphical model representation of xseq with the plate notation. Circles represent random variables and arrows denote dependencies between variables. Boxes are plates that represent replicates. For example, the graph represents a gene mutated in *M* patients (we assume that a gene is mutated only once in a patient), and the gene is connected to *N* genes. (**c**) xseq predicts the posterior marginal probabilities of each gene (*P*(*D*)), each mutation (*P*(*F*)) influencing expression and the regulatory probabilities of the genes connected to the mutated gene in a patient (*P*(*G*)).

The xseq model is predicated on the idea that mutations with functional effects on transcription will exhibit measurable signals in mRNA transcripts biochemically related to the mutated gene—thus imposing a transcriptional shadow across part (or all) of a pathway. To infer this property, three key inputs are required for the model (Fig. 1a): a patient-gene matrix encoding the presence/absence of a mutation (any form of somatic genomic aberrations that can be ascribed to a gene, for example, SNVs, indels or copy number alterations); a patient-gene expression matrix encoding continuous value expression data (for example, from RNA sequencing or microarrays); and a graph structure encoding whether two genes are known to be functionally related (for example, obtained through literature, databases or co-expression data). xseq uses a precomputed ‘influence graph'^20^ as a means to incorporate prior gene–gene relationship knowledge into its modelling framework (Methods). For analysis of mutation impact in-*cis*, the graph reduces to the simple case where the mutated gene is only connected to itself. Given the inputs, we calculate the actual expression of the *n*th gene connected to mutated gene *g* in patient *m*, denoted by *Y*_*g*,*m*,*n*_.

The output of xseq consists of: (a) the probability that a recurrently mutated gene *g* influences gene expression across the population of patients (denoted by *P*(*D*_*g*_=1), Supplementary Table 2); and (b) the probability that an individual mutation in gene *g* in an individual patient *m* influences expression within that patient (denoted by *P*(*F*_*g*,*m*_=1)).

In addition to the random variables *D*_*g*_, *F*_*g*,*m*_ and *Y*_*g*,*m*,*n*_, xseq also models the gene expression distribution over the patient population with gene-specific three component mixture models of Student's *t*-emission densities. The three mixture components represent downregulation, neutral or upregulation, respectively (Fig. 1a). *G*_*g*,*m*,*n*_∈{downregulation, neutral, upregulation} denotes the status of the *n*th gene connected to gene *g* in patient *m*. The central assumption is that a mutation in gene *g* of patient *m* impacting gene expression (denoted by *F*_*g*,*m*_=1) more frequently co-associates with non-neutral states in its connected genes, compared with the mutations that do not impact expression. The specific direction of expression is encoded by *H*_*g*,*n*_∈{downregulation, upregulation} to denote the *n*th gene connected to gene *g* is upregulated or downregulated when mutations in *g* influence expression. (We also consider a simplified model, xseq simple without modelling the directionality of gene regulation for a specific gene, that is, without the *H* variable in Fig. 1b for simplicity of inference.) To represent a recurrent pattern of expression impact across multiple patients, we consider information across all patients with a mutation in gene *g*. This allows for borrowing of statistical strength across multiple gene expression patterns co-associating with mutations to generalize whether a mutated gene is impacting expression across the population (denoted by *D*_*g*_=1). (Supplementary Fig. 1a shows a simple xseq model.)

We use the Belief Propagation algorithm^21^ for inference and the Expectation Maximization (EM) algorithm for parameter learning^22^ (Supplementary methods). The inference problem is to compute the posterior probabilities *P*(*D*_*g*_), *P*(*F*_*g*,*m*_) and *P*(*G*_*g*,*m*,*n*_) given the input. The learning problem is to estimate the conditional probabilities of a variable given its parents, for example, *θ*_*F*=1|*D*=1_—the probability of a mutation impacting expression in a specific patient given that this gene's mutations impact expression across patients (Fig. 1b; Supplementary Table 2). For clarity of presentation, we have removed the subscripts and directly refer to *D*, *F*, *G* and *Y*. For example, we use *P*(*D*), *P*(*F*) and *P*(*G*) to denote the posteriors *P*(*D*_*g*_), *P*(*F*_*g*,*m*_) and *P*(*G*_*g*,*m*,*n*_), respectively.

To add interpretative capacity to xseq outputs, we developed binary classifiers to determine if genes with high *P*(*D*) showed tumour suppressor properties *P*(TSG) or activating oncogenic properties *P*(OCG) (Methods, Modelling loss-of-function mutations and hotspot mutations). The classifiers were motivated by the pattern of distributed loss-of-function mutations across a gene for tumour suppressors (for example, *TP53*) and hotspot mutations at one or relatively few loci for oncogenes (for example, *KRAS*).

### Computational benchmarking and validation of xseq

**Figure 2:**
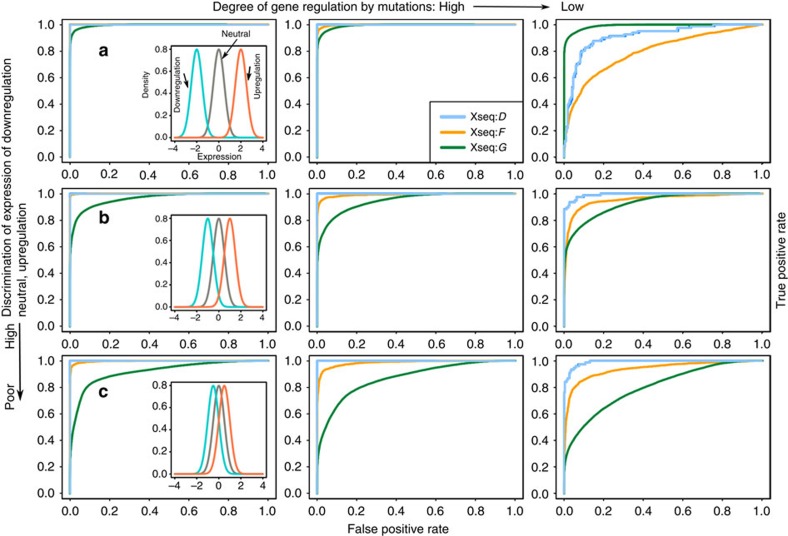
Theoretical performance of xseq on simulated data sets. Each plot depicts a receiver operating characteristic (ROC) curve, which displays the true positive rate as a function of false-positive rate. (**a**) The expression of genes that are downregulated, neutral and upregulated is highly discriminative (first row), (**b**) moderately discriminative (second row) and (**c**) poorly discriminative (third row, see the enclosed figures, where cyan is downregulation, grey is neutral and red is upregulation, respectively). The ROC curves in the first column, second column and the third column were computed when the degree of dysregulation of the expression of connected genes by mutations was high, moderate and low, respectively.

**Figure 3:**
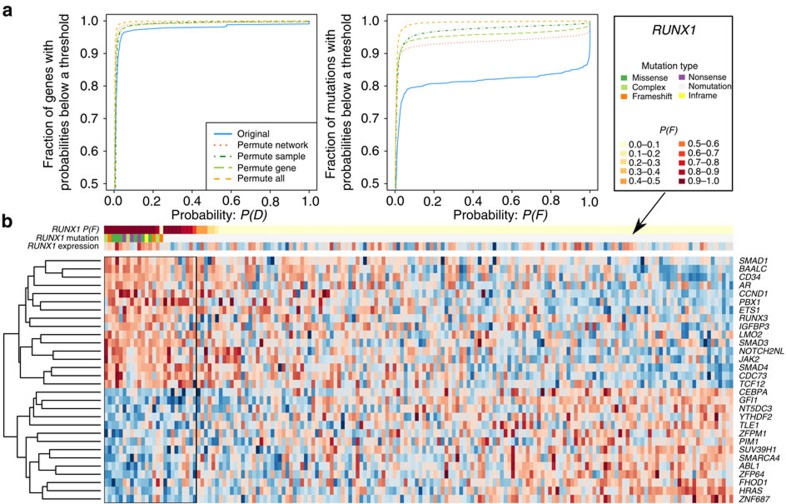
Permutation analysis of the TCGA acute myeloid leukaemia data sets. (**a**) Left panel shows the empirical distribution functions of *P*(*D*), and the right panel shows the empirical distribution functions of *P*(*F*) estimated from different permuted data sets. (**b**) Heatmap shows the expression of genes connected to *RUNX1*: red represents high expression and blue represents low expression. Here columns represent patients and rows represent genes. For the patients without *RUNX1* mutations, we ‘assume' the mutations still exist and estimate the probabilities of individual mutations *P*(*F*). The mutation type ‘complex' of a gene in a patient represents the gene harbouring multiple types of mutations in the patient.

We examined the theoretical performance of xseq via simulation and permutation analyses. To investigate the performance of xseq under different noise levels, we simulated data (Supplementary Fig. 2; Supplementary Methods) from 9 hyperparameter sets, and 10 independent realizations of data for each hyperparameter set. Overall, xseq had high sensitivity and specificity in recovering the latent variables, for example, even for the most challenging data we simulated, xseq achieved mean area under the curves of 0.99 and 0.94 for *D* and *F*, respectively (Fig. 2). xseq performance was improved when given the true values for *H* (Supplementary Fig. 3). xseq simple also performed well, but with inferior performance relative to xseq (Supplementary Figs 4–5). We performed a simulation analysis of the *cis*-effects of somatic mutations, and found that the results were similar to those obtained from *trans*-simulations (Supplementary Fig. 6). We also permuted the TCGA acute myeloid leukaemia (Table 1) data set^23^ for testing. The false discovery rates (FDRs) for *D* and *F* were 0.002 and 0.02, respectively (Fig. 3; Methods).

We executed a cross-validation analysis by splitting each TCGA data set into approximately equally sized discovery and validation data sets. We trained a model on the discovery data set, and used the trained model to predict the validation data set, with 10 repeats for each tumour type. We defined the validation rate as the proportion of high-probability predicted genes (*P*(*D*)≥0.8, see next section on picking the threshold) in the training data also predicted to have high probabilities in the validation data. For bona fide cancer genes (Supplementary Data 1; Methods), the median validation rate was 0.625 across all the 12 tumour types (Supplementary Fig. 7; Supplementary Table 3). For all of the predictions from the discovery data, the median validation rate was 0.492. The validation rate is sensitive to the number of patients (for example, the median validation rate for the predicted bona fide cancer genes in colon adenocarcinoma and breast invasive carcinoma (BRCA) was 0 and 0.73, respectively). Restricting analysis to genes with at least five mutations in both the discovery and validation data sets, median validation rates for the bona fide cancer genes and all the predicted genes increased to 0.68 and 0.63, respectively (Supplementary Fig. 8; Supplementary Table 4).

To examine how the model would translate to independently generated data, we used the METABRIC data^5^ to validate the predicted copy number alterations from TCGA in breast cancer. METABRIC copy number alterations^24^ were generated with Affymetrix SNP6.0; however, gene expression was generated using Illumina microarrays. We applied the xseq model trained on the TCGA breast cancer data to analyse the METABRIC breast cancer data. This analysis generated 14 genes with high probability (*P*(*D*)≥0.8), representing a strict subset of the 42 genes predicted in the TCGA breast cancer data (Supplementary Table 5; Supplementary Figs 9–10).

Finally, we quantitatively benchmarked xseq against CONEXIC^12^ and xseq simple (Supplementary Methods). We found that xseq was more specific but potentially less sensitive than CONEXIC in predicting copy number alterations influencing expression (Supplementary Table 6). xseq increased sensitivity without loss of specificity of results relative to xseq simple (Supplementary Table 7; Supplementary Figs 11–12).

### *Cis*-effect loss-of-function mutations across the TCGA data

**Figure 4:**
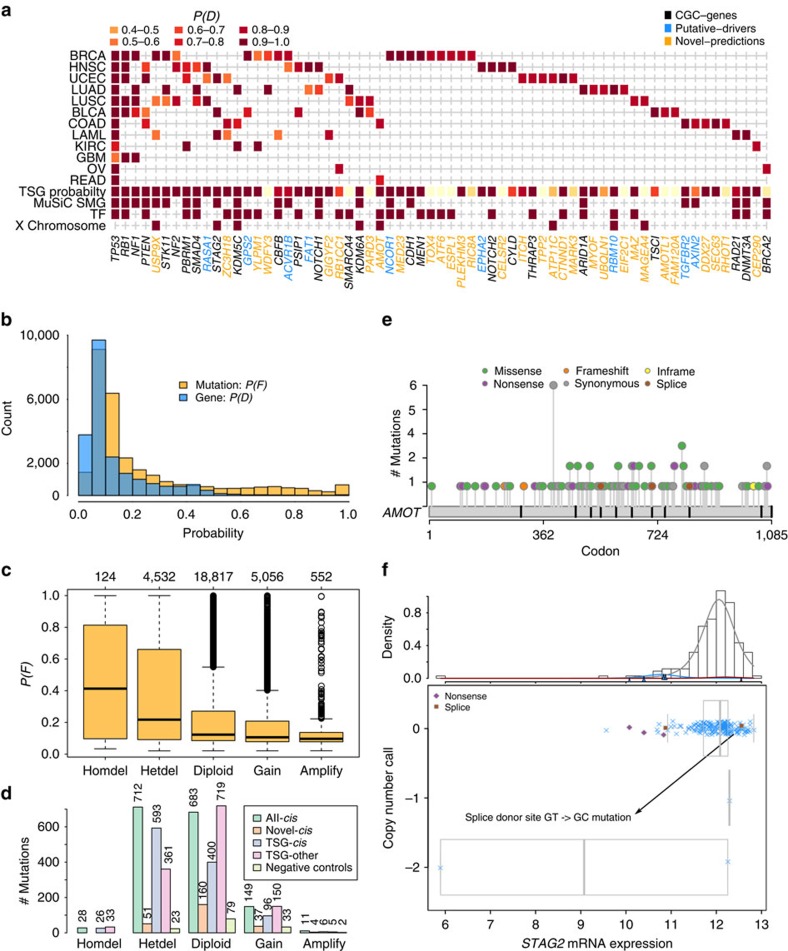
The 65 genes harboured loss-of-function mutations with strong *cis*-effects on the expression of these genes. (**a**) The predicted *cis*-effect loss-of-function mutations across 12 tumour types (*P*(*D*)≥0.8 in at least one tumour type). (**b**) The histograms of posterior marginals of mutations and genes across tumour types. (**c**) The posterior marginals of mutations separated based on copy number status. (**d**) The loss-of-function mutations in the 65 *cis*-effect genes (all-*cis*), 30 novel predictions (novel *cis*), 23 *cis*-effect tumour suppressor genes (TSG-*cis*), 108 non-*cis*-effect TSGs (TSG-other) and 30 negative control genes (negative controls) segregated based on copy number status. (**e**) A ‘novel' tumour suppressor gene *AMOT* is not significantly mutated based on frequency-based methods, but *AMOT* is enriched in loss-of-function mutations (tumour suppressor gene probability *P*(TSG)=0.92). (**f**) The loss-of-function mutations in *STAG2* typically correlate with lower expression, except for a splice donor site mutation GT→GC mutation (both GT and GC are used by the splicing machinery). MuSiC SMG, significantly mutated genes predicted by MuSiC; TF, transcription factor; TSG probability, tumour suppressor gene probability.

We began analysis of the TCGA data by focusing on the *cis*-effect impacts of loss-of-function mutations (frameshift, nonsense and splice site mutations) on gene expression, yielding 65 genes across the 12 data sets with *P*(*D*)≥0.8 (Fig. 4a; Supplementary Data 2). (We chose the threshold of 0.8 for *P*(*D*) to balance prediction of novel genes with introduction of false positives, see Supplementary Fig. 13. Changes to the results with thresholds in the range 0.75 to 0.85 were minor.) To place these predictions in the context of known cancer genes, we compiled a list of 603 bona fide cancer genes (Methods) from the Cancer Gene Census (CGC) database^25^ (Fig. 4a, black coloured genes), Vogelstein *et al*.^1^ and Lawrence *et al*.^26^ (Fig. 4a, blue coloured genes). In total, 34/65 xseq predictions overlapped bona fide cancer genes. We compared xseq predictions with those^6^ predicted by an orthogonal method, MuSiC^27^, which computes the statistical significance of the population mutation frequency of a gene above an expected background mutation rate to predict its role as a cancer gene. As MuSiC uses only mutation data, and not expression data, we used it as a benchmark to determine the effect of integrating gene expression data on cancer gene discovery. MuSiC predicted 22/65 genes as significantly mutated. Importantly, 13/43 of the xseq genes that were not predicted by MuSiC were present in the list of bona fide cancer genes, suggesting that integrating gene expression information can complement the existing mutation frequency-based methods to identify mutated cancer genes.

We next characterized the tumour suppressor properties of the 65 xseq *cis*-effect predictions for consistency with known patterns of enrichment for loss-of-function mutations (Methods). We found 51/65 genes with tumour suppressor characteristics (*P*(TSG)≥0.2, Supplementary Fig. 14a–b; Supplementary Data 2). Results were robust to a more conservative threshold, yielding 47/65 genes with *P*(TSG)>=0.5 (Supplementary Fig. 15). The *cis*-effect loss-of-function mutations were co-associated with genomic copy number (one-way analysis of variance test *P* value<0.001, Fig. 4c–d), with xseq *cis*-effect genes enriched for coincidence with hemizygous deletion (Fisher's exact test *P* value<0.001, Fig. 4c–d. The statistical test method when reporting *P* values is omitted from this point onwards if Fisher's exact test is used.)

Additional biological characterization of the cis-effect genes suggested strong enrichment for transcription factors, phosphoproteins and X chromosome genes. Nearly half (30/65) of the *cis*-effect genes encode transcription factors (Fig. 4a; *P* value<0.001), as annotated in the Checkpoint database^28^. Most of the *cis*-effect genes (54/65, *P* value<0.001) encode human phosphoproteins (Supplementary Data 2), consistent with recent work predicting cancer driver genes based on enriched mutations in phosphorylation regions^29^. Finally, *cis*-effect genes were disproportionately found on chromosome X^30^ (8/65, *P* value<0.01; Fig. 4a). Taken together, these data indicate xseq *cis*-effect predicted genes' properties are well aligned with known characteristics of tumour suppressor genes.

For the 30 novel predictions (not in our bona fide cancer driver gene list nor significantly mutated based on MuSiC analysis), we searched for literature in support of their tumour suppressor roles in cancer. In total, we found strong connections to tumour suppressor genes for at least 17 genes (Supplementary Discussion). The tumour suppressor roles of several of these genes have recently been elucidated (for example, *UBQLN1* (ref. 31) and *MED23* (ref. 32)). Notably, three genes (*AMOT*, *AMOTL1* and *ITCH*) encode proteins in the Hippo signalling pathway, involved in restraining cell division and promoting apoptosis. We further characterized the 30 genes according to criteria presented above for all the 65 genes. Of the 30 novel predictions, 18 genes accumulated enriched loss-of-function mutations (*P*(TSG)≥0.2, *P* value<0.001), 10 genes encode transcription factors (*P* value<0.05), 21 genes (*P* value<0.001) encode human phosphoproteins, three genes reside on the X chromosome (*P* value<0.1). All of the novel genes were rarely mutated in the 12 studied cancer types (based on MuSiC results; Supplementary Fig. 16). A total of 51/252 loss-of-function mutations in these genes were in hemizygous deletion regions (*P* value<0.05).

As a comparison with a negative control group of genes, we used the 30 genes flagged as false-positive cancer driver genes in a recent study^3^. These genes are not significantly mutated after correction for gene length, DNA replication time and other factors in estimating the background mutation rates^3^. All 30 genes had *P*(TSG)<0.1. In addition, loss-of-function mutations in these genes were not enriched in hemizygous deletion regions (*P* value=0.7; Fig. 4c–d) and all 30 were predicted to have probabilities *P*(*D*)<0.6 by the xseq model (the *P*(*D*) histogram is in Supplementary Fig. 17), suggesting that the FDR for xseq is relatively low in the TCGA data, as shown in the permutation analysis.

We next estimated the proportion of known tumour suppressor genes harbouring *cis*-effect loss-of-function mutations. We began by enumerating a set of 131 known tumour suppressor genes from both CGC^25^ and Vogelstein *et al*.^1^ (Supplementary Data 3). We found that 23/131 genes (significant enrichment of *cis*-effect genes, *P* value<0.001) were predicted to exhibit *cis*-expression effects indicating that loss-of-function mutations in ∼17.6% of tumour suppressor genes yield concomitant changes in mRNA expression levels.

### *Trans*-effect mutations across the TCGA data

Application of xseq to predict mutations impacting expression *in trans* resulted in a total of 150 genes across the 12 cancer types (*P*(*D*)≥0.8; Supplementary Table 7; Supplementary Fig. 18a). Sixty of the 150 (40%) genes are bona fide cancer genes. We characterized these 60 *trans*-effect genes with annotated roles in cancer according to biological functions and found that 30/60 genes encode transcription factors (*P* value<0.001), 14/60 genes encode protein kinases (*P* value<0.001) and 4/60 genes (*ATRX, BAP1, KDM5A, SETD2*, *P* value<0.01) are chromatin regulatory factors. Moreover, 26/60 genes encode cell cycle proteins (gene ontology term: GO:0007049). By comparison, MuSiC only predicted 35/60 of these genes (Supplementary Data 4). One gene (*ACVR2A*) was predicted by both xseq and MuSiC but was not in the bona fide cancer gene list. Taken together, xseq uniquely predicted 89 novel genes through *trans*-impacting expression analysis (Supplementary Data 5).

Further investigation revealed that 29/89 of the novel predicted genes were known interacting partners of previously characterized bona fide cancer genes. The gene (protein) interactions were assessed based on the high-quality protein–protein interaction networks^33^ (downloaded from the Center for Cancer Systems Biology website^34^). As for the 60 genes above, 23/89 genes encode transcription factors (*P* value<0.1), 7/89 genes encode protein kinases (*P* value<0.05), 3/89 genes are chromatin regulatory factors (*P* value<0.1), 18/89 genes encode proteins of the cell cycle process (*P* value<0.01). Pathway analysis indicated these genes encode proteins involved in major cancer pathways such as cell proliferation, apoptotic process, mitotic cell cycle, chromatin modification, cell migration and focal adhesion (Supplementary Data 6). Nineteen genes were predicted to have *P*(TSG) or *P*(OCG)≥0.2 (Supplementary Data 5). The gene which harboured the largest number of high-probability mutations was *KPNA2* in breast cancer (Supplementary Fig. 19, 43 mutations with *P*(*F*)≥0.5. As shown in Supplementary Fig. 18b–c, *P*(*F*) follows a bimodal distribution centred at 0 and 1. Results are similar if choosing slightly different thresholds for *P*(*F*).)

To examine other sources of evidence of functional effects, we analysed high-probability missense mutations across all the tumour types, and computed the enrichment of phosphorylation-related SNVs (pSNVs)^29^,^35^. (We found 839 missense mutations with *P*(*F*)≥0.5 in the genes with *P*(*D*)≥0.8, and also overlapped the set of missense mutations analysed^35^.) Of these mutations, 620 were unique when the same amino-acid residue replacement in different patients was considered. We performed two analyses where the same amino-acid substitution missense mutations (from different patients) were considered as separate events or collapsed into the same event (unique). The Pan-Cancer data set included 241,700 (236,367 unique) missense mutations. Among them, 16,840 (16,074 unique) mutations were pSNVs. Of the high-probability SNVs, 232/839 of them were pSNVs (134/620 unique). The high-probability missense mutations were highly enriched in pSNVs in both analyses (*P* value <0.001). These results provided additional data to support functional activity of the xseq predictions specifically related to impact on phosphorylation.

### Expression dysregulation across tumour types

Certain genes are frequently mutated in multiple tumour types^6^. We asked whether these mutations across tumour types correlated with the dysregulation of the same set of genes. We focused on those genes whose mutations were predicted to influence gene expression in multiple tumour types. For each gene connected to the mutated gene *g* in a tumour type, we counted how many times this gene was dysregulated (*P*(*G*=‘upregulation')≥0.5 or *P*(*G*=‘downregulation')≥0.5) in the presence of high-probability mutations (*P*(*F*)≥0.5). We analysed downregulation and upregulation independently using a binomial exact test to test the significance of this correlation (high-probability mutations and gene dysregulation). The binomial distribution parameters were obtained by maximum likelihood estimation from all count data. From this analysis, we found 17/20 recurrent genes had at least 1 gene upregulated or downregulated in 2 tumour types (Supplementary Data 7, 8). Mutations in *RB1* correlated with the same group of gene dysregulations across several tumour types. In particular, we observed that *RB1* mutations correlated with E2F family gene upregulations (for example, *E2F1*; Supplementary Data 7; Supplementary Fig. 20) in bladder urothelial carcinoma (BLCA), BRCA, glioblastoma multiforme (GBM), lung squamous cell carcinoma (LUSC), ovarian serous cystadenocarcinoma (OV) and uterine corpus endometrioid carcinoma (UCEC; Table 1), as well as genes encoding mini-chromosome maintenance proteins, for example, *MCM5* (Supplementary Data 7; Supplementary Fig. 21) in BRCA, GBM, lung adenocarcinoma, LUSC and OV. To confirm these correlations, for each gene connected to *RB1* in the original full influence graph (Methods), in each tumour type, we compared the expression of this gene in the patients with *RB1* mutations to the patients without *RB1* mutations using the Limma package^36^. We then aggregated all the obtained *P* values from the genes connected to *RB1* across tumour types, and computed the FDRs^37^. We found that *E2F1* was upregulated in BLCA, BRCA, GBM, LUSC and UCEC (FDR<0.1, Supplementary Fig. 22). We performed a similar analysis for *MCM5* and found it was upregulated in BRCA, GBM, LUSC, OV and UCEC (FDR<0.1; Supplementary Fig. 23).

**Figure 5:**
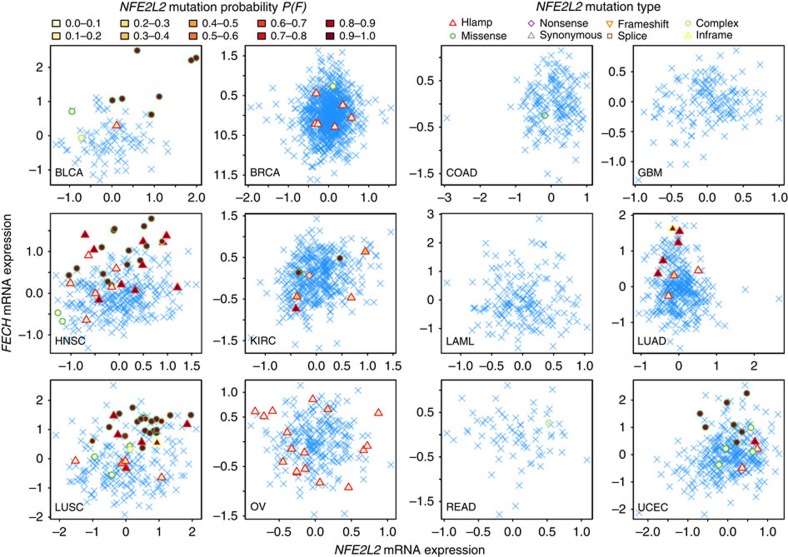
*NFE2L2* mutations and *FECH* upregulation. *NFE2L2* mutations were predicted to correlate with *FECH* expression upregulation in five types of cancer: BLCA, HNSC, LUAD, LUSC and UCEC. Each dot in the scatterplots represents the expression of *NFE2L2* and *FECH* in a patient. A blue cross ‘ × ' means the patient does not have *NFE2L2* mutations. Other types of symbols represent different kinds of mutations (Hlamp, copy number amplifications). The filled colours encode the estimated mutation probability *P*(*F*) from *trans*-analysis (both *FECH* expression and the expression of other *NFE2L2* interaction partners determine *P*(*F*)). HNSC, head and neck squamous cell carcinoma; LUAD, lung adenocarcinoma.

In addition, aberrations (mutations and amplifications) in the transcription factor *NFE2L2* in six different tumour types (BLCA, head and neck squamous cell carcinoma, kidney renal clear cell carcinoma, lung adenocarcinoma, LUSC and UCEC) exhibited *trans*-effects on gene expression (*P*(*D*)≥0.8). Two genes, *MAFG* (Supplementary Figs 24–25) and *FECH* (Fig. 5; Supplementary Fig. 26) were significantly upregulated in five and four tumour types, respectively, in the presence of *NFE2L2* aberrations (FDR<0.1; Supplementary Data 7). As *MAFG*, *FECH* and *NFE2L2* reside on chromosome 17, 18 and 2, respectively, the correlations are not likely caused by gene dosage effects. Several other genes were also upregulated in the presence of *NFE2L2* aberrations, for example, *NQO1, TXNRD1, PRDX1, GSR, GPX2, GCLM, FTL, AKR1C1, TXN, SQSTM1, GSTA1, KEAP1, GSTA4, ABCC1*, and *GCLC* were upregulated in six to three tumour types (Supplementary Data 7). The boxplots in Supplementary Fig. 27 and the scatter plots in Supplementary Fig. 28 show the correlation between *NFE2L2* aberrations and its direct regulator and binding partner, *KEAP1* expression upregulation.

### Stratifying patients harbouring the same gene mutations

**Figure 6:**
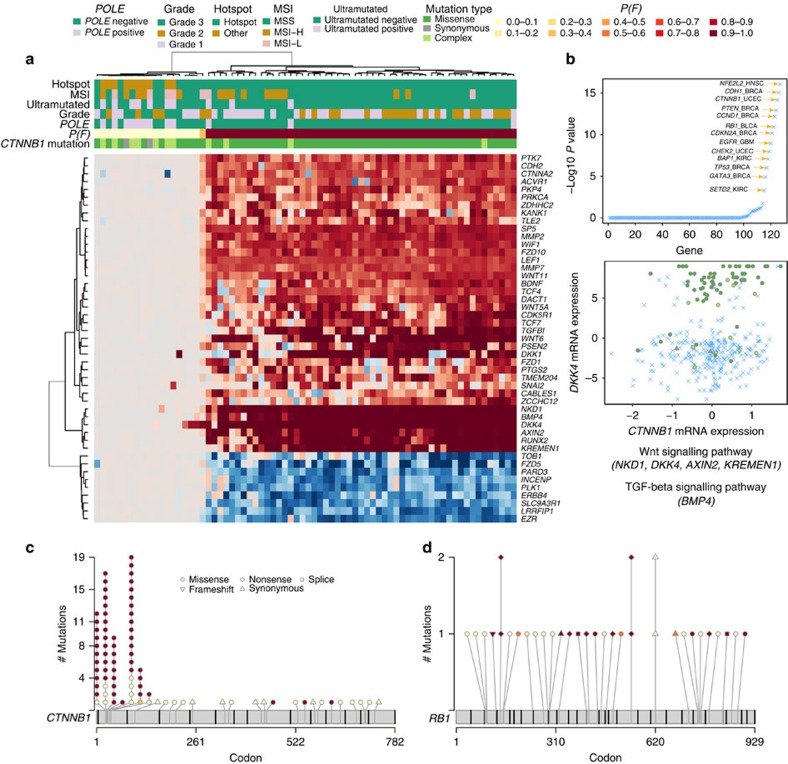
Patients harbouring the same gene mutations but with variations in *trans*-associated gene expression. (**a**) In UCEC, *CTNNB1* mutations correlated with the upregulation of a set of genes, and downregulation of another set of genes. The most extreme upregulated genes included *BMP4* (in TGF-β signalling pathway), *NKD1*, *AXIN2*, *DKK4* and *KREMEN1* (in Wnt signalling pathway). The downregulated genes included Wnt signalling pathway gene *FZD5*. Here red colour in the heatmap represents gene upregulation and blue colour represents gene downregulation. (**b**) The smallest unimodality dip-test *P* values of *P*(*F*) of the 127 significantly mutated genes across tumour types. (**c**) The mutation sites, mutation types and *P*(*F*) (filled colours) of *CTNNB1* mutations (**d**) and *RB1* mutations in UCEC. MSI, microsatellite instability; MSS microsatellite stable; MSI-H, MSI-high; MSI-L, MSI-low; TGF-β, transforming growth factor-beta.

We investigated whether xseq probabilities *P*(*F*) could stratify patients harbouring mutations in the same cancer driver gene. We analysed each of the 127 genes from Kandoth *et al*.^6^ in each tumour type for the presence of bimodal xseq *P*(*F*) distributions over patients harbouring mutations in the genes of interest (Supplementary Methods). Twenty-two commonly mutated genes exhibited bimodal distributions in at least one tumour type (Supplementary Table 8; Fig. 6b; Supplementary Fig. 29). This was particularly evident for *CTNNB1* mutations in UCEC (Fig. 6a); 53/72 patients harboured high-probability *CTNNB1* mutations (*P*(*F*)≥0.5), with all 53 patients harbouring *CTNNB1* hotspot mutations (mutations hitting codons between 31 and 45). By contrast, only 9/19 patients without high xseq probability *CTNNB1* mutations harboured hotspot mutations (Fig. 6c). In addition, 9/19 patients harboured *POLE* mutations, or were annotated as ‘ultramutated' (tumours with more mutations than Q3+IQR × 4.5, where Q3 is the third quartile of mutation counts across a corresponding tumour type, and IQR is the interquartile range, as defined in syn1729383), suggesting that the *CTNNB1* mutations were inconsequential passenger mutations (Supplementary Fig. 30, *CTNNB1 P*(*F*) distribution). Moreover, patients in the *P*(*F*)≥0.5 group were significantly younger than patients with *P*(*F*)<0.5 (mean age 57.5 versus 65.7 years old, one-sided *t*-test *P* value<0.01).

Similar results for *RB1* mutations in UCEC are shown in Fig. 6d. All 11 loss-of-function mutations (in eight patients) were predicted to have high probabilities (*P*(*F*)≥0.5); however, only 2/13 patients that did not harbour loss-of-function mutations were predicted to accumulate high-probability mutations (*P*(*F*)≥0.5, Fig. 6d). Taken together, although genes such as *CTNNB1* and *RB1* frequently harbour driver mutations, they still likely accumulate passenger mutations without impact on gene expression. As such, patients' tumours with these ‘inert' mutations do not exhibit expected pathway dysregulation. xseq is therefore capable of sub-stratifying patients into meaningful phenotypic groups, separating patients with mutations and dysregulated pathways from those patients with mutations, but normal pathway activities.

*TP53* mutations in UCEC also showed bimodal distributions (Supplementary Fig. 31). *TP53* frequently accumulates both loss-of-function mutations and missense mutations. The variation in *P*(*F*) cannot be explained by the types and positions of the mutations (Supplementary Fig. 32). However, patients with *P*(*F*)>=0.5 were more likely to harbour copy number hemizygous deletions (36 patients harboured co-occurring hemizygous deletions, compared with eight patients with *P*(*F*)<0.5; only nine patients lacked copy number alterations in the group with *P*(*F*)≥0.5 compared with 12 in the group with *P*(*F*)<0.5, *P* value<0.005.)

## Discussion

We developed a probabilistic model, xseq to quantitatively assess the association of mutations with dysregulated gene expression in 12 tumour types. Computational benchmarking and assessment of independent data sets have demonstrated the robustness of xseq. Our results have implications for the interpretation of somatic mutations in retrospective, discovery-based studies.

Systematic analysis of mutation and expression landscapes from >2,700 tumours uncovered several novel patterns. We revealed 30 novel tumour suppressor candidate genes by *cis*-effect loss of expression analysis. These genes showed the hallmarks of tumour suppressor genes including a distribution of loss-of-function mutations, and biallelic inactivation through loss-of-function mutations and heterozygous deletions. In addition, we assessed the landscape of mutations impacting gene expression *in trans* across the 12 tumour types. These results implicated 89 novel genes with mutations impacting gene expression. In total, 33% of these genes had functional relationships with cancer genes in core tumourigenic processes. These genes were not nominated by mutation analysis alone, suggesting that integrated analysis of mutations and gene expression is a complementary approach towards comprehensive identification of functional mutations. Recent synthesis of mutation rates and discovery ‘saturation' in genome-wide sequencing studies has indicated that current standard of study design has under-sampled important mutations, and that for some 50 tumour types, sequencing of >2,000 cases are needed to reach comprehensive sampling^26^. The combined *cis*- and *trans*-analyses led to the elucidation of >100 novel candidate cancer genes predicted to impact expression. Integration of gene expression data directly into analysis of mutations will therefore help to bridge the discovery gap left by DNA mutation analysis alone.

Results from xseq analysis identified two important characteristics for biological interpretation of mutations. The *trans*-analysis revealed that the same mutated gene in different patients can exhibit distinct expression impacts. In our analysis, constitutive activation of Wnt signalling genes due to *CTNNB1* mutation segregates almost exclusively with known hotspot mutations. However, several cases exhibited mutation in *CTNNB1* without evidence of Wnt activation, resulting in low xseq probabilities. These cases were primarily phenotyped as hypermutators due to mismatch repair deficiency and/or *POLE* mutations^38^, and patients were statistically older at diagnosis. Thus, a real phenotypic distinction associates with low and high xseq *P*(*F*) probabilities, providing evidence for integrative analysis of mutations and expression as a route to stratifying phenotypically distinct tumours in the context of the same mutations.

xseq analysis identified several genes that had consistent expression impact across tumour types. Despite distinct histologies and cell contexts of source tumours, *RB1* loss-of-function mutations and *NFE2L2* mutations/amplifications exhibited similar expression patterns. *RB1* binds and inhibits the E2F transcription factor family. Accordingly, we observed that *RB1* mutations correlated with E2F family gene upregulation across tumour types. *NFE2L2* binds to its regulator *KEAP1* and regulates the expression of antioxidant-related genes to protect against oxidative damage. We observed *NFE2L2* mutations correlated with upregulation of *KEAP1*, as well as of oxidative stress genes (for example, *GCLM, GCLC, TXNRD1, GPX2* and *NQO1*). While therapeutic responses to targeted inhibitors administered against the same mutation can have variable effects due to intrinsic gene expression context (for example, *BRAF* inhibition in melanoma and colorectal cancer^39^), the mutations we outlined (such as *RB1* and *NFE2L2* mutations) exhibit stable profiles and represent important targets for future development of broadly applicable therapeutics. An intriguing evolutionary implication arises from these mutations: phenotypic impact is selected for in multiple heterogeneous tumour microenvironments, indicating independent convergence of phenotype transcending cell context.

xseq is not able to distinguish different mutations of a gene within a specific patient—a limitation, as these mutations may result in different functional impacts. Although genes are rarely mutated multiple times within a single patient, some large tumour suppressor genes (such as *ARID1A*) accumulate multiple mutations, as a result of their long coding sequences. Similarly, in glioblastoma and lung cancers, *EGFR* is frequently mutated multiple times in single patients, often due to the emergence of clonal populations following the administration of *EGFR* inhibitors^40^. Examining the expression impact properties of such mutations in clonal populations would likely require advanced single-cell methods^41^.

In conclusion, this work provides a route towards closing the cancer gene discovery gap in the field of cancer genome sequencing. Direct, model-based integration of mutations and co-acquired gene expression measurements from tumour samples enhances interpretation capacity of discovered mutations leading to optimal selectivity of targets for functional studies and development of novel therapeutics.

## Methods

### A generative model of the effects of mutations on expression

The xseq model specifies how the expression *Y* of a group of genes in a patient is influenced by the somatic mutation status of a gene *g* in the patient (Fig. 1b; Supplementary Fig. 1). The main question we address is whether gene *g* co-associates with disrupted expression to itself (*cis*-method) or its connected genes as defined by an influence graph (see below).

On the basis of the xseq model structure in Fig. 1b, for a mutated gene *g*, xseq specifies a joint distribution^42^ (assuming *g* is mutated in *M* patients and *g* has *N* connected genes):


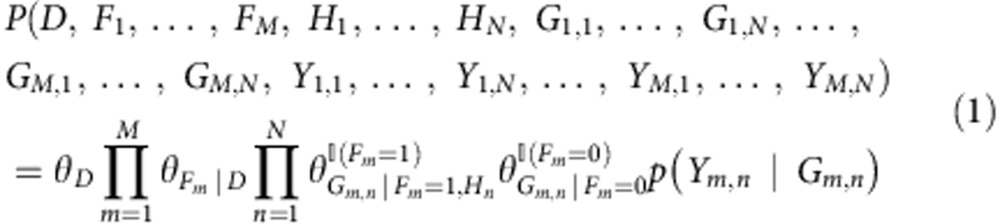


where 
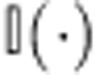
 is the indicator function, and 
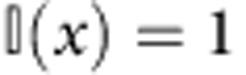
 when *x*=TRUE, otherwise 
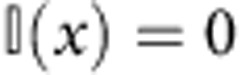
. The *θ*s are the parameters (conditional probabilities) of xseq (Supplementary Table 2). These parameters can take different values based on the subscripts, for example, *θ*_*D*_ can take *θ*_*D*=0_ and *θ*_*D*=1_ based on the value of *D*. For simplicity, we consider the case with only one mutated gene and we remove ‘*g*' in the notations. Here we assume that a gene is just mutated once in a specific patient, that is, *M* equals to the number of accumulated mutations in gene *g*. Therefore, *m* is the patient (mutation) index, and *n* is the gene index, that is, *Y*_*m*,*n*_ represents the expression of the *n*th gene connected to *g* in the *m*th patients harbouring mutations in *g*. We now explain how we execute parameter learning and inference over this joint distribution.

### Inference of latent variables and parameter estimation

It is computationally intractable to marginalize the joint distribution in equation (1) to infer the posterior marginals *P*(*D*), *P*(*F*) and *P*(*G*). Instead, xseq uses the belief propagation algorithm^21^,^43^ to efficiently do exact inference of these posterior marginals. Here we assume that the variable 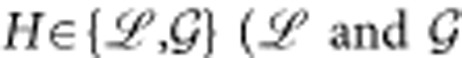 means downregulation of expression and upregulation of expression, respectively) has been estimated (estimation of *H* is discussed below). Then, we can convert the non-tree-structured xseq model to a tree for efficient inference (Supplementary Methods). The belief propagation algorithm has time and memory complexity exponential in the maximum number of parents per node (two for xseq when *H* is given). Detailed descriptions of the belief propagation algorithm for xseq inference can be found in Supplementary Methods.

The EM algorithm^22^ is used to learn the parameters (Supplementary Table 2) in xseq. EM algorithm iterates between the E-step and the M-step to find a local maximum of the objective likelihood function. Below, we listed the M-step update equations, and present the detailed derivations of the formulas in Supplementary Methods.


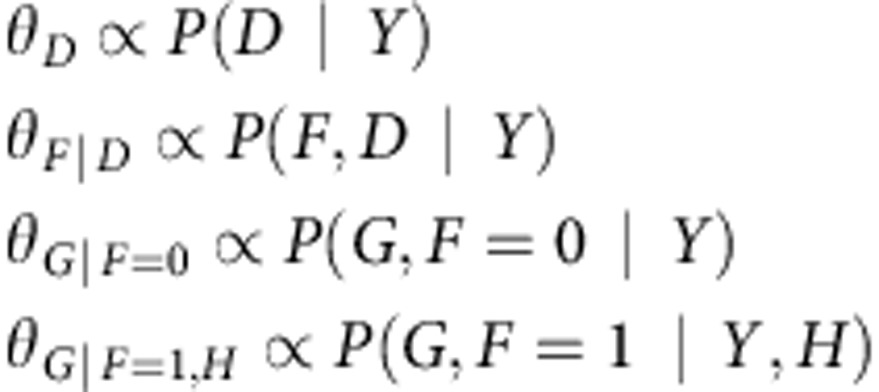


The terms *P*(*D* | *Y*), *P*(*F*,*D* | *Y*), *P*(*G*,*F*=0 | *Y*) and *P*(*G*,*F*=1 | *Y*,*H*) are computed in the E-step using the belief propagation algorithm.

Fixing *H* is key for converting the model to a tree structure for efficient inference and learning (Supplementary Methods). There may be several ways to estimate *H*, for example, using differential expression analysis to test whether a gene is upregulated or downregulated, or directly getting this information from pathway databases. In our experiments, to systematically analyse all the data sets, for the *n*th gene connected to gene *g*, we estimate the upregulation probability by 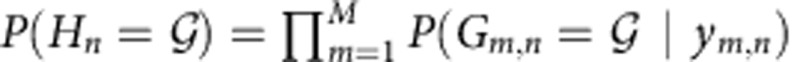, where *m* is the *m*th patients harbouring gene *g* mutations, *M* is the total number of patients harbouring gene *g* mutations and *y*_*m*,*n*_ is the expression of the *n*th gene connected to *g* in patient *m*. 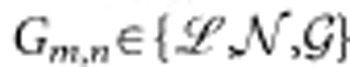 means downregulation, neutral and upregulation of the *n*th gene connected to *g* in patient *m*, respectively. The probability *P*(*G*_*m*,*n*_= | *y*_*m*,*n*_) is estimated off-line by the posterior distribution of *y*_*m*,*n*_ being generated from the ‘upregulation' component (see Conditional distributions of gene expression values). Similarly, we can estimate the downregulation probability: 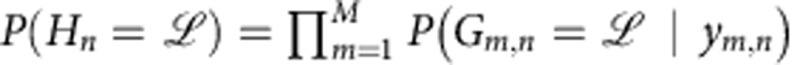.

### Conditional distributions of gene expression values

The conditional distributions *p*(*Y*=*y* | *G*) are modelled as Student's *t*-distributions and estimated off-line. For example, the conditional distribution of gene *g* expression distribution is modelled as a Student's *t*-distribution when gene *g* is downregulated:


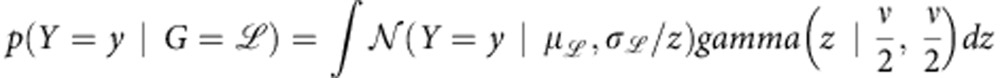


where *y* is the expression level of gene *g*, 
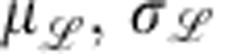
 and *ν* are the parameters of the Student's *t*-distribution. As the parameter *ν* increases, the Student's *t*-distribution approaches a Gaussian distribution 
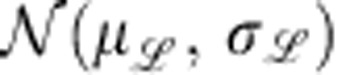
. Compared with Gaussian distributions, the Student's *t*-distributions are more robust to outliers, especially when *ν* is small. Now, the observed gene expression distribution is a mixture of three Student's *t*-distribution:


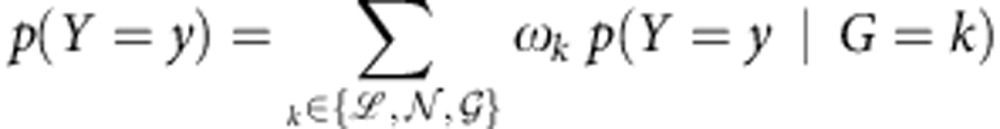


where *ω*_*k*_ is the mixture weight of mixture component *k*. We also use the EM algorithm to uncover the parameters of the Student's *t*-distributions.

### Influence graph

In principle, the influence graph can be any such graph encoding gene regulation. The *i*th vertex of the graph represents gene (protein) *g*_*i*_ and edge *w*_*i*,*j*_ represents the association strength between gene (protein) *g*_*i*_ and *g*_*j*_. For analysis presented in this study, we constructed a combined functional gene association network by merging the STRING v9.1 (ref. 44) functional protein association network, the pathway data sets from KEGG^45^, WikiPathway^46^ and BioCyc^47^, and transcription factor-targets networks. The pathways have already been integrated into the IntPath database^48^. The transcription factor-targets network^49^ is downloaded through the transcription factor encyclopedia web API. The ENCODE^50^ transcription factor ‘proximal' and ‘distal' networks are also included in the combined network (download from the website^51^). The majority of these interactions are transcription factor-target gene interactions (∼1% between transcription factor interactions in the ‘proximal' network^50^).

For each data set, we construct a weighted network. The weight of an interaction represents our prior confidence of the interaction. For the data sets that do not provide weights for interactions, a default weight of 0.8 is used. The STRING protein–protein interaction network is already a weighted network so we use their provided weights. To merge these networks, for a specific interaction, we take the largest weight for this interaction across different networks. We then only keep the interactions with at least median confidence (threshold of 0.4, the default threshold suggested for the STRING database). In this combined network, 17,258 genes (proteins) connect to 19,070 genes (proteins) through 898,032 interactions. This network is almost weakly connected (22 genes do not connect to rest genes).

Then, for each mutated gene, we test whether the genes connected to it are differentially expressed with adjusted *P* value (BH method) threshold of 0.05. If there exist differentially expressed genes, we only keep these genes and set their connection weights to 1. In addition, if a gene is not differently expressed in a specific tumour type but differently expressed in other tumour types based on Fishers' combined *P* value FDR≤0.05, we also set their connection weights to 1. If no differentially expressed genes exist for a given mutated gene, we use the network from the original weighted network.

### Code availability

The model and the influence and learning algorithms have been implemented in the statistical programming language R^52^, and can be downloaded from The Comprehensive R Archive Network^53^, or from our website^54^.

### Permutation analysis

We performed several permutations to investigate the influence of each component of xseq on the final predictions. First, we switched the patient names within the mutation matrix (Fig. 3a, permute sample; Supplementary Figs 33–34). Even after permutation, some mutations were still predicted to have high probabilities *P*(*F*). To help explain this phenomenon, we generated an expression heatmap (Fig. 3b), which showed the expression of genes connected to *RUNX1*. We can see that some patients without *RUNX1* mutations still showed similar expression pattern to those with *RUNX1* mutations. This ‘phenocopy'^55^ effect could result in some patients without mutations but high predicted probabilities *P*(*F*). Phenocopying may be a common event in cancer because of DNA methylation and other epigenetic alterations, and it may suggest novel treatment opportunities. For example, there is increasing evidence that treating of patients based on phenotypes (expression) instead of genotypes (DNA mutations) produces better outcomes in some types of cancer^56^. We also switched the gene names within the mutation matrix (Fig. 3a, permute gene), and the results showed similar performance to those by switching patients.

Next, we randomly drew the same number of connected genes as given by the combined network (Fig. 3a, permute network). Because the gene regulation information is sparse and some master regulators can influence the expression of huge number of genes, the model may still predict a few mutations with high probability *P*(*F*) because these ‘randomly drawn' genes might be truly regulated by the mutated genes. Finally, if both the mutation matrix and the network were shuffled, there were very few predicted high-probability mutations (dashed orange curves in Fig. 3a, permute all). We performed the same processing steps after permutations thus minimizing the possibility of introducing bias. In addition, we kept the expression matrix the same in all permutation analyses.

### Collecting bona fide driver genes

We collected the genes from the manually curated, and widely used CGC database^25^ and two major recent review papers^1^,^26^ as our reference bona fide cancer driver genes. Specifically, we collected 519 genes from CGC^25^, 125 genes predicted by the ‘20/20 rule'^1^(more details below), 66 recently discovered frequently mutated genes collected in the Supplementary Table 4 of Lawrence *et al*^26^ and 33 genes predicted by MutSig and have strong and consistent connections to cancer^26^. In summary, these data sets include 603 unique genes in total (Supplementary Data 1). The 127 significantly mutated genes predicted by the MuSiC suite^6^ are not counted because we use this data set for several comparisons. Notice that in our analysis, we use the samples with expression, copy number alterations and mutations. Therefore, xseq analyzes a subset of mutations used by the MuSiC suite.

### Modelling loss-of-function mutations and hotspot mutations

The mutation patterns of most known tumour suppressor genes, and oncogenes are highly characteristic and non-random^1^. In a recent review^1^, a ‘20/20 rule' is used to identify driver genes: for oncogenes, at least 20% of all the mutations are required to be hotspot missense mutations or in-frame indels; for tumour suppressor genes, at least 20% of all the mutations are required to be loss-of-function mutations.

Here we extend the ‘20/20 rule' using mixture-of-binomial modelling of loss-of-function mutations and hotspot mutations. We analyse oncogenes and tumour suppressor genes separately. When predicting oncogenes, we first count the number of hotspot mutations *n*_*g*,rec_ (recurrent missense mutations and in-frame indels) in gene *g* and the total number of mutations *N*_*g*_ in gene *g*. Then, we model the mutation count distribution as a mixture of two binomial distributions: one component for oncogenes and the other component for non-oncogenes:


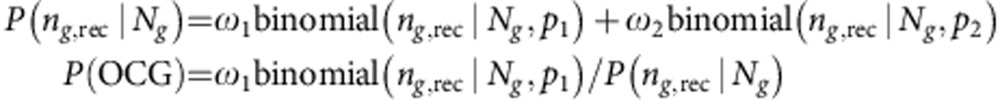


Then *P*(OCG) is defined as the posterior of *n*_*g*,rec_ in the mixture component with higher success rate, namely *p*_1_ here. The mixture parameters **ω**=(*ω*_1_,*ω*_2_) and success rates **p**=(*p*_1_,*p*_2_) are estimated by the EM algorithm.

Similarly, when predicting tumour suppressor genes, we first count the number of loss-of-function mutations *n*_*g*,loss_ in gene *g*, and *N*_*g*_. Then, we model the count distribution as a mixture of two binomial distributions: one component representing tumour suppressor genes and the other representing non-tumour suppressor genes:


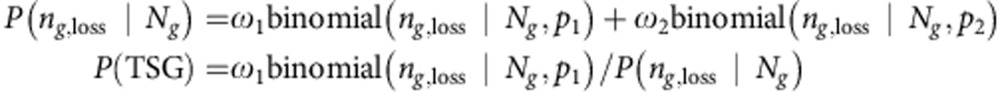


*P*(TSG) is defined as the posterior of *n*_*g*,loss_ in the mixture component with higher success rate (*p*_1_ here). Again, the mixture parameters **ω** and success rates **p** are estimated by the EM algorithm.

The mixture-of-binomial approach can be considered as a generation of the ‘20/20 rule' because of its ability to estimate the parameters from data, and to account for the total number of mutations to compute posterior probabilities of genes to be oncogenes or tumour suppressor genes. To make the estimated parameters more accurate, we also added extra genome-wide screen data from COSMIC v64 (ref. 57), downloaded from syn1855816, and the Pan-Cancer data downloaded from syn1710680. Supplementary Figure 14 shows the binomial mixture modelling of oncogenes and tumour suppressor genes for all the genome-wide screen somatic mutation data. We used a threshold of 0.2 for *P*(TSG) and *P*(OCG) to call genes with tumour suppressor gene properties and oncogene properties, respectively (Supplementary Fig. 15).

### Expression information in predicting driver mutations

Some mutated genes are not expressed in cancer cells, and therefore the mutations in these genes are less likely to be pathogenic. Currently, the expression information has not yet been fully explored for the identification of driver mutations^58^, and only a few methods take expression information into account to assess the background mutation rates^3^.

We adopted a mixture modelling approach to predict whether a gene is ‘highly expressed' in a tumour type. We first log_2_ transform the tumour gene RNA-seq by Expectation Maximization (RSEM) abundance estimation values. To prevent taking the log_2_ of 0, we remove the gene expression values if they are ⩽ before log_2_-transformation. We then compute the 90th percentile of the expression of a gene across patients in a tumour type to represent the overall expression of that gene. Here we use the 90th percentile instead of median considering the gene dosage effects of copy number deletions on expression in cancer. It is unlikely that a gene is deleted in 90% of all the analysed samples (for example, for the Pan-Cancer data sets, the mostly frequently homozygously deleted gene is *CDKN2A*, which is deleted in 57% of patients in GBM). If we also consider heterozygous deletions, then the most frequently deleted gene is *EBF3*, which is deleted in 90% of patients in GBM. The 90th percentile expression of a gene may overestimate the expression level of the gene in the studied tumour type (since we are more concerned about losing important genes). Next, we model the 90th percentile expression of genes as a mixture of two Gaussian distributions: one component representing ‘highly expressed' and the other component representing ‘lowly expressed'. A gene is considered to be ‘highly expressed' if its posterior probability in the ‘highly expressed' group is ≥0.8. In the presence of outliers (some genes are expressed at extremely high or low levels), we first remove outliers based on the boxplot rule, and then fit the data. We assign a posterior probability of 1 to the highly expressed outliers, and a posterior probability of 0 to the lowly expressed outliers. Supplementary Figure 35 shows Gaussian mixture modelling of expression across the 12 cancer types.

We note parenthetically that another approach to prevent taking log_2_ of 0 is to add a small number, for example, 0.5 to the RSEM abundance estimation values before log_2_ transformation^59^. As we use 90th percentile of the expression of a gene across patients to represent the expression of that gene, this approach may result in many lowly expressed genes to have exactly the same expression value (Supplementary Fig. 36a, gene expression data from acute myeloid leukaemia). Consequently, a mixture of Gaussian distributions may not fit the data well since it is rare to observe exact the same value from a continuous distribution. Despite this limitation, posterior probabilities computed from both approaches are highly correlated as can be seen from the scatter plots in Supplementary Fig. 36b (Pearson correlation coefficient of 0.984, Spearman correlation coefficient of 0.995).

### Compensating for the *cis*-effects of copy number alterations

Before analysing the *trans*-effects of somatic mutations, we first remove the *cis*-effects of copy number alterations on expression; copy number alterations are common in cancer and the majority have *cis*-effects on expression. Numerous studies have carried out integrative analysis of copy number and gene expression data^60^,^61^,^62^,^63^. Here we use the Gaussian process (GP) regression to model the expression *y*_*i*_ of a gene in a patient *i*, as a function of its copy number log2 value *x*_*i*_. GP regression is flexible to add extra variables such as DNA methylation data as independent variables if necessary, and can capture nonlinear relationships between copy number alterations and expression.

GP regression models the joint distribution of *y*_*i*_ as a joint Gaussian distribution. The covariance matrix is constructed based on the given copy number data, cov(*x*_*i*_,*x*_*j*_)=*k*(*x*_*i*_,*x*_*j*_), where *k* is the squared exponential kernel function. The hyperparameters of the kernel function are computed by optimizing the log-marginal likelihood function using scaled conjugate gradient algorithms. To remove the *cis*-effects of copy number alterations, we subtract the regression values from the original expression values to get the residuals that are considered to be regulated by *trans*-effect mutations. Supplementary Figure 37 shows the scatter plots of copy number alteration and expression values for *PTEN* across the 12 cancer types. The GP regression lines and the 95% confidence intervals of the regression lines are overlaid on the scatter plots. Supplementary Figure 38 shows the scatter plots of *PTEN* expression across cancer types after removing the *cis*-effects of copy number alterations on expression.

## Additional information

**How to cite this article:** Ding, J. *et al*. Systematic analysis of somatic mutations impacting gene expression in 12 tumour types. *Nat. Commun*. 6:8554 doi: 10.1038/ncomms9554 (2015).

## Supplementary Material

Supplementary Information Supplementary Figures 1-38, Supplementary Tables 1-8, Supplementary Discussion, Supplementary Methods and Supplementary References

Supplementary Data 1 The collected 603 bona fide driver genes. These genes are either from Cancer Gene Census^25^ or reported in recent studies^1^,^26^.

Supplementary Data 2 The 65 xseq predicted genes with cis-effect loss-of-function mutations.

Supplementary Data 3 The 131 candidate tumour suppressor genes and their P(TSG) probabilities.

Supplementary Data 4 The 60 xseq predicted trans-effect genes. These trans-effect genes were annotated as bona fide cancer driver genes.

Supplementary Data 5 The 89 novel xseq predicted trans-effect genes. The 89 novel xseq pre-dicted trans-effect genes which were not significantly mutated6 and were not annotated as bona fide cancer driver genes.

Supplementary Data 6 Enriched pathways and gene ontology terms in the novel trans-effect genes.

Supplementary Data 7 Genes stably up-regulated associated with mutations across tumour types.

Supplementary Data 8 Genes stably down-regulated associated with mutations across tumour types.

## Figures and Tables

**Table 1:** List of the twelve cancer types analysed.

Data	Mutation	RNASeq	SNP6.0	Overlap
BLCA	99	96	125	94
BRCA	772	822	879	743
COAD	155	192	414	149
GBM	291	167	576	144
HNSC	306	303	306	295
KIRC	417	428	452	390
LAML	196	173	197	167
LUAD	230	355	358	169
LUSC	178	220	342	177
OV	316	266	581	159
READ	69	71	163	65
UCEC	248	333	493	235

BLCA, bladder urothelial carcinoma; BRCA, breast invasive carcinoma; COAD, colon adenocarcinoma; GBM, glioblastoma multiforme; HNSC, head and neck squamous cell carcinoma; KIRC, kidney renal clear cell carcinoma; LAML, acute myeloid leukaemia, also denoted as AML; LUAD, lung adenocarcinoma; LUSC, lung squamous cell carcinoma; OV, ovarian serous cystadenocarcinoma; READ, rectum adenocarcinoma; UCEC, uterine corpus endometrioid carcinoma.

The numbers are the sample counts. Totally, 563,024 somatic mutations in the overlapped samples (363,676 missense mutations, 132,981 synonymous mutations, 33,838 nonsense mutations, 13,260 frameshift indels, 6,952 non-coding RNA mutations, 8,699 splice site mutations, 3,141 in-frame indels and 477 stop gain mutations). In *trans*-analysis, we added the 37,308 homozygous deletions in 2,084 genes (focal copy number deletion peaks), and 69,643 amplifications in 960 genes (focal copy number amplification peaks).
