# Supplementary material for: Systematic analysis of somatic mutations impacting gene expression in 12 tumour types
Source: Nat Commun. 2015 Oct 5;6:8554. doi: 10.1038/ncomms9554 (PMC4600750; doi:10.1038/ncomms9554)
Supplement: Supplementary Information — Supplementary Figures 1-38, Supplementary Tables 1-8, Supplementary Discussion, Supplementary Methods and Supplementary References [file ncomms9554-s1.pdf]

# Supplementary Figures

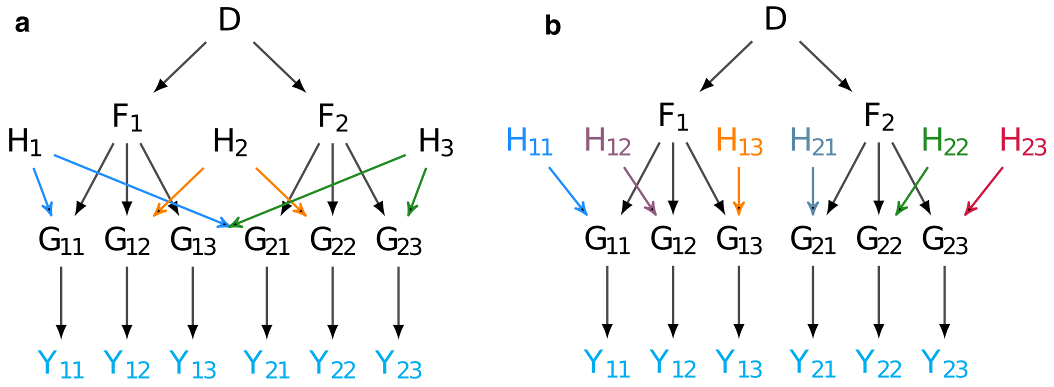


Supplementary Fig. 1: A simple `xseq` model. This `xseq` model with just 1 mutated gene $g$, 2 patients harbouring mutations in gene $g$ and 3 genes connected to $g$. Here we drop '$g$' in the figure for simplicity. (a) $H$ is hidden and (b) $H$ is observed.

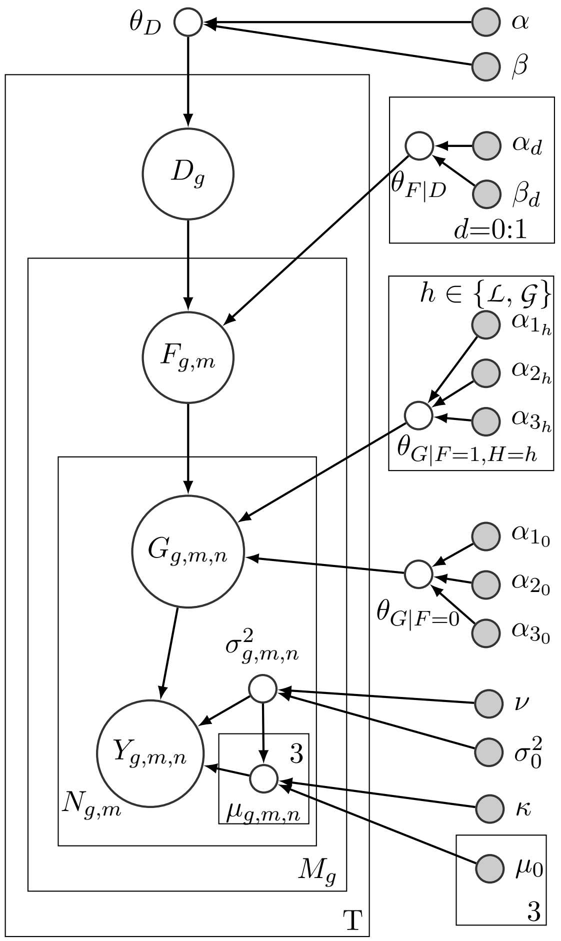


Supplementary Fig. 2: The `xseq` model used to sample $T$ mutated genes. The $g$th gene has $M_g$ mutations. Of the $m$th patient with a mutation in gene $g$, $N_{g,m}$ genes connected to $g$. Here the shaded nodes represent observed variables and the unshaded nodes represent hidden variables.

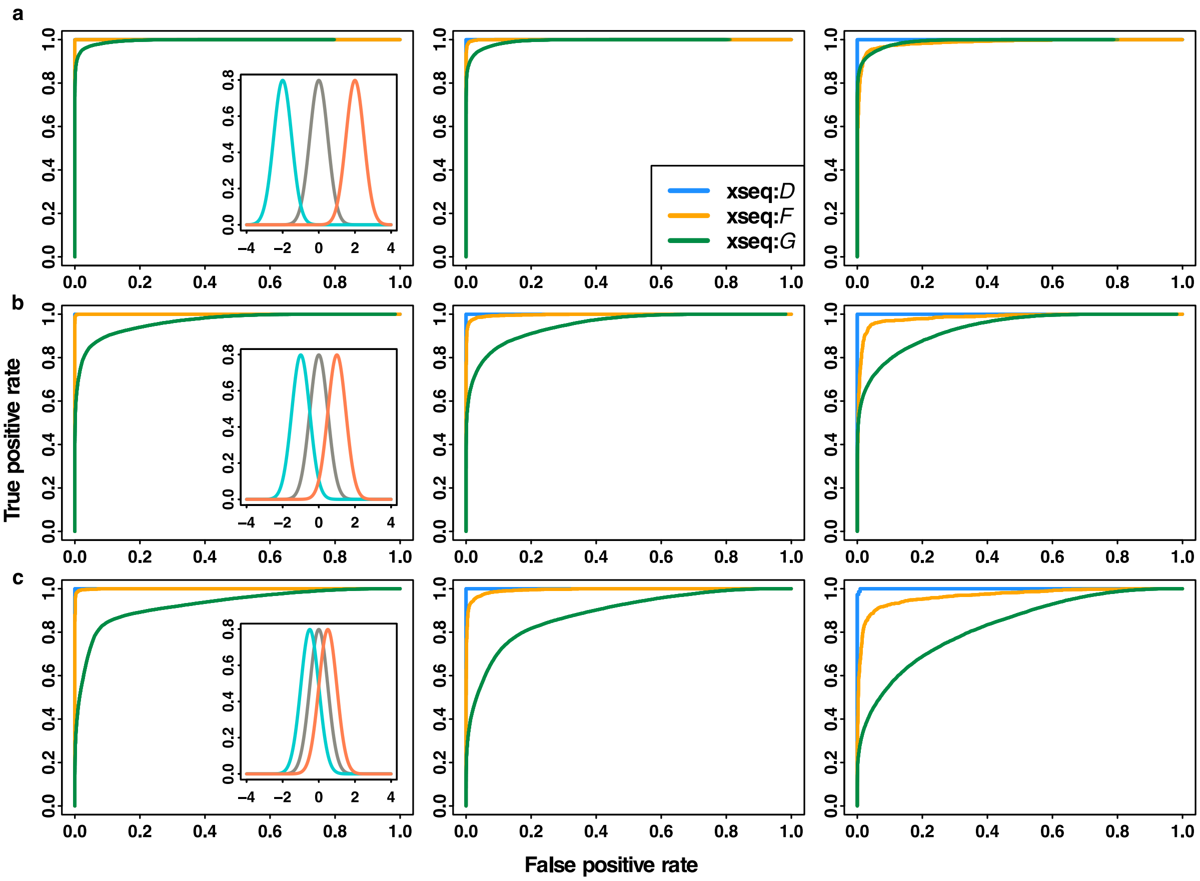


Supplementary Fig. 3: The `xseq` prediction ROC curves by given the true $H$ values. (a) The expression of genes which are down-regulated, neutral and up-regulated is highly discriminative (first row), (b) moderately discriminative (second row) and (c) poorly discriminative (third row, see the enclosed figures, where cyan is down-regulation, grey is neutral and red is up-regulation, respectively). The ROC curves in the first column, second column and the third column were computed when the degree of dysregulation of the expression of connected genes by mutations was high, moderate and low, respectively.

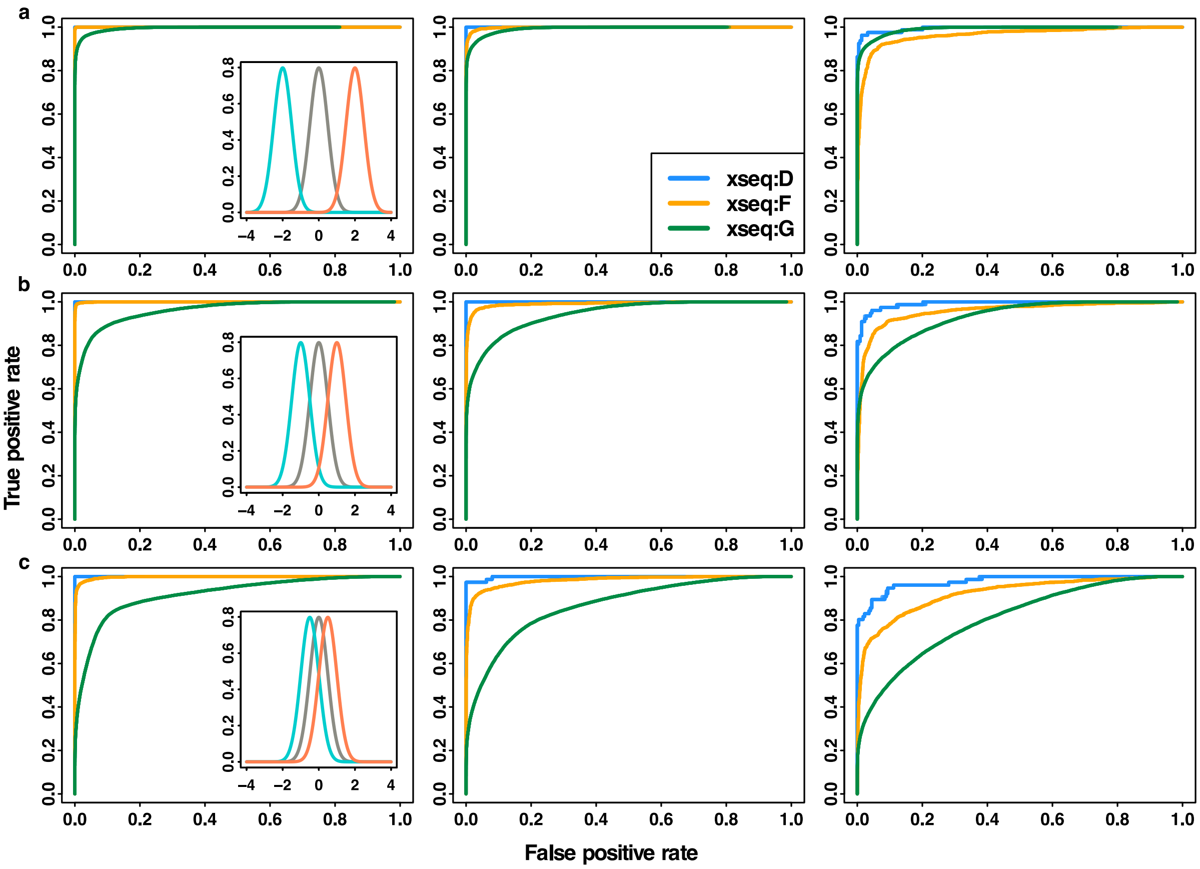


Supplementary Fig. 4: The `xseq-simple` model prediction ROC curves from different simulated datasets. (a) The expression of genes which are down-regulated, neutral and up-regulated is highly discriminative (first row), (b) moderately discriminative (second row) and (c) poorly discriminative (third row, see the enclosed figures, where blue is down-regulation, grey is neutral and red is up-regulation, respectively). The ROC curves in the first column, second column and the third column were computed when the degree of dysregulation of the expression of connected genes by mutations was high, moderate and low, respectively.

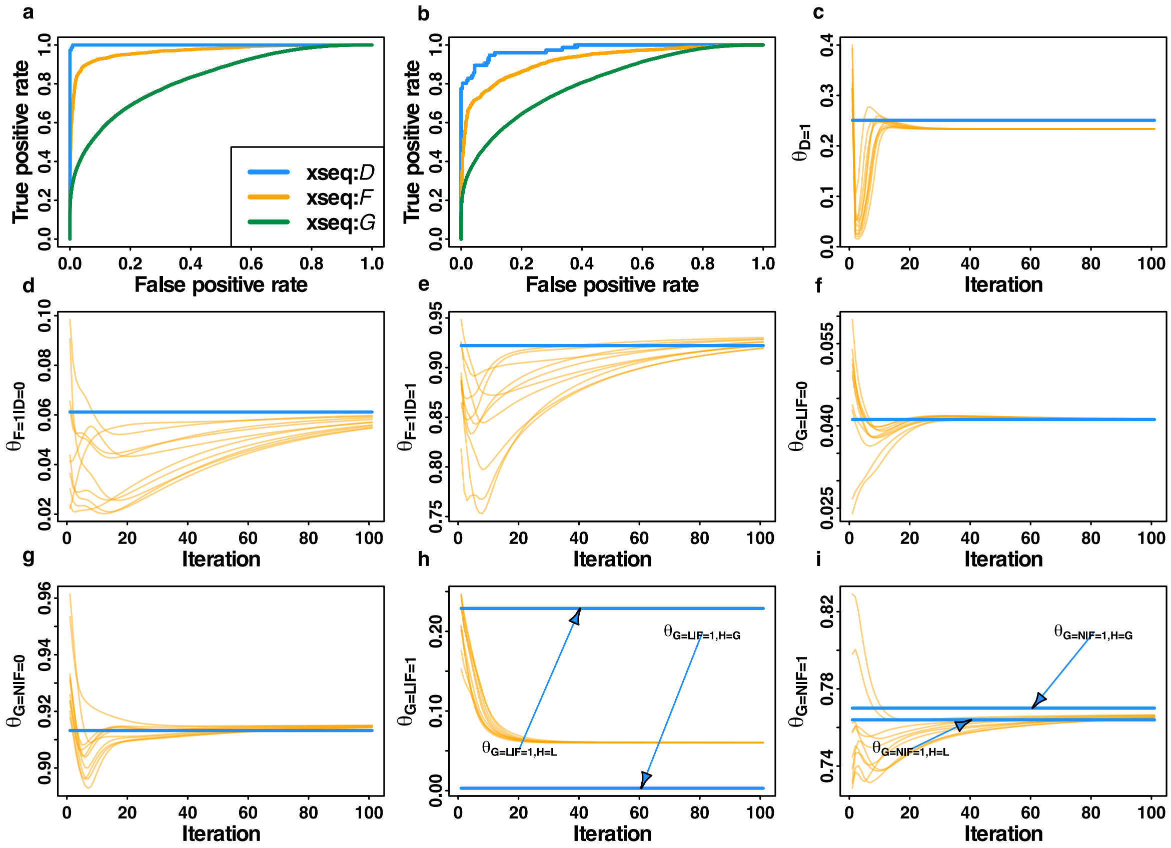


Supplementary Fig. 5: `xseq-simple` parameter trace plots during EM-iterations for the most challenging case. (a) The ROC curves of the predictions based on the true parameters (b) The ROC curves of `xseq-simple` predictions based on the parameters learned from the EM algorithm. (c)-(i) The trace plots of `xseq-simple` parameters during EM iterations.

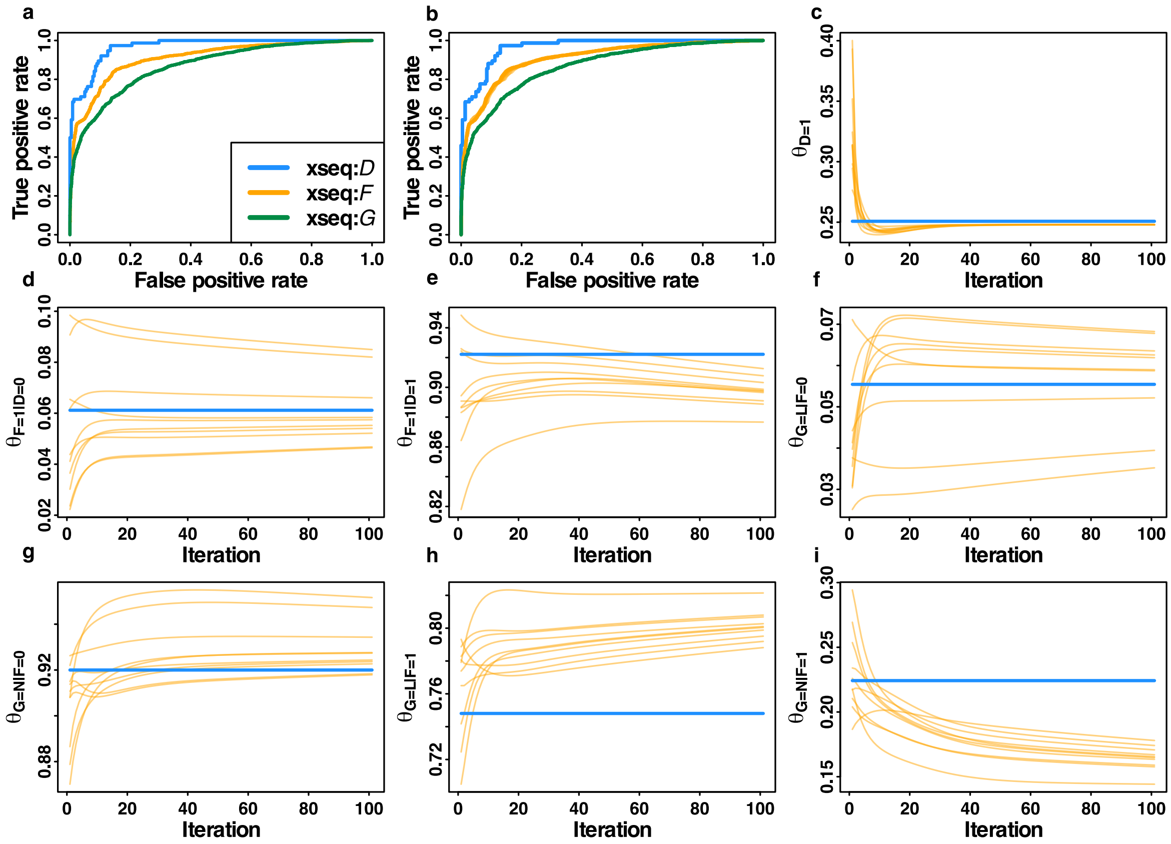


Supplementary Fig. 6: `xseq-simple` parameter trace plots for simulation analyses of mutation cis-effects. (a) The ROC curves of the predictions based on the true parameters (b) The ROC curves of `xseq-simple` predictions based on the parameters learned from the EM algorithm. (c)-(i) The trace plots of `xseq-simple` parameters during EM iterations.

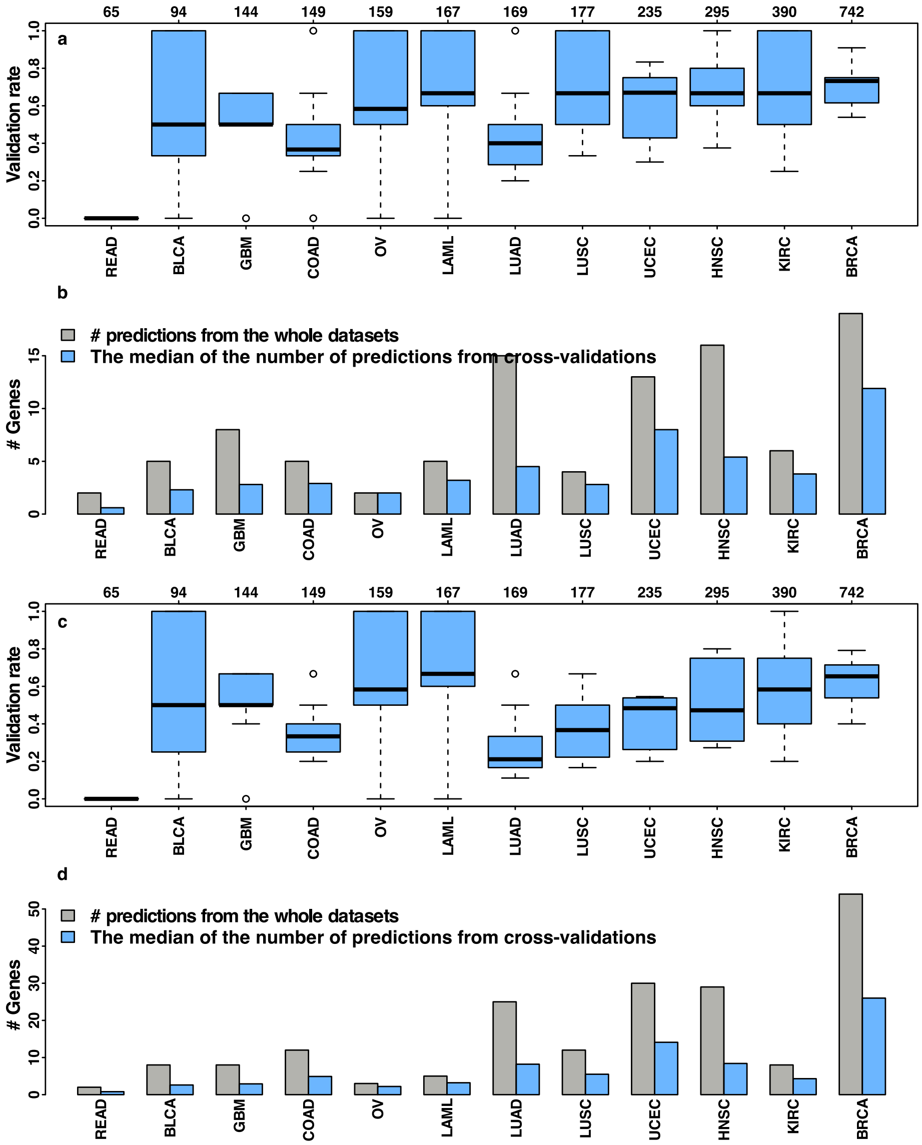


Supplementary Fig. 7: The validation rates from repeated cross-validation experiments. (a) The validation rates for *bona fide* cancer genes from different types of tumours (b) Comparison of the median number of predicted *bona fide* cancer genes from cross-validation experiments with the number of predicted *bona fide* cancer genes from analyzing the original complete The Cancer Genome Atlas (TCGA) datasets (c) The validation rates for all predicted genes across different types of tumours (d) Comparison of the median number of all predicted genes from cross-validation experiments with the number of predicted genes from the original compete TCGA datasets.

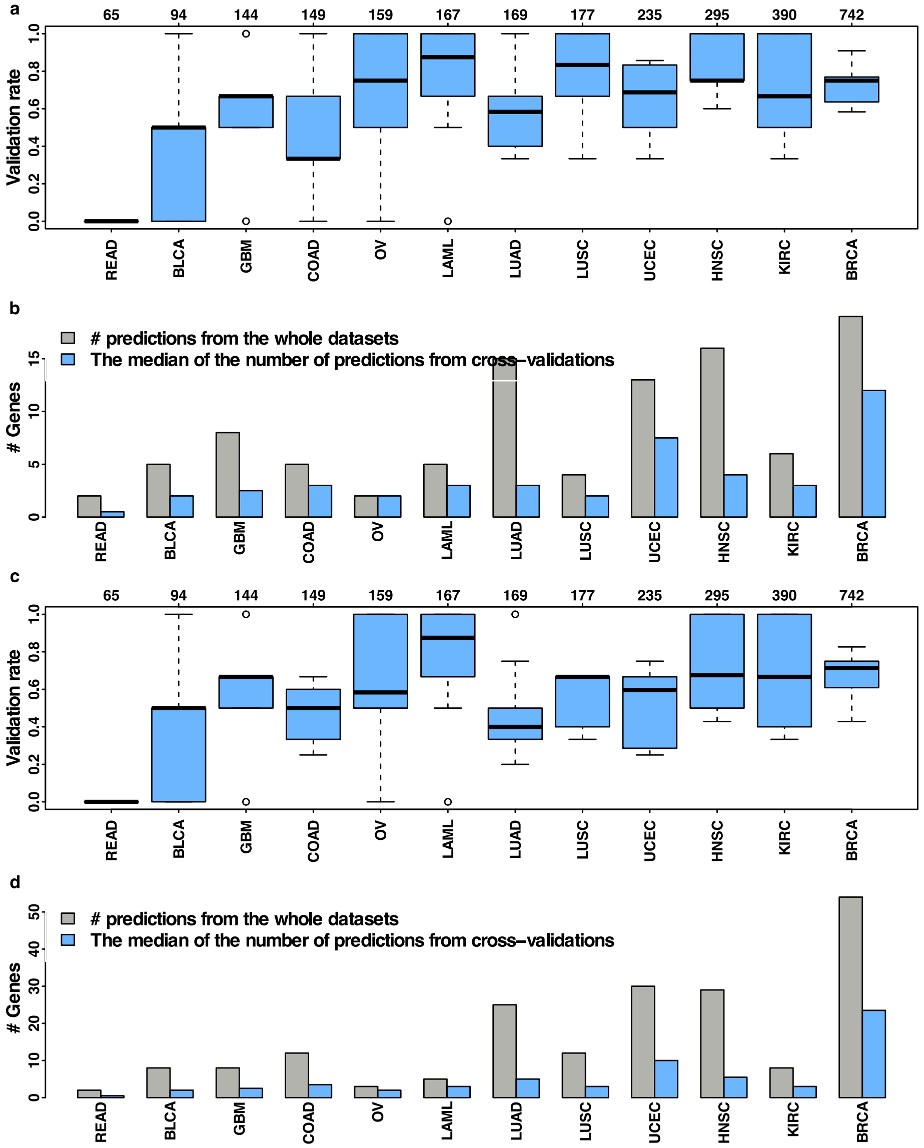


Supplementary Fig. 8: The validation rates from 10-repeated cross-validation experiments after filtering out rarely mutated genes. In summering results, we only kept the genes that had at least 5 mutations in both the discovery and validation datasets. (a) The validation rates for *bona fide* cancer genes from different types of tumours (b) Comparison of the median number of predicted *bona fide* cancer genes from cross-validation experiments with the number of predicted *bona fide* cancer genes from analyzing the original complete TCGA datasets (c) The validation rates for all predicted genes across different types of tumours (d) Comparison of the median number of all predicted genes from cross-validation experiments with the number of predicted genes from the original compete TCGA datasets.

Supplementary Fig. 9: Trans-effects of copy number alterations of genes in the TCGA breast cancer dataset. Heatmaps show the expression of the TCGA breast cancer genes connected to the 14 high probability genes predicted in both TCGA and Molecular Taxonomy of Breast Cancer International Consortium (METABRIC) datasets.

## BRCA_MYC

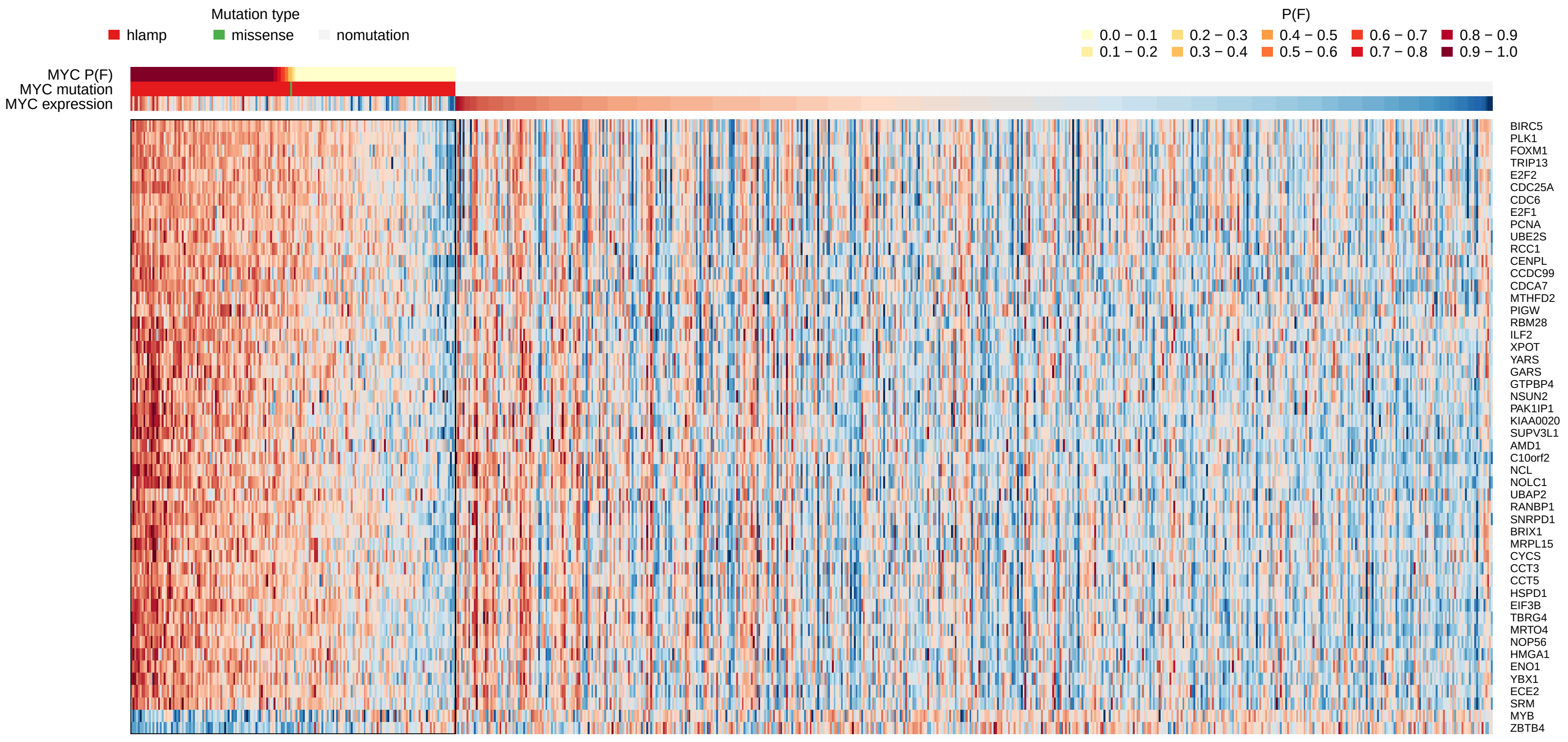

Mutation type
hlamp
missense
nomutation
P(F)
0.0 − 0.1
0.1 − 0.2
0.2 − 0.3
0.3 − 0.4
0.4 − 0.5
0.5 − 0.6
0.6 − 0.7
0.7 − 0.8
0.8 − 0.9
0.9 − 1.0
MYC P(F)
MYC mutation
MYC expression
BIRC5
PLK1
FOXM1
TRIP13
E2F2
CDC25A
CDC6
E2F1
PCNA
UBE2S
RCC1
CENPL
CCDC99
CDCA7
MTHFD2
PIGW
RBM28
ILF2
XPOT
YARS
GARS
GTPBP4
NSUN2
PAK1IP1
KIAA0020
SUPV3L1
AMD1
C10orf2
NCL
NOLC1
UBAP2
RANBP1
SNRPD1
BRIX1
MRPL15
CYCS
CCT3
CCT5
HSPD1
EIF3B
TBRG4
MRTO4
NOP56
HMGA1
ENO1
YBX1
ECE2
SRM
MYB
ZBTB4

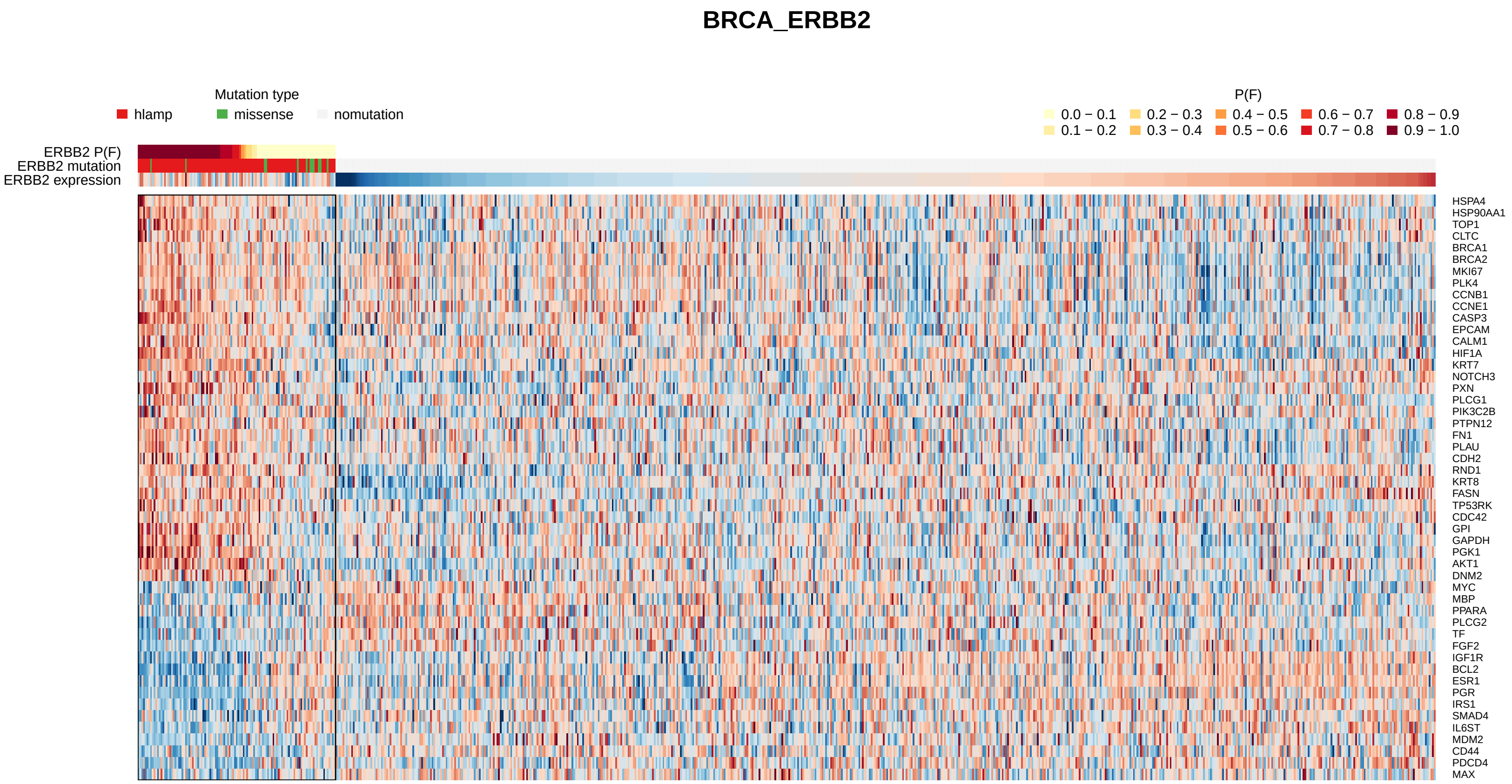

BRCA_ERBB2
Mutation type
hlamp
missense
nomutation
P(F)
0.0 − 0.1
0.1 − 0.2
0.2 − 0.3
0.3 − 0.4
0.4 − 0.5
0.5 − 0.6
0.6 − 0.7
0.7 − 0.8
0.8 − 0.9
0.9 − 1.0
ERBB2 P(F)
ERBB2 mutation
ERBB2 expression
HSPA4
HSP90AA1
TOP1
CLTC
BRCA1
BRCA2
MKI67
PLK4
CCNB1
CCNE1
CASP3
EPCAM
CALM1
HIF1A
KRT7
NOTCH3
PXN
PLCG1
PIK3C2B
PTPN12
FN1
PLAU
CDH2
RND1
KRT8
FASN
TP53RK
CDC42
GPI
GAPDH
PGK1
AKT1
DNM2
MYC
MBP
PPARA
PLCG2
TF
FGF2
IGF1R
BCL2
ESR1
PGR
IRS1
SMAD4
IL6ST
MDM2
CD44
PDCD4
MAX

# BRCA_CCNE1

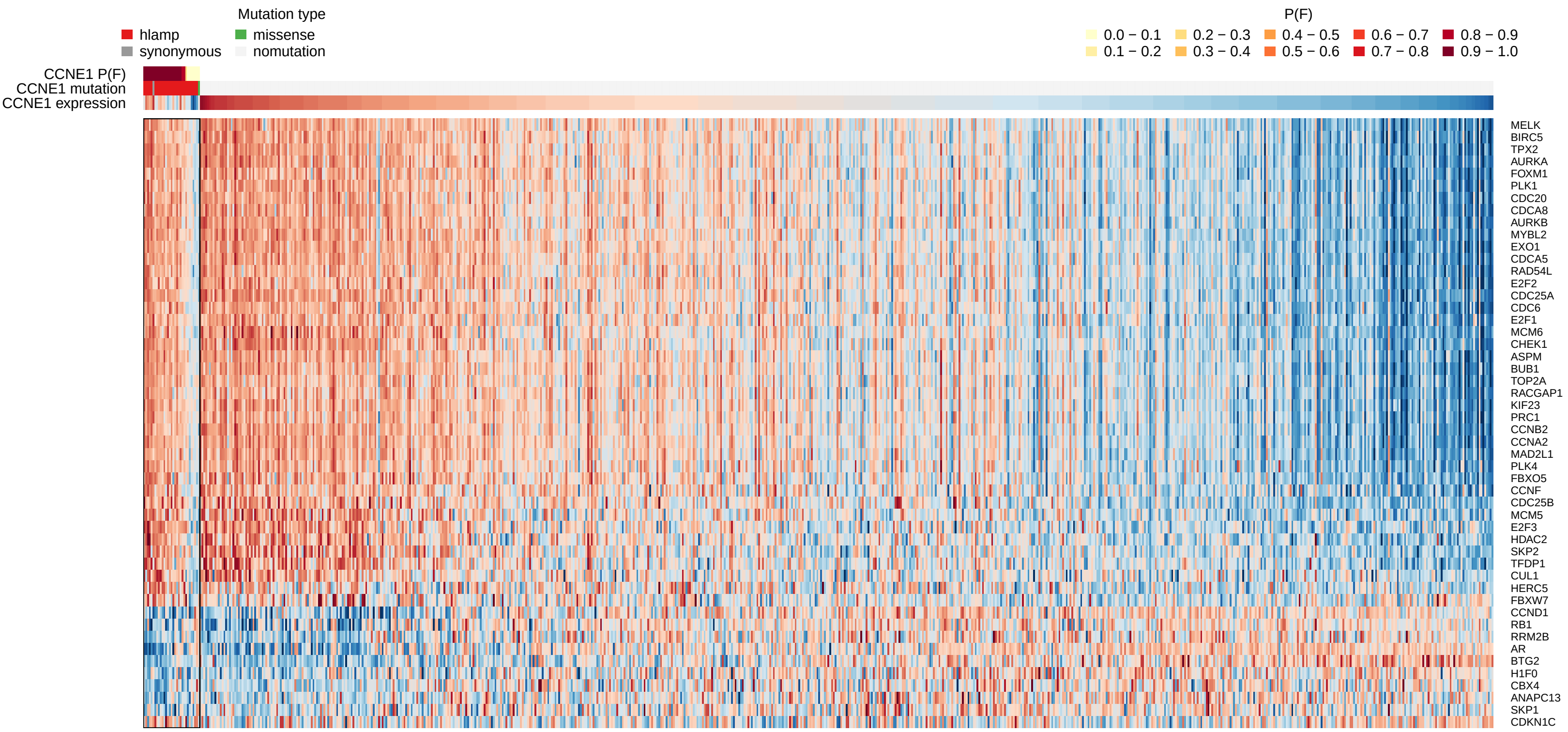
Mutation type
hlamp
synonymous
missense
nomutation
P(F)
0.0 − 0.1
0.1 − 0.2
0.2 − 0.3
0.3 − 0.4
0.4 − 0.5
0.5 − 0.6
0.6 − 0.7
0.7 − 0.8
0.8 − 0.9
0.9 − 1.0
CCNE1 P(F)
CCNE1 mutation
CCNE1 expression
MELK
BIRC5
TPX2
AURKA
FOXM1
PLK1
CDC20
CDCA8
AURKB
MYBL2
EXO1
CDCA5
RAD54L
E2F2
CDC25A
CDC6
E2F1
MCM6
CHEK1
ASPM
BUB1
TOP2A
RACGAP1
KIF23
PRC1
CCNB2
CCNA2
MAD2L1
PLK4
FBXO5
CCNF
CDC25B
MCM5
E2F3
HDAC2
SKP2
TFDP1
CUL1
HERC5
FBXW7
CCND1
RB1
RRM2B
AR
BTG2
H1F0
CBX4
ANAPC13
SKP1
CDKN1C

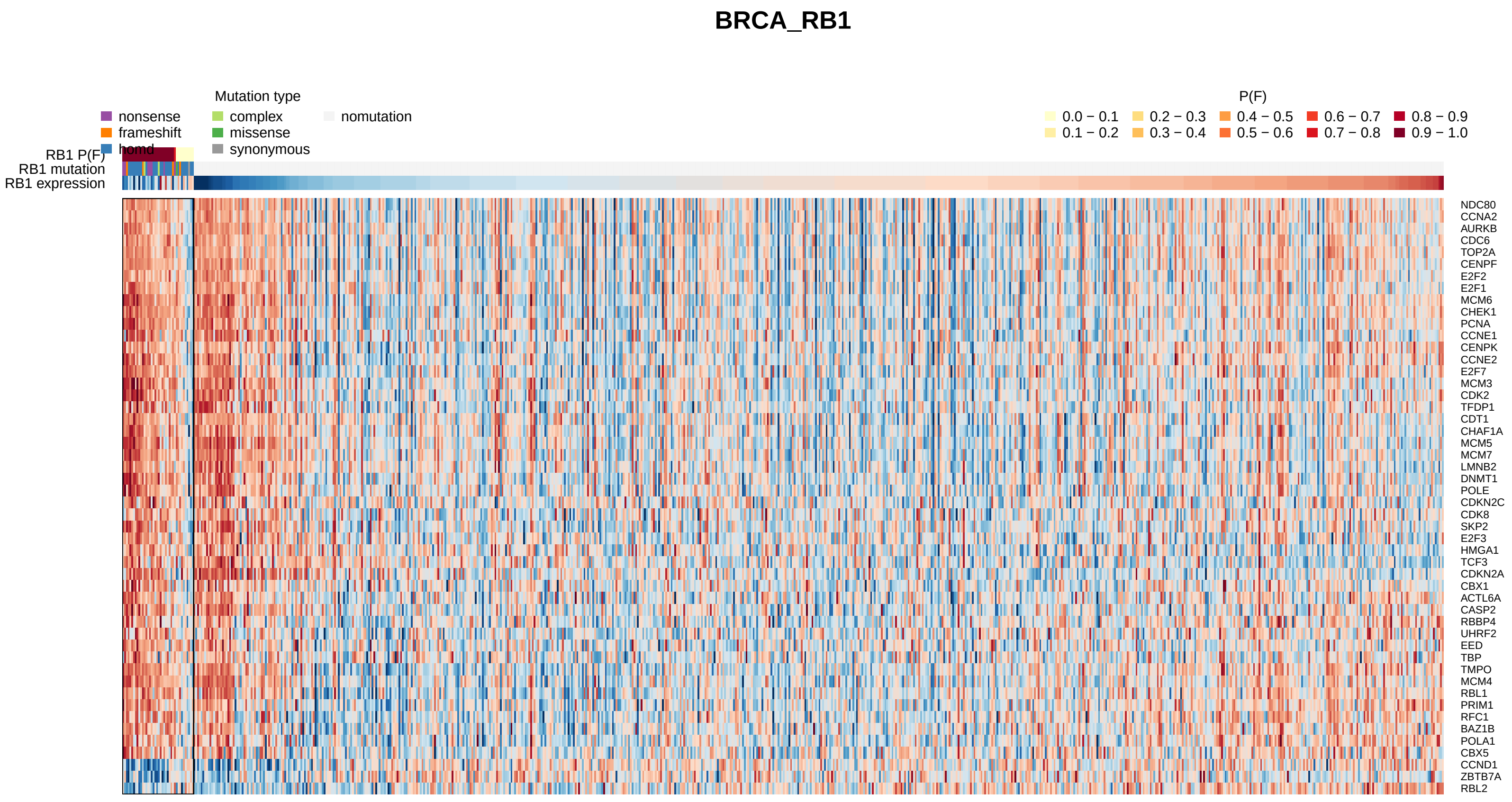
BRCA_RB1
Mutation type
nonsense
frameshift
homd
complex
missense
synonymous
nomutation
P(F)
0.0 − 0.1
0.1 − 0.2
0.2 − 0.3
0.3 − 0.4
0.4 − 0.5
0.5 − 0.6
0.6 − 0.7
0.7 − 0.8
0.8 − 0.9
0.9 − 1.0
RB1 P(F)
RB1 mutation
RB1 expression
NDC80
CCNA2
AURKB
CDC6
TOP2A
CENPF
E2F2
E2F1
MCM6
CHEK1
PCNA
CCNE1
CENPK
CCNE2
E2F7
MCM3
CDK2
TFDP1
CDT1
CHAF1A
MCM5
MCM7
LMNB2
DNMT1
POLE
CDKN2C
CDK8
SKP2
E2F3
HMGA1
TCF3
CDKN2A
CBX1
ACTL6A
CASP2
RBBP4
UHRF2
EED
TBP
TMPO
MCM4
RBL1
PRIM1
RFC1
BAZ1B
POLA1
CBX5
CCND1
ZBTB7A
RBL2

# BRCA_CCND1

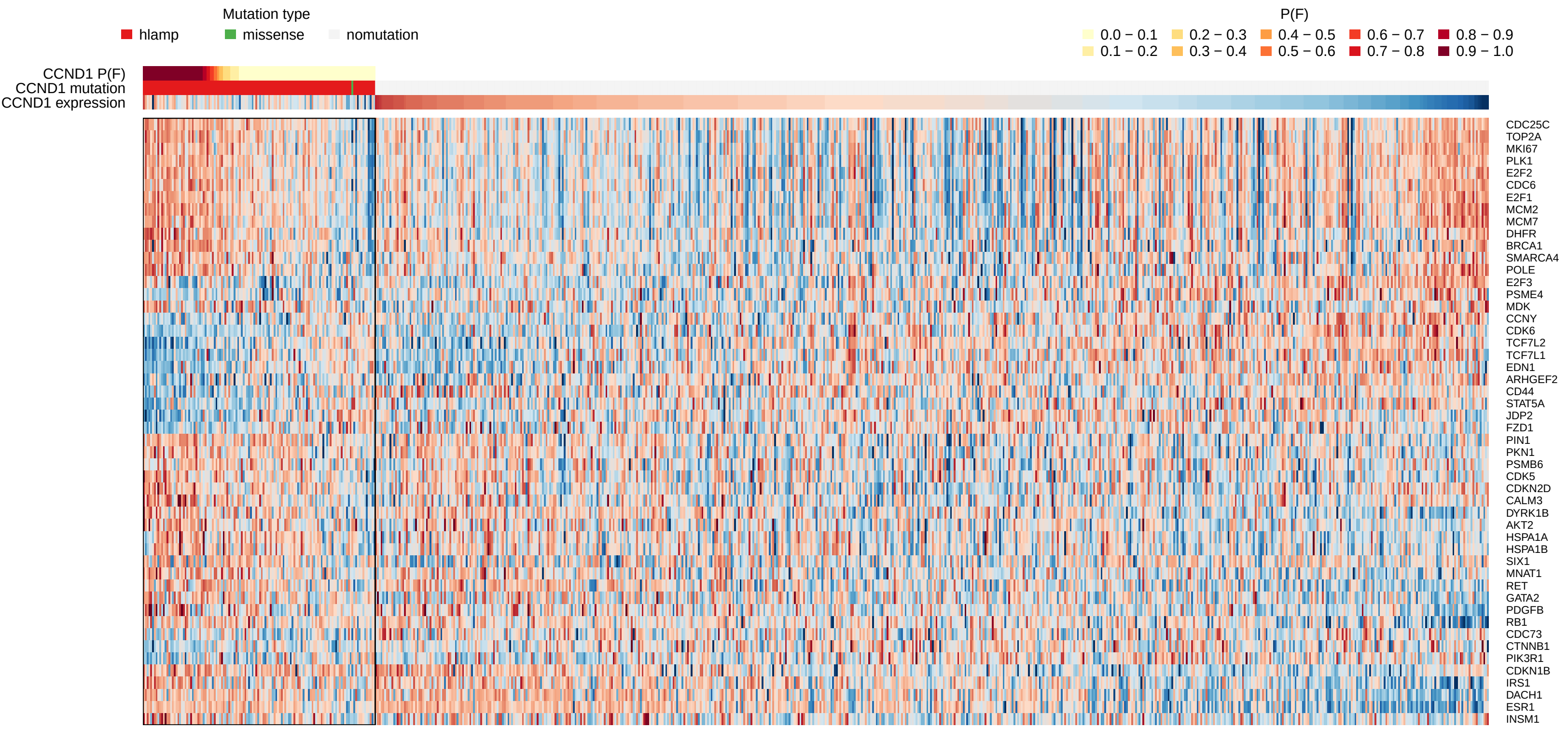

Mutation type
hlamp
missense
nomutation
P(F)
0.0 − 0.1
0.1 − 0.2
0.2 − 0.3
0.3 − 0.4
0.4 − 0.5
0.5 − 0.6
0.6 − 0.7
0.7 − 0.8
0.8 − 0.9
0.9 − 1.0
CCND1 P(F)
CCND1 mutation
CCND1 expression
CDC25C
TOP2A
MKI67
PLK1
E2F2
CDC6
E2F1
MCM2
MCM7
DHFR
BRCA1
SMARCA4
POLE
E2F3
PSME4
MDK
CCNY
CDK6
TCF7L2
TCF7L1
EDN1
ARHGEF2
CD44
STAT5A
JDP2
FZD1
PIN1
PKN1
PSMB6
CDK5
CDKN2D
CALM3
DYRK1B
AKT2
HSPA1A
HSPA1B
SIX1
MNAT1
RET
GATA2
PDGFB
RB1
CDC73
CTNNB1
PIK3R1
CDKN1B
IRS1
DACH1
ESR1
INSM1

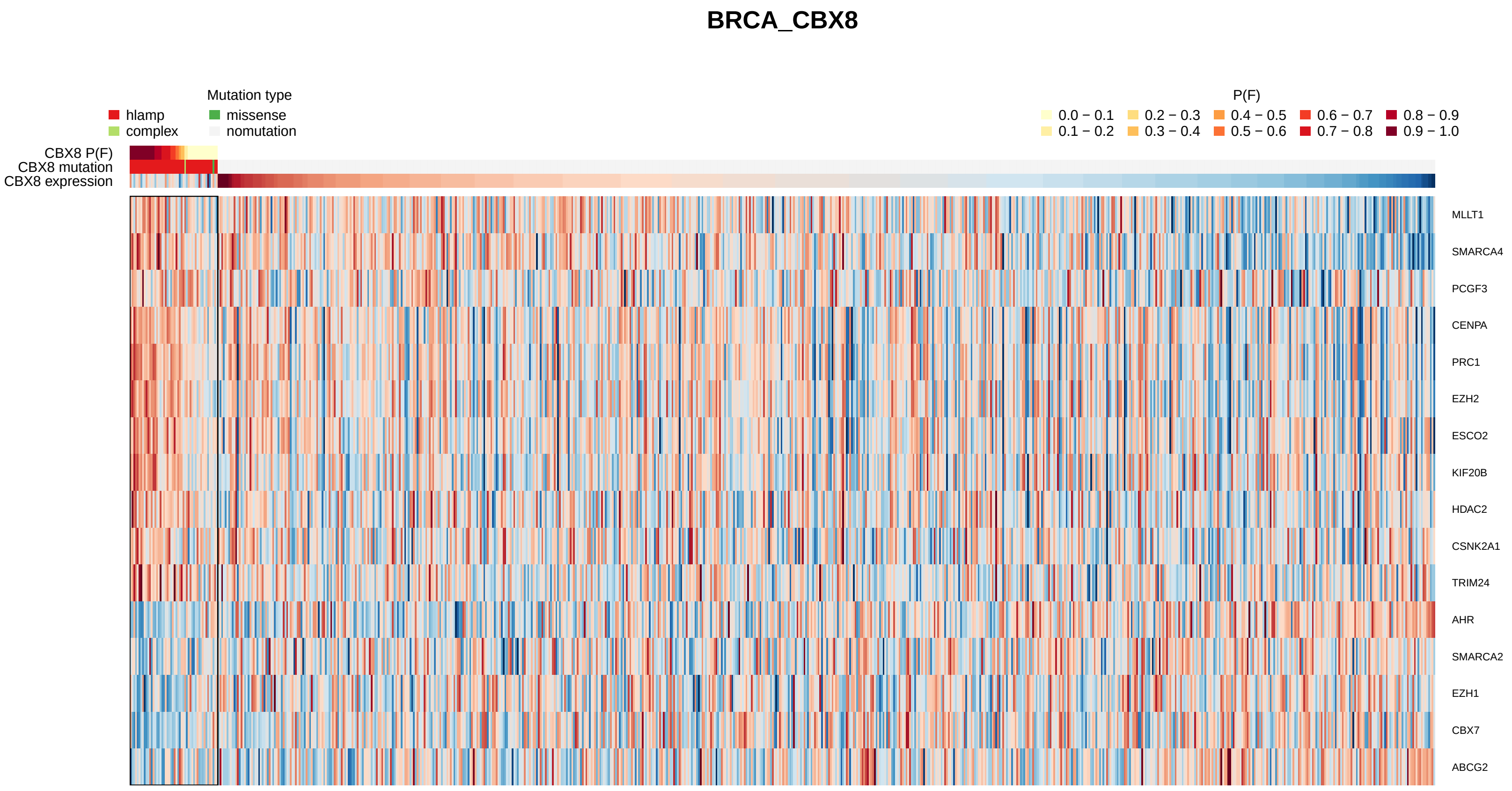
BRCA_CBX8
Mutation type
hlamp
complex
missense
nomutation
P(F)
0.0 − 0.1
0.1 − 0.2
0.2 − 0.3
0.3 − 0.4
0.4 − 0.5
0.5 − 0.6
0.6 − 0.7
0.7 − 0.8
0.8 − 0.9
0.9 − 1.0
CBX8 P(F)
CBX8 mutation
CBX8 expression
MLLT1
SMARCA4
PCGF3
CENPA
PRC1
EZH2
ESCO2
KIF20B
HDAC2
CSNK2A1
TRIM24
AHR
SMARCA2
EZH1
CBX7
ABCG2

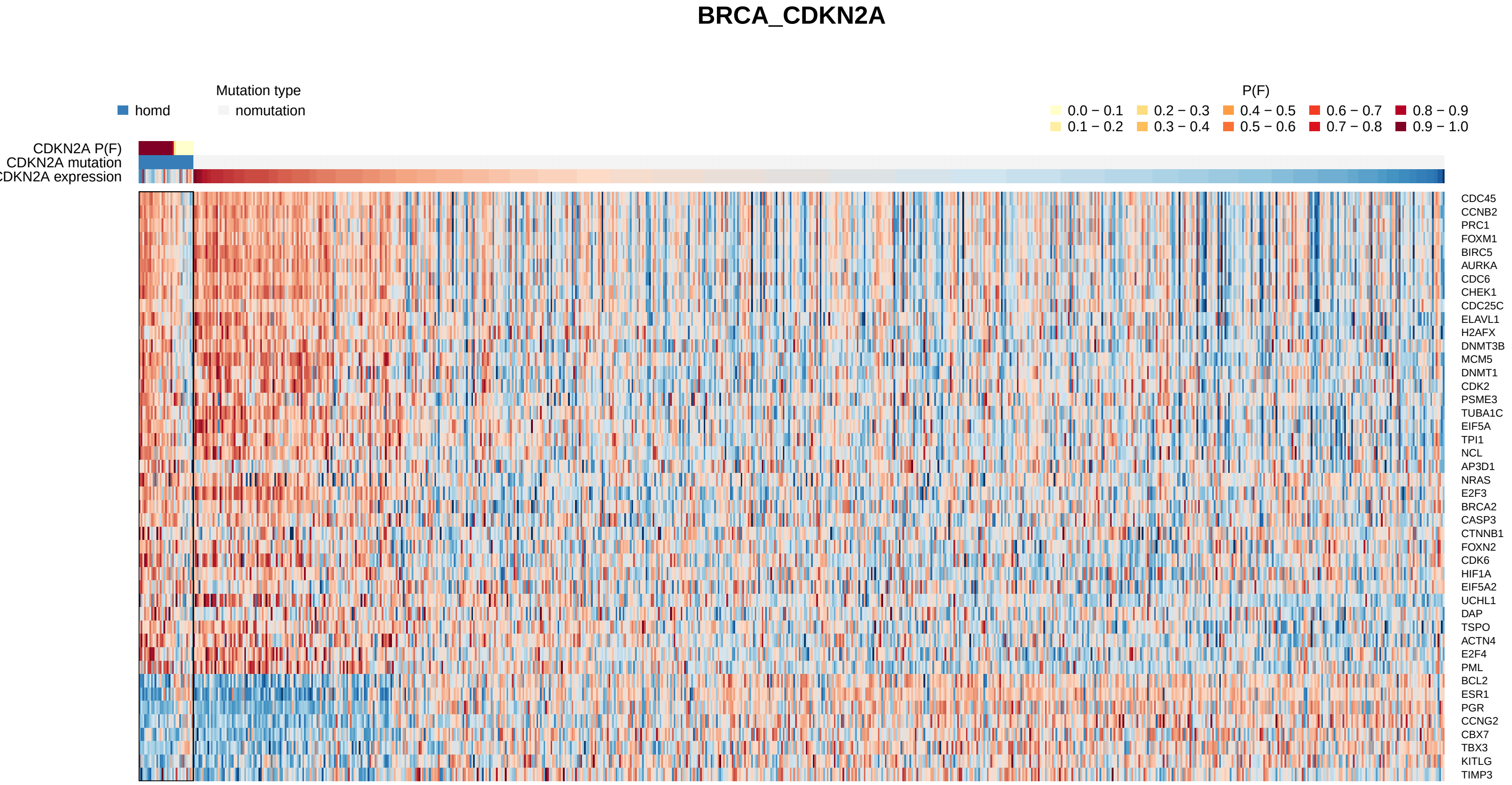
BRCA_CDKN2A
Mutation type
homd
nomutation
P(F)
0.0 − 0.1
0.1 − 0.2
0.2 − 0.3
0.3 − 0.4
0.4 − 0.5
0.5 − 0.6
0.6 − 0.7
0.7 − 0.8
0.8 − 0.9
0.9 − 1.0
CDKN2A P(F)
CDKN2A mutation
CDKN2A expression
CDC45
CCNB2
PRC1
FOXM1
BIRC5
AURKA
CDC6
CHEK1
CDC25C
ELAVL1
H2AFX
DNMT3B
MCM5
DNMT1
CDK2
PSME3
TUBA1C
EIF5A
TPI1
NCL
AP3D1
NRAS
E2F3
BRCA2
CASP3
CTNNB1
FOXN2
CDK6
HIF1A
EIF5A2
UCHL1
DAP
TSPO
ACTN4
E2F4
PML
BCL2
ESR1
PGR
CCNG2
CBX7
TBX3
KITLG
TIMP3

## BRCA_E2F3

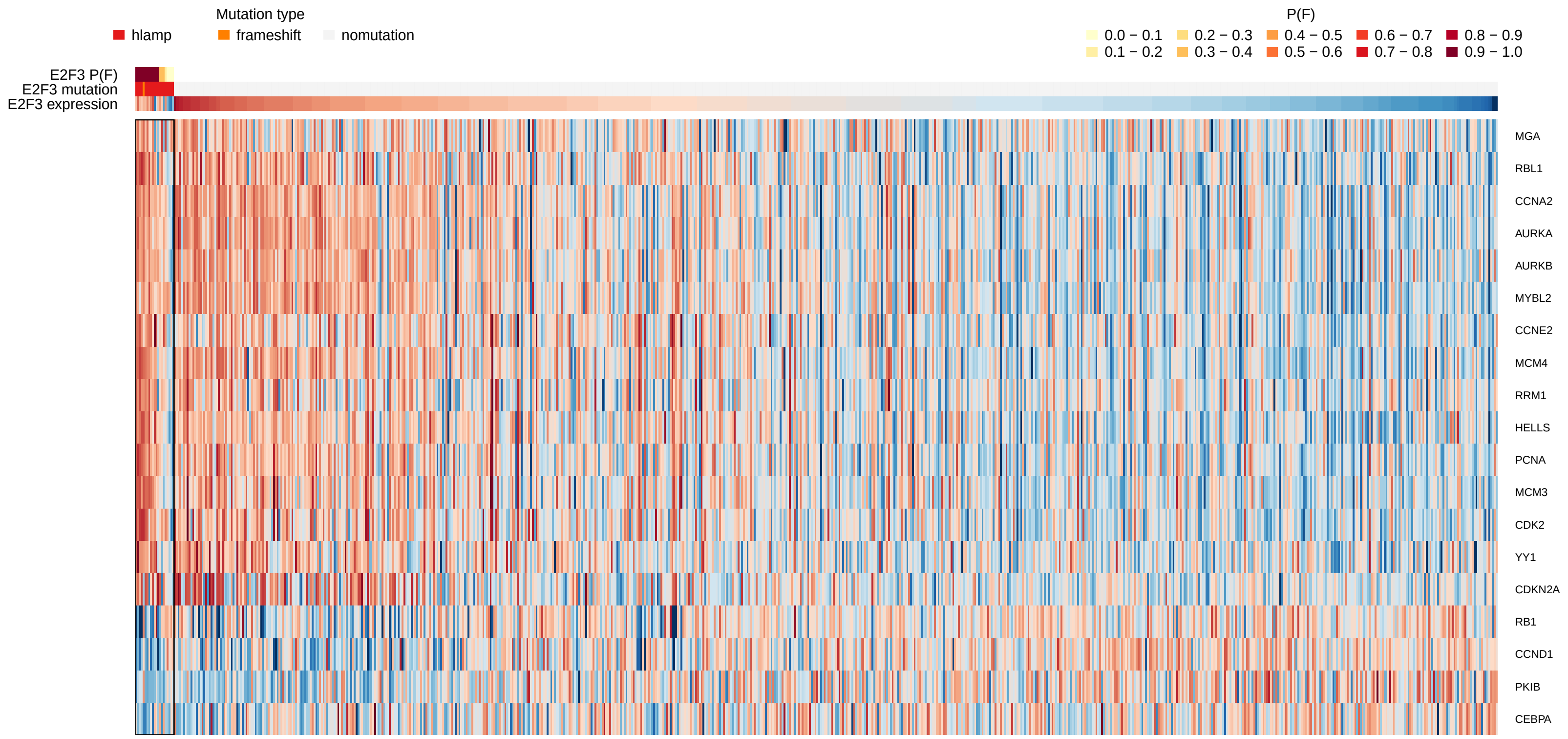

Mutation type
hlamp
frameshift
nomutation
P(F)
0.0 − 0.1
0.1 − 0.2
0.2 − 0.3
0.3 − 0.4
0.4 − 0.5
0.5 − 0.6
0.6 − 0.7
0.7 − 0.8
0.8 − 0.9
0.9 − 1.0
E2F3 P(F)
E2F3 mutation
E2F3 expression
MGA
RBL1
CCNA2
AURKA
AURKB
MYBL2
CCNE2
MCM4
RRM1
HELLS
PCNA
MCM3
CDK2
YY1
CDKN2A
RB1
CCND1
PKIB
CEBPA

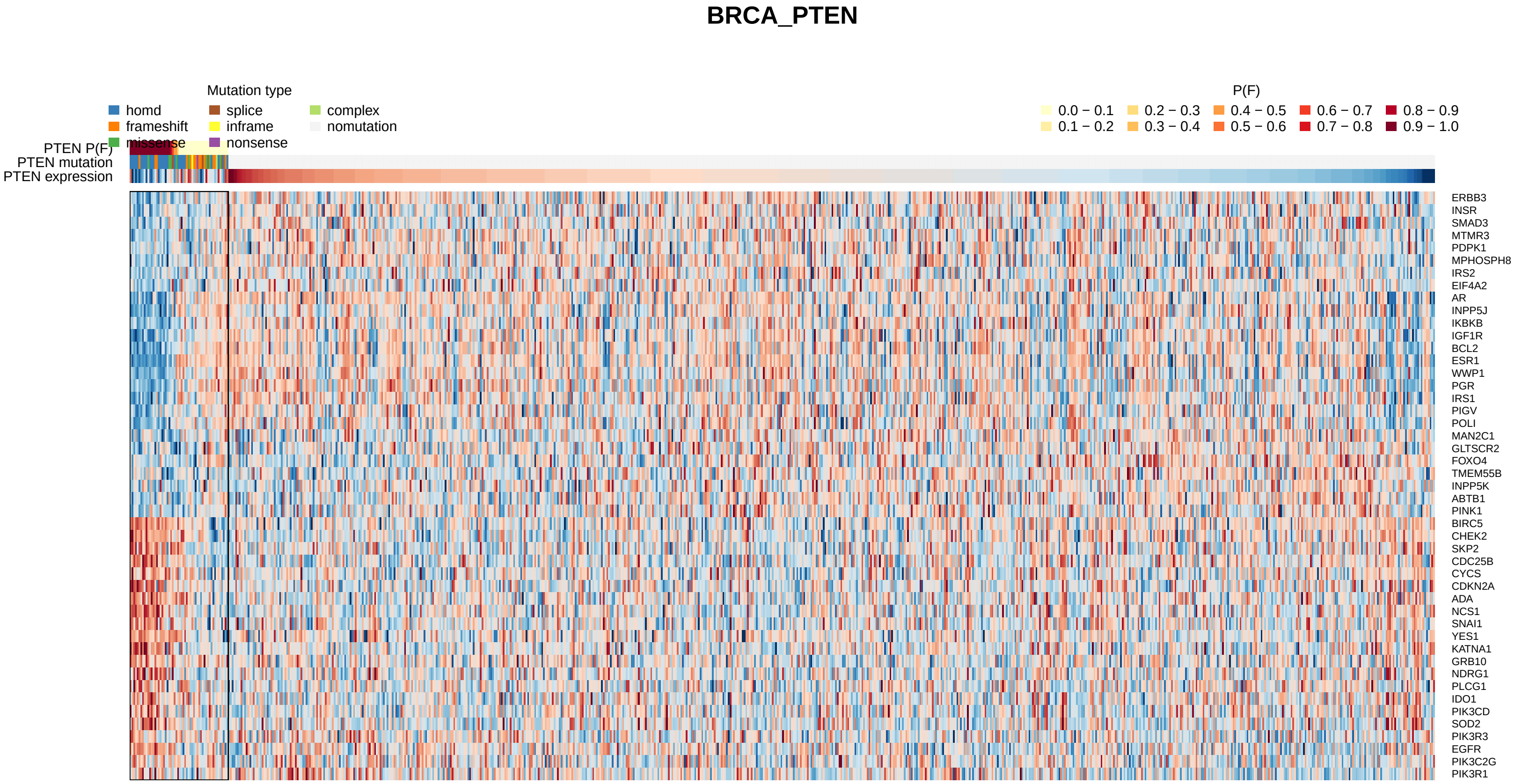
BRCA_PTEN
Mutation type
homd
frameshift
missense
splice
inframe
nonsense
complex
nomutation
P(F)
0.0 − 0.1
0.1 − 0.2
0.2 − 0.3
0.3 − 0.4
0.4 − 0.5
0.5 − 0.6
0.6 − 0.7
0.7 − 0.8
0.8 − 0.9
0.9 − 1.0
PTEN P(F)
PTEN mutation
PTEN expression
ERBB3
INSR
SMAD3
MTMR3
PDPK1
MPHOSPH8
IRS2
EIF4A2
AR
INPP5J
IKBKB
IGF1R
BCL2
ESR1
WWP1
PGR
IRS1
PIGV
POLI
MAN2C1
GLTSCR2
FOXO4
TMEM55B
INPP5K
ABTB1
PINK1
BIRC5
CHEK2
SKP2
CDC25B
CYCS
CDKN2A
ADA
NCS1
SNAI1
YES1
KATNA1
GRB10
NDRG1
PLCG1
IDO1
PIK3CD
SOD2
PIK3R3
EGFR
PIK3C2G
PIK3R1

# BRCA_PPP2R2A

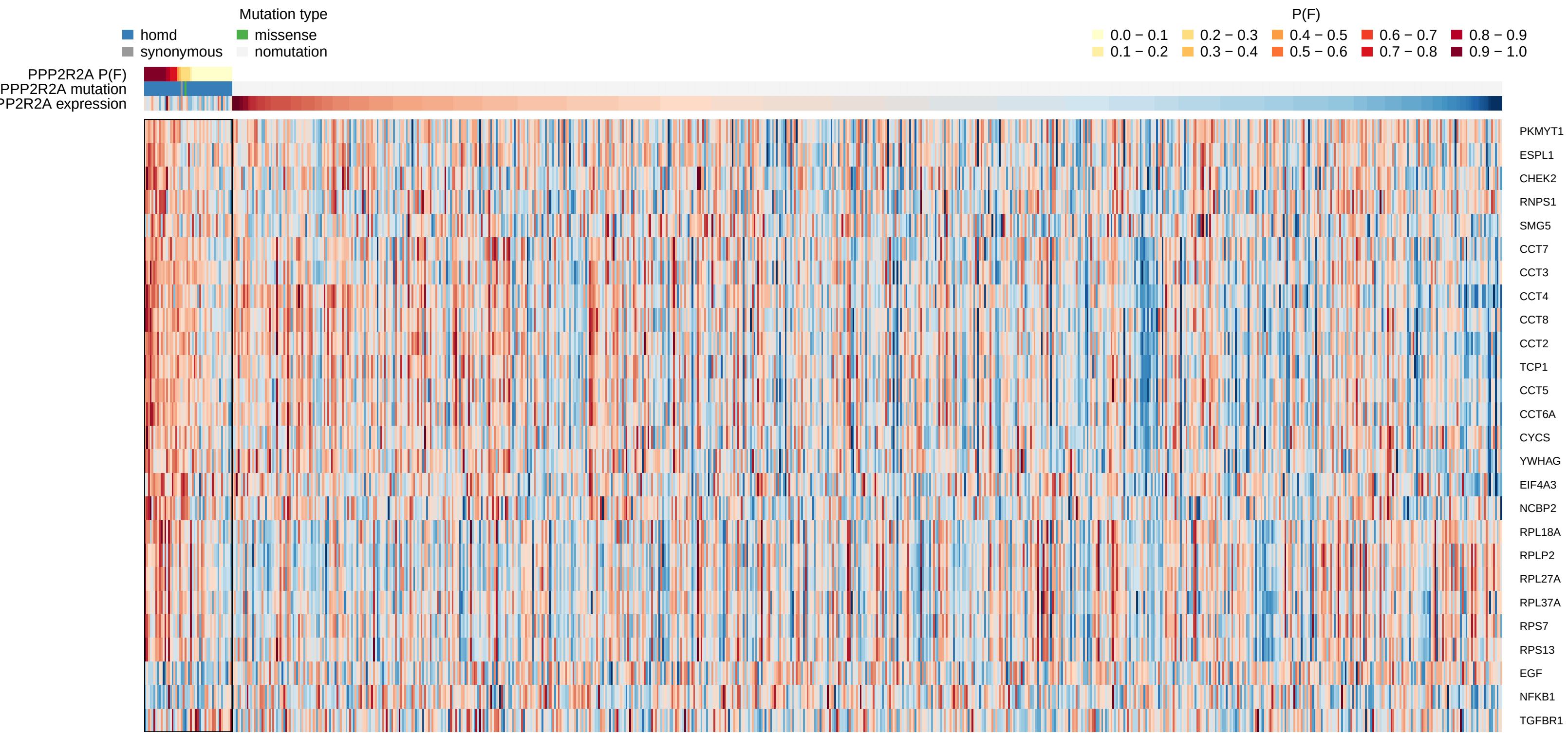

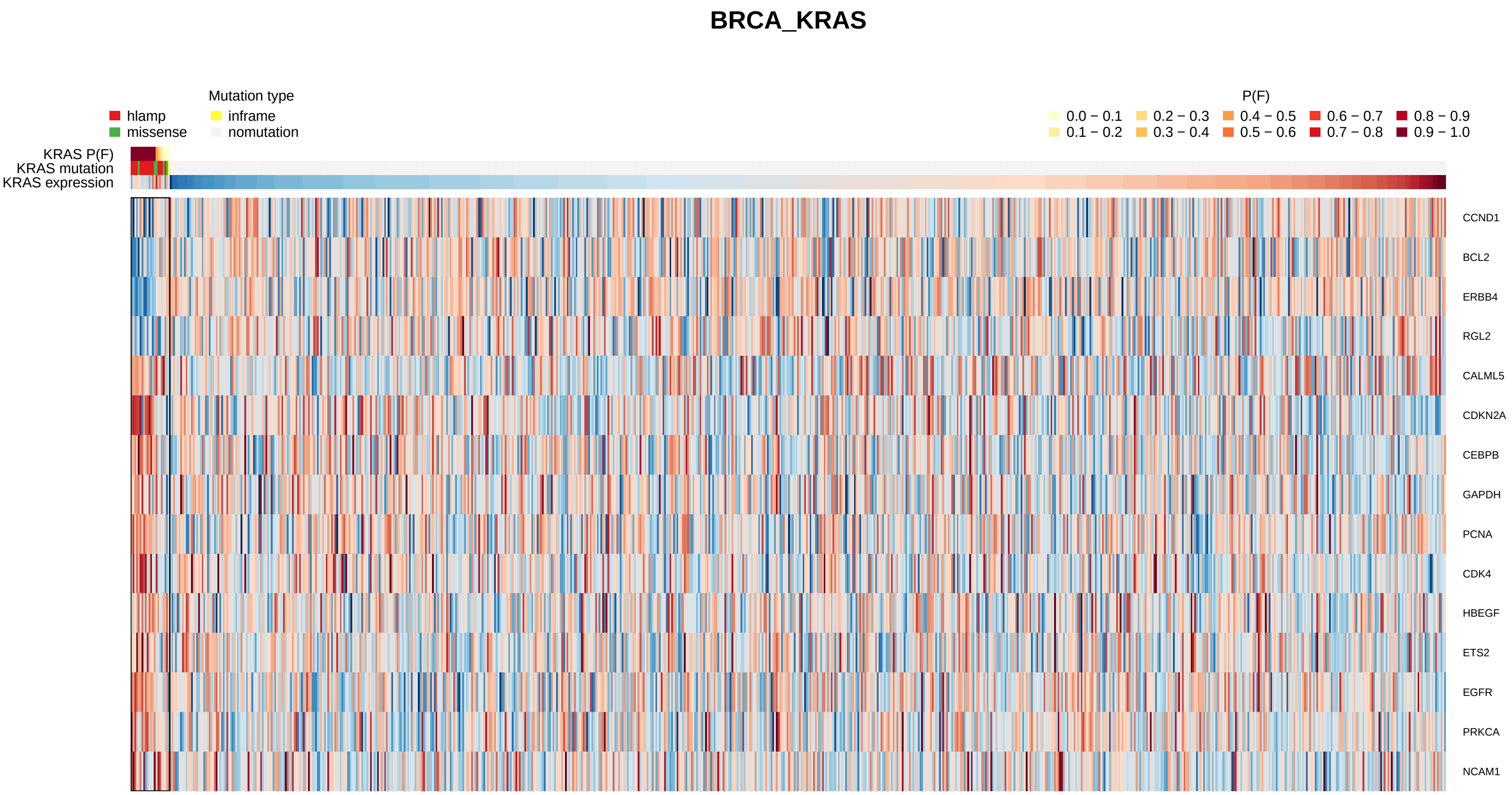
BRCA_KRAS
Mutation type
hlamp
missense
inframe
nomutation
P(F)
0.0 − 0.1
0.1 − 0.2
0.2 − 0.3
0.3 − 0.4
0.4 − 0.5
0.5 − 0.6
0.6 − 0.7
0.7 − 0.8
0.8 − 0.9
0.9 − 1.0
KRAS P(F)
KRAS mutation
KRAS expression
CCND1
BCL2
ERBB4
RGL2
CALML5
CDKN2A
CEBPB
GAPDH
PCNA
CDK4
HBEGF
ETS2
EGFR
PRKCA
NCAM1

# BRCA_ING1

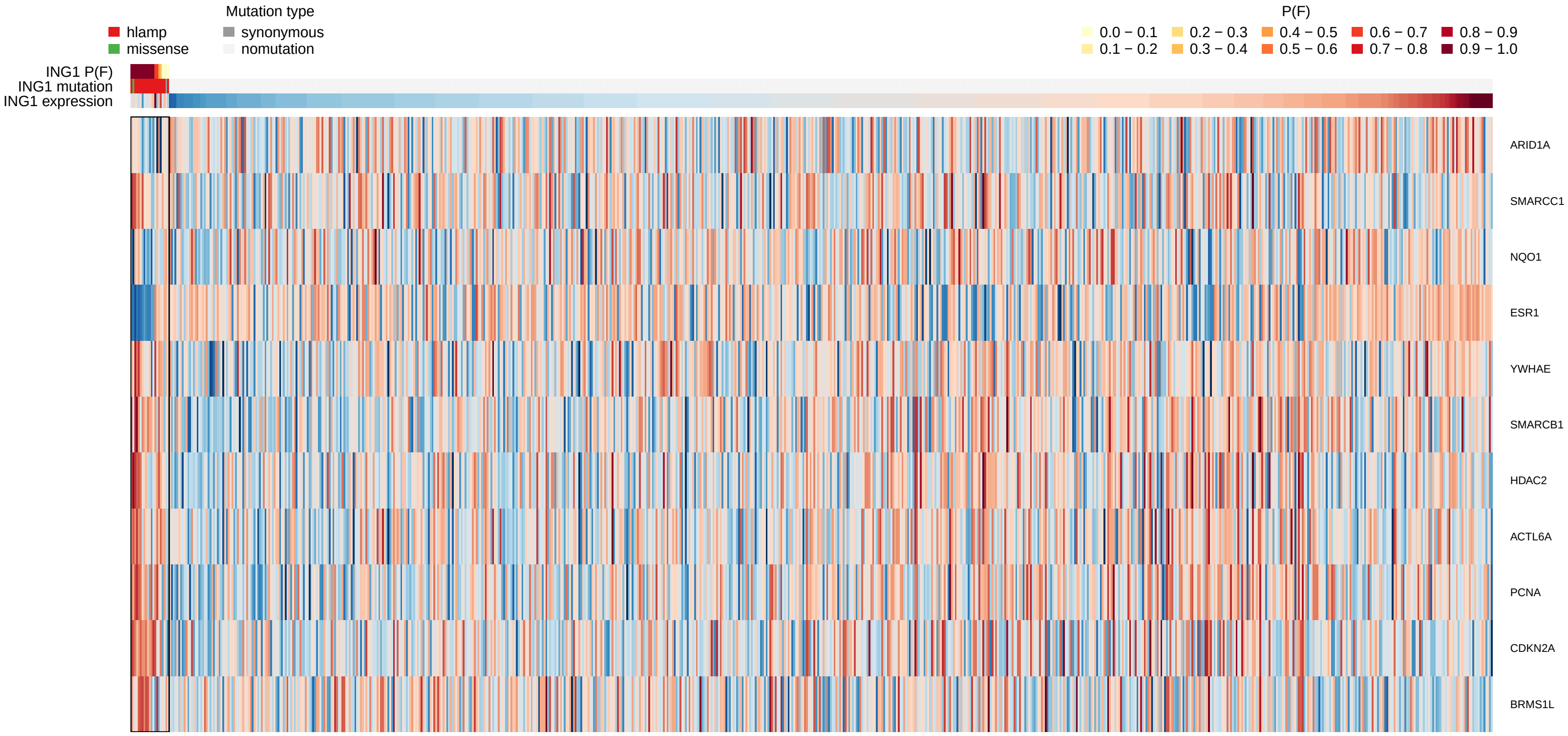
Mutation type
hlamp
missense
synonymous
nomutation
P(F)
0.0 − 0.1
0.1 − 0.2
0.2 − 0.3
0.3 − 0.4
0.4 − 0.5
0.5 − 0.6
0.6 − 0.7
0.7 − 0.8
0.8 − 0.9
0.9 − 1.0
ING1 P(F)
ING1 mutation
ING1 expression
ARID1A
SMARCC1
NQO1
ESR1
YWHAE
SMARCB1
HDAC2
ACTL6A
PCNA
CDKN2A
BRMS1L

# BRCA_WNK1

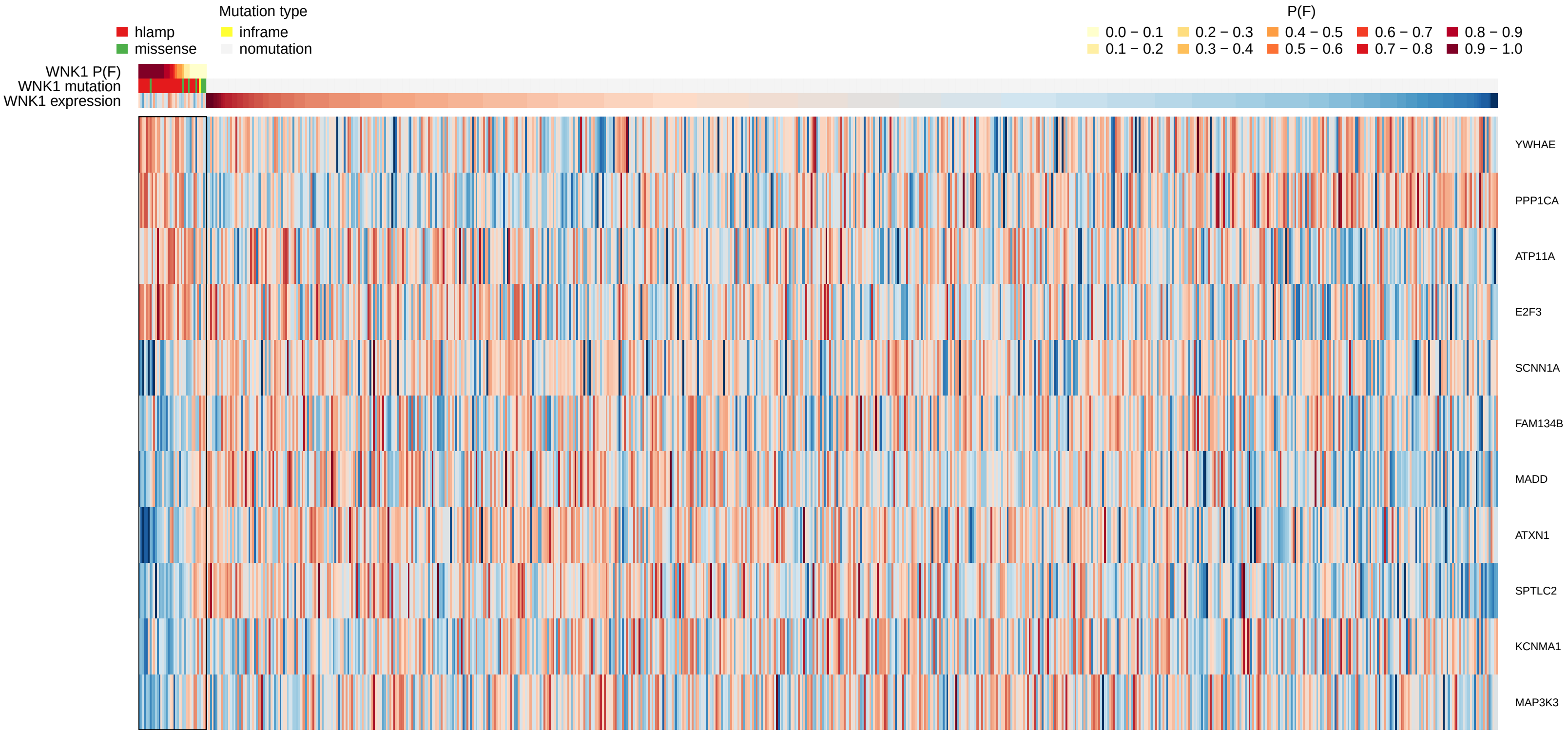
Mutation type
hlamp
missense
inframe
nomutation
P(F)
0.0 − 0.1
0.1 − 0.2
0.2 − 0.3
0.3 − 0.4
0.4 − 0.5
0.5 − 0.6
0.6 − 0.7
0.7 − 0.8
0.8 − 0.9
0.9 − 1.0
WNK1 P(F)
WNK1 mutation
WNK1 expression
YWHAE
PPP1CA
ATP11A
E2F3
SCNN1A
FAM134B
MADD
ATXN1
SPTLC2
KCNMA1
MAP3K3

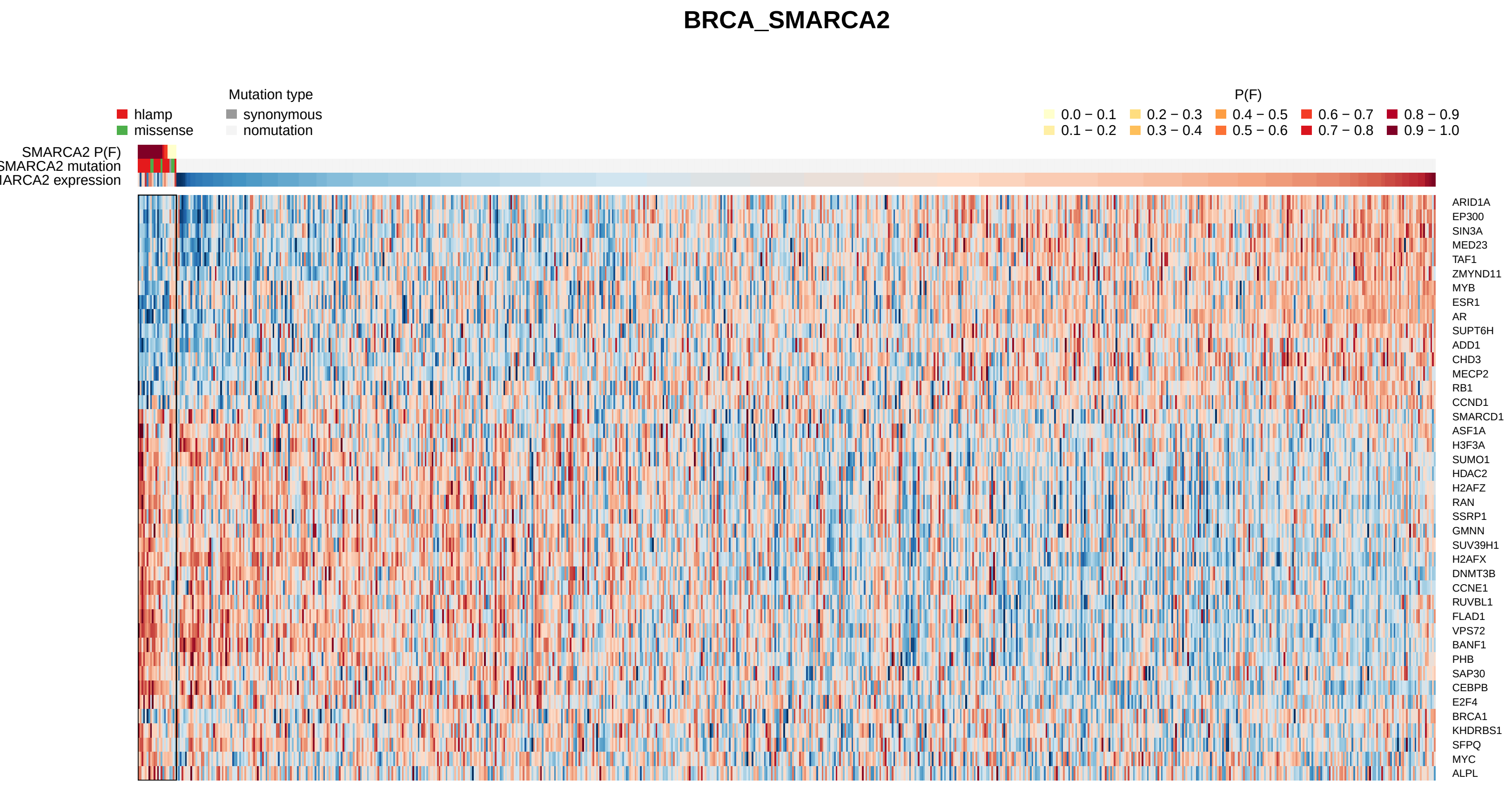
BRCA_SMARCA2
Mutation type
hlamp
missense
synonymous
nomutation
P(F)
0.0 − 0.1
0.1 − 0.2
0.2 − 0.3
0.3 − 0.4
0.4 − 0.5
0.5 − 0.6
0.6 − 0.7
0.7 − 0.8
0.8 − 0.9
0.9 − 1.0
SMARCA2 P(F)
SMARCA2 mutation
SMARCA2 expression
ARID1A
EP300
SIN3A
MED23
TAF1
ZMYND11
MYB
ESR1
AR
SUPT6H
ADD1
CHD3
MECP2
RB1
CCND1
SMARCD1
ASF1A
H3F3A
SUMO1
HDAC2
H2AFZ
RAN
SSRP1
GMNN
SUV39H1
H2AFX
DNMT3B
CCNE1
RUVBL1
FLAD1
VPS72
BANF1
PHB
SAP30
CEBPB
E2F4
BRCA1
KHDRBS1
SFPQ
MYC
ALPL

Supplementary Fig. 10: Trans-effects of copy number alterations of genes in the METABRIC breast cancer dataset. Heatmaps show the expression of the METABRIC breast cancer genes connected to the 14 high probability genes predicted in both TCGA and METABRIC datasets as well as the posterior marginal probabilities $P(F)$ in the METABRIC breast cancer dataset.

# METABRIC_MYC

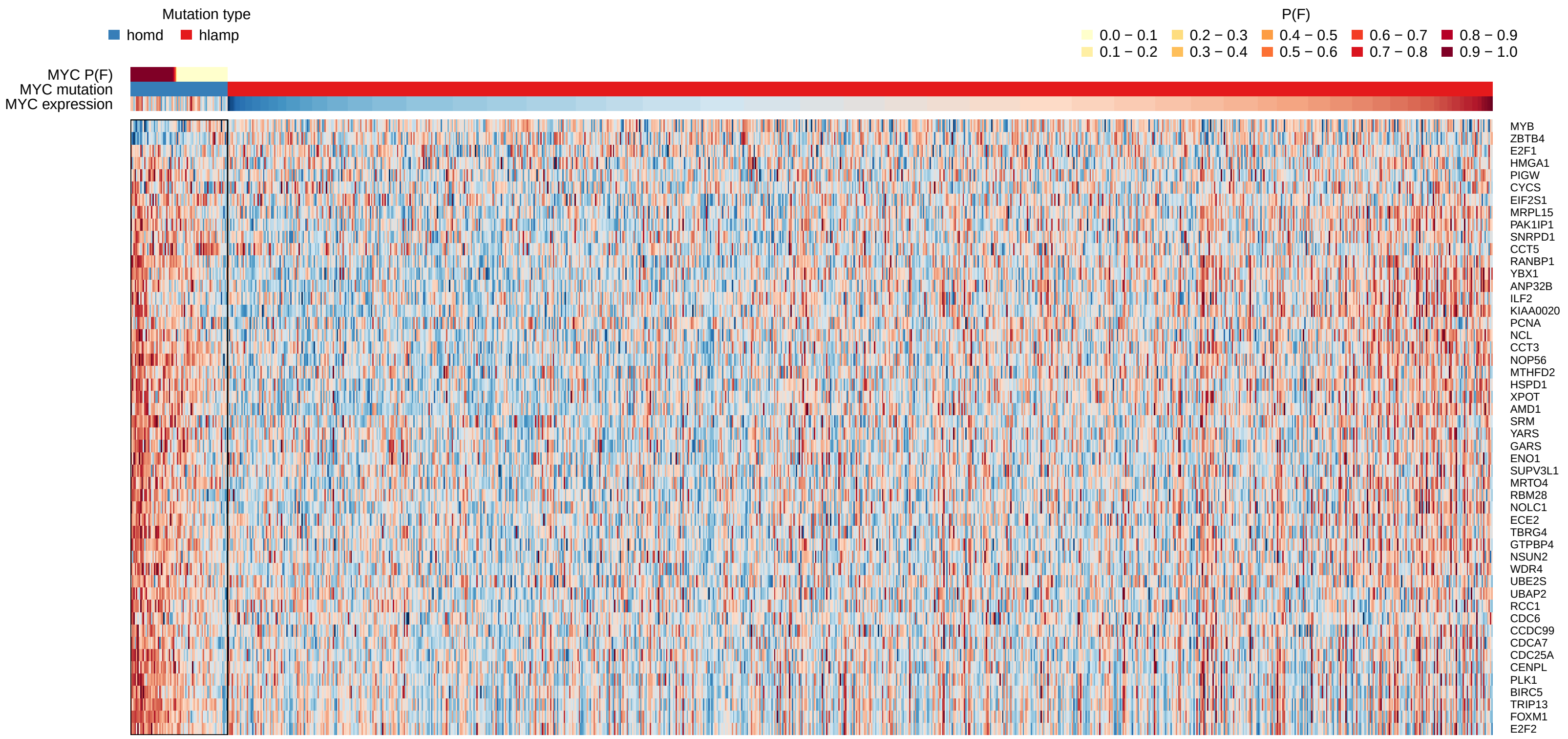

## METABRIC_ERBB2

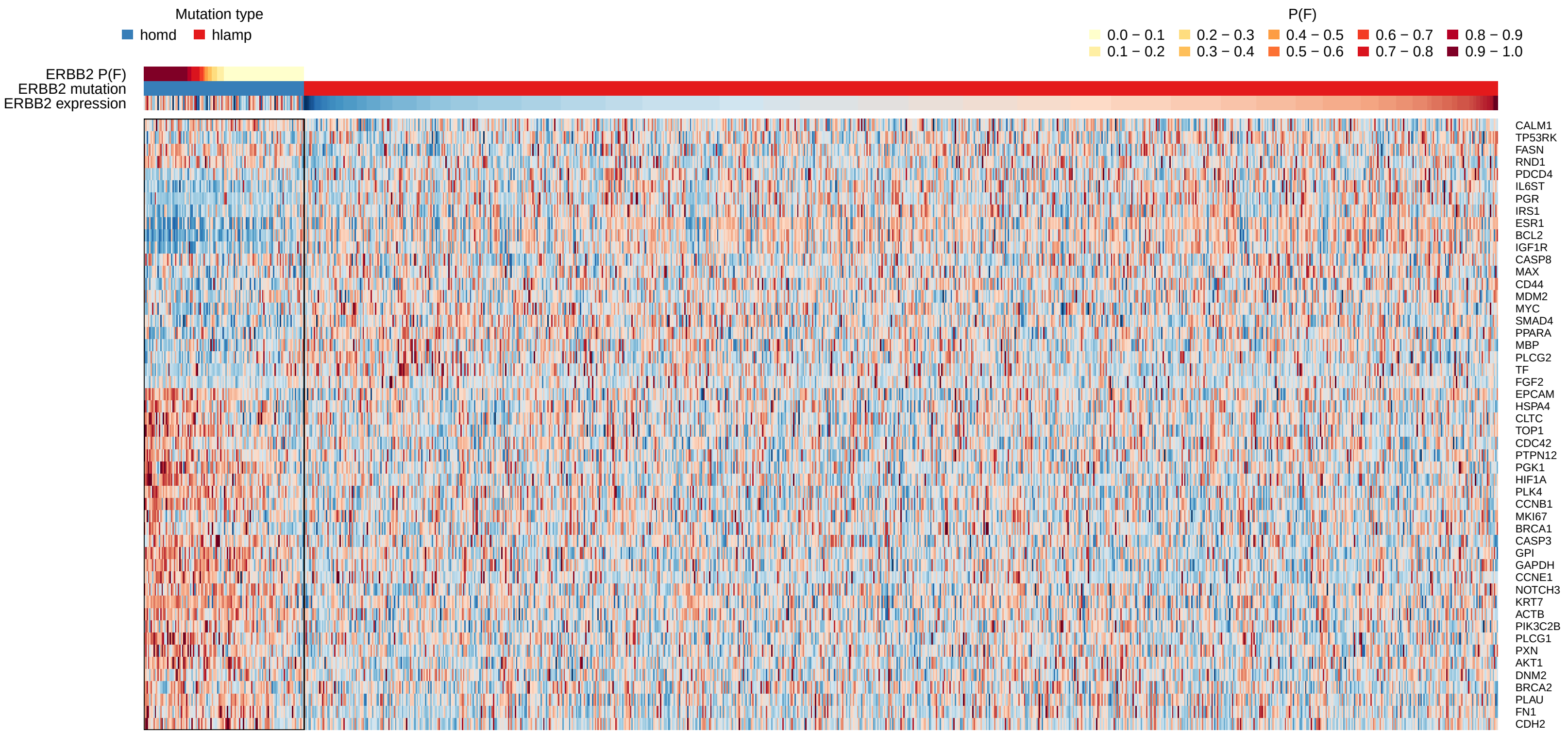

# METABRIC_CCNE1

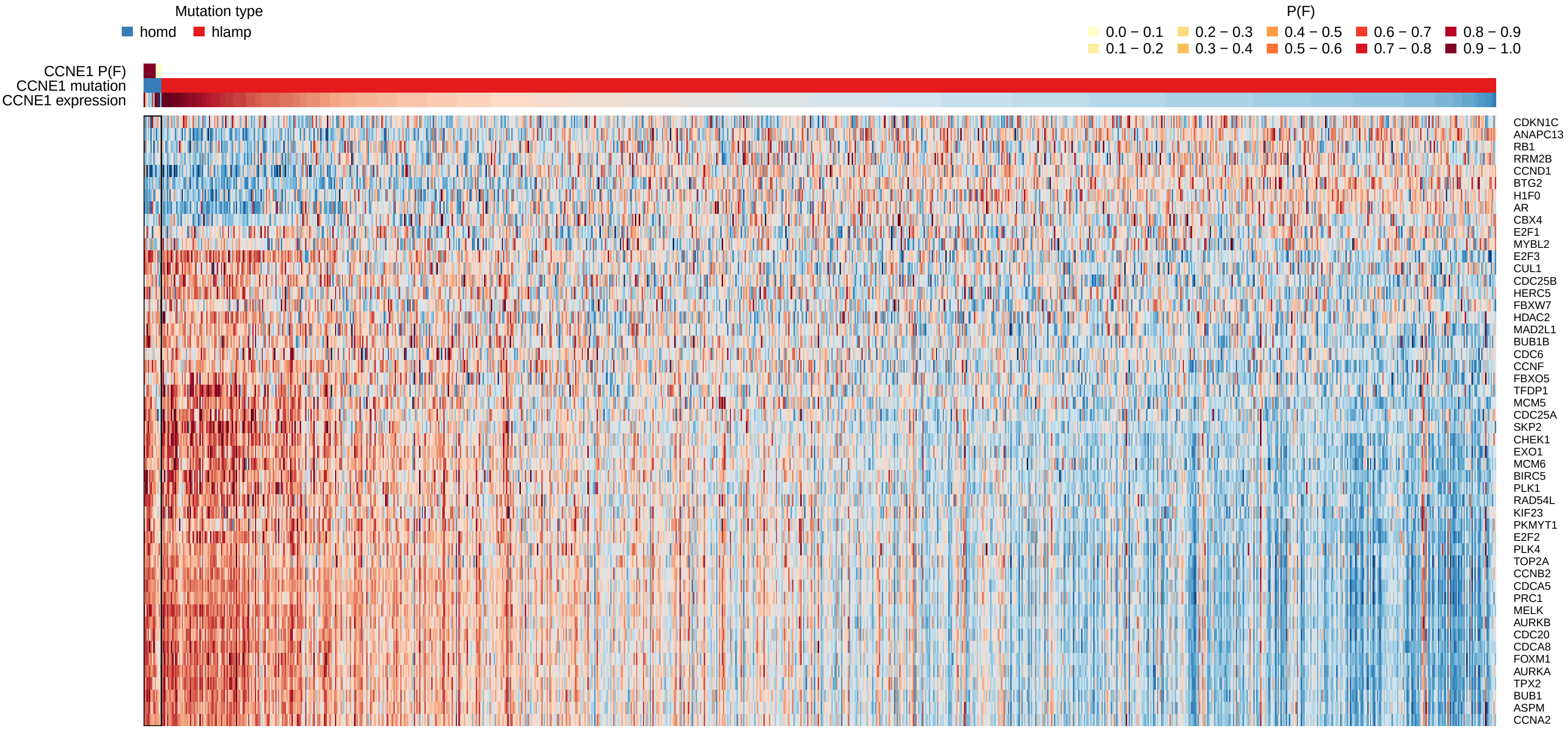

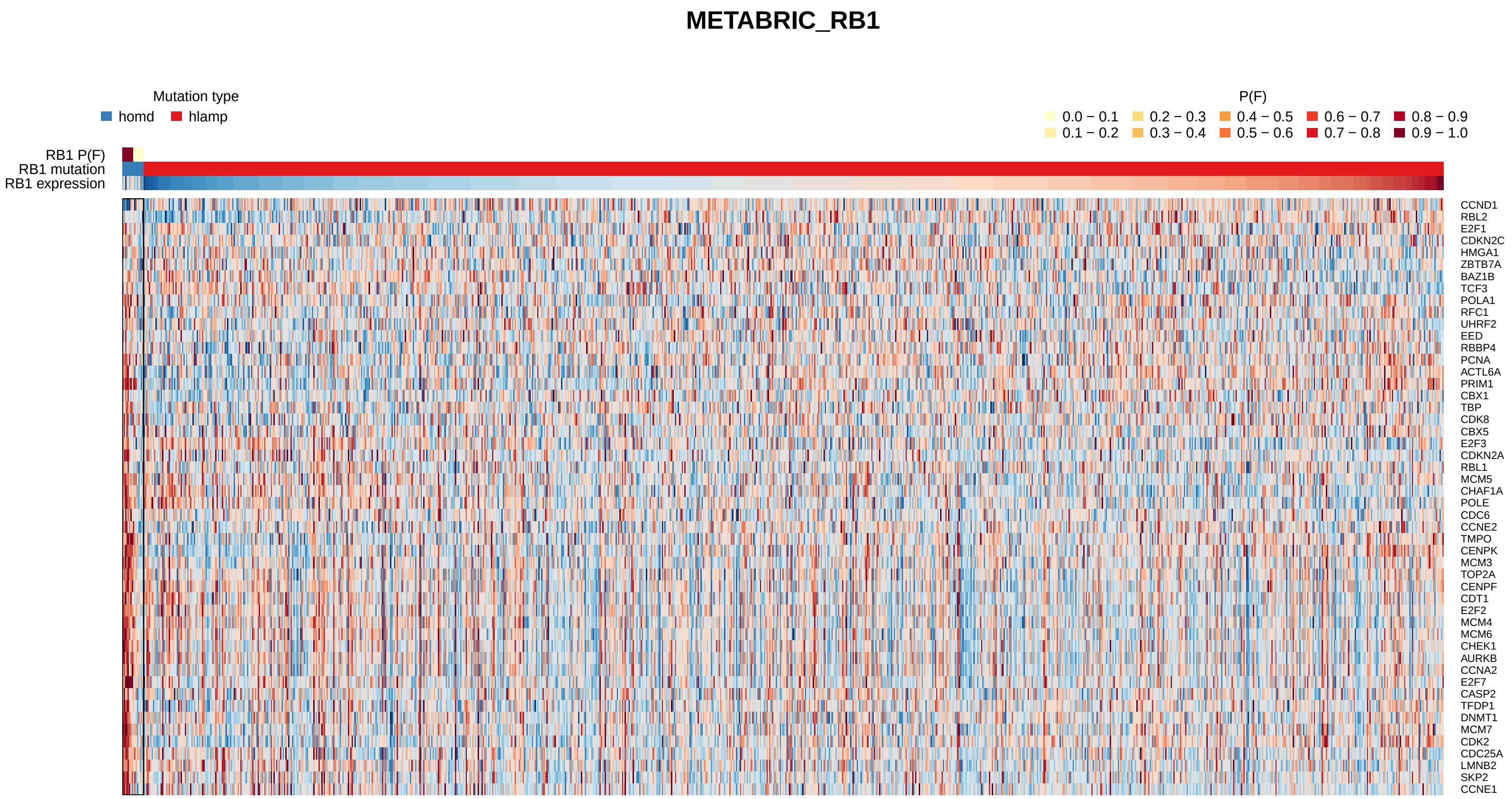
METABRIC_RB1
Mutation type
homd
hlamp
P(F)
0.0 − 0.1
0.1 − 0.2
0.2 − 0.3
0.3 − 0.4
0.4 − 0.5
0.5 − 0.6
0.6 − 0.7
0.7 − 0.8
0.8 − 0.9
0.9 − 1.0
RB1 P(F)
RB1 mutation
RB1 expression
CCND1
RBL2
E2F1
CDKN2C
HMGA1
ZBTB7A
BAZ1B
TCF3
POLA1
RFC1
UHRF2
EED
RBBP4
PCNA
ACTL6A
PRIM1
CBX1
TBP
CDK8
CBX5
E2F3
CDKN2A
RBL1
MCM5
CHAF1A
POLE
CDC6
CCNE2
TMPO
CENPK
MCM3
TOP2A
CENPF
CDT1
E2F2
MCM4
MCM6
CHEK1
AURKB
CCNA2
E2F7
CASP2
TFDP1
DNMT1
MCM7
CDK2
CDC25A
LMNB2
SKP2
CCNE1

# METABRIC_CCND1

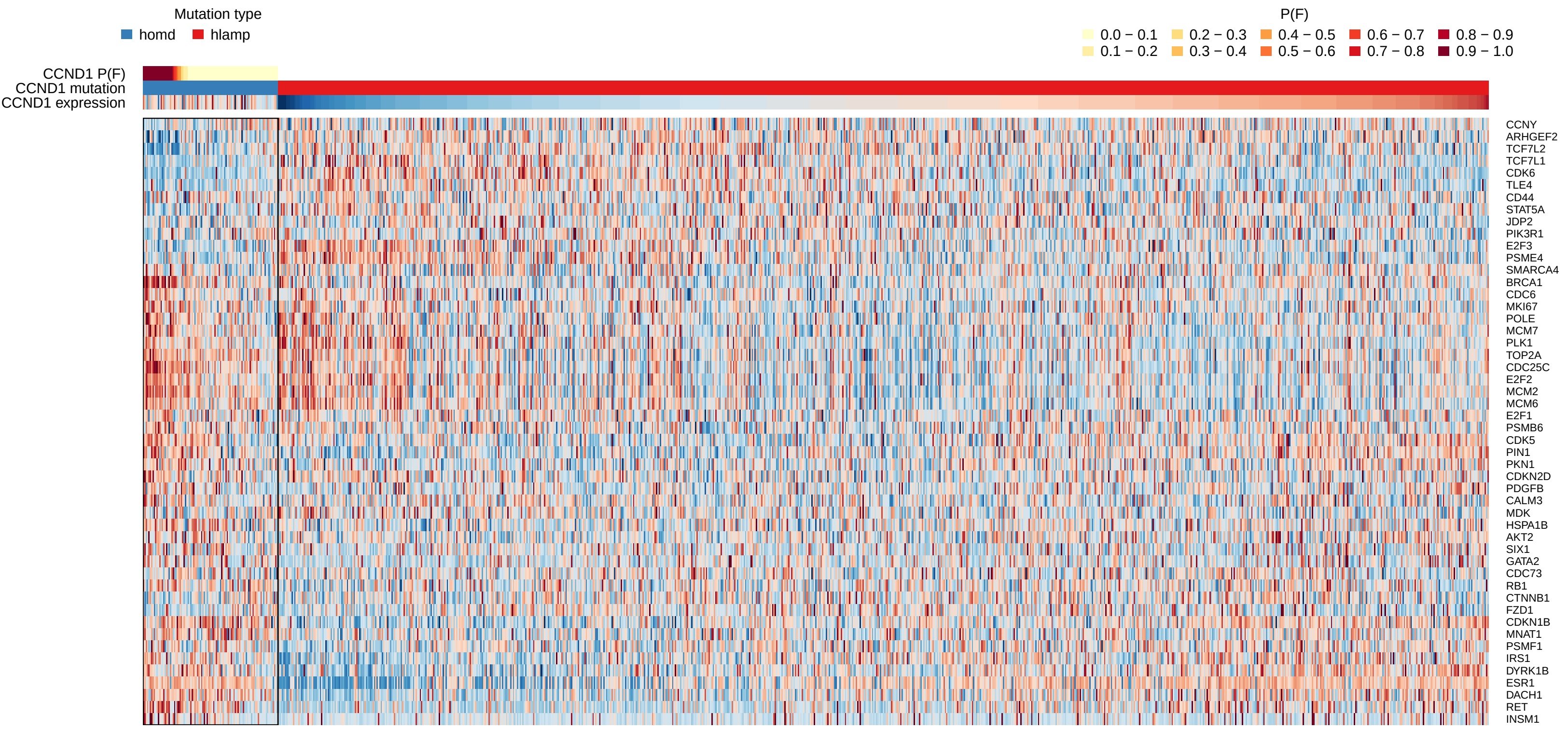

## METABRIC_CBX8

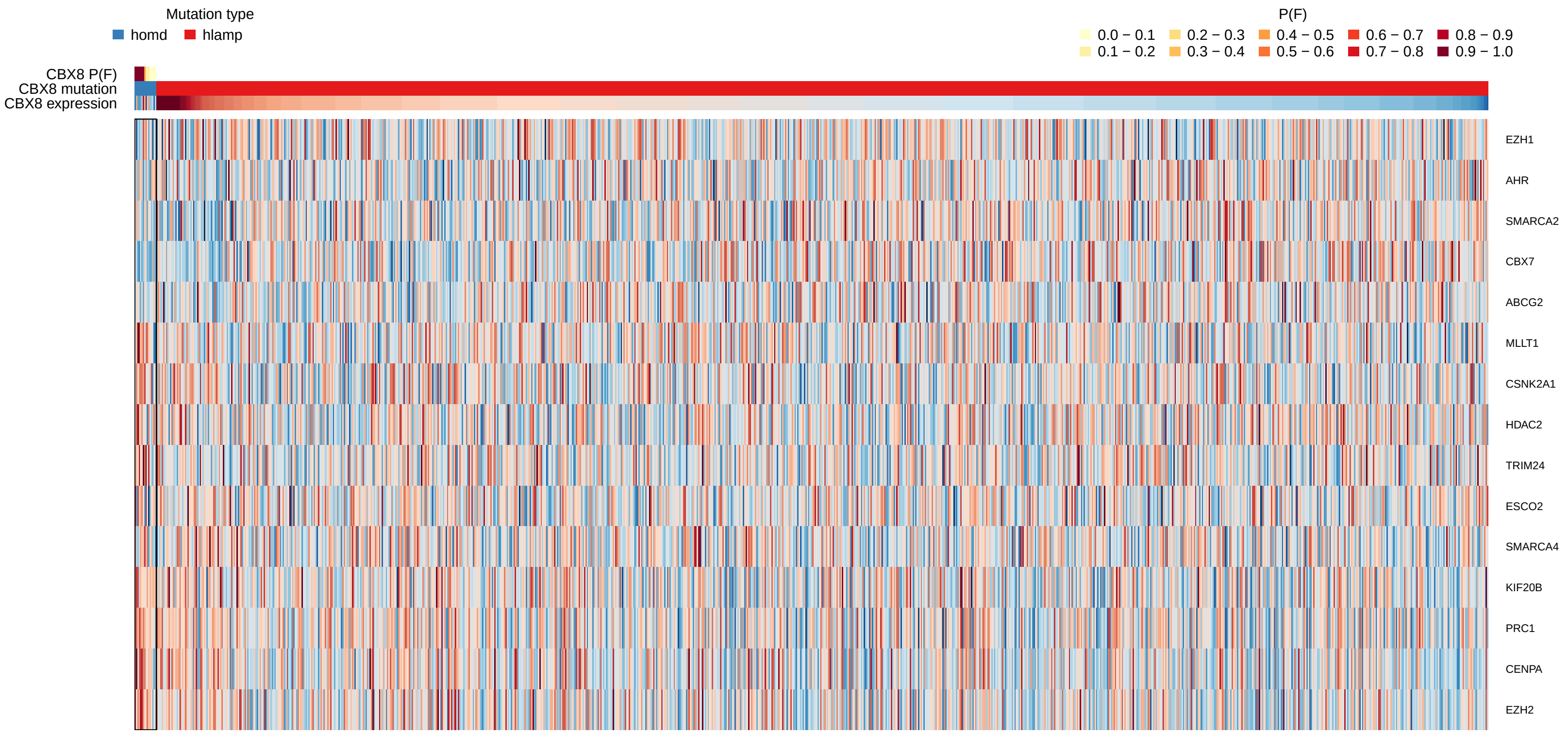

## METABRIC_CDKN2A

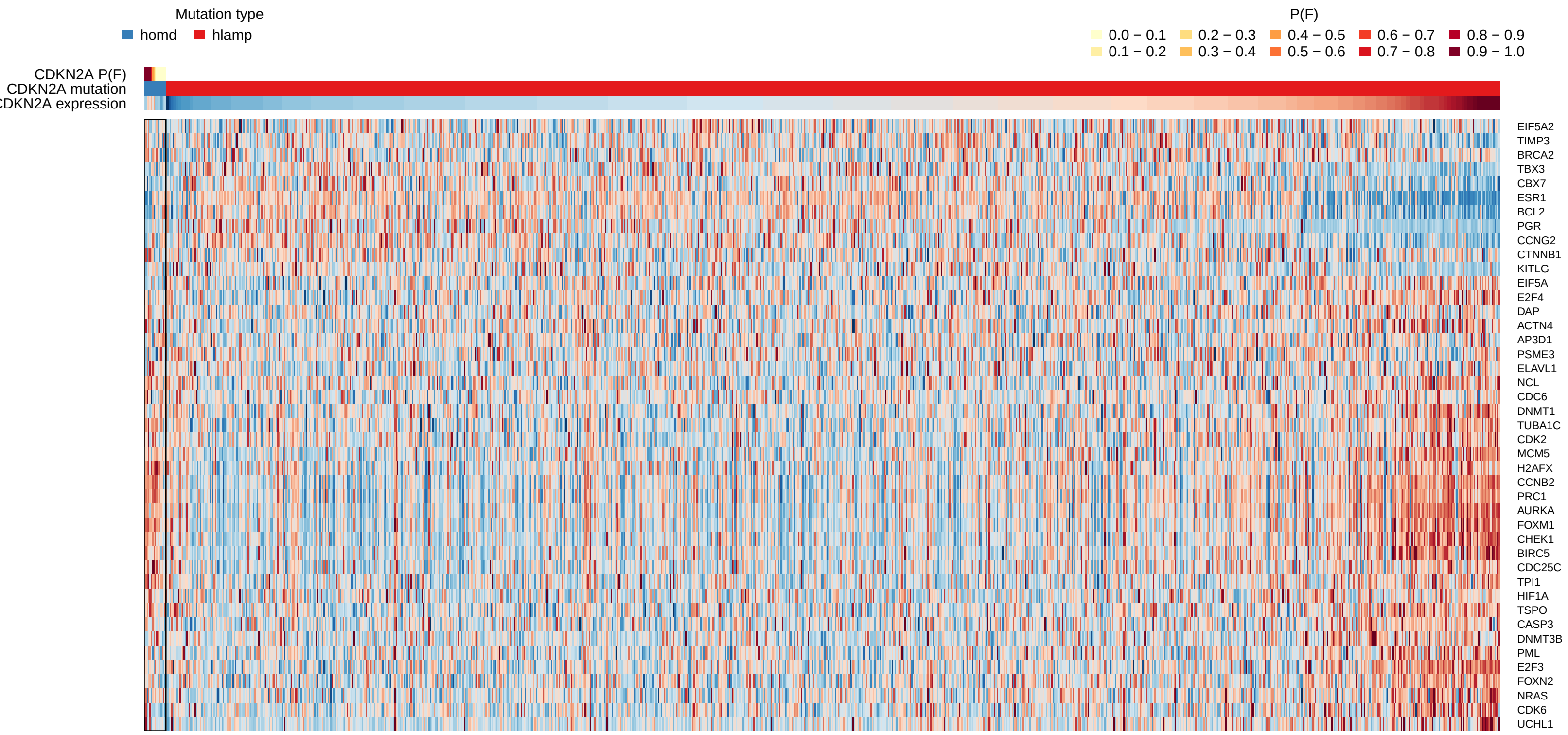

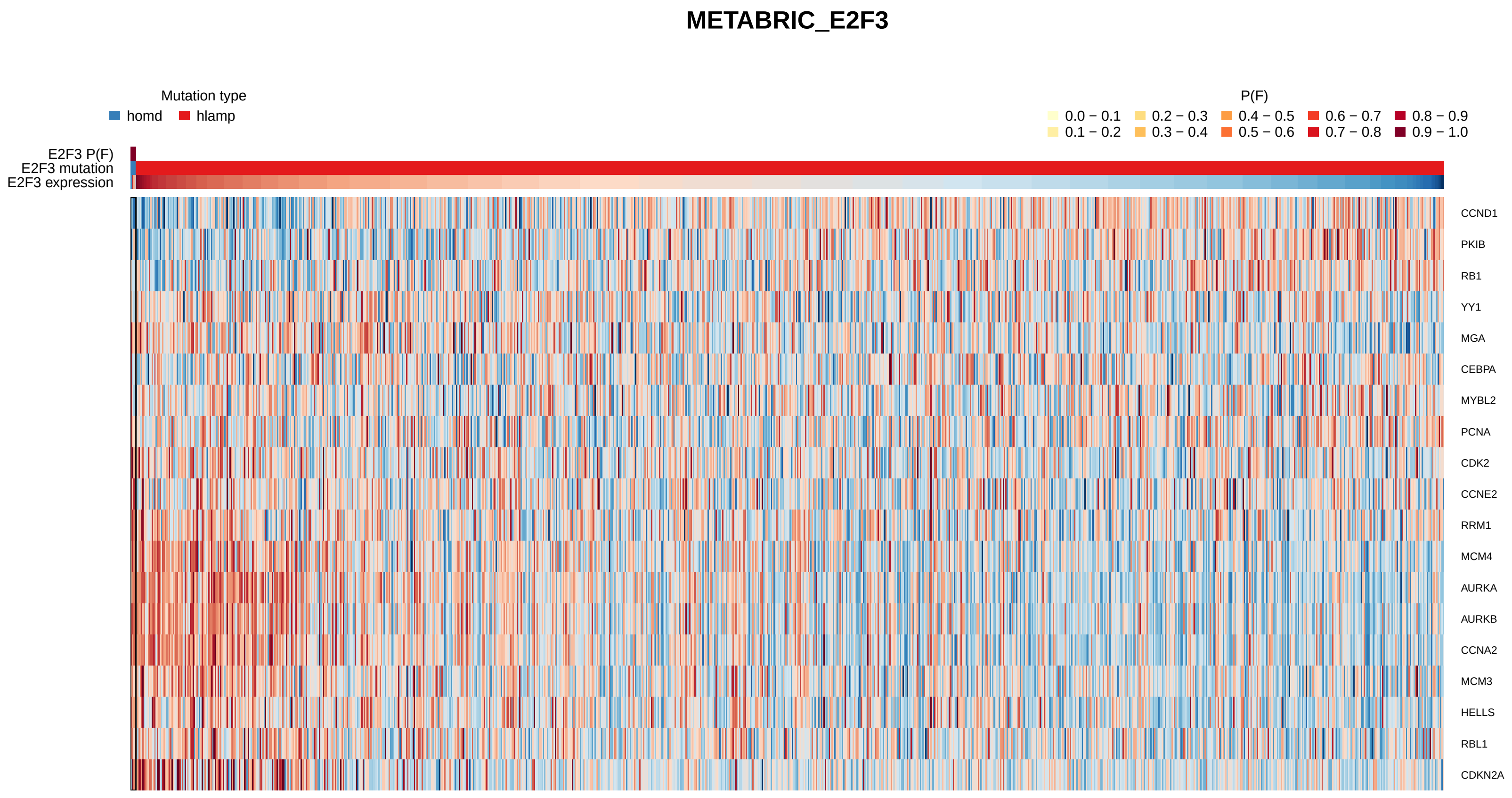
METABRIC_E2F3
Mutation type
homd
hlamp
P(F)
0.0 − 0.1
0.1 − 0.2
0.2 − 0.3
0.3 − 0.4
0.4 − 0.5
0.5 − 0.6
0.6 − 0.7
0.7 − 0.8
0.8 − 0.9
0.9 − 1.0
E2F3 P(F)
E2F3 mutation
E2F3 expression
CCND1
PKIB
RB1
YY1
MGA
CEBPA
MYBL2
PCNA
CDK2
CCNE2
RRM1
MCM4
AURKA
AURKB
CCNA2
MCM3
HELLS
RBL1
CDKN2A

# METABRIC_PTEN

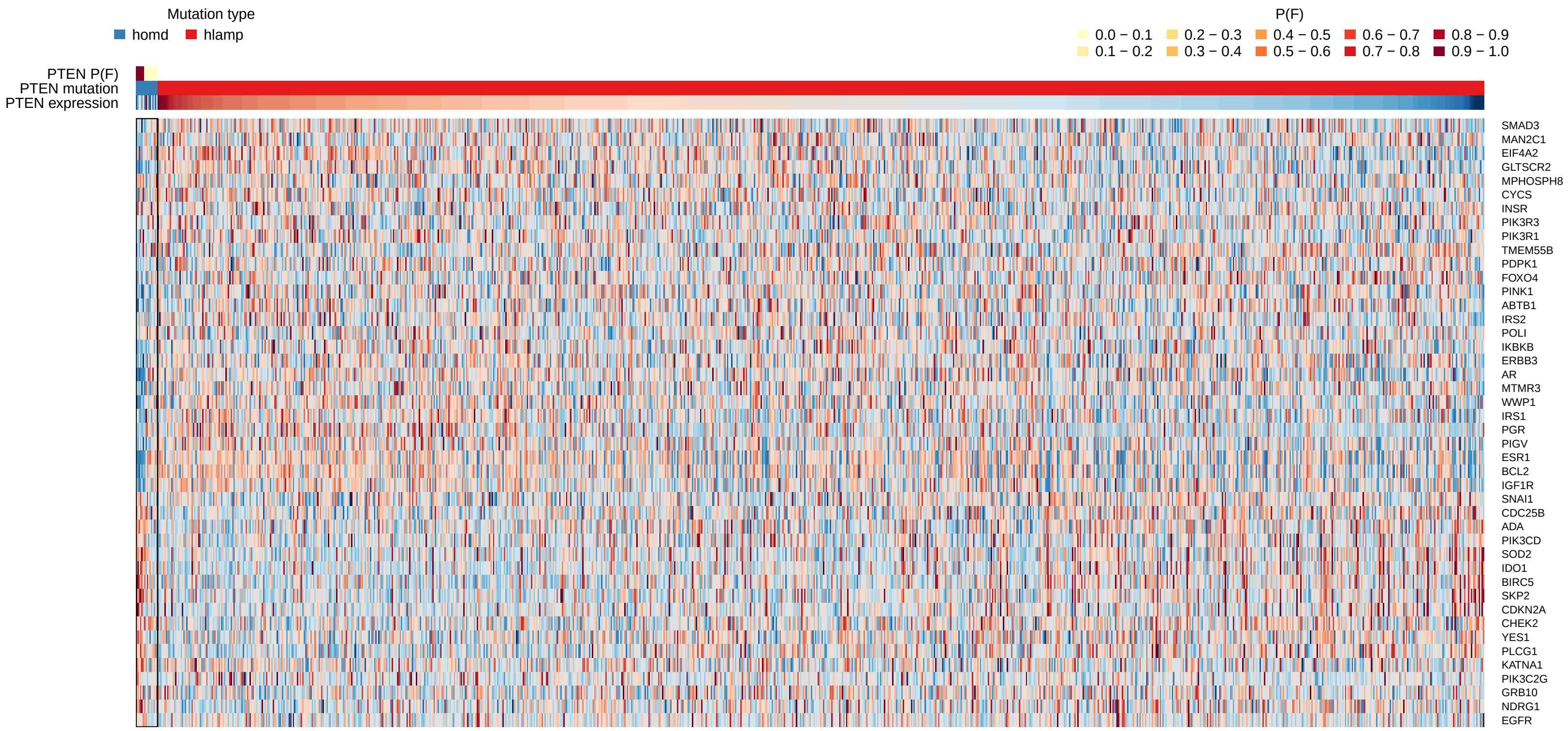

# METABRIC_PPP2R2A

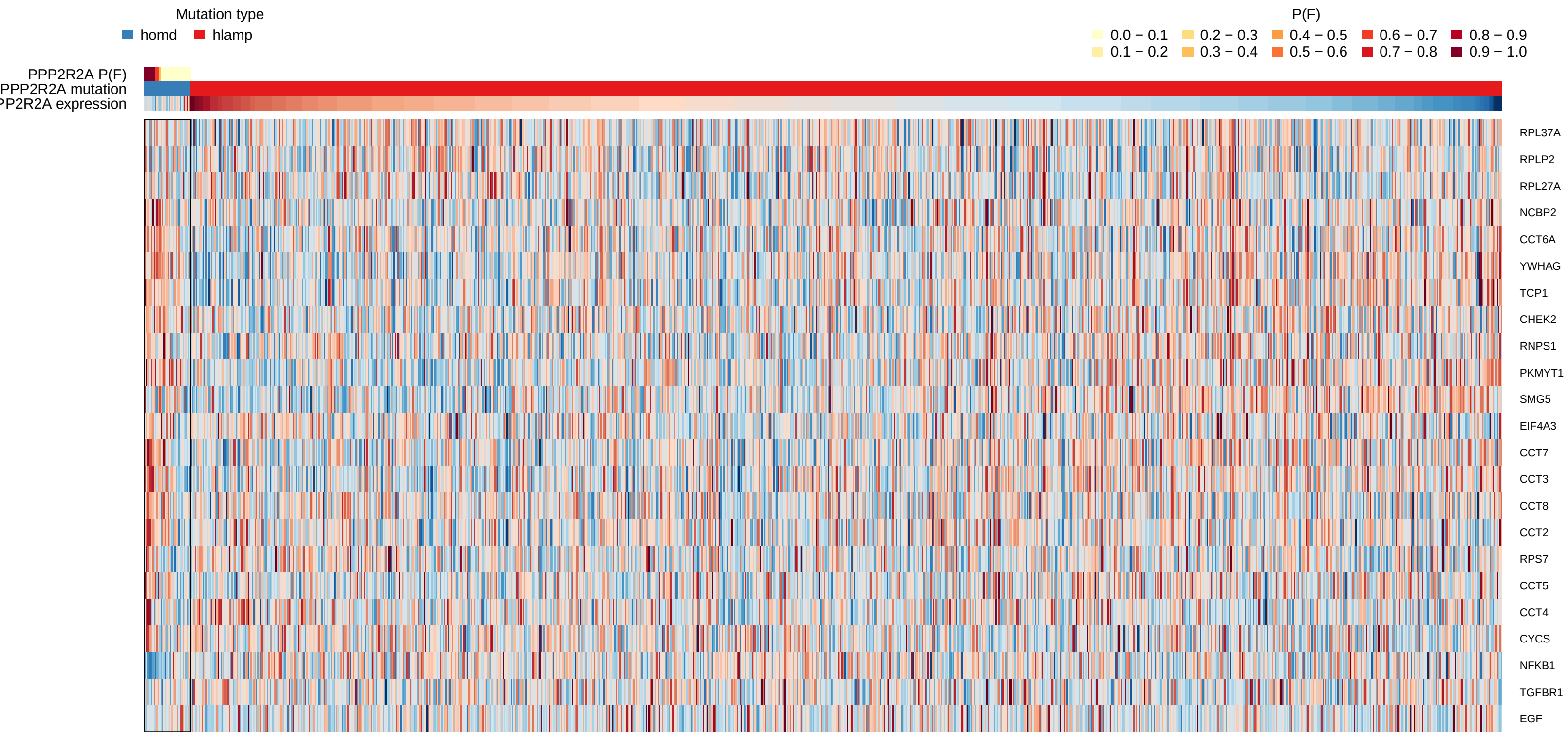

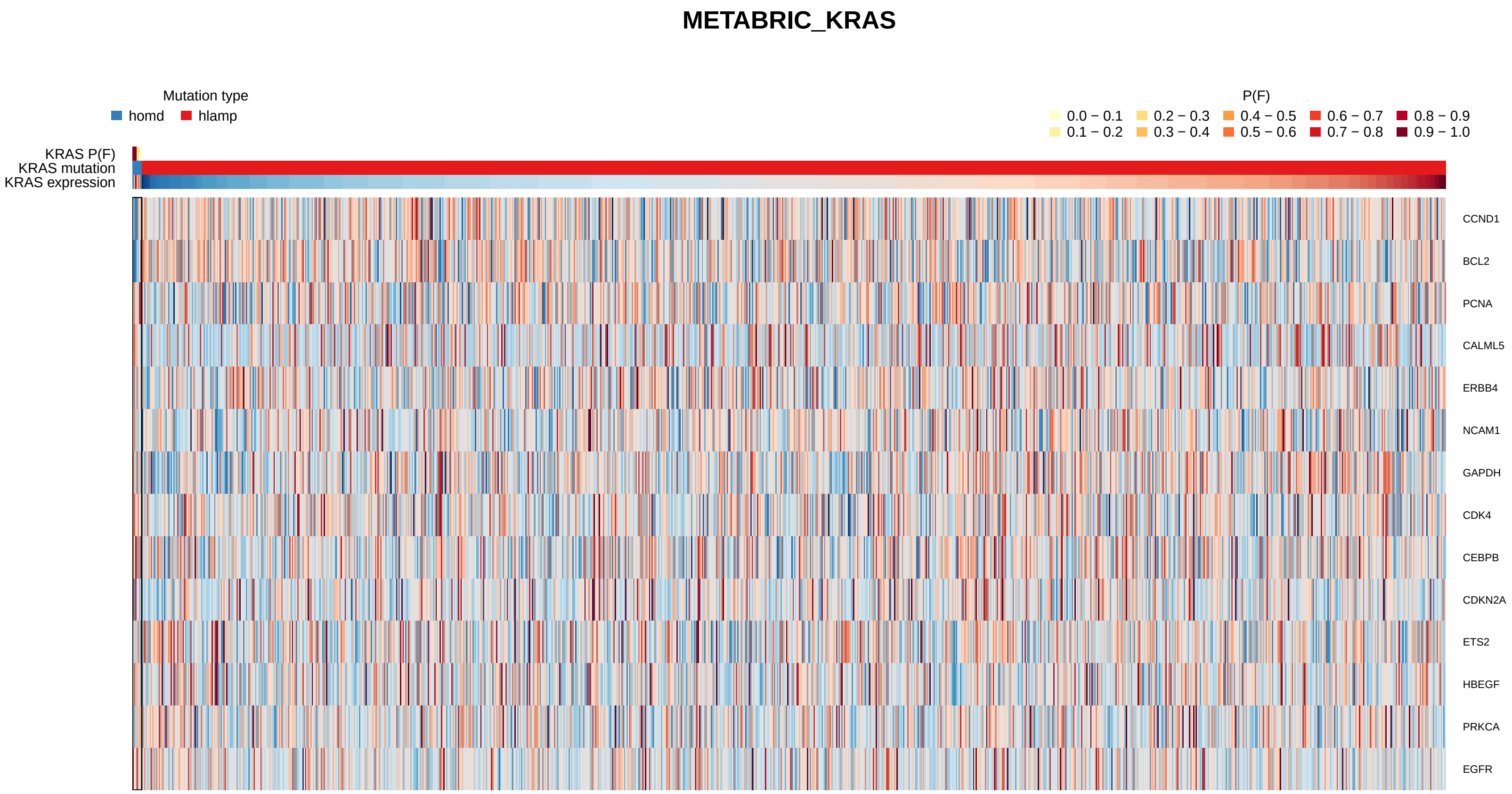
METABRIC_KRAS
Mutation type
homd
hlamp
P(F)
0.0 − 0.1
0.1 − 0.2
0.2 − 0.3
0.3 − 0.4
0.4 − 0.5
0.5 − 0.6
0.6 − 0.7
0.7 − 0.8
0.8 − 0.9
0.9 − 1.0
KRAS P(F)
KRAS mutation
KRAS expression
CCND1
BCL2
PCNA
CALML5
ERBB4
NCAM1
GAPDH
CDK4
CEBPB
CDKN2A
ETS2
HBEGF
PRKCA
EGFR

# METABRIC_ING1

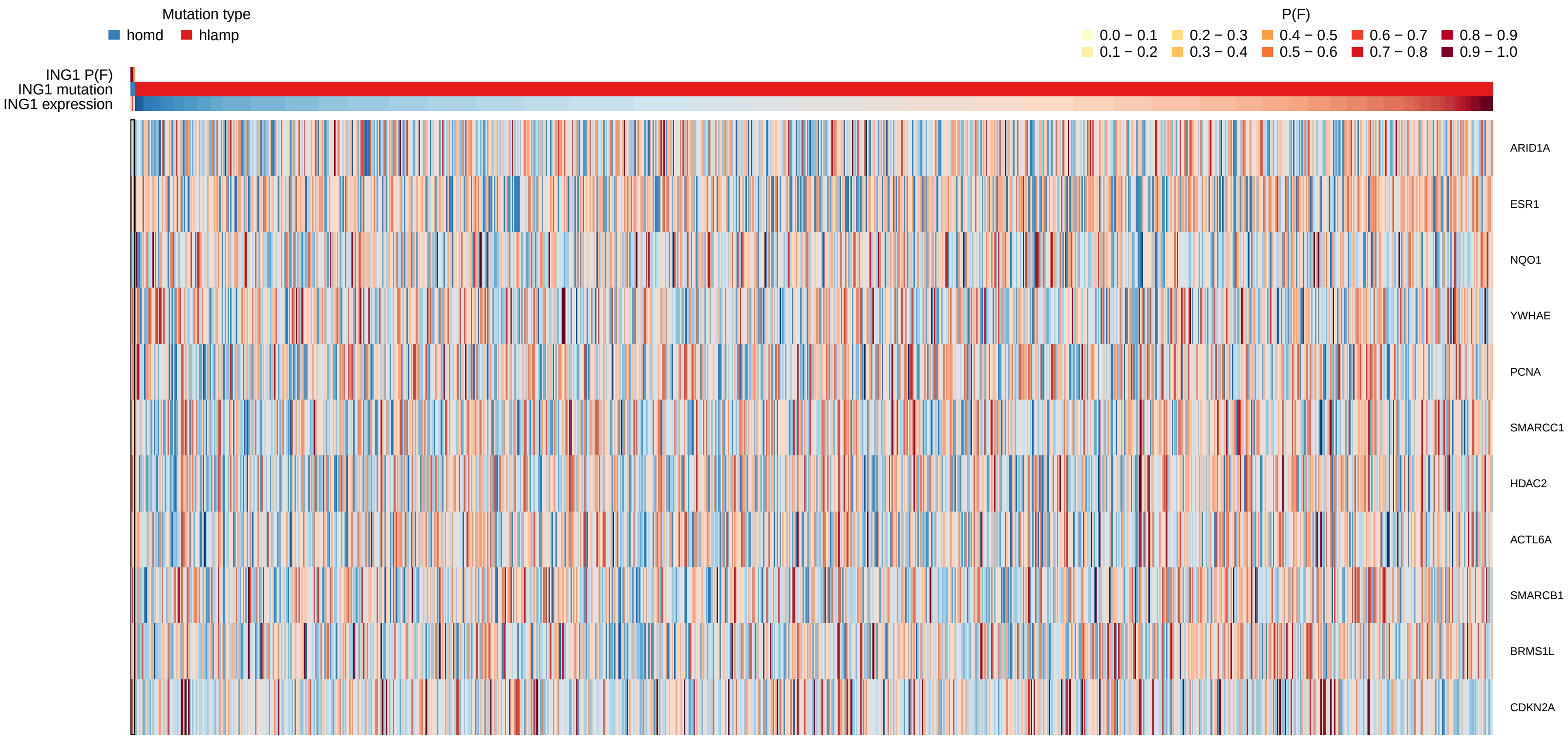

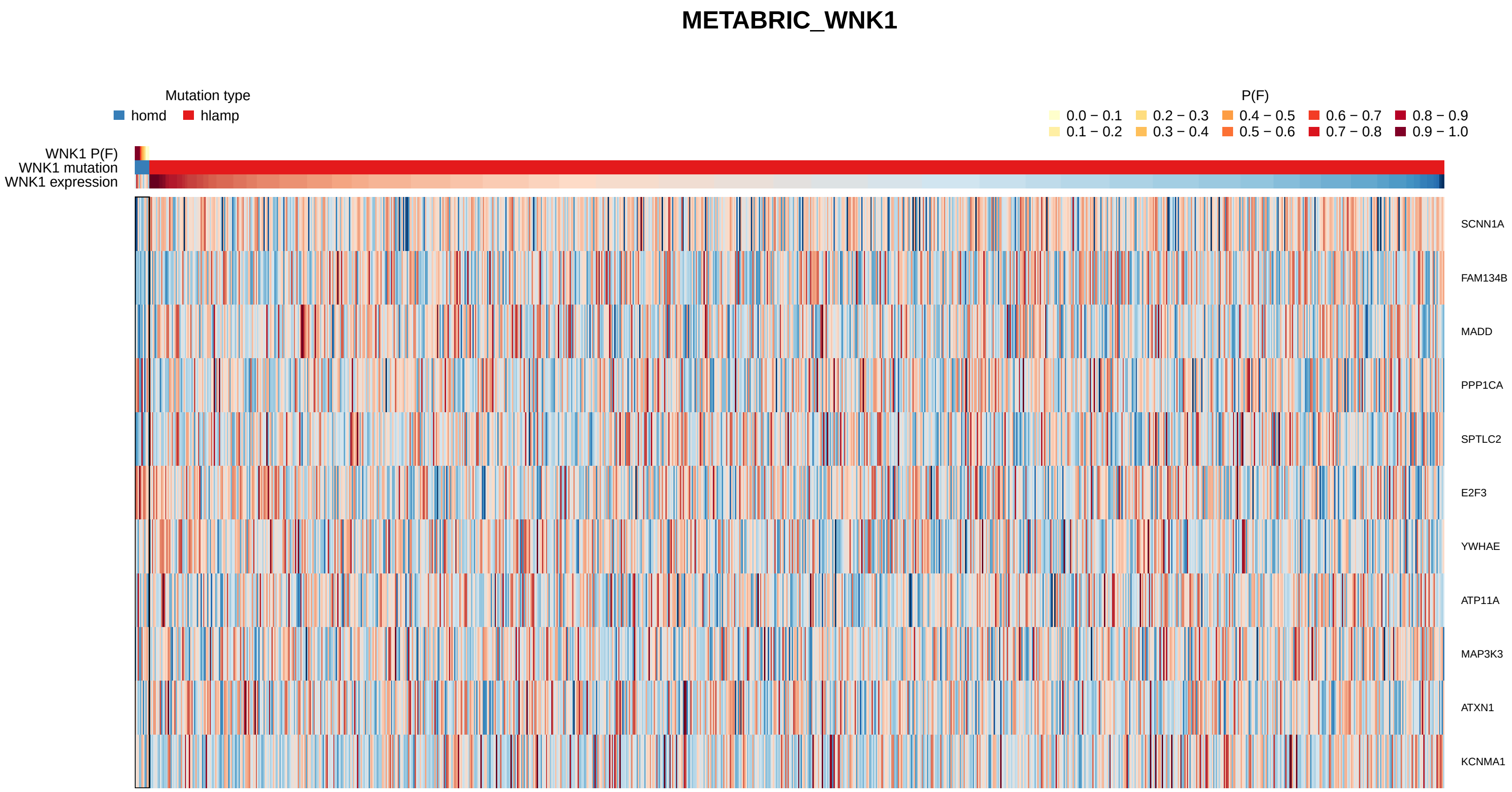
METABRIC_WNK1
Mutation type
homd
hlamp
P(F)
0.0 − 0.1
0.1 − 0.2
0.2 − 0.3
0.3 − 0.4
0.4 − 0.5
0.5 − 0.6
0.6 − 0.7
0.7 − 0.8
0.8 − 0.9
0.9 − 1.0
WNK1 P(F)
WNK1 mutation
WNK1 expression
SCNN1A
FAM134B
MADD
PPP1CA
SPTLC2
E2F3
YWHAE
ATP11A
MAP3K3
ATXN1
KCNMA1

# METABRIC_SMARCA2

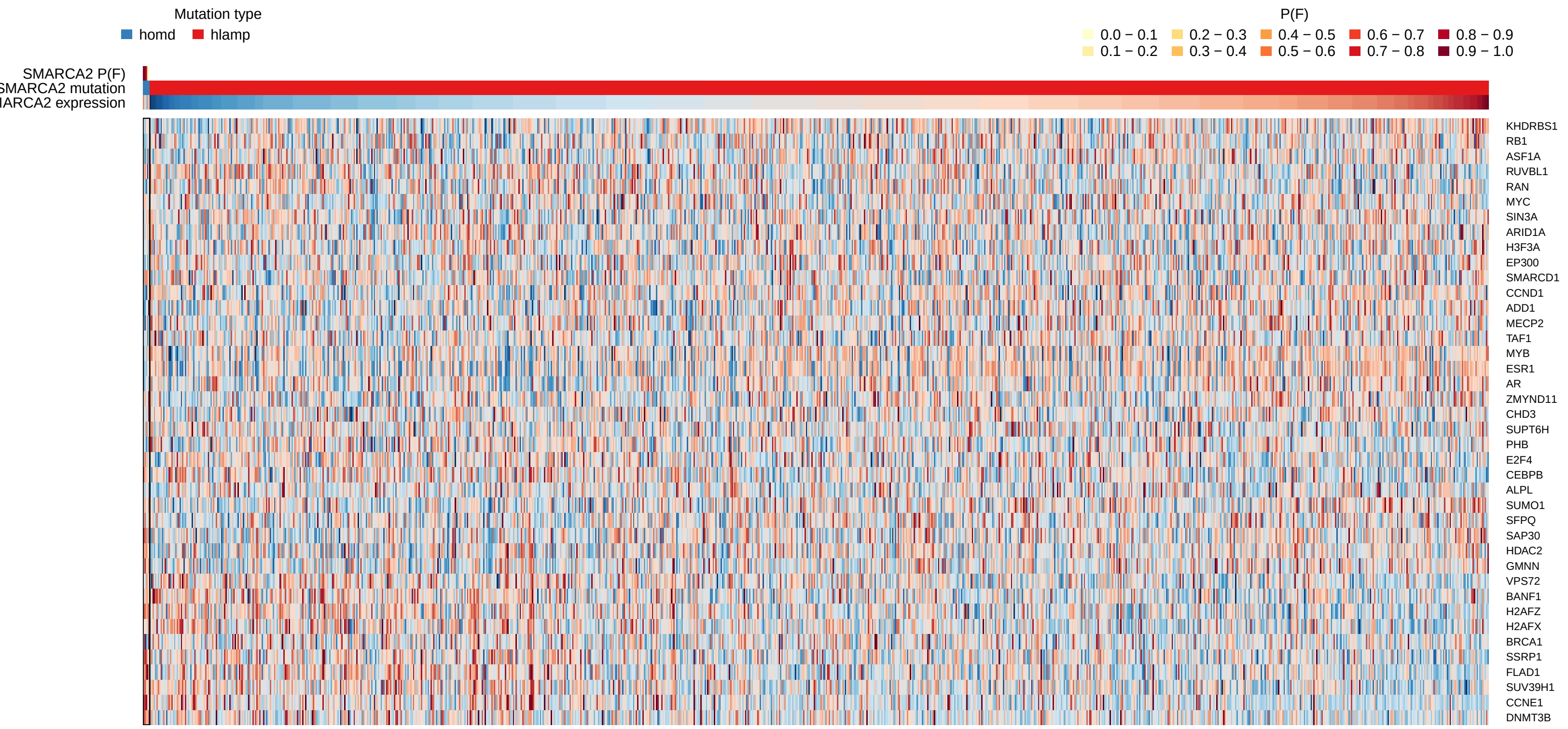

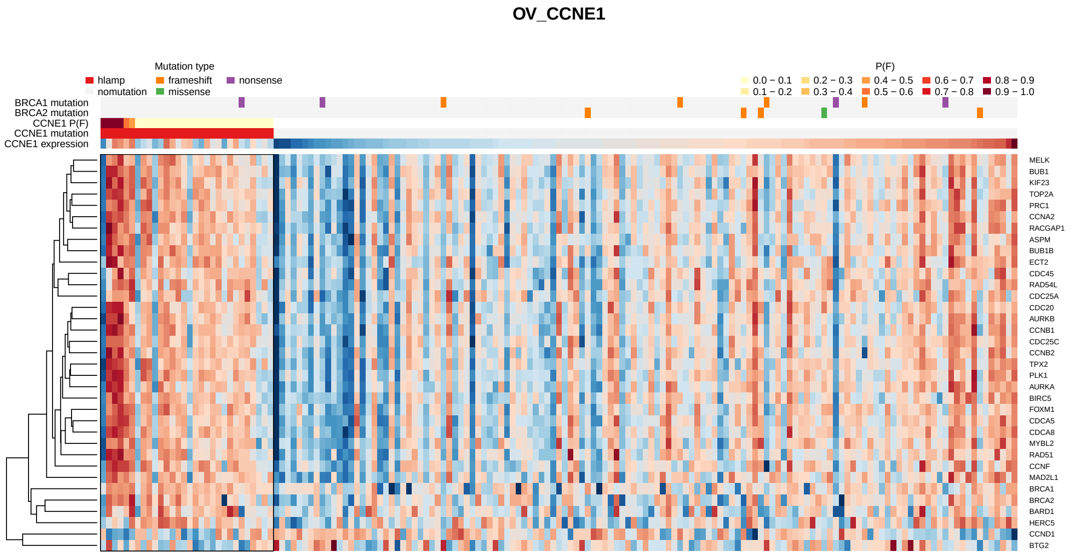


Supplementary Fig. 11: *CCNE1* amplifications in high grade serous ovarian cancer prediction by `xseq-simple`. *CCNE1* amplification probabilities $P(F)$ predicted by the simplified version of `xseq` without considering the directionality of gene regulation.

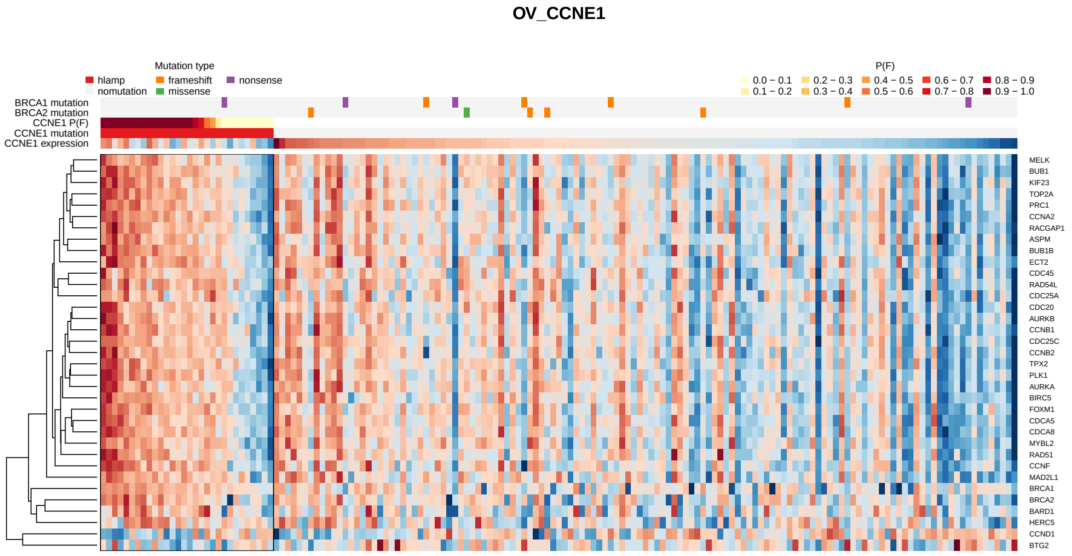


Supplementary Fig. 12: *CCNE1* amplifications in high grade serous ovarian cancer prediction by `xseq`. *CCNE1* amplification probabilities $P(F)$ predicted by `xseq` when the directionality of gene regulation was considered.

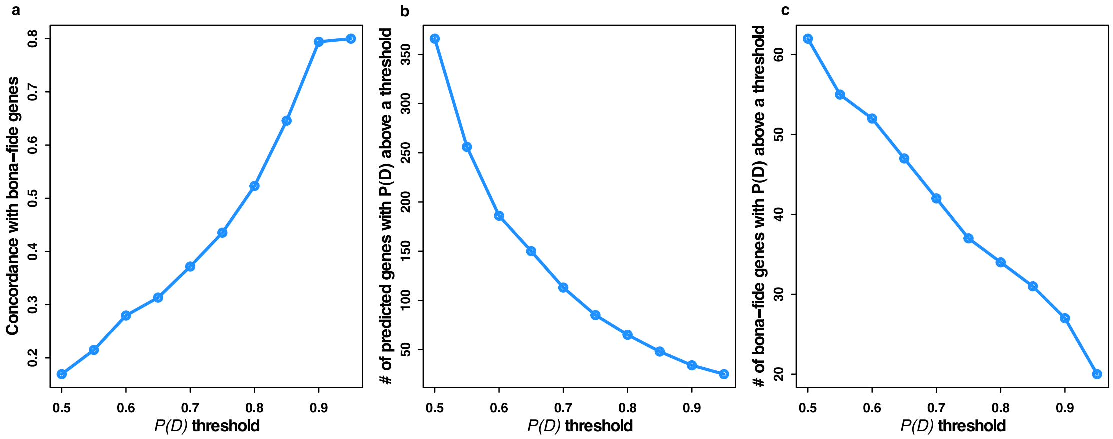


Supplementary Fig. 13: Concordance with the collected *bona fide* driver gene for cis-effect genes. (a) Concordance with *bona fide* driver genes as a function of `xseq` probabilities. (b) The number of genes above a given `xseq` probability threshold. (c) The number of predicted *bona fide* genes above a given `xseq` probability threshold. We think that the threshold of 0.75, 0.8, and 0.85 make a good compromise in predicting novel genes and introducing false-positives. All the 3 thresholds result in concordances above 0.4, and they generate 85, 65, 48 candidate genes, respectively. Here we picked 0.8 and presented the results in manuscript. However, if we chose 0.85, for example, most of the results should still hold, e.g., MuSiC only predicted 20/48 genes.

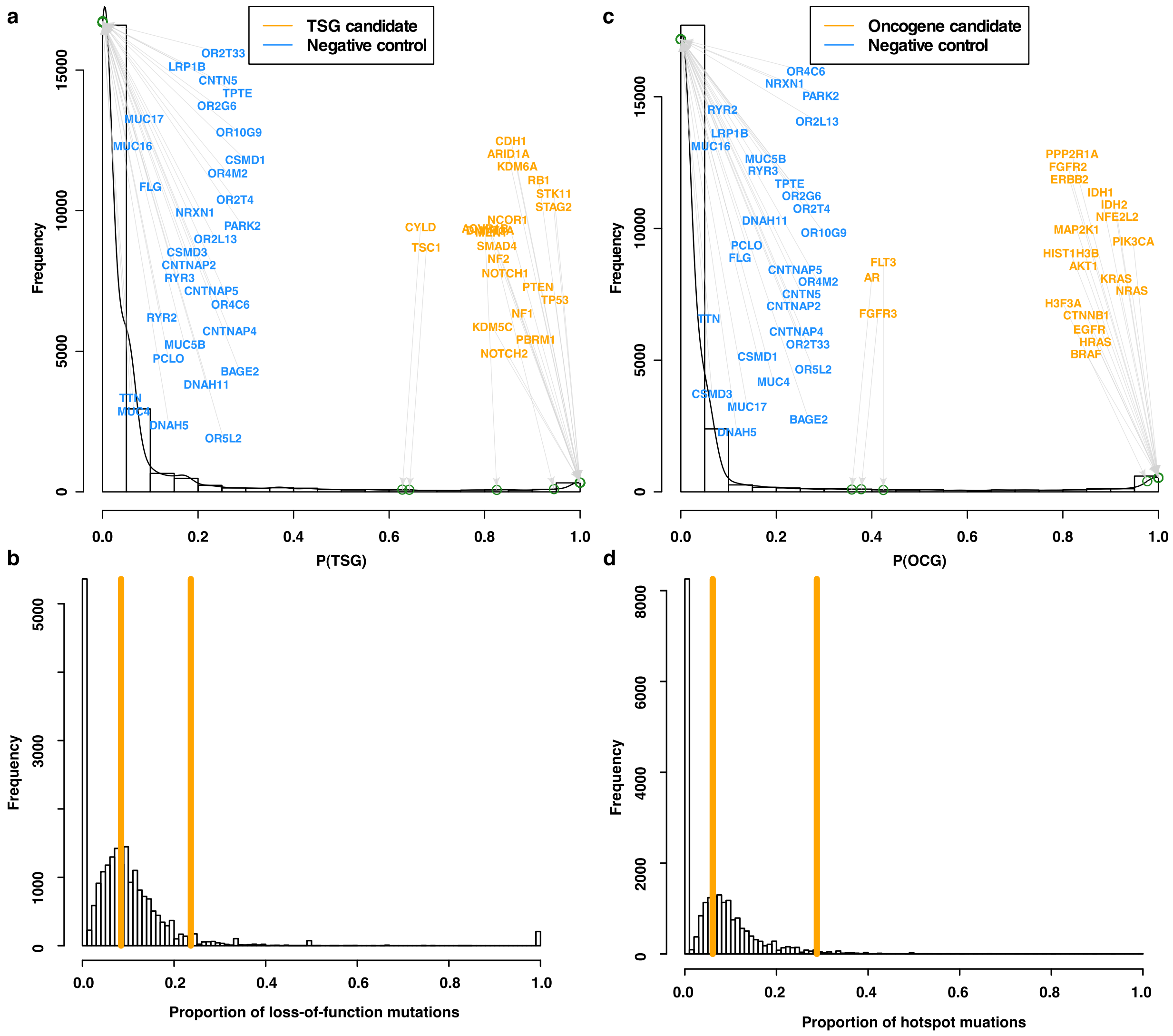


Supplementary Fig. 14: Mixture-of-Binomial modelling of loss-of-function mutations and hotspot mutations. (a) The $P$(TSG) (tumour suppressor gene) posterior distribution. We labeled the $P$(TSG) of the 30 negative control genes (blue colour), as well as 21/23 predicted cis-effect tumour suppressor genes with high $P$(TSG) (orange colour). (b) The proportion of loss-of-function mutations distribution. The two vertical lines represent the estimated success rates of the two binomial distributions (blue: not tumour suppressor genes, orange: tumour suppressor genes). (c) The $P$(OCG) (oncogene) posterior distributions. We also labeled the 30 negative control genes (blue colour), as well as 20 selected oncogenes for illustration purpose. (d) The proportion of recurrent mutation distribution. The two vertical lines represent the estimated success rates of the two binomial distributions (blue: not oncogene, orange: oncogene).

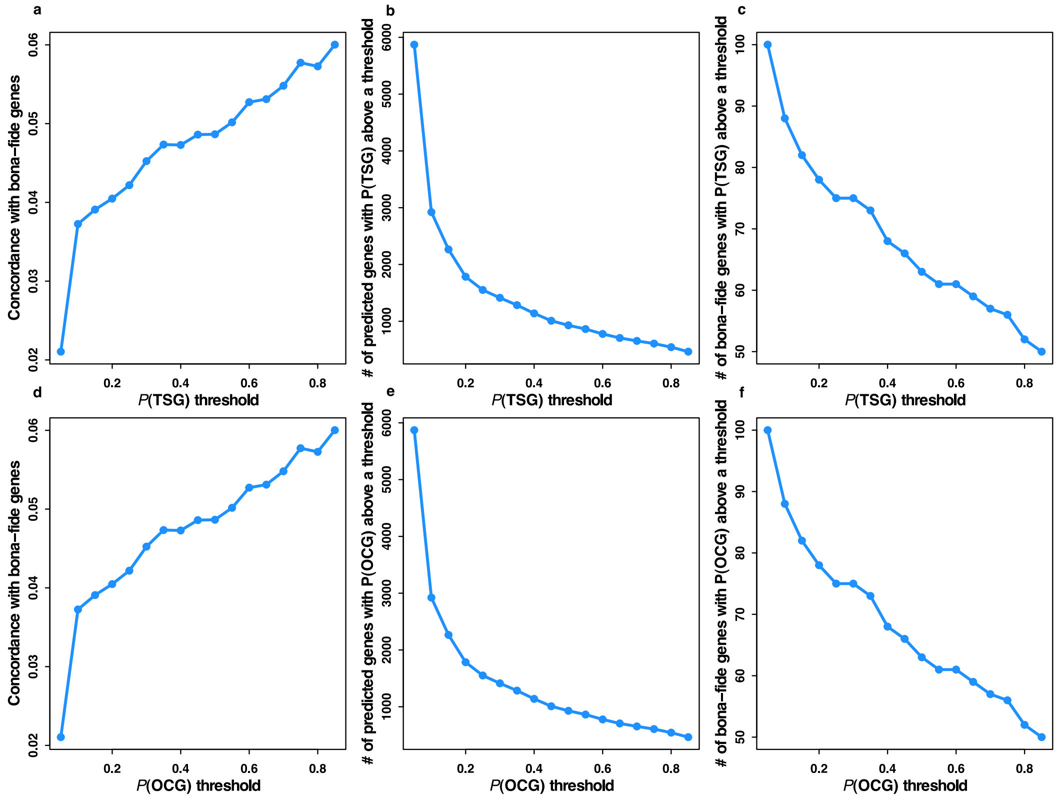


Supplementary Fig. 15: Mixture-of-Binomial predictions concordance with the collected *bona fide* driver gene. (a,d) Concordance with *bona fide* driver genes as a function of classifiers' probabilities. (b,e) The number of genes above a given classifier probability threshold. (c,f) The number of predicted *bona fide* genes above a given classifier probability threshold. The first row (a-c) is for $P$(TSG) and the second row (e-f) is for $P$(OCG). Here we used a probability threshold of 0.2 to call gene harbouring enriched loss-of-function mutations or hot-spot mutations. If we set the threshold below 0.2, the number of predictions increase abruptly. If we used a more conservative threshold, e.g., 0.5, the results did not change much: 51/65 cis genes had $P$(TSG) >= 0.2, and 47/65 genes had $P$(TSG) >= 0.5.

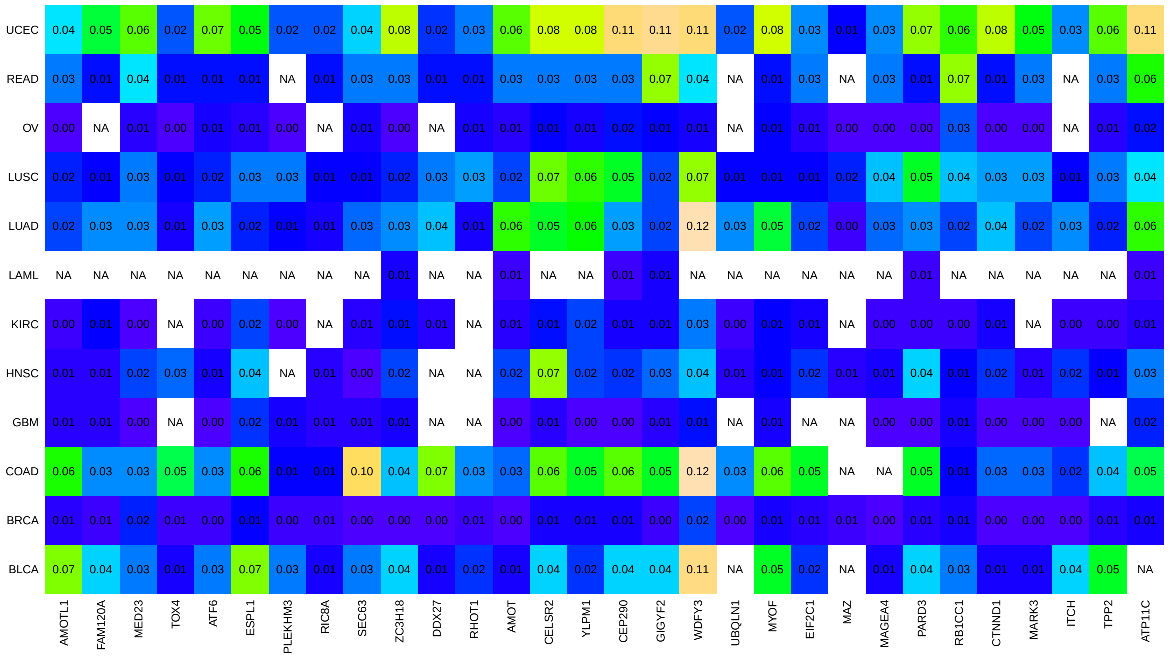


Supplementary Fig. 16: Mutation frequencies of the 30 novel predicted cis-effect genes across 12 tumour types.

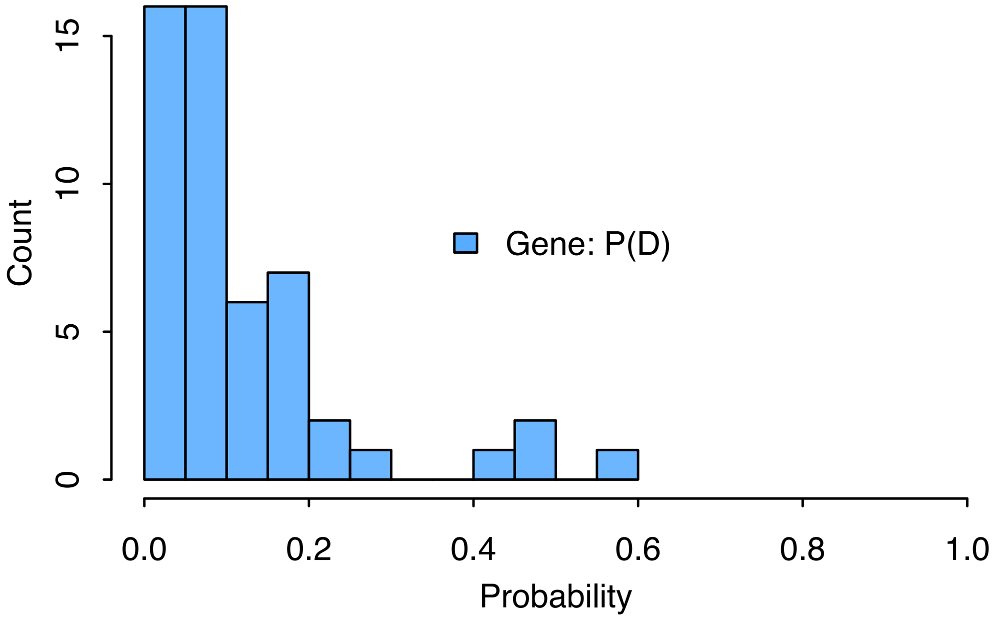


Supplementary Fig. 17: The posterior probability $P(D)$ distribution of the 30 negative control genes across tumour types.

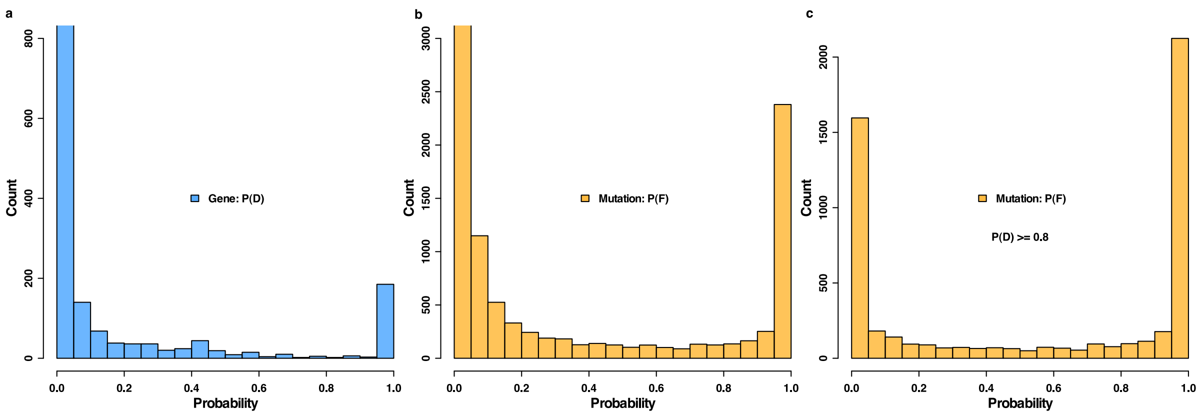


Supplementary Fig. 18: Posterior distributions from trans-analysis. The posterior distributions of (a) $P(D)$, (b) $P(F)$ and (c) $P(F)$ in the high probability genes with $P(D) \geq 0.8$ from trans-analysis. As most genes and mutations had low probabilities, to better visualize the high probability distributions, the y-axises were limited to 800 in (a), and $3,000$ in ($b$).

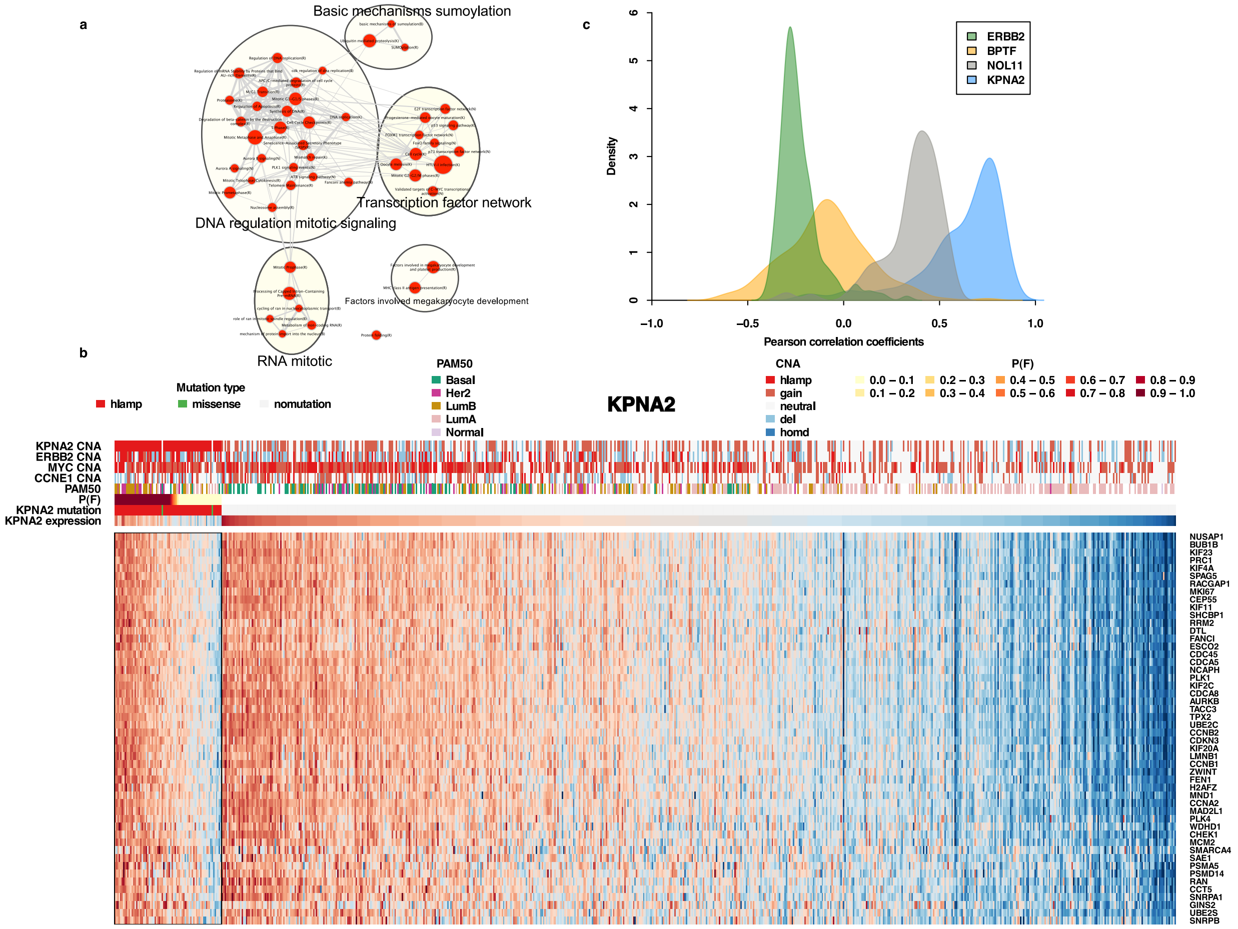


Supplementary Fig. 19: *KPNA2* amplifications in breast cancer correlated with a set of gene up-regulations. (a) These up-regulated genes play roles in DNA-regulation, mitotic signalling, transcription factor networks. (b) The tumours harbouring high-probability *KPNA2* amplifications were mostly Lumina B breast cancer. *KPNA2* expression was highly correlated with the expression of the up-regulated genes. (c) Compared to *KPNA2*, expression of genes in the same loci as *KPNA2* (e.g, *ERBB2, BPTF, NOL11*) had lower correlation with the genes up-regulated with *KPNA2* amplification.

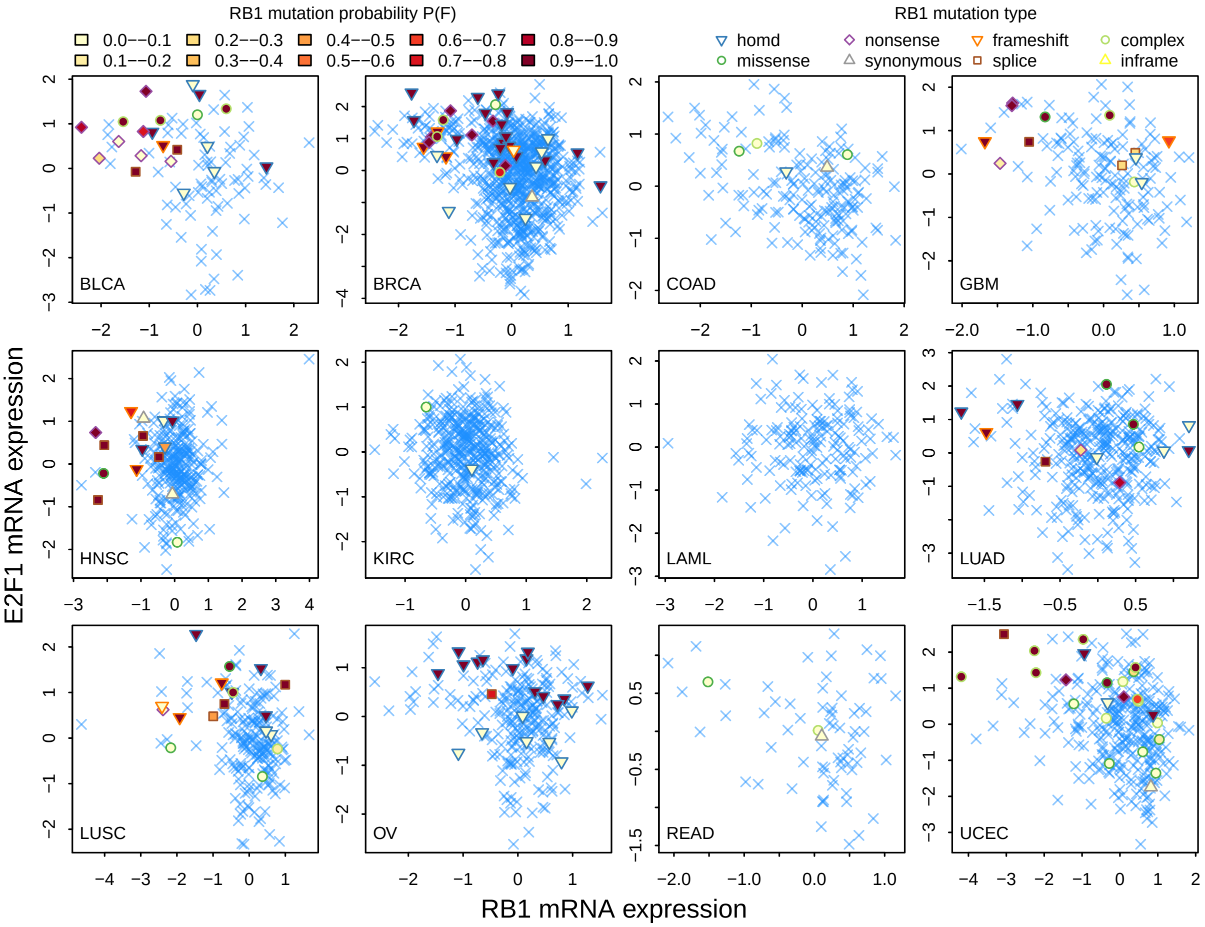


Supplementary Fig. 20: Scatter plots show *RB1* mutations and *E2F1* up-regulation. Each dot represents a patient, and blue crosses mean that those patients did not harbour *RB1* mutations. The posterior marginal $P(F)$ of the *RB1* mutations in different patients are colour coded.

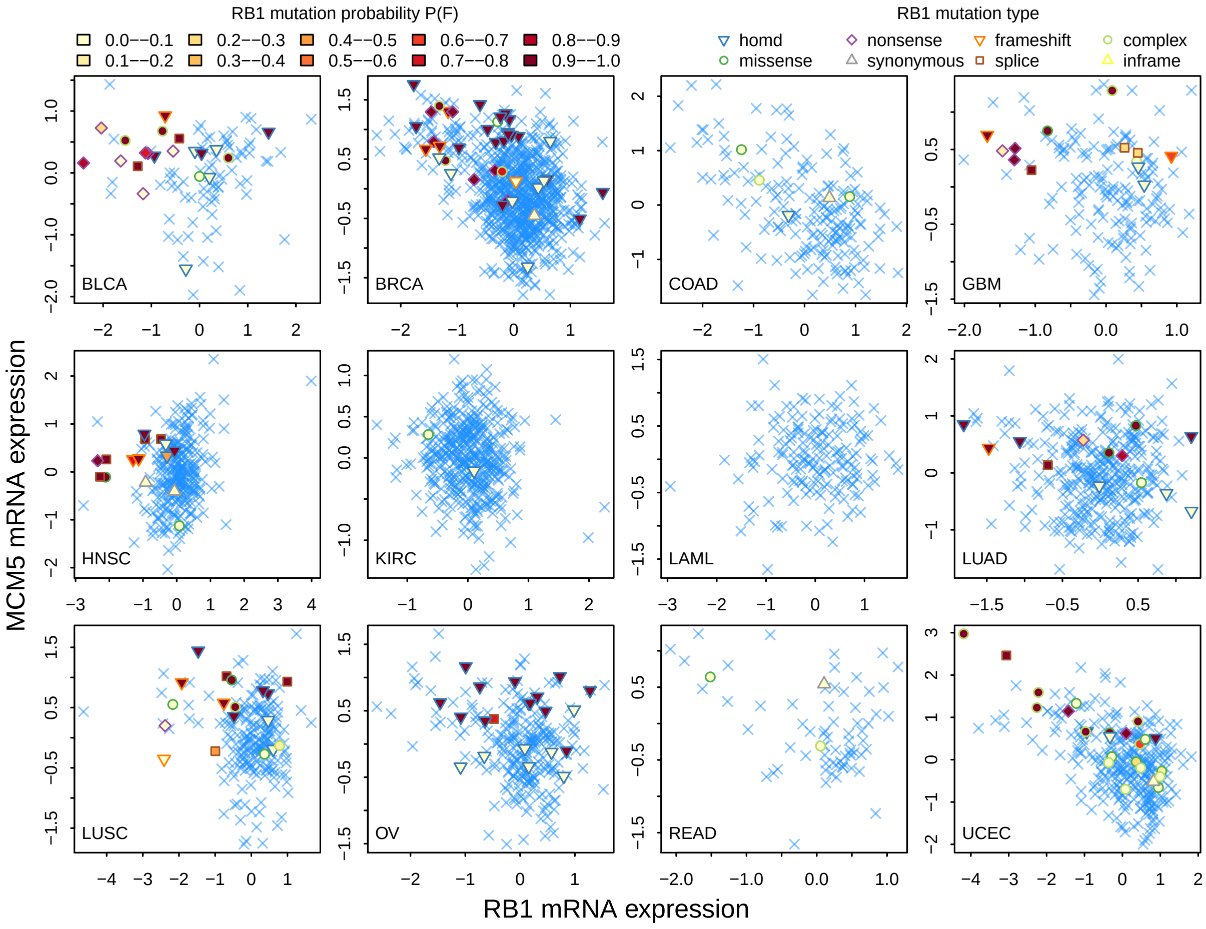


Supplementary Fig. 21: Scatter plots show *RB1* mutations and *MCM5* up-regulation. Each dot represents a patient, and blue crosses mean that those patients did not harbour *RB1* mutations. The posterior marginal $P(F)$ of the *RB1* mutations in different patients are colour coded.

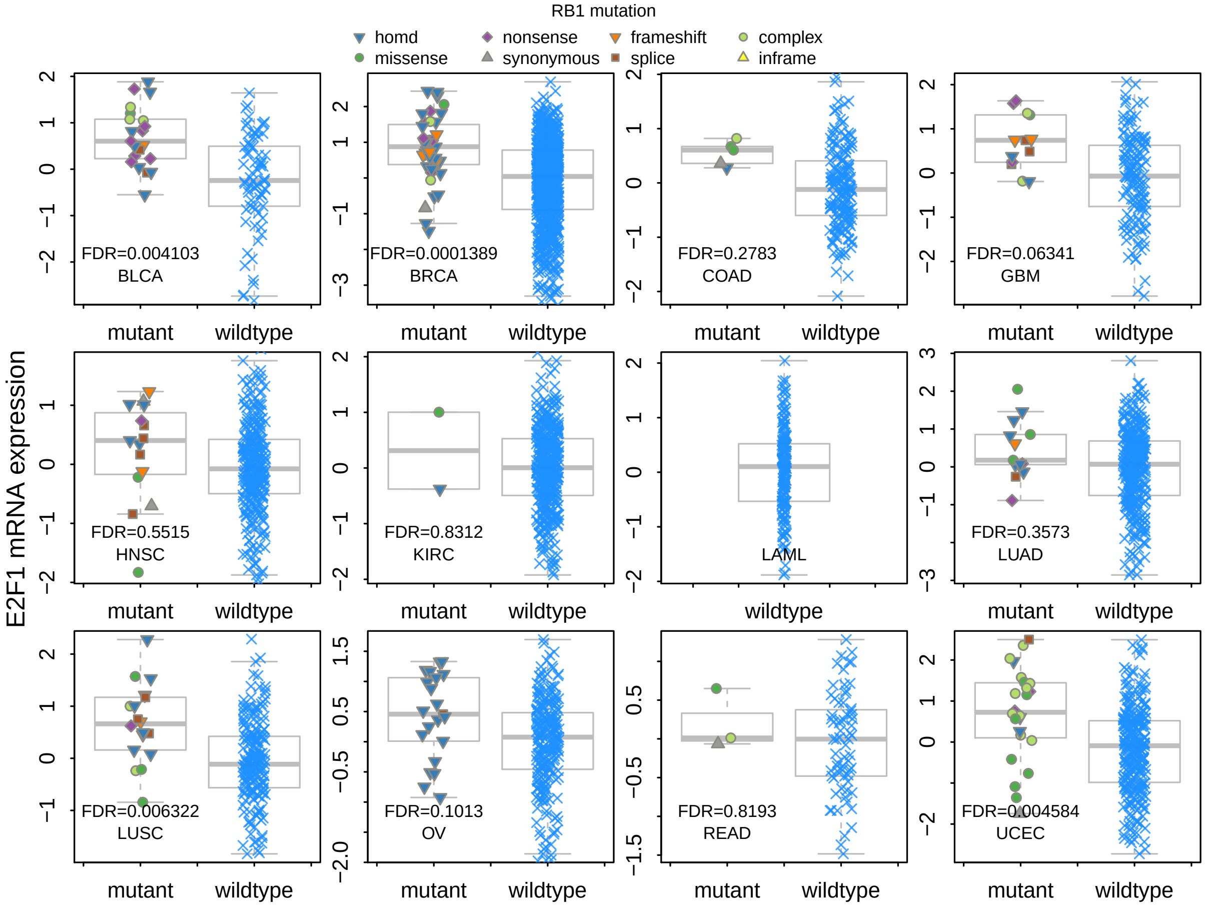


Supplementary Fig. 22: Boxplots show *RB1* mutations and *E2F1* up-regulation. *RB1* mutations correlated with *E2F1* up-regulation in several cancer types, especially BLCA, BRCA, GBM, LUSC and UCEC (FDR < 0.1). Here differential expression analysis was conducted by Limma[1] (Linear Models for Microarray Data).

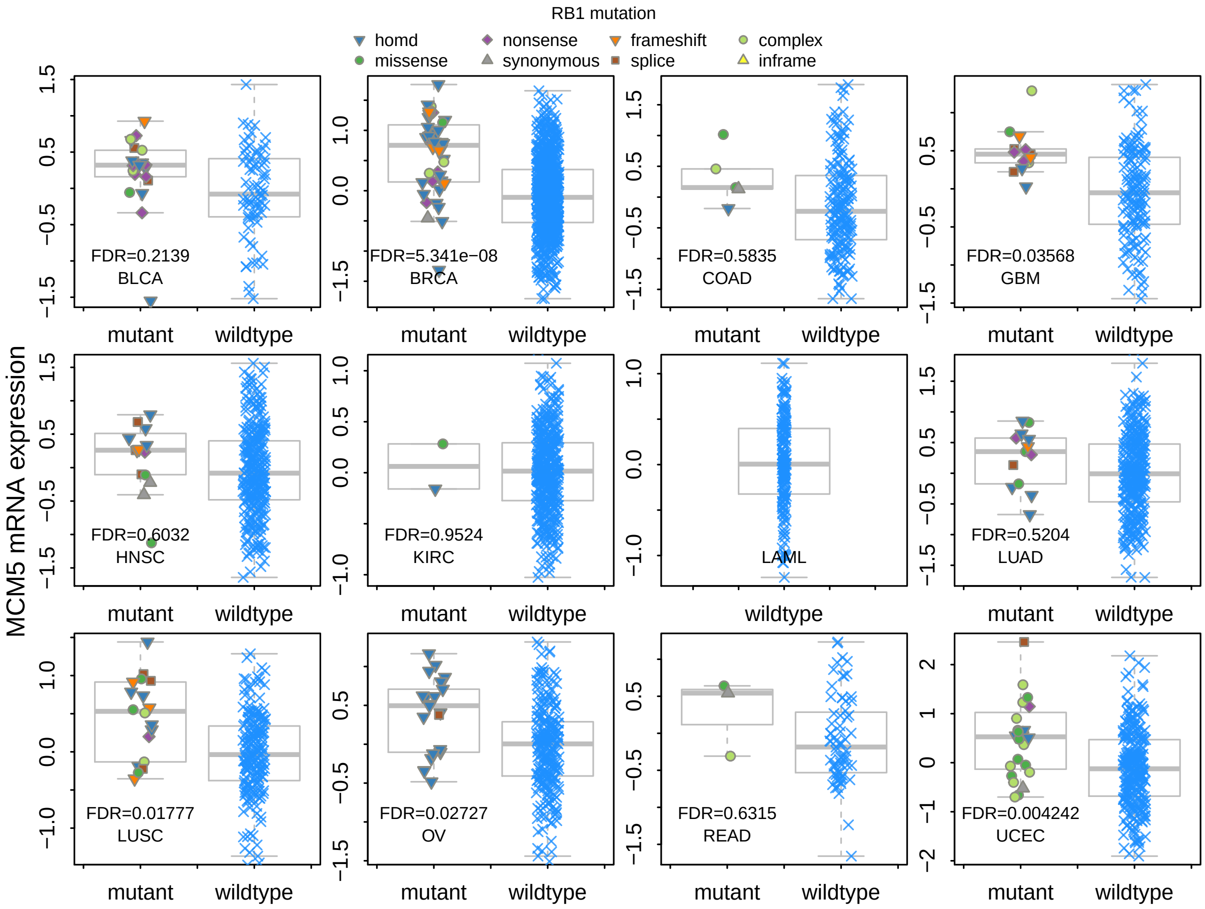


Supplementary Fig. 23: Boxplots show *RB1* mutations and *MCM5* up-regulation. *RB1* mutations correlated with *MCM5* up-regulation in several cancer types, especially BRCA, GBM, LUSC, OV and UCEC (Limma FDR $< 0.1$).

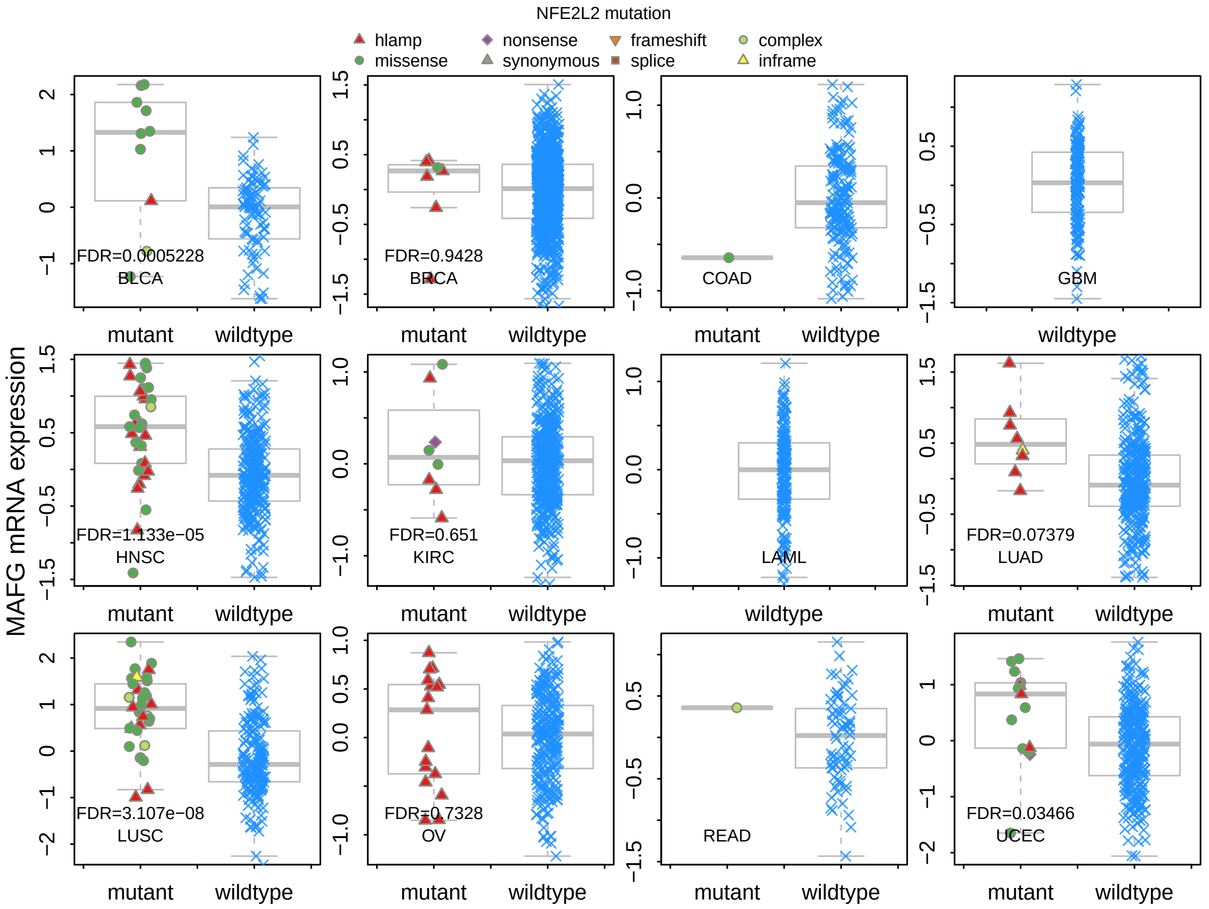


Supplementary Fig. 24: Boxplots show *NFE2L2* mutations and *MAFG* up-regulation. In BLCA, HNSC, LUAD, LUSC, and UCEC, *MAFG* was up-regulated in the patients with *NFE2L2* mutations (Limma FDR $< 0.1$).

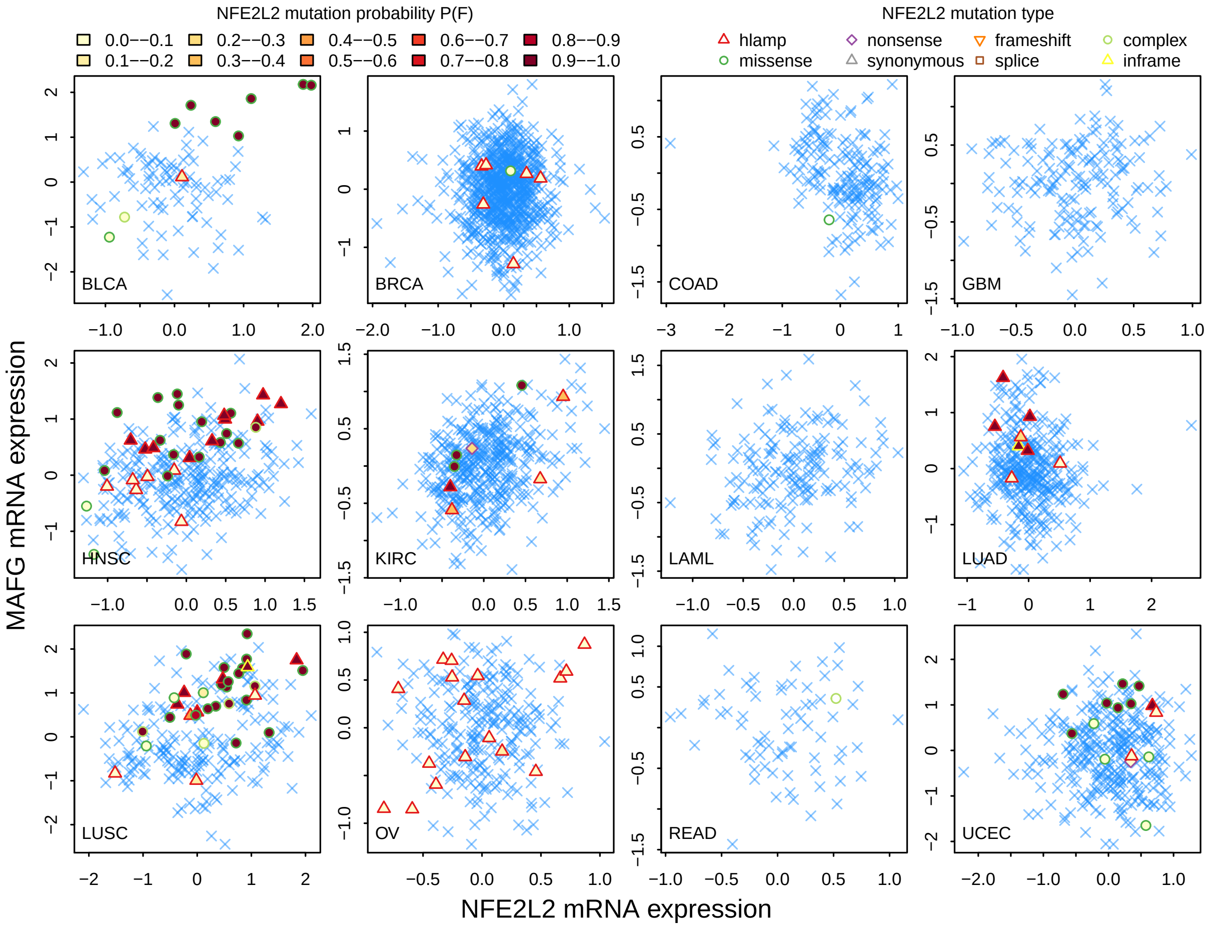


Supplementary Fig. 25: Scatter plots show *NFE2L2* mutations and *MAFG* up-regulation. Each dot represents a patient, and blue crosses mean that those patients did not harbour *NFE2L2* mutations. The posterior marginal $P(F)$ of the *NFE2L2* mutations in different patients are colour coded.

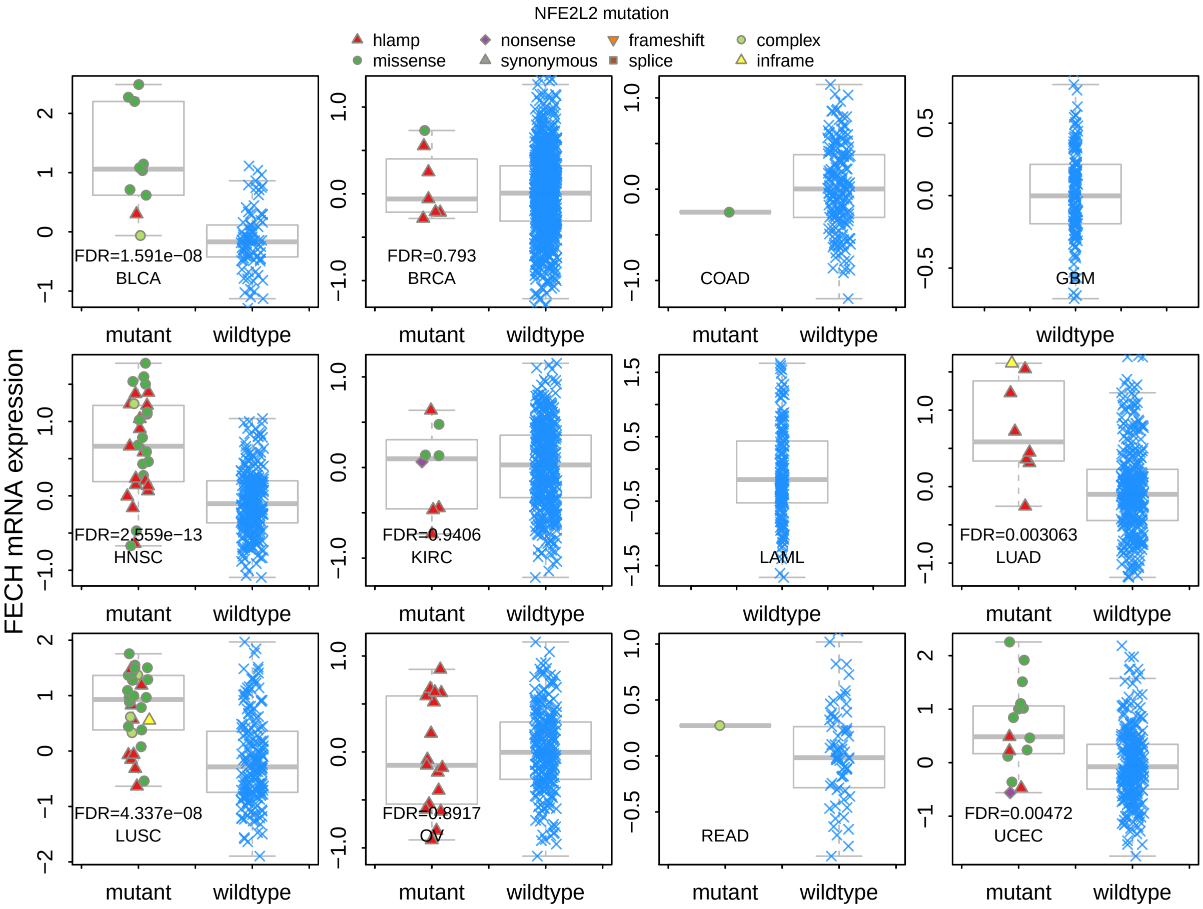


Supplementary Fig. 26: Boxplots show *NFE2L2* mutations and *FECH* up-regulation. In BLCA, HNSC, LUAD, LUSC, and UCEC, *FECH* was up-regulated in the patients with *NFE2L2* mutations or amplifications (Limma FDR < 0.1).

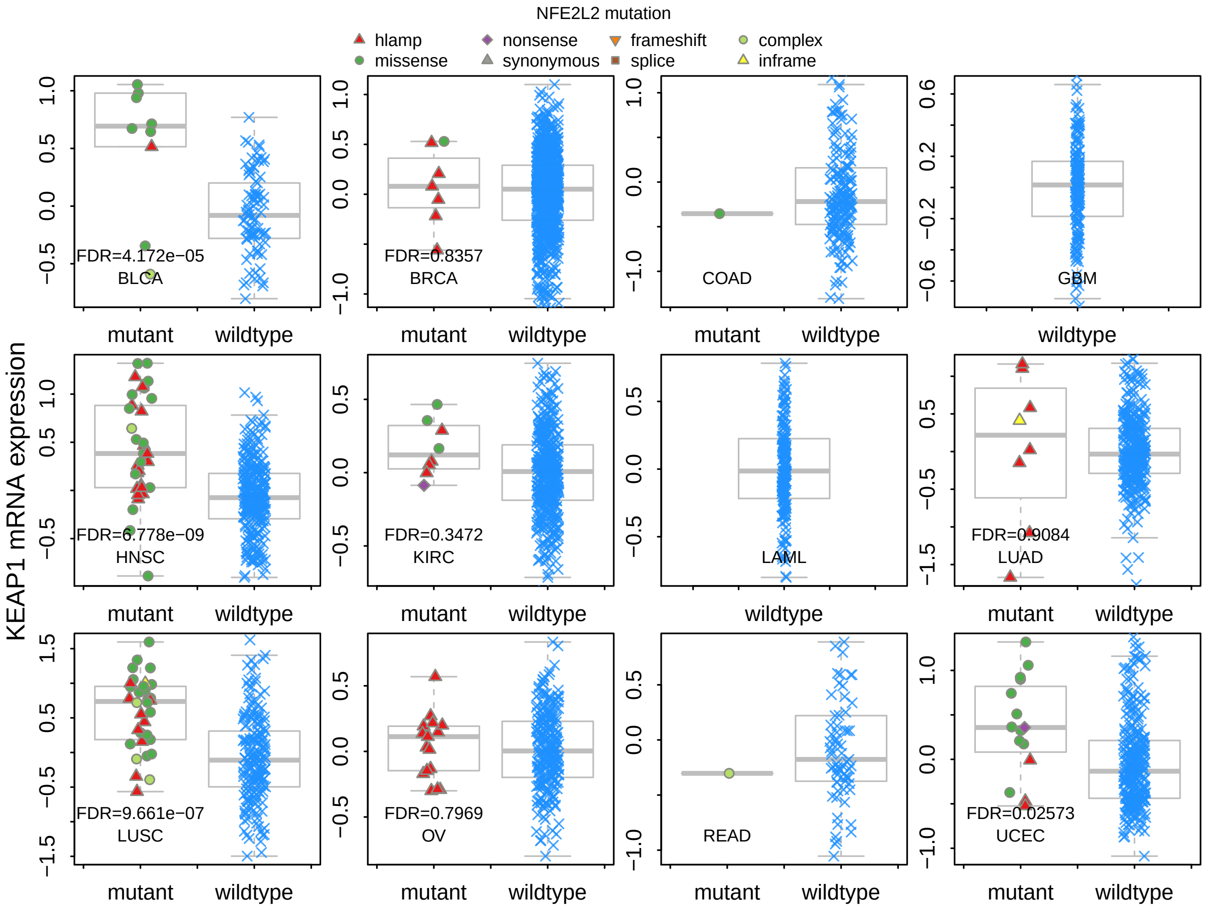


Supplementary Fig. 27: Boxplots show *NFE2L2* mutations and *KEAP1* up-regulation. In BLCA, HNSC, LUSC, and UCEC, *KEAP1* was up-regulated in the patients with *NFE2L2* mutations or amplifications (Limma FDR < 0.1).

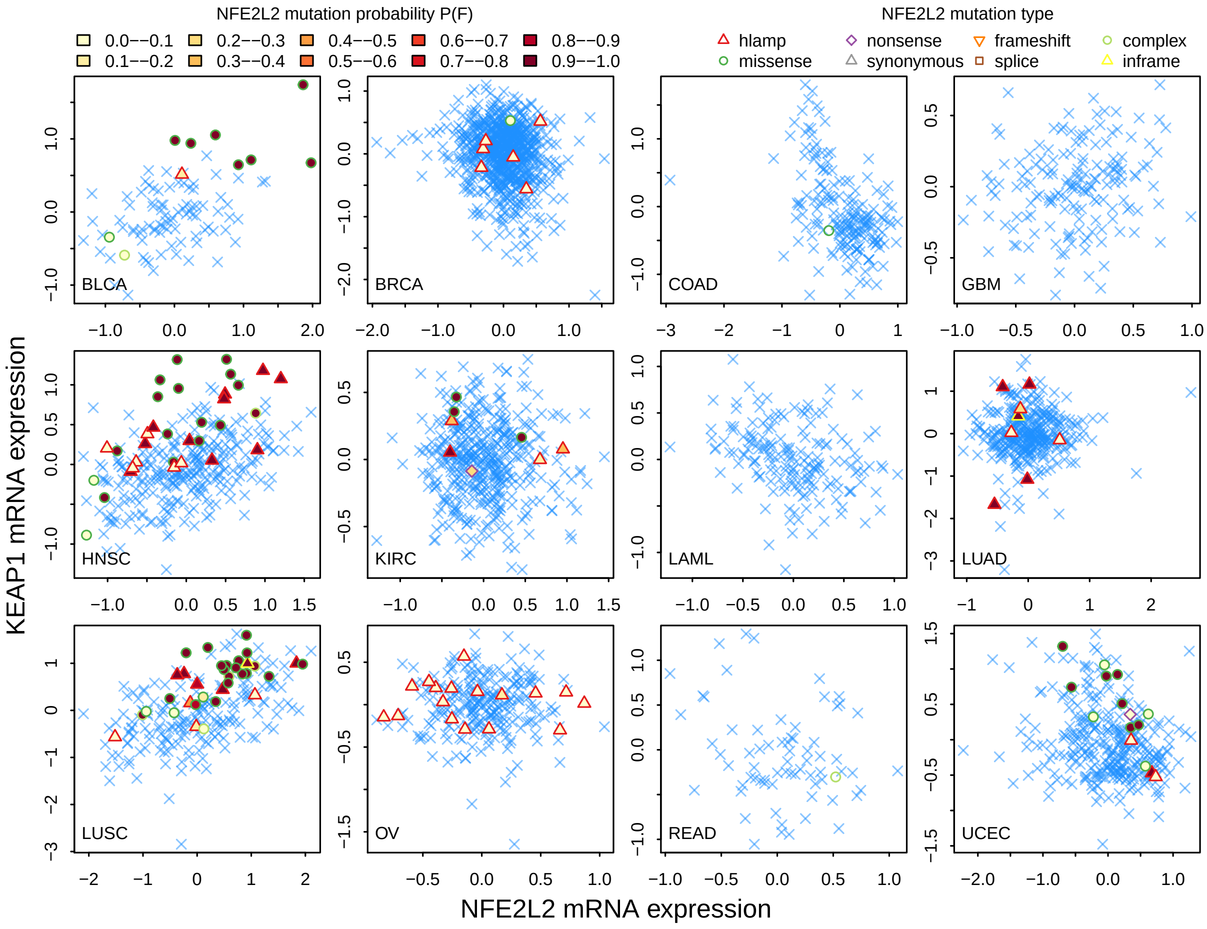


Supplementary Fig. 28: Scatter plots show *NFE2L2* mutations and *KEAP1* up-regulation. Each dot represents a patient, and blue crosses mean that those patients did not harbour *RB1* mutations. The posterior marginal $P(F)$ of the *RB1* mutations in different patients are colour coded.

Supplementary Fig. 29: The trans-effects of bimodal distribution mutations. The heatmaps of the genes connected to the significantly mutated genes whose mutations showed bimodal distributions in `xseq` probabilities $P(F)$.

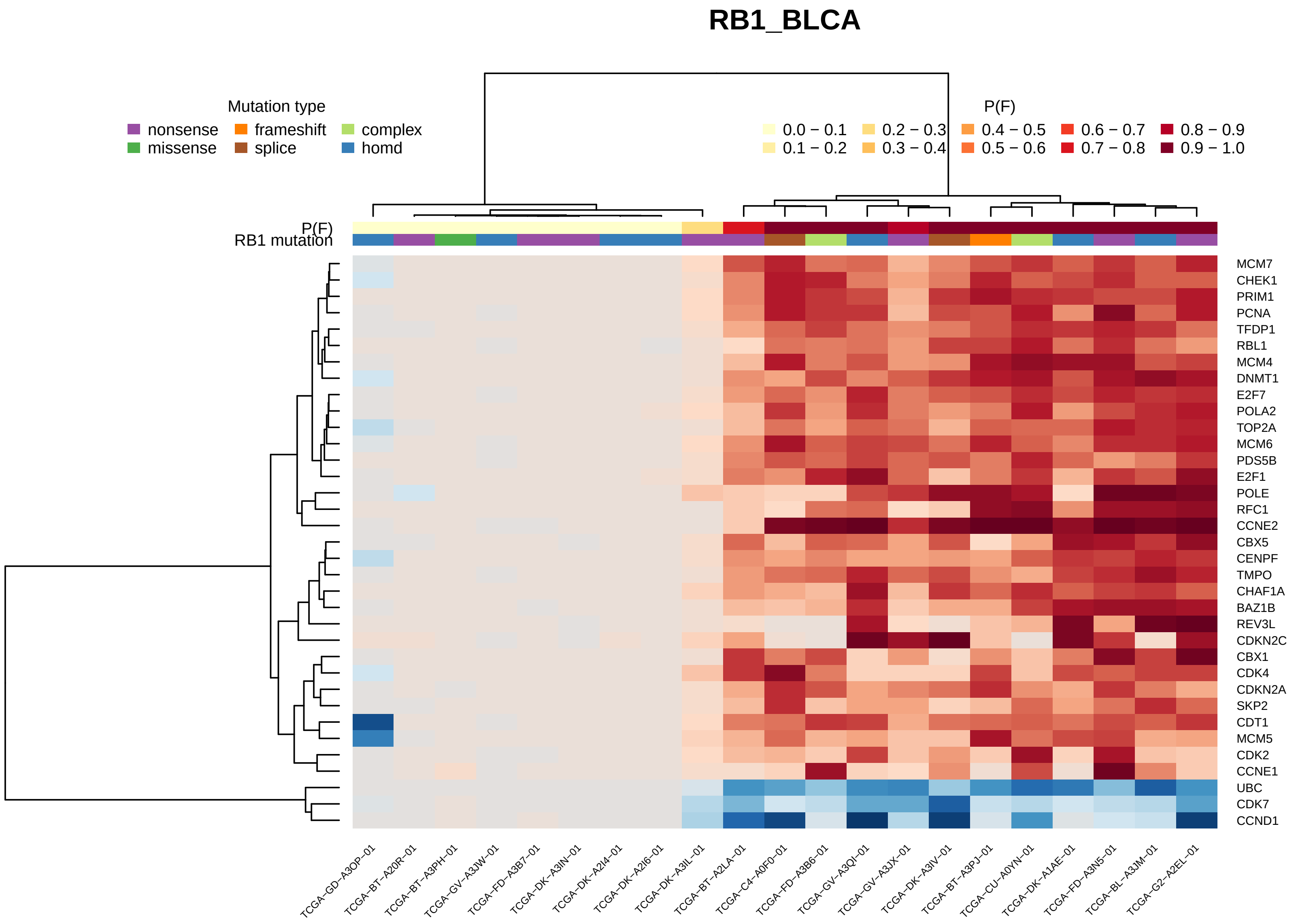
RB1_BLCA
Mutation type
nonsense
frameshift
complex
missense
splice
homd
P(F)
0.0 − 0.1
0.1 − 0.2
0.2 − 0.3
0.3 − 0.4
0.4 − 0.5
0.5 − 0.6
0.6 − 0.7
0.7 − 0.8
0.8 − 0.9
0.9 − 1.0
P(F)
RB1 mutation
MCM7
CHEK1
PRIM1
PCNA
TFDP1
RBL1
MCM4
DNMT1
E2F7
POLA2
TOP2A
MCM6
PDS5B
E2F1
POLE
RFC1
CCNE2
CBX5
CENPF
TMPO
CHAF1A
BAZ1B
REV3L
CDKN2C
CBX1
CDK4
CDKN2A
SKP2
CDT1
MCM5
CDK2
CCNE1
UBC
CDK7
CCND1
TCGA−GD−A3OP−01
TCGA−BT−A20R−01
TCGA−BT−A3PH−01
TCGA−GV−A3JW−01
TCGA−FD−A3B7−01
TCGA−DK−A3IN−01
TCGA−DK−A2I4−01
TCGA−DK−A2I6−01
TCGA−DK−A3IL−01
TCGA−BT−A2LA−01
TCGA−C4−A0F0−01
TCGA−FD−A3B6−01
TCGA−GV−A3QI−01
TCGA−GV−A3JX−01
TCGA−DK−A3IV−01
TCGA−BT−A3PJ−01
TCGA−CU−A0YN−01
TCGA−DK−A1AE−01
TCGA−FD−A3N5−01
TCGA−BL−A3JM−01
TCGA−G2−A2EL−01

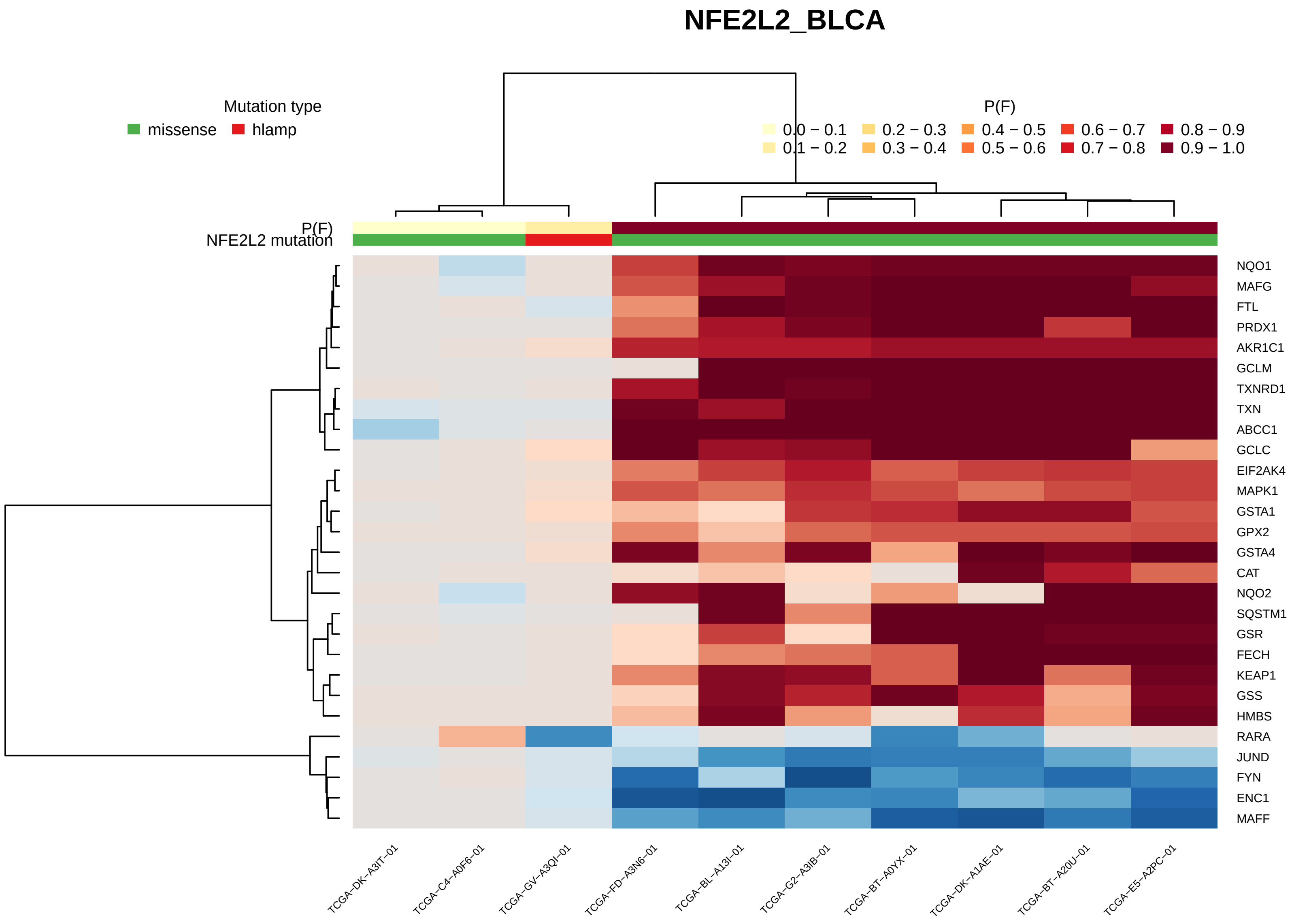

NFE2L2_BLCA
Mutation type
missense
hlamp
P(F)
0.0 − 0.1
0.1 − 0.2
0.2 − 0.3
0.3 − 0.4
0.4 − 0.5
0.5 − 0.6
0.6 − 0.7
0.7 − 0.8
0.8 − 0.9
0.9 − 1.0
P(F)
NFE2L2 mutation
NQO1
MAFG
FTL
PRDX1
AKR1C1
GCLM
TXNRD1
TXN
ABCC1
GCLC
EIF2AK4
MAPK1
GSTA1
GPX2
GSTA4
CAT
NQO2
SQSTM1
GSR
FECH
KEAP1
GSS
HMBS
RARA
JUND
FYN
ENC1
MAFF
TCGA−DK−A3IT−01
TCGA−C4−A0F6−01
TCGA−GV−A3QI−01
TCGA−FD−A3N6−01
TCGA−BL−A13I−01
TCGA−G2−A3IB−01
TCGA−BT−A0YX−01
TCGA−DK−A1AE−01
TCGA−BT−A20U−01
TCGA−E5−A2PC−01

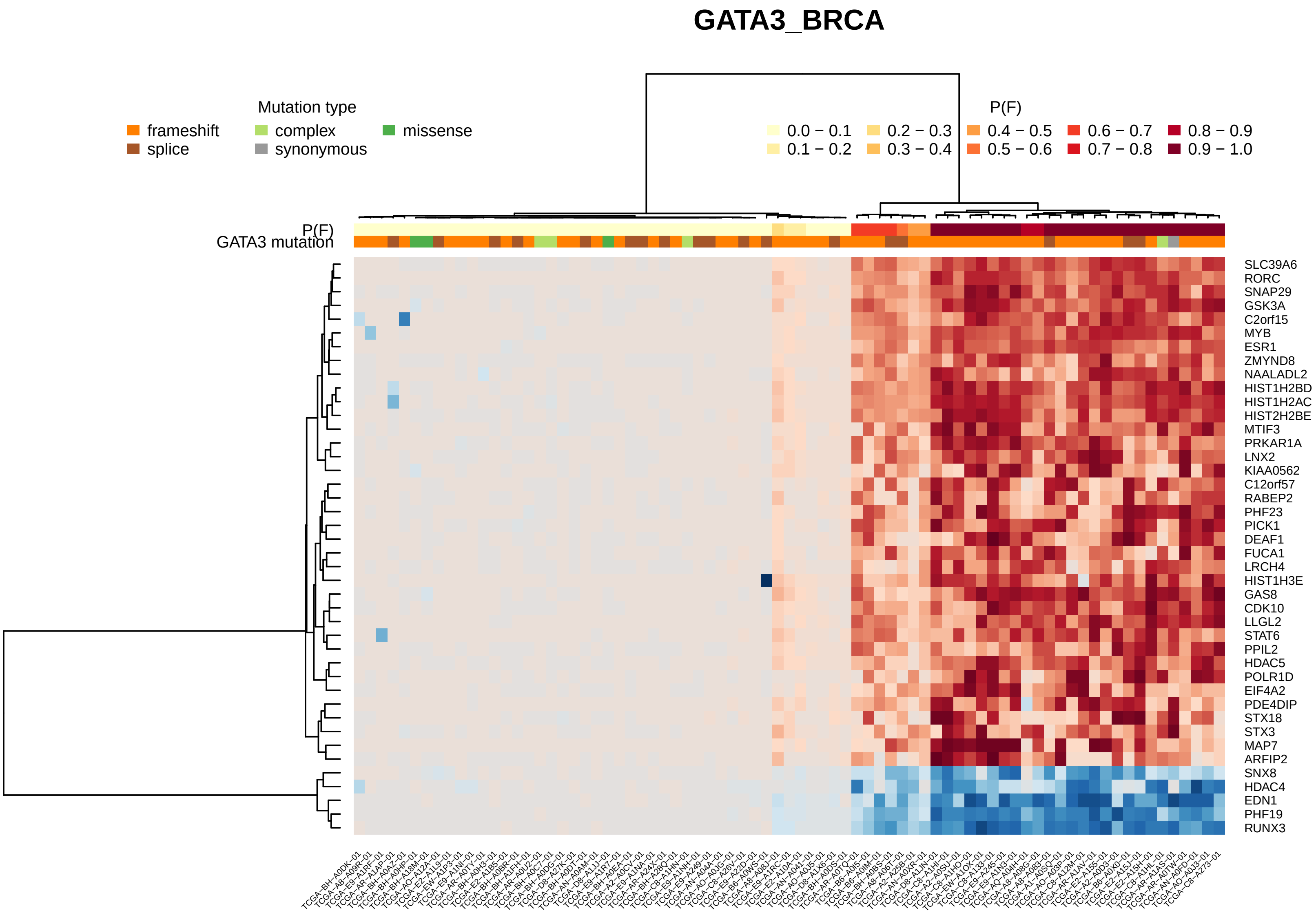
GATA3_BRCA
Mutation type
frameshift
splice
complex
synonymous
missense
P(F)
0.0 − 0.1
0.1 − 0.2
0.2 − 0.3
0.3 − 0.4
0.4 − 0.5
0.5 − 0.6
0.6 − 0.7
0.7 − 0.8
0.8 − 0.9
0.9 − 1.0
P(F)
GATA3 mutation
SLC39A6
RORC
SNAP29
GSK3A
C2orf15
MYB
ESR1
ZMYND8
NAALADL2
HIST1H2BD
HIST1H2AC
HIST2H2BE
MTIF3
PRKAR1A
LNX2
KIAA0562
C12orf57
RABEP2
PHF23
PICK1
DEAF1
FUCA1
LRCH4
HIST1H3E
GAS8
CDK10
LLGL2
STAT6
PPIL2
HDAC5
POLR1D
EIF4A2
PDE4DIP
STX18
STX3
MAP7
ARFIP2
SNX8
HDAC4
EDN1
PHF19
RUNX3

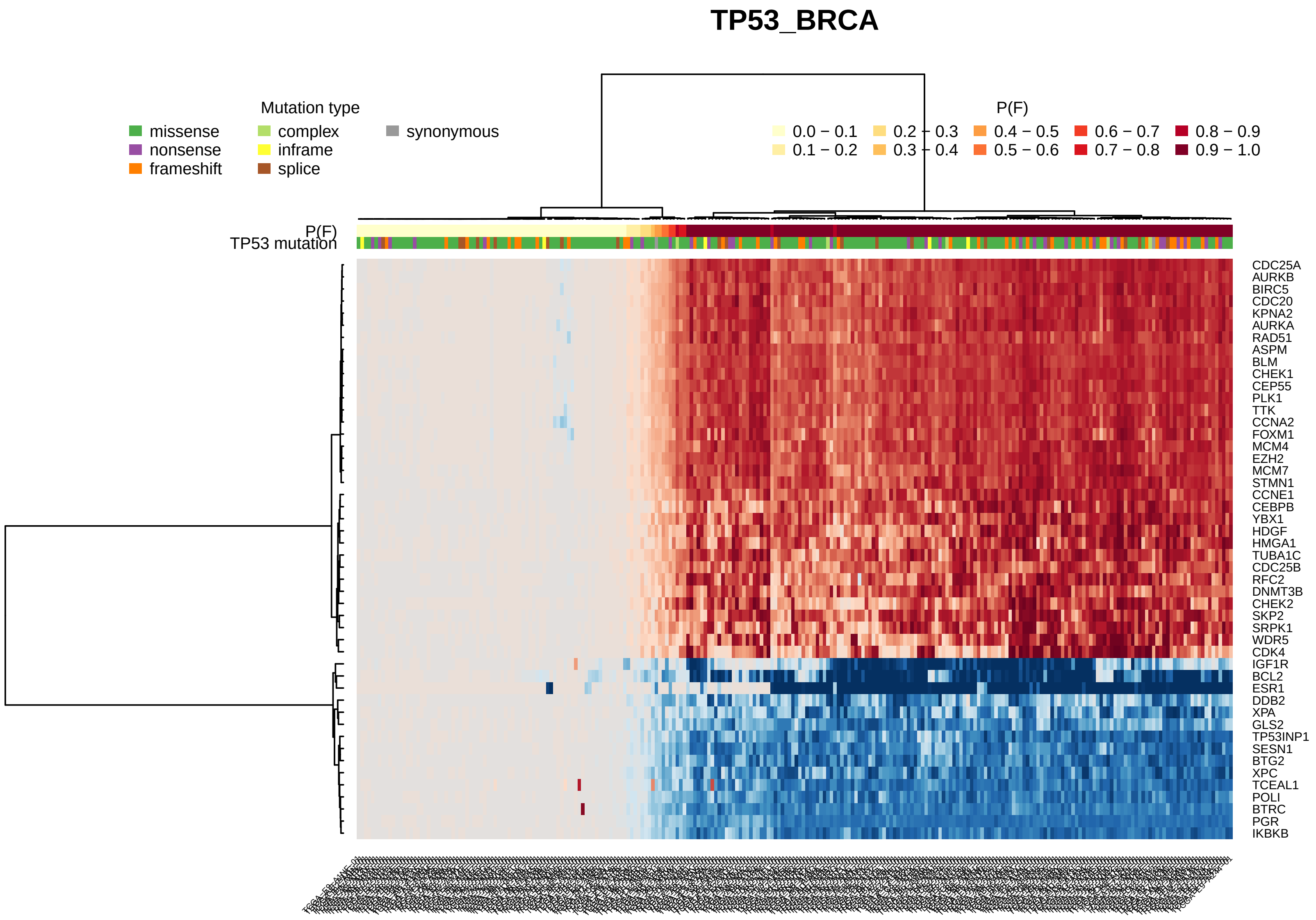

TP53_BRCA
Mutation type
missense
nonsense
frameshift
complex
inframe
splice
synonymous
P(F)
0.0 − 0.1
0.1 − 0.2
0.2 − 0.3
0.3 − 0.4
0.4 − 0.5
0.5 − 0.6
0.6 − 0.7
0.7 − 0.8
0.8 − 0.9
0.9 − 1.0
P(F)
TP53 mutation
CDC25A
AURKB
BIRC5
CDC20
KPNA2
AURKA
RAD51
ASPM
BLM
CHEK1
CEP55
PLK1
TTK
CCNA2
FOXM1
MCM4
EZH2
MCM7
STMN1
CCNE1
CEBPB
YBX1
HDGF
HMGA1
TUBA1C
CDC25B
RFC2
DNMT3B
CHEK2
SKP2
SRPK1
WDR5
CDK4
IGF1R
BCL2
ESR1
DDB2
XPA
GLS2
TP53INP1
SESN1
BTG2
XPC
TCEAL1
POLI
BTRC
PGR
IKBKB

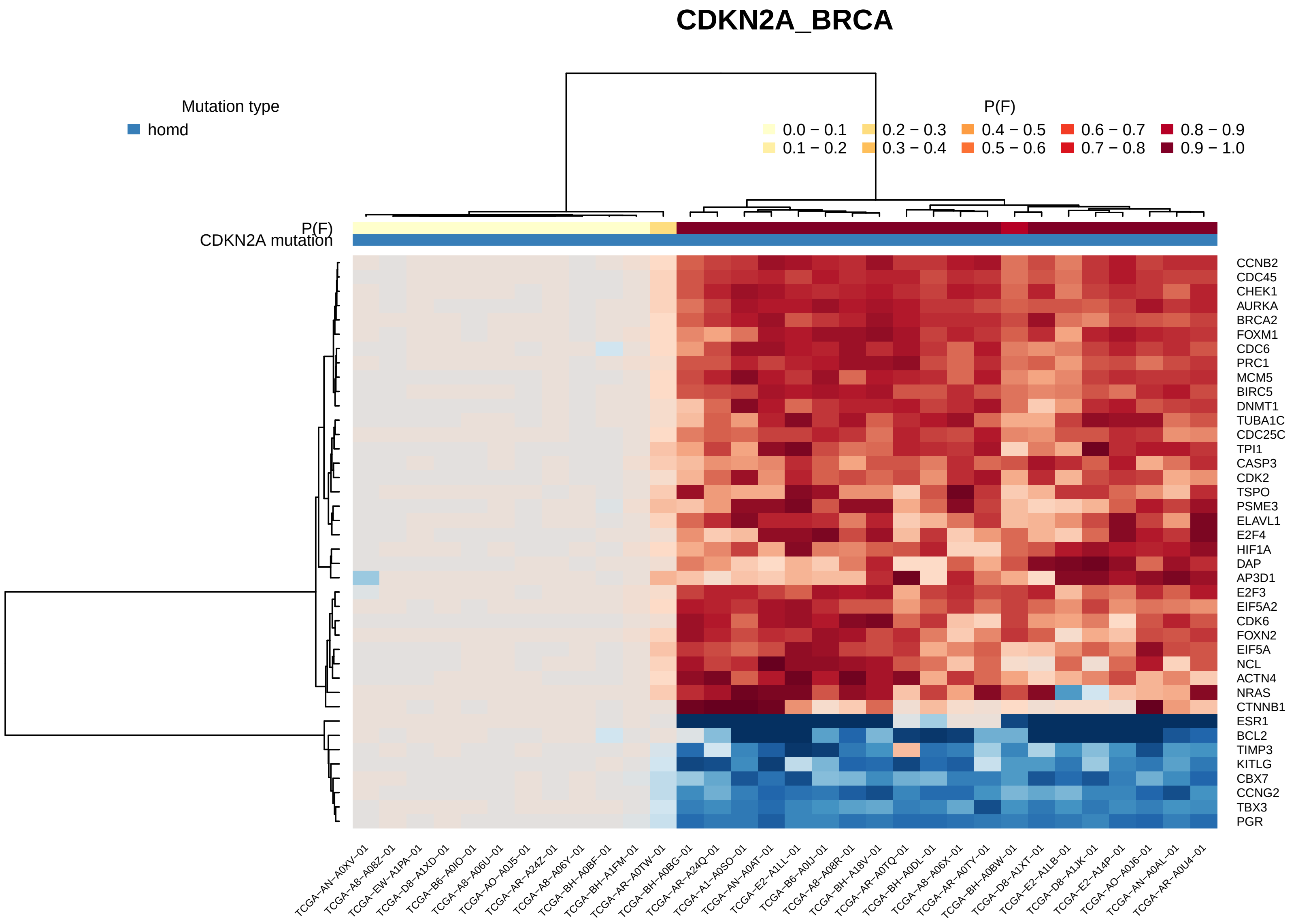

CDKN2A_BRCA
Mutation type
homd
P(F)
0.0 − 0.1
0.1 − 0.2
0.2 − 0.3
0.3 − 0.4
0.4 − 0.5
0.5 − 0.6
0.6 − 0.7
0.7 − 0.8
0.8 − 0.9
0.9 − 1.0
P(F)
CDKN2A mutation
CCNB2
CDC45
CHEK1
AURKA
BRCA2
FOXM1
CDC6
PRC1
MCM5
BIRC5
DNMT1
TUBA1C
CDC25C
TPI1
CASP3
CDK2
TSPO
PSME3
ELAVL1
E2F4
HIF1A
DAP
AP3D1
E2F3
EIF5A2
CDK6
FOXN2
EIF5A
NCL
ACTN4
NRAS
CTNNB1
ESR1
BCL2
TIMP3
KITLG
CBX7
CCNG2
TBX3
PGR
TCGA−AN−A0XV−01
TCGA−A8−A08Z−01
TCGA−EW−A1PA−01
TCGA−D8−A1XD−01
TCGA−B6−A0IO−01
TCGA−A8−A06U−01
TCGA−AO−A0J5−01
TCGA−AR−A24Z−01
TCGA−A8−A06Y−01
TCGA−BH−A0BF−01
TCGA−BH−A1FM−01
TCGA−AR−A0TW−01
TCGA−BH−A0BG−01
TCGA−AR−A24Q−01
TCGA−A1−A0SO−01
TCGA−AN−A0AT−01
TCGA−E2−A1LL−01
TCGA−B6−A0IJ−01
TCGA−A8−A08R−01
TCGA−BH−A18V−01
TCGA−AR−A0TQ−01
TCGA−BH−A0DL−01
TCGA−A8−A06X−01
TCGA−AR−A0TY−01
TCGA−BH−A0BW−01
TCGA−D8−A1XT−01
TCGA−E2−A1LB−01
TCGA−D8−A1JK−01
TCGA−E2−A14P−01
TCGA−AO−A0J6−01
TCGA−AN−A0AL−01
TCGA−AR−A0U4−01

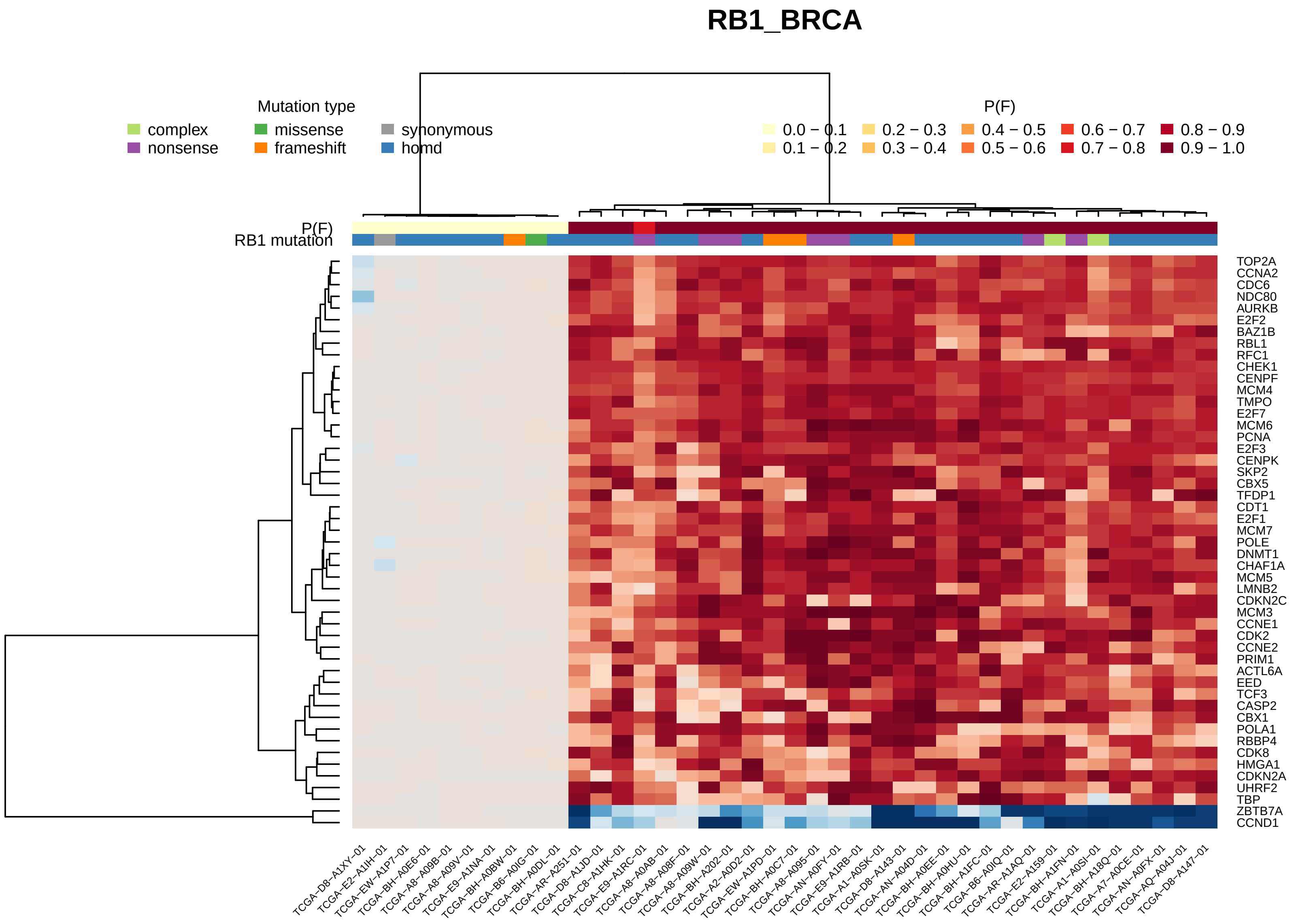
RB1_BRCA
Mutation type
complex
nonsense
missense
frameshift
synonymous
homd
P(F)
0.0 − 0.1
0.1 − 0.2
0.2 − 0.3
0.3 − 0.4
0.4 − 0.5
0.5 − 0.6
0.6 − 0.7
0.7 − 0.8
0.8 − 0.9
0.9 − 1.0
P(F)
RB1 mutation
TOP2A
CCNA2
CDC6
NDC80
AURKB
E2F2
BAZ1B
RBL1
RFC1
CHEK1
CENPF
MCM4
TMPO
E2F7
MCM6
PCNA
E2F3
CENPK
SKP2
CBX5
TFDP1
CDT1
E2F1
MCM7
POLE
DNMT1
CHAF1A
MCM5
LMNB2
CDKN2C
MCM3
CCNE1
CDK2
CCNE2
PRIM1
ACTL6A
EED
TCF3
CASP2
CBX1
POLA1
RBBP4
CDK8
HMGA1
CDKN2A
UHRF2
TBP
ZBTB7A
CCND1
TCGA−D8−A1XY−01
TCGA−E2−A1IH−01
TCGA−EW−A1P7−01
TCGA−BH−A0E6−01
TCGA−A8−A09B−01
TCGA−A8−A09V−01
TCGA−E9−A1NA−01
TCGA−BH−A0BW−01
TCGA−B6−A0IG−01
TCGA−BH−A0DL−01
TCGA−AR−A251−01
TCGA−D8−A1JD−01
TCGA−C8−A1HK−01
TCGA−E9−A1RC−01
TCGA−A8−A0AB−01
TCGA−A8−A08F−01
TCGA−A8−A09W−01
TCGA−BH−A202−01
TCGA−A2−A0D2−01
TCGA−EW−A1PD−01
TCGA−BH−A0C7−01
TCGA−A8−A095−01
TCGA−AN−A0FY−01
TCGA−E9−A1RB−01
TCGA−A1−A0SK−01
TCGA−D8−A143−01
TCGA−AN−A04D−01
TCGA−BH−A0EE−01
TCGA−BH−A0HU−01
TCGA−BH−A1FC−01
TCGA−B6−A0IQ−01
TCGA−AR−A1AQ−01
TCGA−E2−A159−01
TCGA−BH−A1FN−01
TCGA−A1−A0SI−01
TCGA−BH−A18Q−01
TCGA−A7−A0CE−01
TCGA−AN−A0FX−01
TCGA−AQ−A04J−01
TCGA−D8−A147−01

# CCND1_BRCA

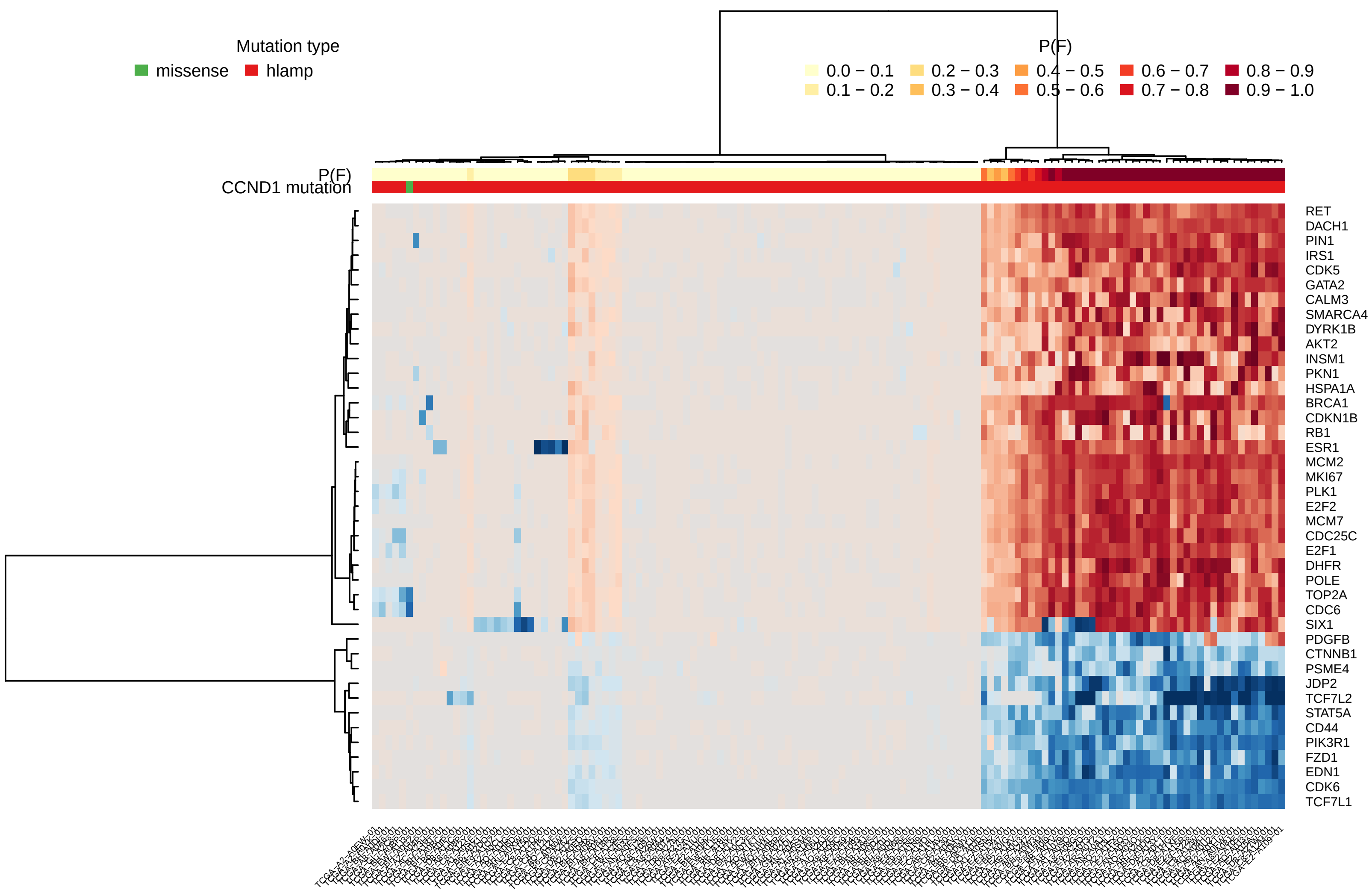
Mutation type
missense
hlamp
P(F)
0.0 − 0.1
0.1 − 0.2
0.2 − 0.3
0.3 − 0.4
0.4 − 0.5
0.5 − 0.6
0.6 − 0.7
0.7 − 0.8
0.8 − 0.9
0.9 − 1.0
P(F)
CCND1 mutation
RET
DACH1
PIN1
IRS1
CDK5
GATA2
CALM3
SMARCA4
DYRK1B
AKT2
INSM1
PKN1
HSPA1A
BRCA1
CDKN1B
RB1
ESR1
MCM2
MKI67
PLK1
E2F2
MCM7
CDC25C
E2F1
DHFR
POLE
TOP2A
CDC6
SIX1
PDGFB
CTNNB1
PSME4
JDP2
TCF7L2
STAT5A
CD44
PIK3R1
FZD1
EDN1
CDK6
TCF7L1

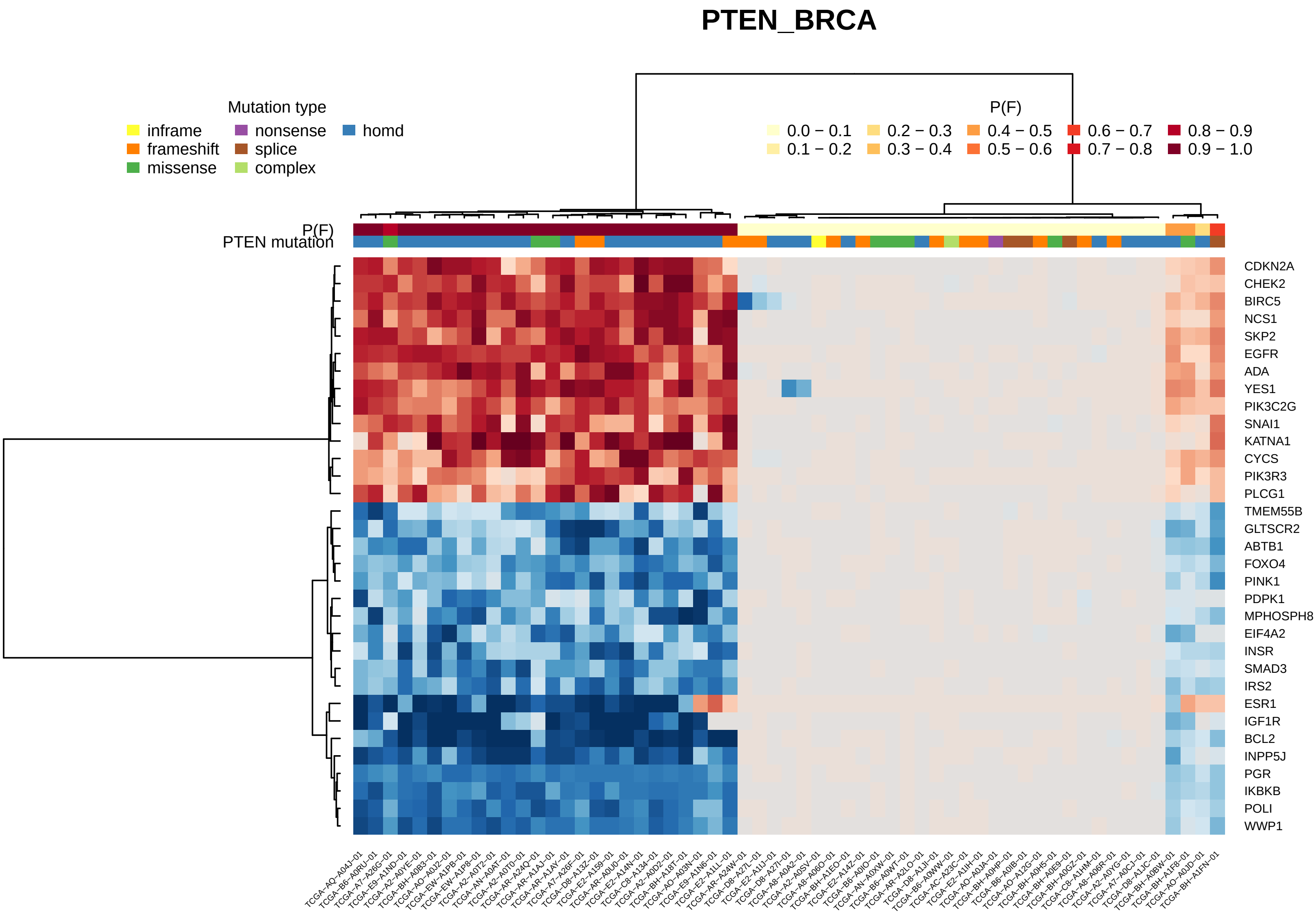
PTEN_BRCA
Mutation type
inframe
frameshift
missense
nonsense
splice
complex
homd
P(F)
0.0 − 0.1
0.1 − 0.2
0.2 − 0.3
0.3 − 0.4
0.4 − 0.5
0.5 − 0.6
0.6 − 0.7
0.7 − 0.8
0.8 − 0.9
0.9 − 1.0
P(F)
PTEN mutation
CDKN2A
CHEK2
BIRC5
NCS1
SKP2
EGFR
ADA
YES1
PIK3C2G
SNAI1
KATNA1
CYCS
PIK3R3
PLCG1
TMEM55B
GLTSCR2
ABTB1
FOXO4
PINK1
PDPK1
MPHOSPH8
EIF4A2
INSR
SMAD3
IRS2
ESR1
IGF1R
BCL2
INPP5J
PGR
IKBKB
POLI
WWP1

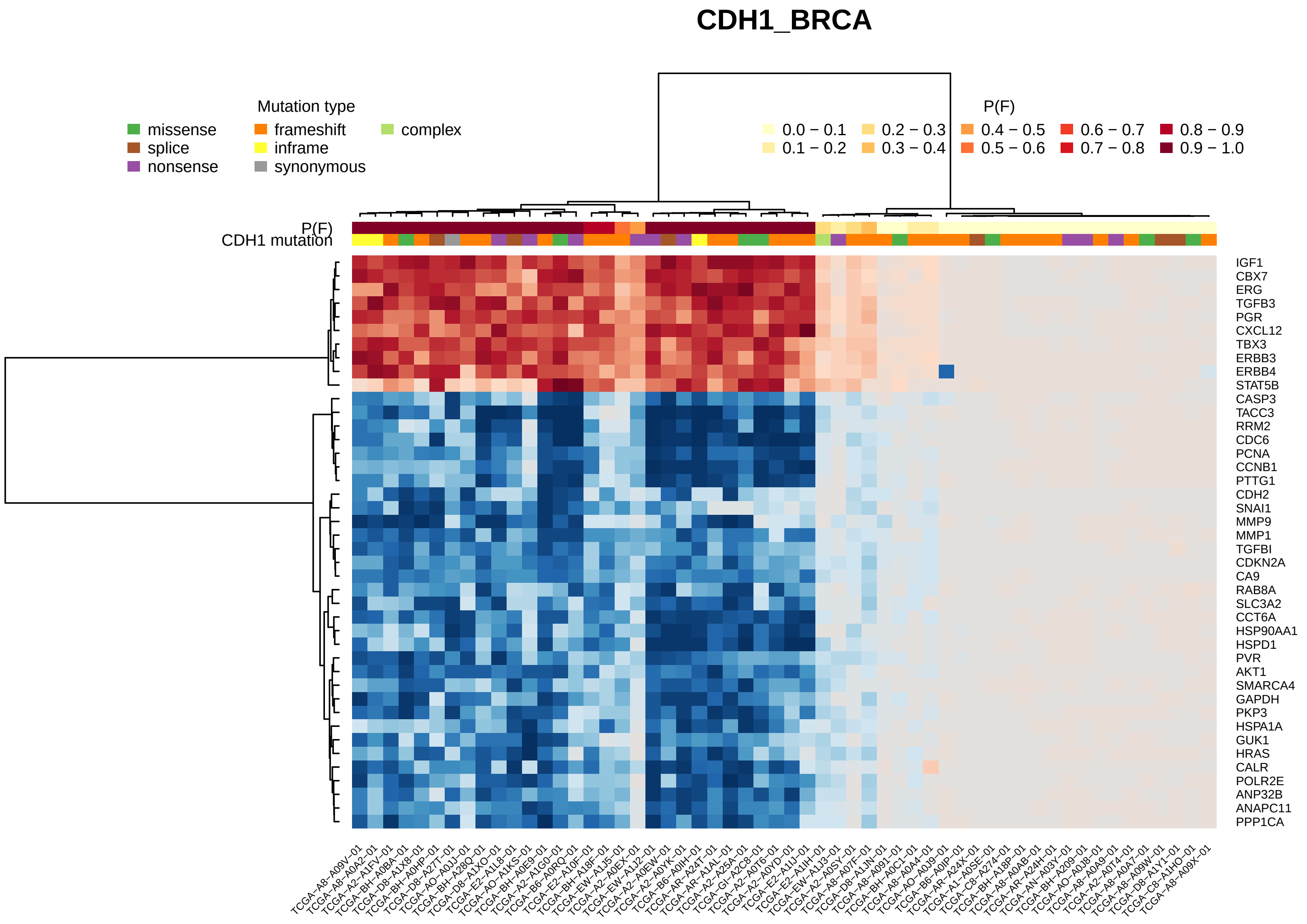
CDH1_BRCA
Mutation type
missense
splice
nonsense
frameshift
inframe
synonymous
complex
P(F)
0.0 − 0.1
0.1 − 0.2
0.2 − 0.3
0.3 − 0.4
0.4 − 0.5
0.5 − 0.6
0.6 − 0.7
0.7 − 0.8
0.8 − 0.9
0.9 − 1.0
P(F)
CDH1 mutation
IGF1
CBX7
ERG
TGFB3
PGR
CXCL12
TBX3
ERBB3
ERBB4
STAT5B
CASP3
TACC3
RRM2
CDC6
PCNA
CCNB1
PTTG1
CDH2
SNAI1
MMP9
MMP1
TGFBI
CDKN2A
CA9
RAB8A
SLC3A2
CCT6A
HSP90AA1
HSPD1
PVR
AKT1
SMARCA4
GAPDH
PKP3
HSPA1A
GUK1
HRAS
CALR
POLR2E
ANP32B
ANAPC11
PPP1CA
TCGA−A8−A09V−01
TCGA−A8−A0A2−01
TCGA−A2−A1FV−01
TCGA−BH−A0BA−01
TCGA−D8−A1X8−01
TCGA−BH−A0HP−01
TCGA−D8−A27T−01
TCGA−AO−A0JJ−01
TCGA−BH−A28Q−01
TCGA−D8−A1XO−01
TCGA−E2−A1L8−01
TCGA−AO−A1KS−01
TCGA−BH−A0E9−01
TCGA−A2−A1G0−01
TCGA−B6−A0RQ−01
TCGA−E2−A10F−01
TCGA−BH−A18F−01
TCGA−EW−A1J5−01
TCGA−A2−A0EX−01
TCGA−EW−A1J2−01
TCGA−A2−A0EW−01
TCGA−A2−A0YK−01
TCGA−B6−A0IH−01
TCGA−AR−A24T−01
TCGA−AR−A1AL−01
TCGA−A2−A25A−01
TCGA−GI−A2C8−01
TCGA−A2−A0T6−01
TCGA−A2−A0YD−01
TCGA−E2−A1IJ−01
TCGA−E2−A1IH−01
TCGA−EW−A1J3−01
TCGA−A2−A0SY−01
TCGA−A8−A07F−01
TCGA−D8−A1JN−01
TCGA−A8−A091−01
TCGA−BH−A0C1−01
TCGA−A8−A0A4−01
TCGA−AO−A0J9−01
TCGA−B6−A0IP−01
TCGA−AR−A24X−01
TCGA−A1−A0SE−01
TCGA−C8−A274−01
TCGA−BH−A18P−01
TCGA−A8−A0AB−01
TCGA−AR−A24H−01
TCGA−AN−A03Y−01
TCGA−BH−A209−01
TCGA−AO−A0J8−01
TCGA−A8−A0A9−01
TCGA−A2−A0T4−01
TCGA−A8−A0A7−01
TCGA−A8−A09W−01
TCGA−D8−A1Y1−01
TCGA−C8−A1HO−01
TCGA−A8−A09X−01

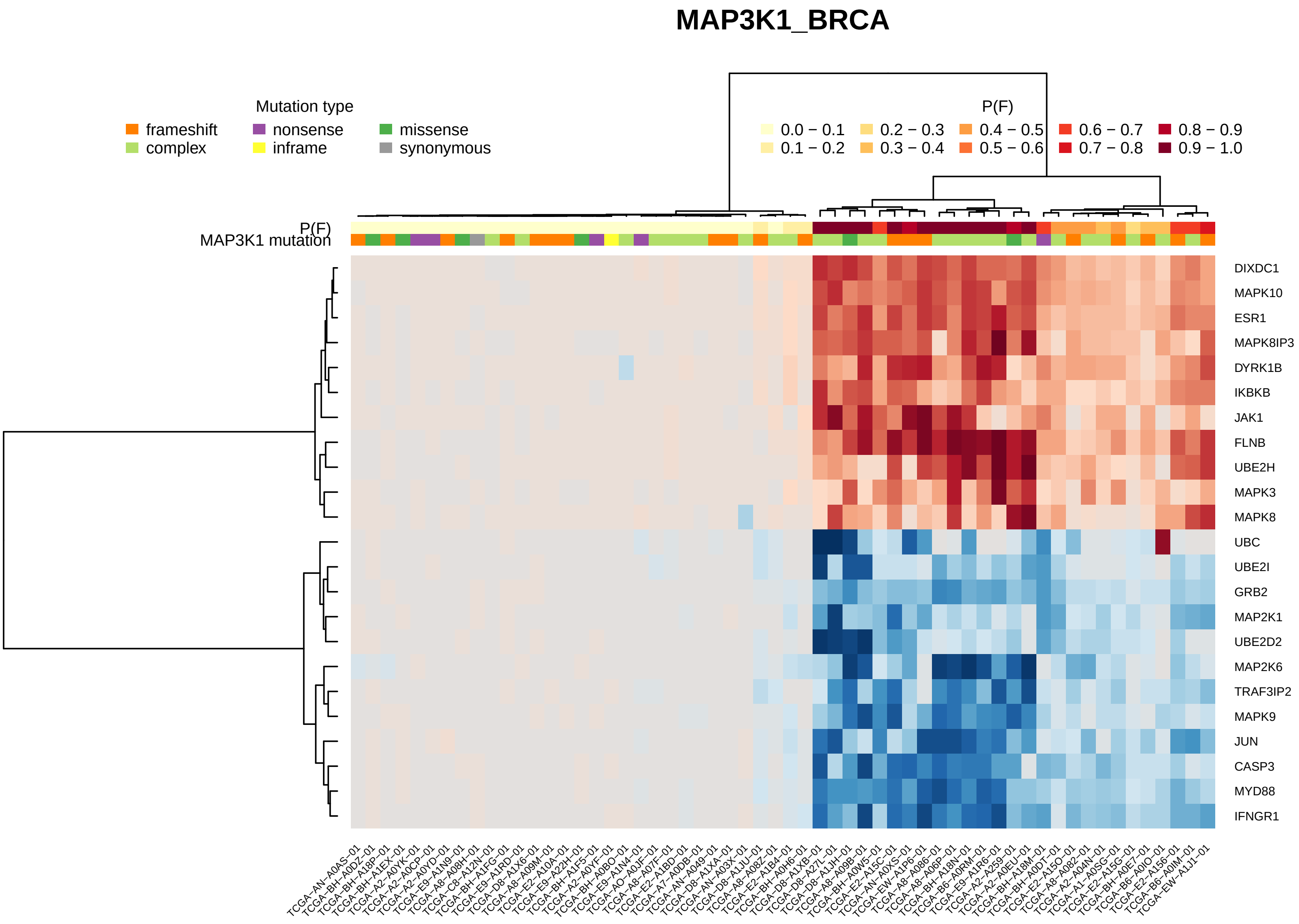
MAP3K1_BRCA
Mutation type
frameshift
complex
nonsense
inframe
missense
synonymous
P(F)
0.0 − 0.1
0.1 − 0.2
0.2 − 0.3
0.3 − 0.4
0.4 − 0.5
0.5 − 0.6
0.6 − 0.7
0.7 − 0.8
0.8 − 0.9
0.9 − 1.0
P(F)
MAP3K1 mutation
DIXDC1
MAPK10
ESR1
MAPK8IP3
DYRK1B
IKBKB
JAK1
FLNB
UBE2H
MAPK3
MAPK8
UBC
UBE2I
GRB2
MAP2K1
UBE2D2
MAP2K6
TRAF3IP2
MAPK9
JUN
CASP3
MYD88
IFNGR1

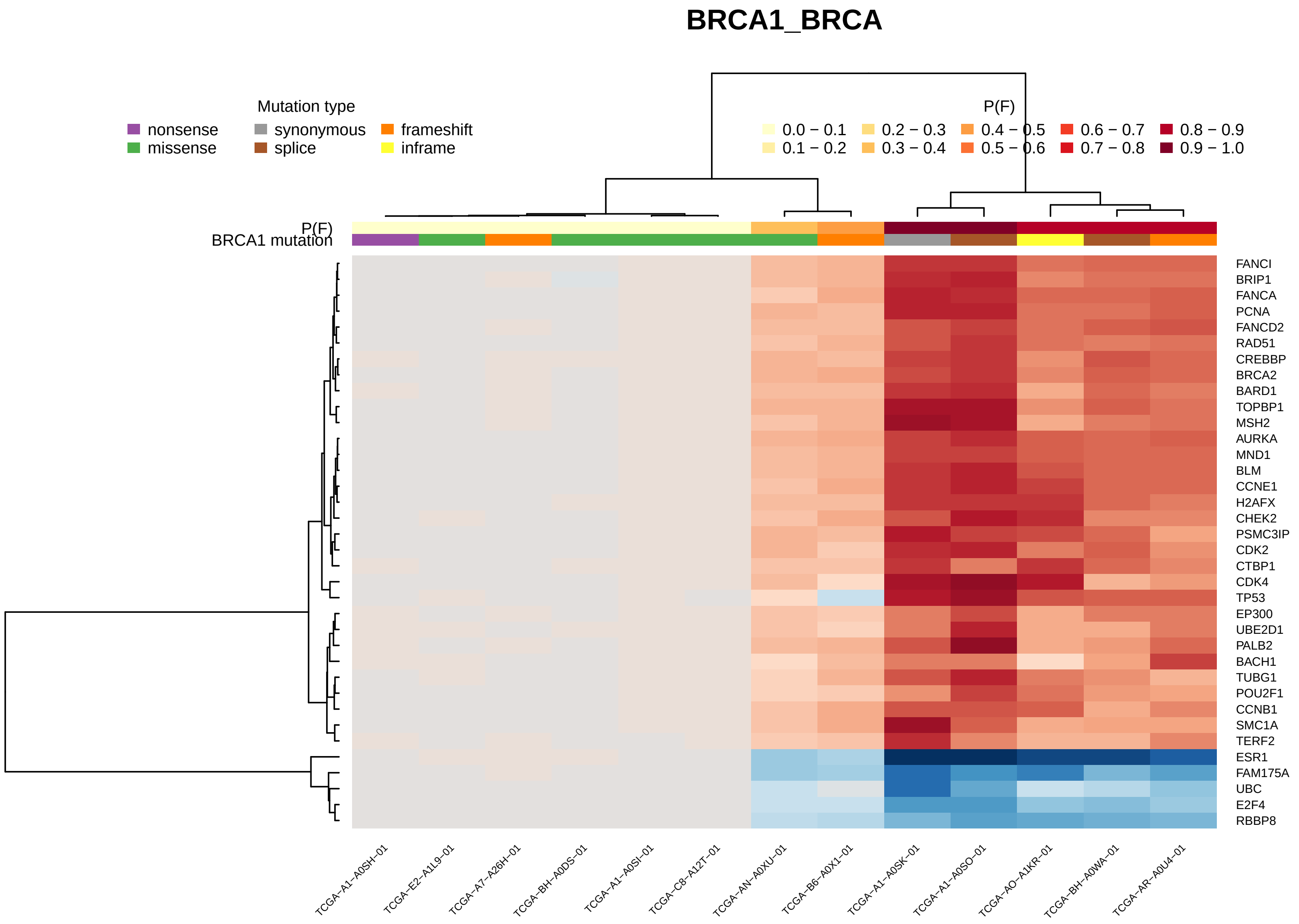

BRCA1_BRCA
Mutation type
nonsense
missense
synonymous
splice
frameshift
inframe
P(F)
0.0 − 0.1
0.1 − 0.2
0.2 − 0.3
0.3 − 0.4
0.4 − 0.5
0.5 − 0.6
0.6 − 0.7
0.7 − 0.8
0.8 − 0.9
0.9 − 1.0
P(F)
BRCA1 mutation
FANCI
BRIP1
FANCA
PCNA
FANCD2
RAD51
CREBBP
BRCA2
BARD1
TOPBP1
MSH2
AURKA
MND1
BLM
CCNE1
H2AFX
CHEK2
PSMC3IP
CDK2
CTBP1
CDK4
TP53
EP300
UBE2D1
PALB2
BACH1
TUBG1
POU2F1
CCNB1
SMC1A
TERF2
ESR1
FAM175A
UBC
E2F4
RBBP8
TCGA−A1−A0SH−01
TCGA−E2−A1L9−01
TCGA−A7−A26H−01
TCGA−BH−A0DS−01
TCGA−A1−A0SI−01
TCGA−C8−A12T−01
TCGA−AN−A0XU−01
TCGA−B6−A0X1−01
TCGA−A1−A0SK−01
TCGA−A1−A0SO−01
TCGA−AO−A1KR−01
TCGA−BH−A0WA−01
TCGA−AR−A0U4−01

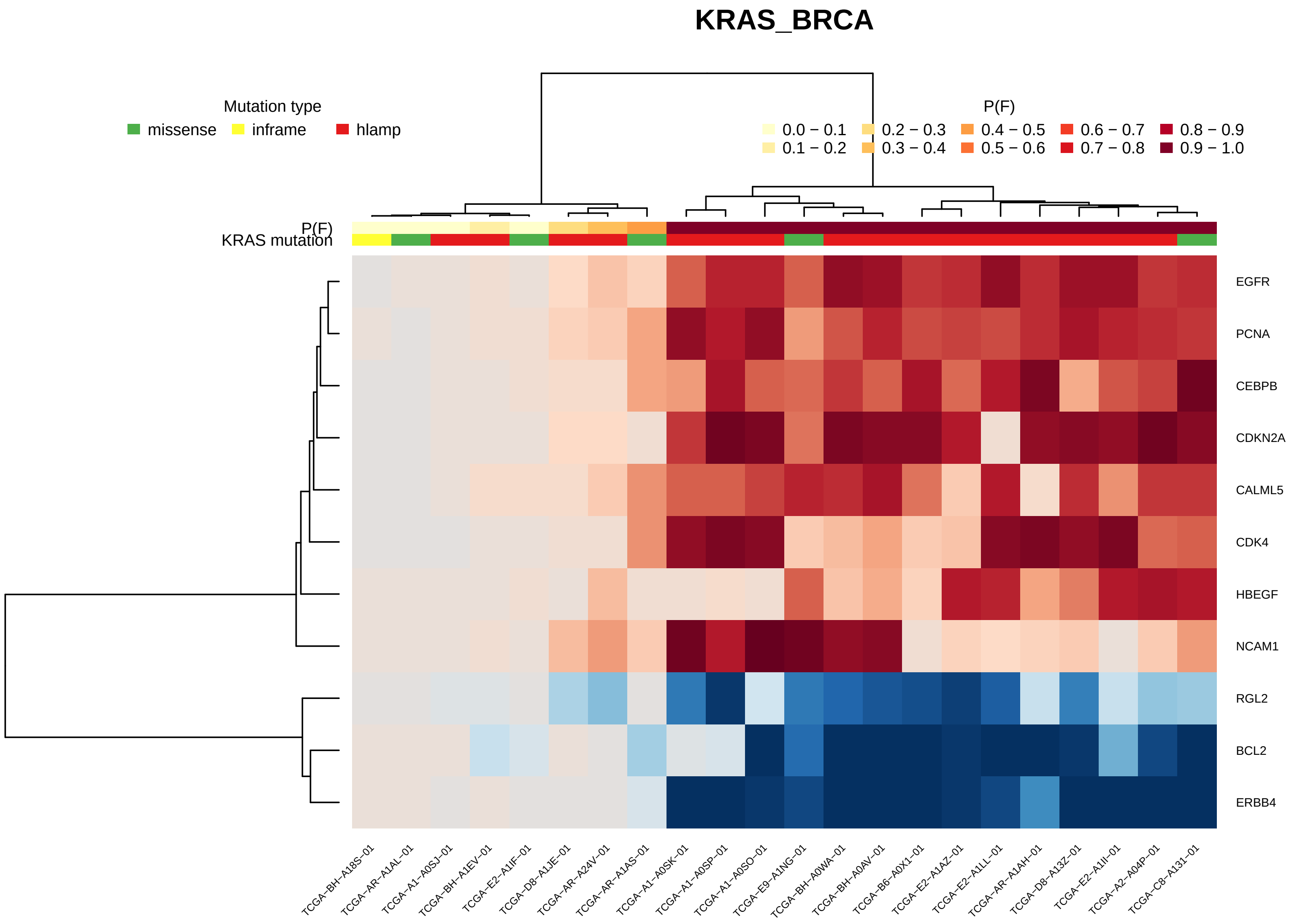
KRAS_BRCA
Mutation type
missense
inframe
hlamp
P(F)
0.0 − 0.1
0.1 − 0.2
0.2 − 0.3
0.3 − 0.4
0.4 − 0.5
0.5 − 0.6
0.6 − 0.7
0.7 − 0.8
0.8 − 0.9
0.9 − 1.0
P(F)
KRAS mutation
EGFR
PCNA
CEBPB
CDKN2A
CALML5
CDK4
HBEGF
NCAM1
RGL2
BCL2
ERBB4
TCGA−BH−A18S−01
TCGA−AR−A1AL−01
TCGA−A1−A0SJ−01
TCGA−BH−A1EV−01
TCGA−E2−A1IF−01
TCGA−D8−A1JE−01
TCGA−AR−A24V−01
TCGA−AR−A1AS−01
TCGA−A1−A0SK−01
TCGA−A1−A0SP−01
TCGA−A1−A0SO−01
TCGA−E9−A1NG−01
TCGA−BH−A0WA−01
TCGA−BH−A0AV−01
TCGA−B6−A0X1−01
TCGA−E2−A1AZ−01
TCGA−E2−A1LL−01
TCGA−AR−A1AH−01
TCGA−D8−A13Z−01
TCGA−E2−A1II−01
TCGA−A2−A04P−01
TCGA−C8−A131−01

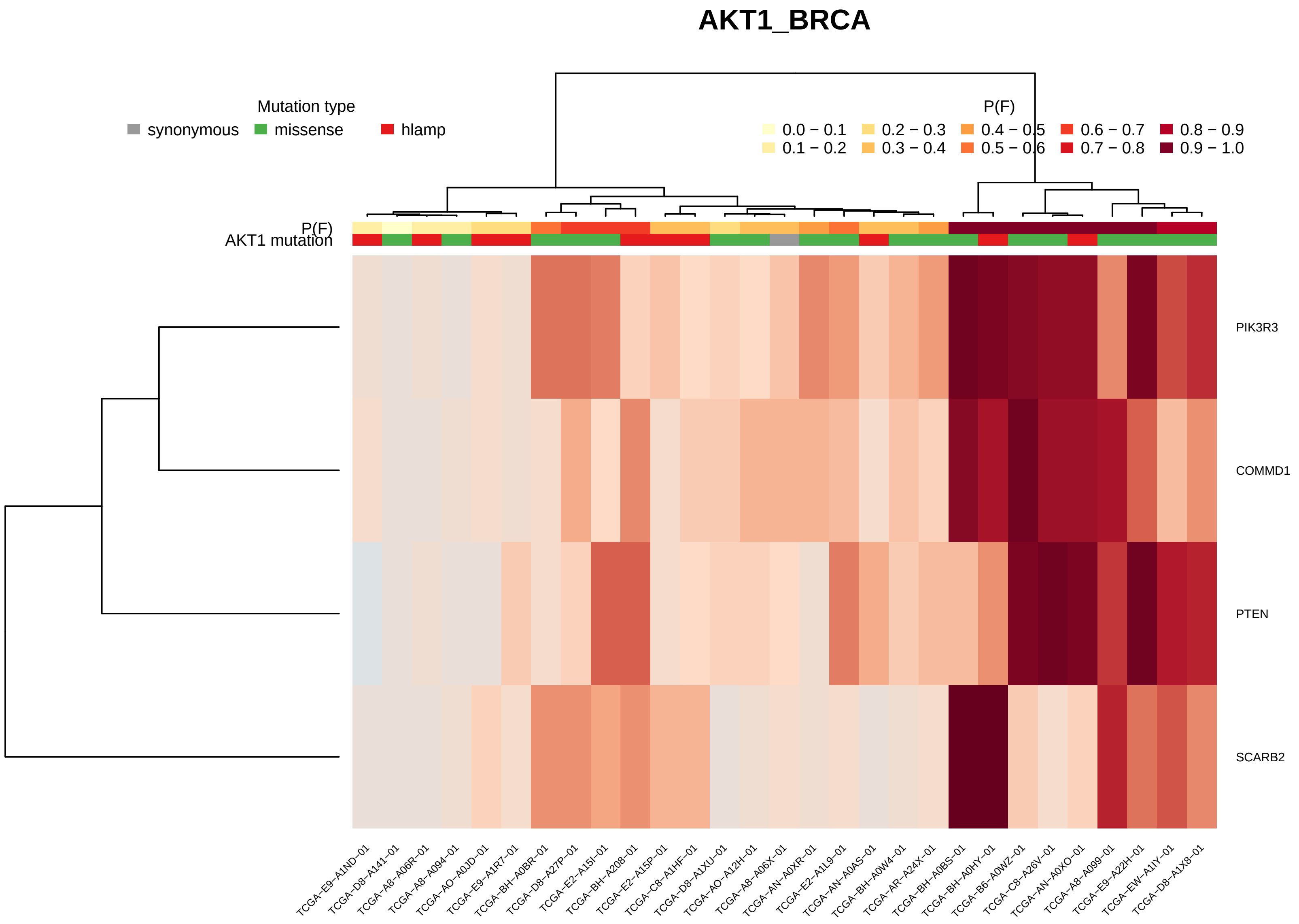

AKT1_BRCA
Mutation type
synonymous
missense
hlamp
P(F)
0.0 − 0.1
0.1 − 0.2
0.2 − 0.3
0.3 − 0.4
0.4 − 0.5
0.5 − 0.6
0.6 − 0.7
0.7 − 0.8
0.8 − 0.9
0.9 − 1.0
P(F)
AKT1 mutation
PIK3R3
COMMD1
PTEN
SCARB2
TCGA−E9−A1ND−01
TCGA−D8−A141−01
TCGA−A8−A06R−01
TCGA−A8−A094−01
TCGA−AO−A0JD−01
TCGA−E9−A1R7−01
TCGA−BH−A0BR−01
TCGA−D8−A27P−01
TCGA−E2−A15I−01
TCGA−BH−A208−01
TCGA−E2−A15P−01
TCGA−C8−A1HF−01
TCGA−D8−A1XU−01
TCGA−AO−A12H−01
TCGA−A8−A06X−01
TCGA−AN−A0XR−01
TCGA−E2−A1L9−01
TCGA−AN−A0AS−01
TCGA−BH−A0W4−01
TCGA−AR−A24X−01
TCGA−BH−A0BS−01
TCGA−BH−A0HY−01
TCGA−B6−A0WZ−01
TCGA−C8−A26V−01
TCGA−AN−A0XO−01
TCGA−A8−A099−01
TCGA−E9−A22H−01
TCGA−EW−A1IY−01
TCGA−D8−A1X8−01

# MAP2K4_BRCA

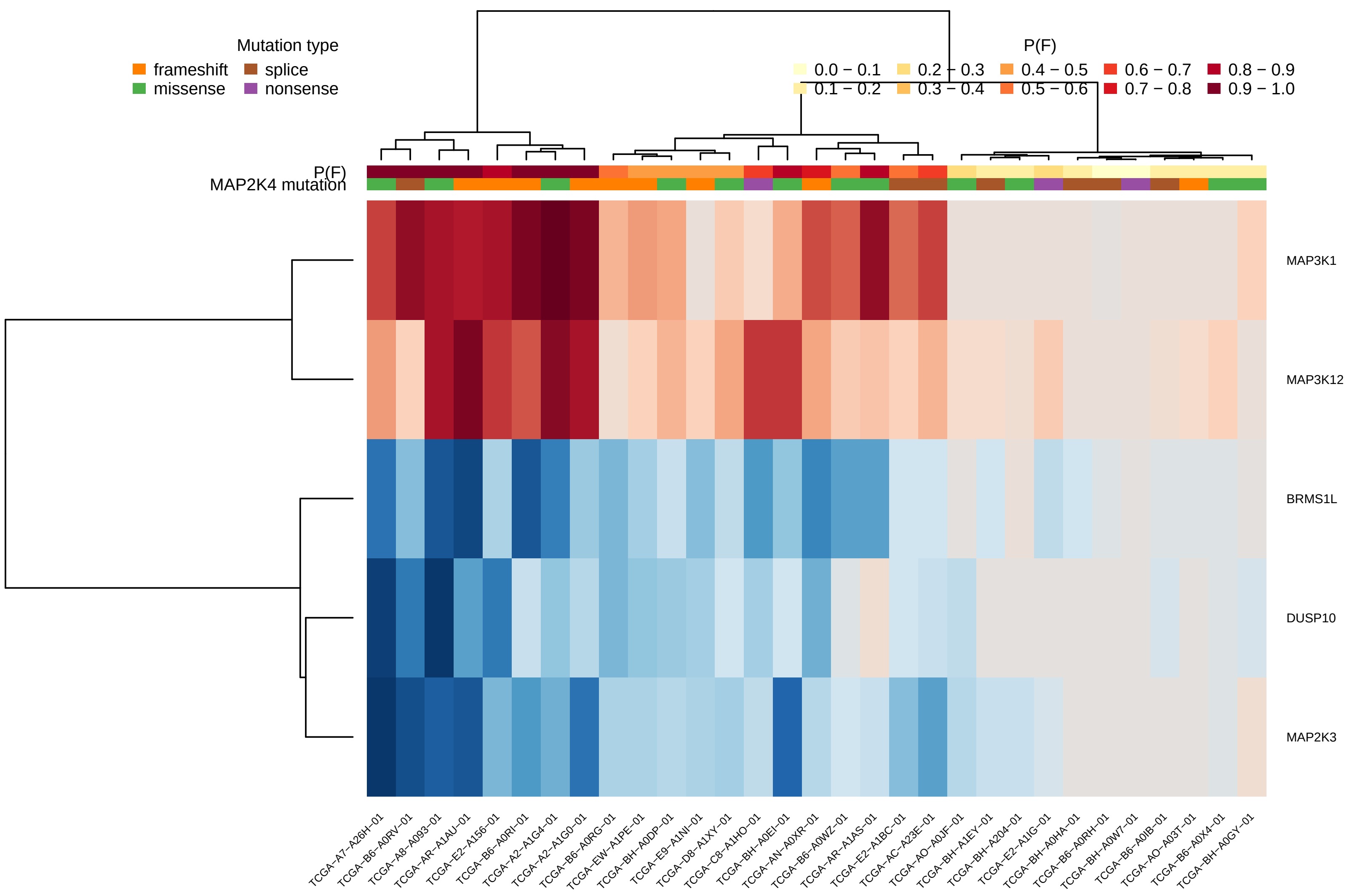

Mutation type
frameshift
splice
missense
nonsense
P(F)
0.0 − 0.1
0.1 − 0.2
0.2 − 0.3
0.3 − 0.4
0.4 − 0.5
0.5 − 0.6
0.6 − 0.7
0.7 − 0.8
0.8 − 0.9
0.9 − 1.0
P(F)
MAP2K4 mutation
MAP3K1
MAP3K12
BRMS1L
DUSP10
MAP2K3
TCGA−A7−A26H−01
TCGA−B6−A0RV−01
TCGA−A8−A093−01
TCGA−AR−A1AU−01
TCGA−E2−A156−01
TCGA−B6−A0RI−01
TCGA−A2−A1G4−01
TCGA−A2−A1G0−01
TCGA−B6−A0RG−01
TCGA−EW−A1PE−01
TCGA−BH−A0DP−01
TCGA−E9−A1NI−01
TCGA−D8−A1XY−01
TCGA−C8−A1HO−01
TCGA−BH−A0EI−01
TCGA−AN−A0XR−01
TCGA−B6−A0WZ−01
TCGA−AR−A1AS−01
TCGA−E2−A1BC−01
TCGA−AC−A23E−01
TCGA−AO−A0JF−01
TCGA−BH−A1EY−01
TCGA−BH−A204−01
TCGA−E2−A1IG−01
TCGA−BH−A0HA−01
TCGA−B6−A0RH−01
TCGA−BH−A0W7−01
TCGA−B6−A0IB−01
TCGA−AO−A03T−01
TCGA−B6−A0X4−01
TCGA−BH−A0GY−01

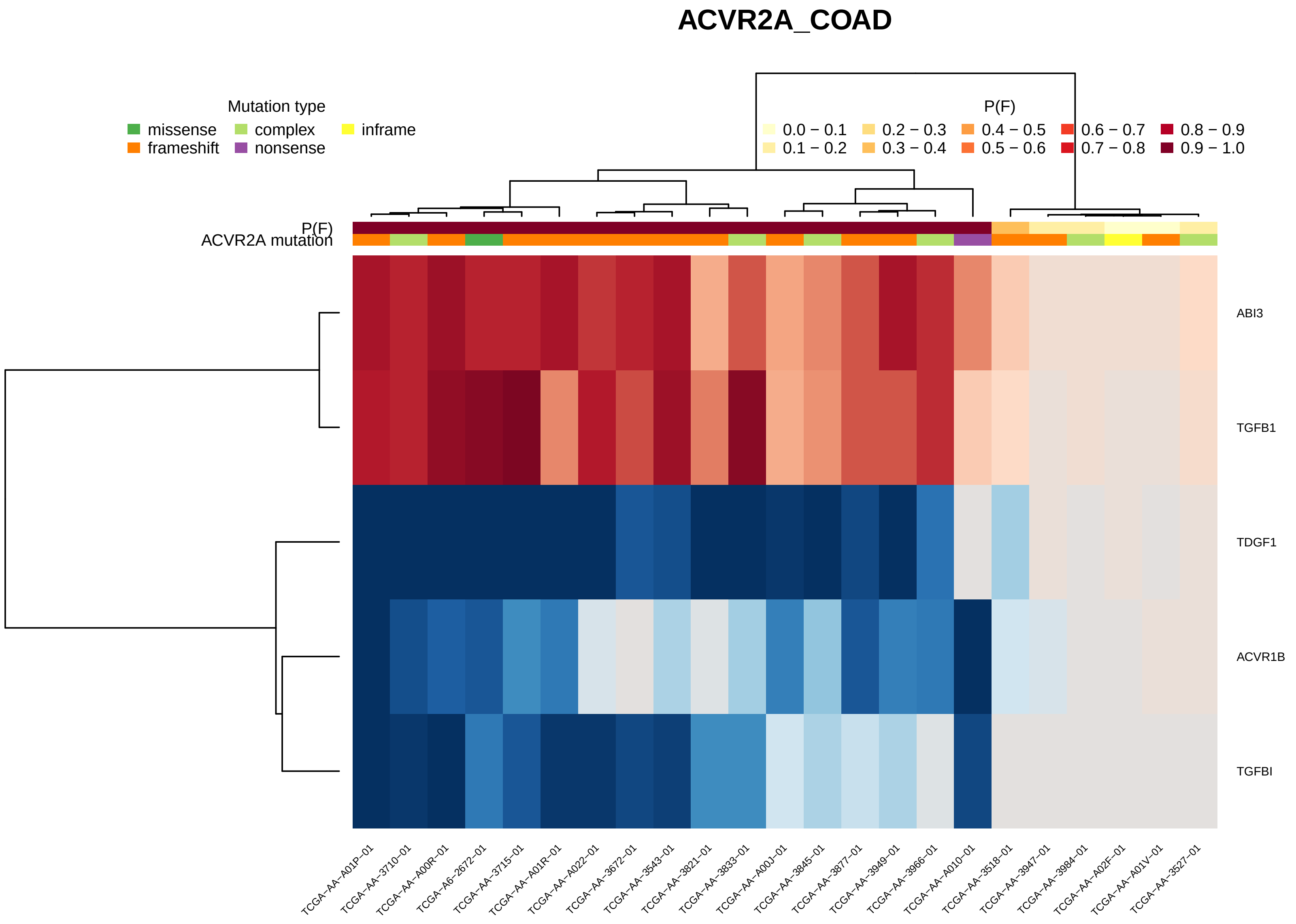

ACVR2A_COAD
Mutation type
missense
complex
inframe
frameshift
nonsense
P(F)
0.0 − 0.1
0.1 − 0.2
0.2 − 0.3
0.3 − 0.4
0.4 − 0.5
0.5 − 0.6
0.6 − 0.7
0.7 − 0.8
0.8 − 0.9
0.9 − 1.0
P(F)
ACVR2A mutation
ABI3
TGFB1
TDGF1
ACVR1B
TGFBI
TCGA−AA−A01P−01
TCGA−AA−3710−01
TCGA−AA−A00R−01
TCGA−A6−2672−01
TCGA−AA−3715−01
TCGA−AA−A01R−01
TCGA−AA−A022−01
TCGA−AA−3672−01
TCGA−AA−3543−01
TCGA−AA−3821−01
TCGA−AA−3833−01
TCGA−AA−A00J−01
TCGA−AA−3845−01
TCGA−AA−3877−01
TCGA−AA−3949−01
TCGA−AA−3966−01
TCGA−AA−A010−01
TCGA−AA−3518−01
TCGA−AA−3947−01
TCGA−AA−3984−01
TCGA−AA−A02F−01
TCGA−AA−A01V−01
TCGA−AA−3527−01

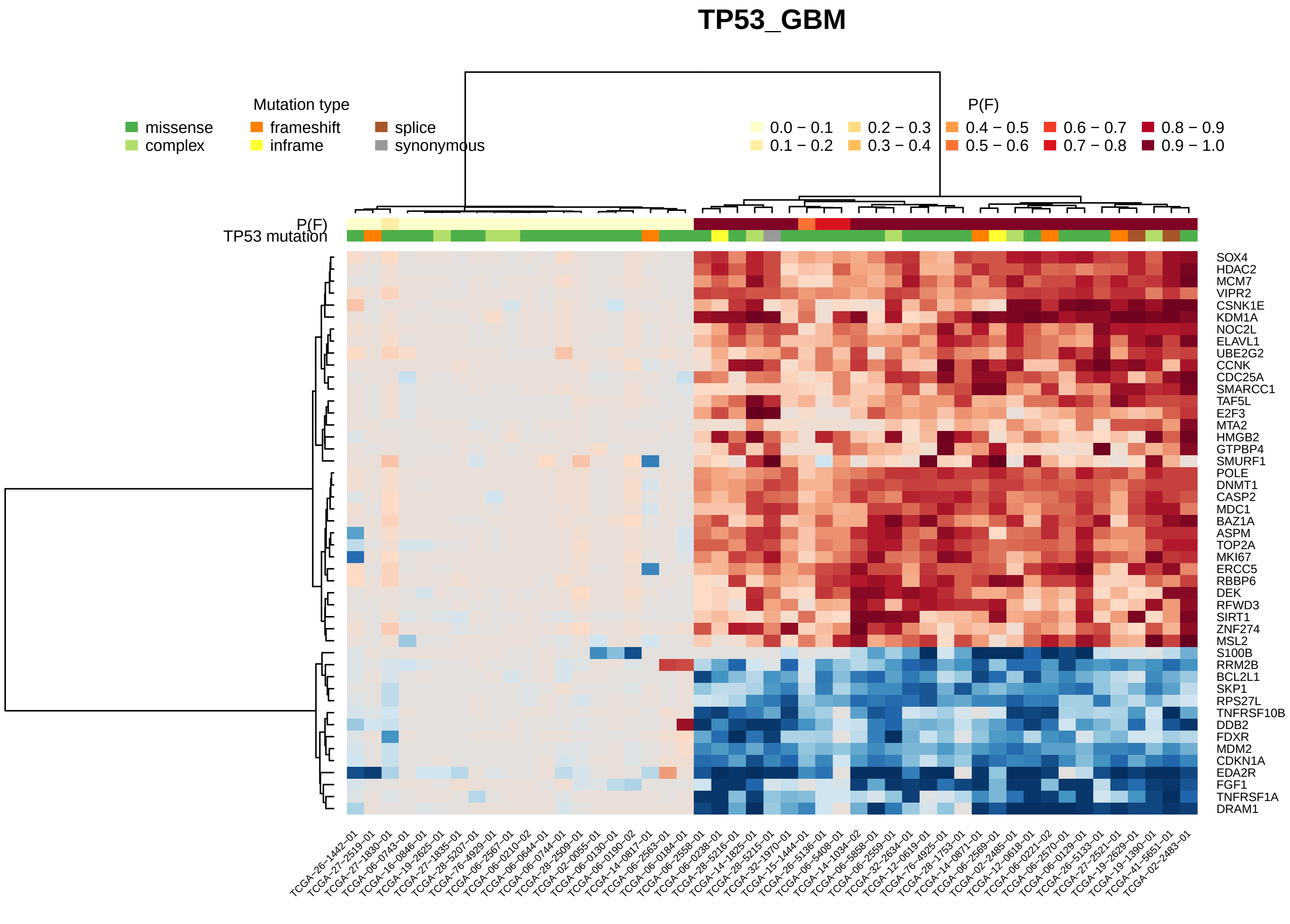

TP53_GBM
Mutation type
missense
complex
frameshift
inframe
splice
synonymous
P(F)
0.0 − 0.1
0.1 − 0.2
0.2 − 0.3
0.3 − 0.4
0.4 − 0.5
0.5 − 0.6
0.6 − 0.7
0.7 − 0.8
0.8 − 0.9
0.9 − 1.0
P(F)
TP53 mutation
SOX4
HDAC2
MCM7
VIPR2
CSNK1E
KDM1A
NOC2L
ELAVL1
UBE2G2
CCNK
CDC25A
SMARCC1
TAF5L
E2F3
MTA2
HMGB2
GTPBP4
SMURF1
POLE
DNMT1
CASP2
MDC1
BAZ1A
ASPM
TOP2A
MKI67
ERCC5
RBBP6
DEK
RFWD3
SIRT1
ZNF274
MSL2
S100B
RRM2B
BCL2L1
SKP1
RPS27L
TNFRSF10B
DDB2
FDXR
MDM2
CDKN1A
EDA2R
FGF1
TNFRSF1A
DRAM1
TCGA−26−1442−01
TCGA−27−2519−01
TCGA−27−1830−01
TCGA−06−0743−01
TCGA−16−0846−01
TCGA−19−2625−01
TCGA−27−1835−01
TCGA−28−5207−01
TCGA−76−4929−01
TCGA−06−2567−01
TCGA−06−0210−02
TCGA−06−0644−01
TCGA−06−0744−01
TCGA−28−2509−01
TCGA−02−0055−01
TCGA−06−0130−01
TCGA−06−0190−02
TCGA−14−0817−01
TCGA−06−2563−01
TCGA−06−0184−01
TCGA−06−2558−01
TCGA−06−0238−01
TCGA−28−5216−01
TCGA−14−1825−01
TCGA−28−5215−01
TCGA−32−1970−01
TCGA−15−1444−01
TCGA−26−5136−01
TCGA−06−5408−01
TCGA−14−1034−02
TCGA−06−5858−01
TCGA−06−2559−01
TCGA−32−2634−01
TCGA−12−0619−01
TCGA−76−4925−01
TCGA−28−1753−01
TCGA−14−0871−01
TCGA−06−2569−01
TCGA−02−2485−01
TCGA−12−0618−01
TCGA−06−0221−02
TCGA−06−2570−01
TCGA−06−0129−01
TCGA−26−5133−01
TCGA−27−2521−01
TCGA−19−2629−01
TCGA−19−1390−01
TCGA−41−5651−01
TCGA−02−2483−01

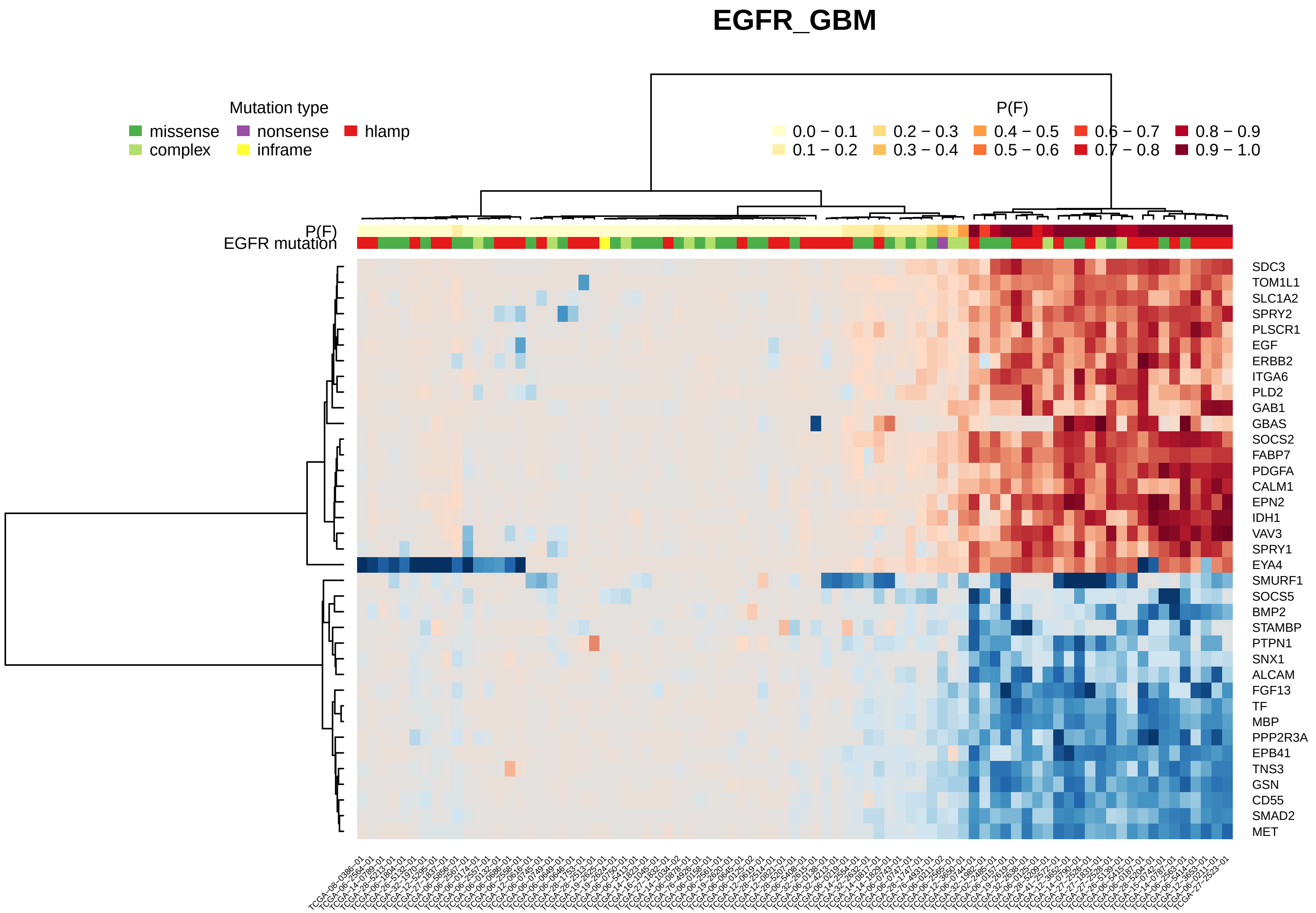
EGFR_GBM
Mutation type
missense
nonsense
hlamp
complex
inframe
P(F)
0.0 − 0.1
0.1 − 0.2
0.2 − 0.3
0.3 − 0.4
0.4 − 0.5
0.5 − 0.6
0.6 − 0.7
0.7 − 0.8
0.8 − 0.9
0.9 − 1.0
P(F)
EGFR mutation
SDC3
TOM1L1
SLC1A2
SPRY2
PLSCR1
EGF
ERBB2
ITGA6
PLD2
GAB1
GBAS
SOCS2
FABP7
PDGFA
CALM1
EPN2
IDH1
VAV3
SPRY1
EYA4
SMURF1
SOCS5
BMP2
STAMBP
PTPN1
SNX1
ALCAM
FGF13
TF
MBP
PPP2R3A
EPB41
TNS3
GSN
CD55
SMAD2
MET

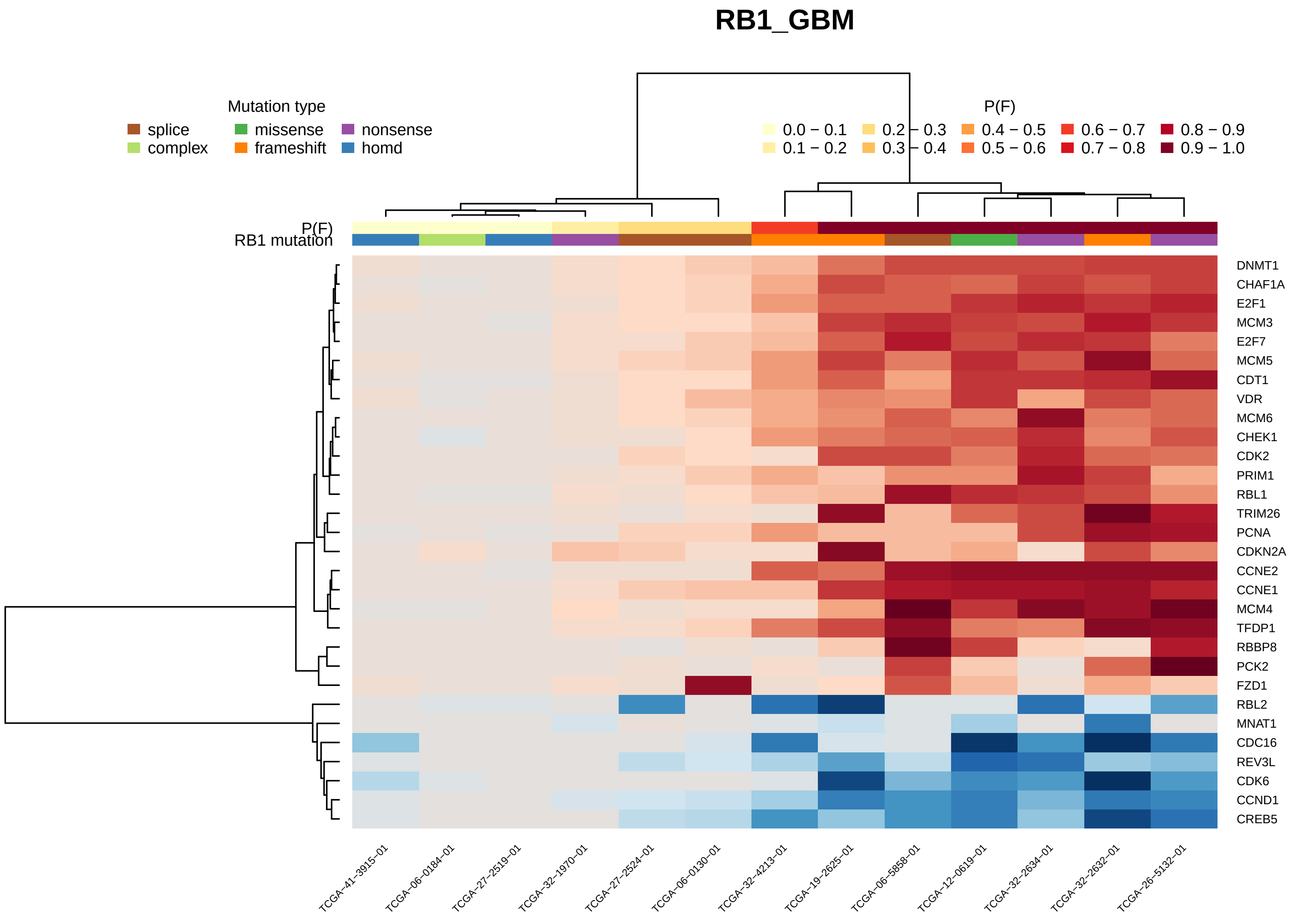

RB1_GBM
Mutation type
splice
complex
missense
frameshift
nonsense
homd
P(F)
0.0 − 0.1
0.1 − 0.2
0.2 − 0.3
0.3 − 0.4
0.4 − 0.5
0.5 − 0.6
0.6 − 0.7
0.7 − 0.8
0.8 − 0.9
0.9 − 1.0
P(F)
RB1 mutation
DNMT1
CHAF1A
E2F1
MCM3
E2F7
MCM5
CDT1
VDR
MCM6
CHEK1
CDK2
PRIM1
RBL1
TRIM26
PCNA
CDKN2A
CCNE2
CCNE1
MCM4
TFDP1
RBBP8
PCK2
FZD1
RBL2
MNAT1
CDC16
REV3L
CDK6
CCND1
CREB5
TCGA−41−3915−01
TCGA−06−0184−01
TCGA−27−2519−01
TCGA−32−1970−01
TCGA−27−2524−01
TCGA−06−0130−01
TCGA−32−4213−01
TCGA−19−2625−01
TCGA−06−5858−01
TCGA−12−0619−01
TCGA−32−2634−01
TCGA−32−2632−01
TCGA−26−5132−01

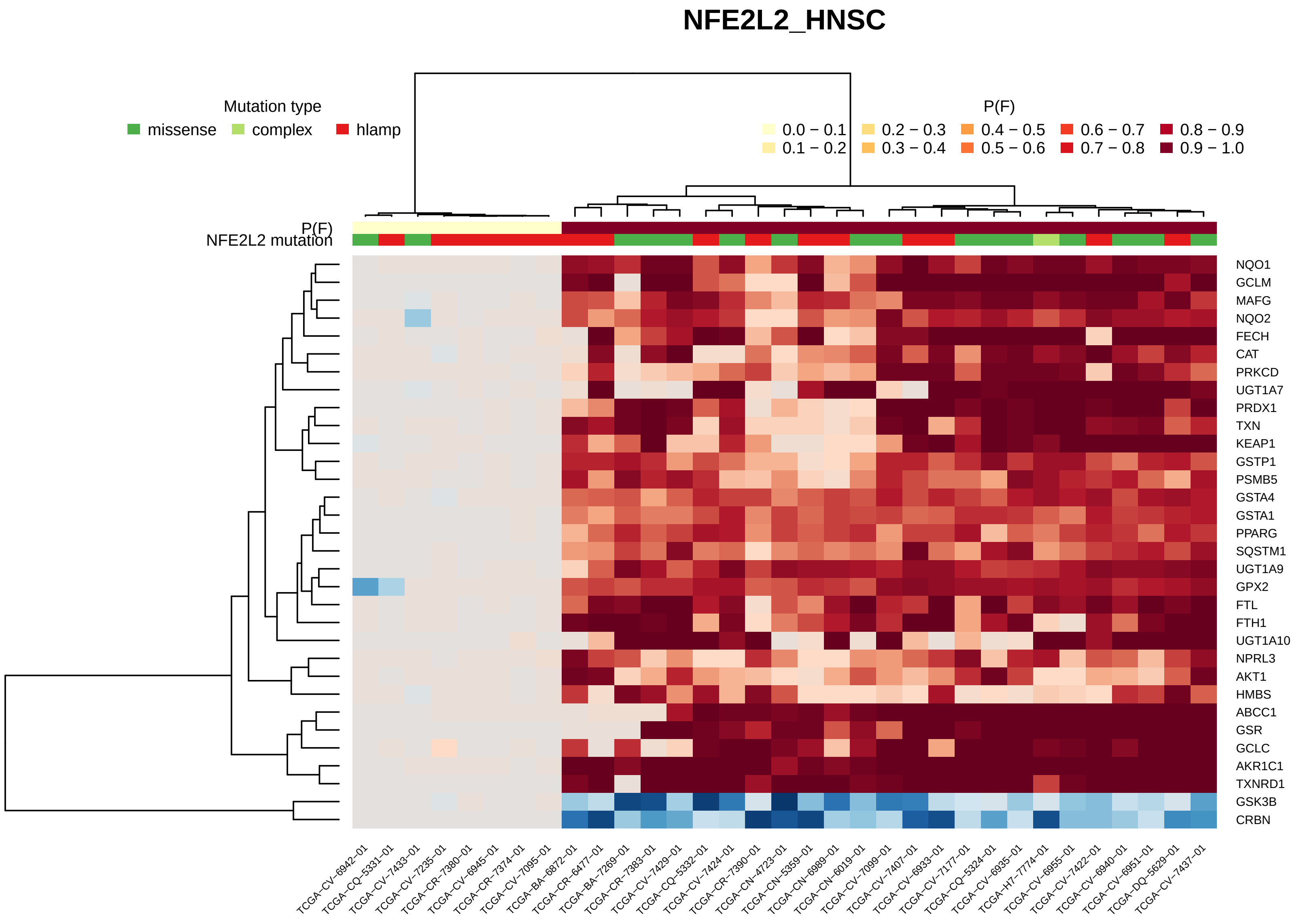
NFE2L2_HNSC
Mutation type
missense
complex
hlamp
P(F)
0.0 − 0.1
0.1 − 0.2
0.2 − 0.3
0.3 − 0.4
0.4 − 0.5
0.5 − 0.6
0.6 − 0.7
0.7 − 0.8
0.8 − 0.9
0.9 − 1.0
P(F)
NFE2L2 mutation
NQO1
GCLM
MAFG
NQO2
FECH
CAT
PRKCD
UGT1A7
PRDX1
TXN
KEAP1
GSTP1
PSMB5
GSTA4
GSTA1
PPARG
SQSTM1
UGT1A9
GPX2
FTL
FTH1
UGT1A10
NPRL3
AKT1
HMBS
ABCC1
GSR
GCLC
AKR1C1
TXNRD1
GSK3B
CRBN
TCGA−CV−6942−01
TCGA−CQ−5331−01
TCGA−CV−7433−01
TCGA−CV−7235−01
TCGA−CR−7380−01
TCGA−CV−6945−01
TCGA−CR−7374−01
TCGA−CV−7095−01
TCGA−BA−6872−01
TCGA−CR−6477−01
TCGA−BA−7269−01
TCGA−CR−7383−01
TCGA−CV−7429−01
TCGA−CQ−5332−01
TCGA−CV−7424−01
TCGA−CR−7390−01
TCGA−CN−4723−01
TCGA−CN−5359−01
TCGA−CN−6989−01
TCGA−CN−6019−01
TCGA−CV−7099−01
TCGA−CV−7407−01
TCGA−CV−6933−01
TCGA−CV−7177−01
TCGA−CQ−5324−01
TCGA−CV−6935−01
TCGA−H7−7774−01
TCGA−CV−6955−01
TCGA−CV−7422−01
TCGA−CV−6940−01
TCGA−CV−6951−01
TCGA−DQ−5629−01
TCGA−CV−7437−01

# NOTCH1_HNSC

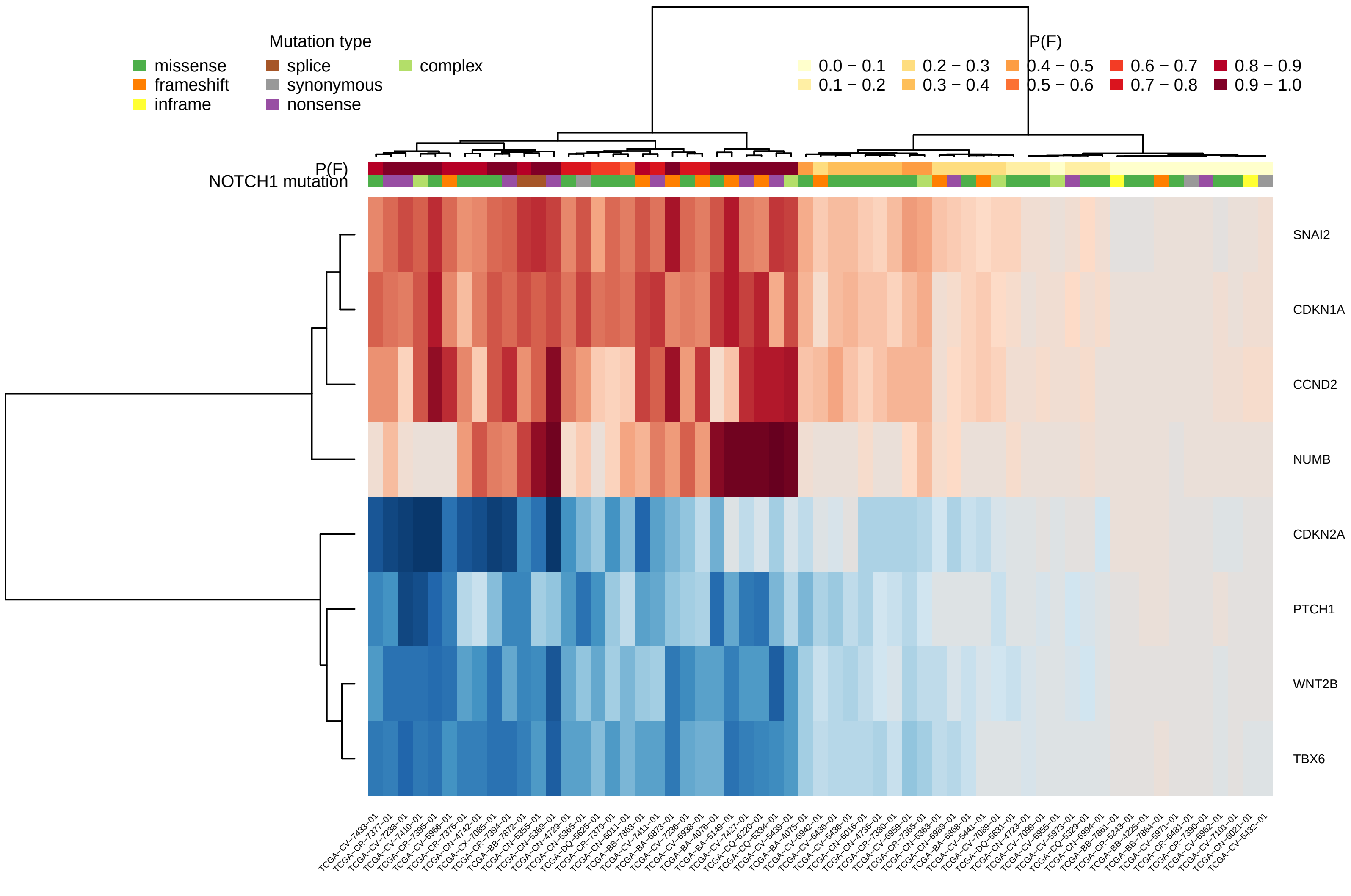
Mutation type
missense
frameshift
inframe
splice
synonymous
nonsense
complex
P(F)
0.0 − 0.1
0.1 − 0.2
0.2 − 0.3
0.3 − 0.4
0.4 − 0.5
0.5 − 0.6
0.6 − 0.7
0.7 − 0.8
0.8 − 0.9
0.9 − 1.0
P(F)
NOTCH1 mutation
SNAI2
CDKN1A
CCND2
NUMB
CDKN2A
PTCH1
WNT2B
TBX6
TCGA−CV−7433−01
TCGA−CR−7377−01
TCGA−CV−7238−01
TCGA−CV−7410−01
TCGA−CR−7395−01
TCGA−CV−5966−01
TCGA−CR−7376−01
TCGA−CN−4742−01
TCGA−CX−7085−01
TCGA−CR−7394−01
TCGA−BB−7872−01
TCGA−CN−5355−01
TCGA−CN−5369−01
TCGA−CN−4729−01
TCGA−CN−5365−01
TCGA−DQ−5625−01
TCGA−CR−7379−01
TCGA−CN−6011−01
TCGA−BB−7863−01
TCGA−CV−7411−01
TCGA−BA−6873−01
TCGA−CV−7236−01
TCGA−CV−6938−01
TCGA−BA−4076−01
TCGA−BA−5149−01
TCGA−CV−7427−01
TCGA−CQ−6220−01
TCGA−CQ−5334−01
TCGA−CV−5439−01
TCGA−BA−4075−01
TCGA−CV−6942−01
TCGA−CV−6436−01
TCGA−CV−5436−01
TCGA−CN−6016−01
TCGA−CN−4736−01
TCGA−CR−7380−01
TCGA−CV−6959−01
TCGA−CR−7365−01
TCGA−CN−5363−01
TCGA−CN−6989−01
TCGA−BA−6868−01
TCGA−CV−5441−01
TCGA−CV−7089−01
TCGA−DQ−5631−01
TCGA−CN−4723−01
TCGA−CV−7099−01
TCGA−CV−6955−01
TCGA−CV−5973−01
TCGA−CQ−5329−01
TCGA−CN−6994−01
TCGA−BB−7861−01
TCGA−CR−5243−01
TCGA−BB−4225−01
TCGA−BB−7864−01
TCGA−CV−5971−01
TCGA−CR−6481−01
TCGA−CR−7390−01
TCGA−CV−6962−01
TCGA−CV−7101−01
TCGA−CN−6021−01
TCGA−CV−5432−01

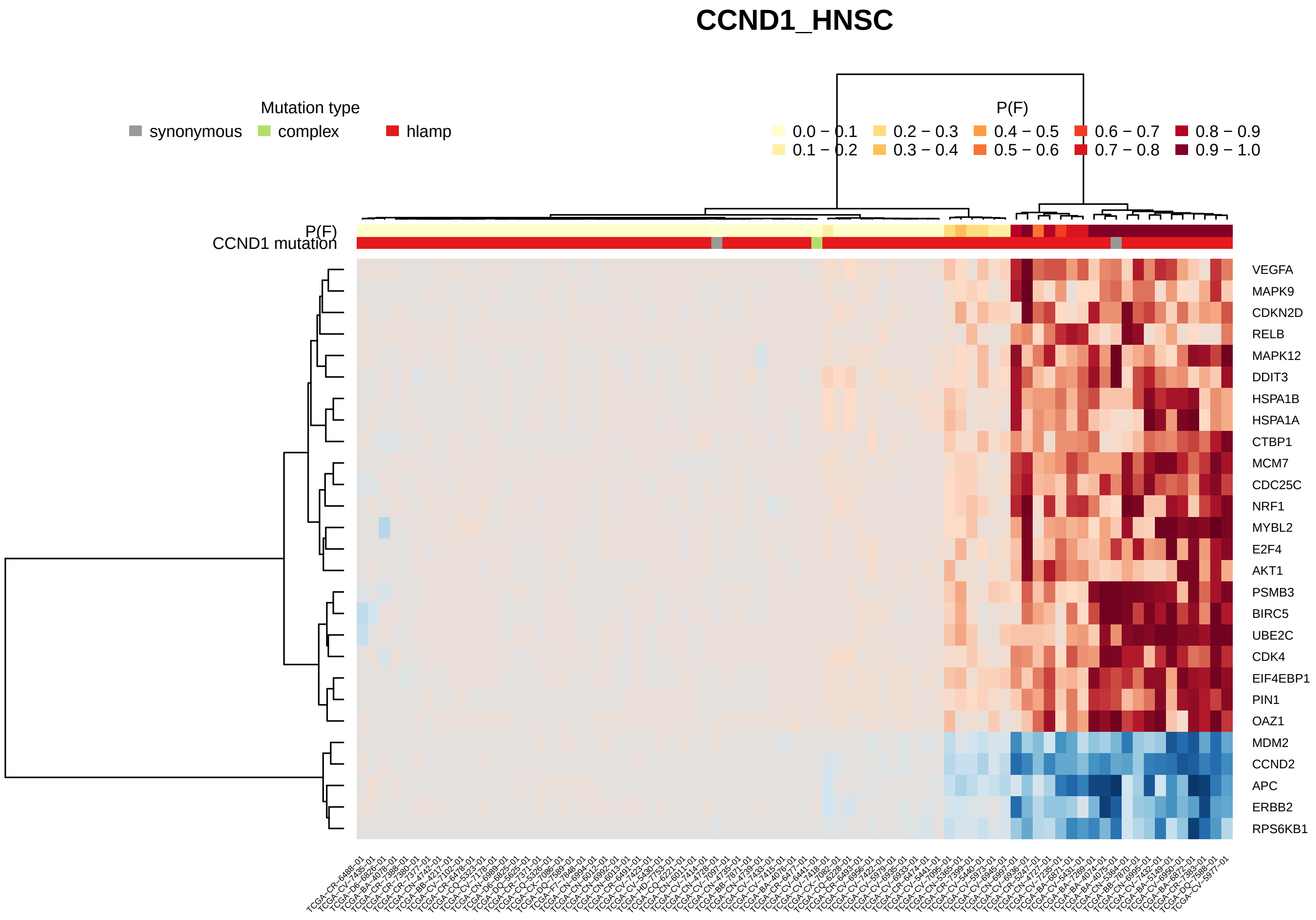

CCND1_HNSC
Mutation type
synonymous
complex
hlamp
P(F)
0.0 − 0.1
0.1 − 0.2
0.2 − 0.3
0.3 − 0.4
0.4 − 0.5
0.5 − 0.6
0.6 − 0.7
0.7 − 0.8
0.8 − 0.9
0.9 − 1.0
P(F)
CCND1 mutation
VEGFA
MAPK9
CDKN2D
RELB
MAPK12
DDIT3
HSPA1B
HSPA1A
CTBP1
MCM7
CDC25C
NRF1
MYBL2
E2F4
AKT1
PSMB3
BIRC5
UBE2C
CDK4
EIF4EBP1
PIN1
OAZ1
MDM2
CCND2
APC
ERBB2
RPS6KB1

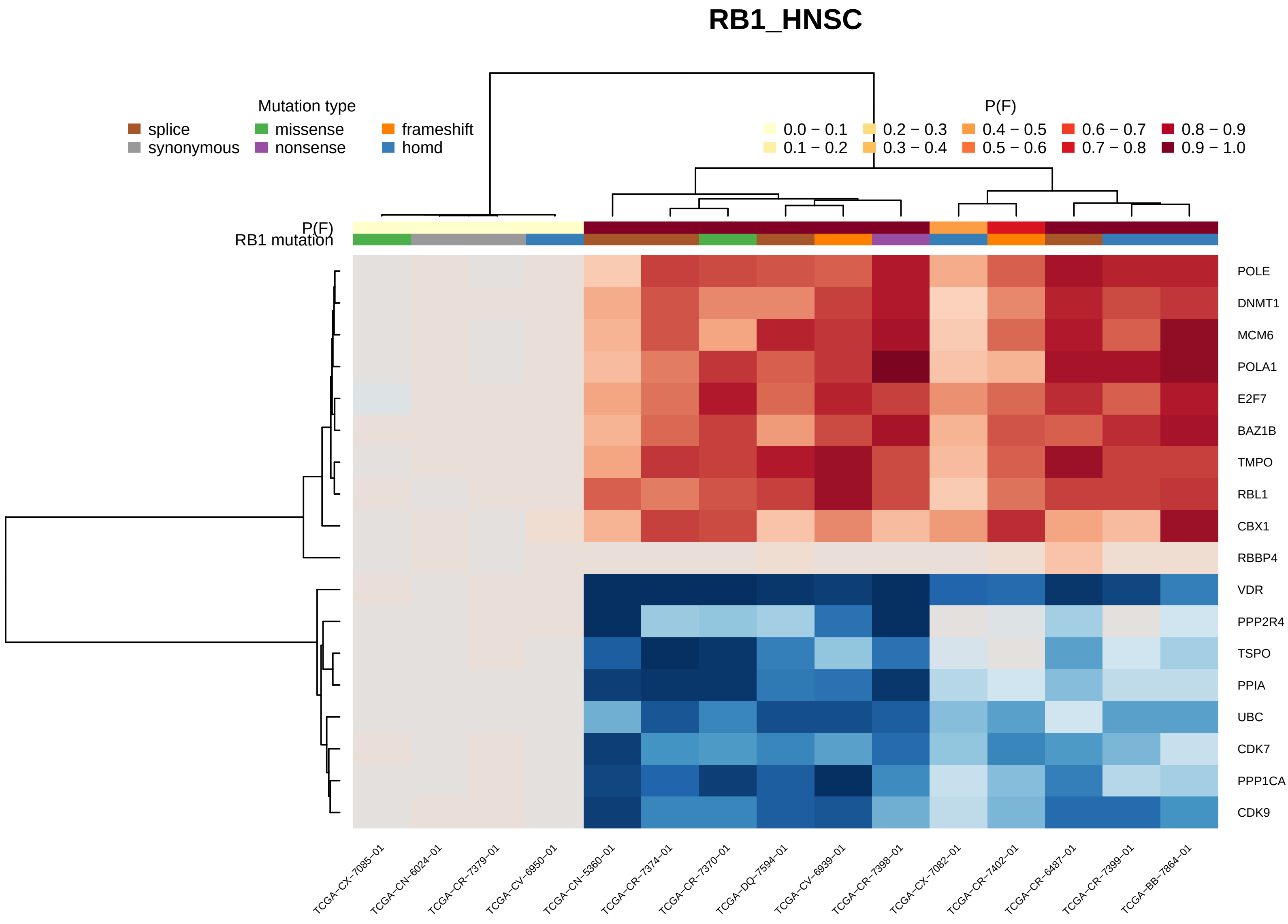

RB1_HNSC
Mutation type
splice
synonymous
missense
nonsense
frameshift
homd
P(F)
0.0 − 0.1
0.1 − 0.2
0.2 − 0.3
0.3 − 0.4
0.4 − 0.5
0.5 − 0.6
0.6 − 0.7
0.7 − 0.8
0.8 − 0.9
0.9 − 1.0
P(F)
RB1 mutation
POLE
DNMT1
MCM6
POLA1
E2F7
BAZ1B
TMPO
RBL1
CBX1
RBBP4
VDR
PPP2R4
TSPO
PPIA
UBC
CDK7
PPP1CA
CDK9
TCGA−CX−7085−01
TCGA−CN−6024−01
TCGA−CR−7379−01
TCGA−CV−6950−01
TCGA−CN−5360−01
TCGA−CR−7374−01
TCGA−CR−7370−01
TCGA−DQ−7594−01
TCGA−CV−6939−01
TCGA−CR−7398−01
TCGA−CX−7082−01
TCGA−CR−7402−01
TCGA−CR−6487−01
TCGA−CR−7399−01
TCGA−BB−7864−01

## KEAP1_HNSC

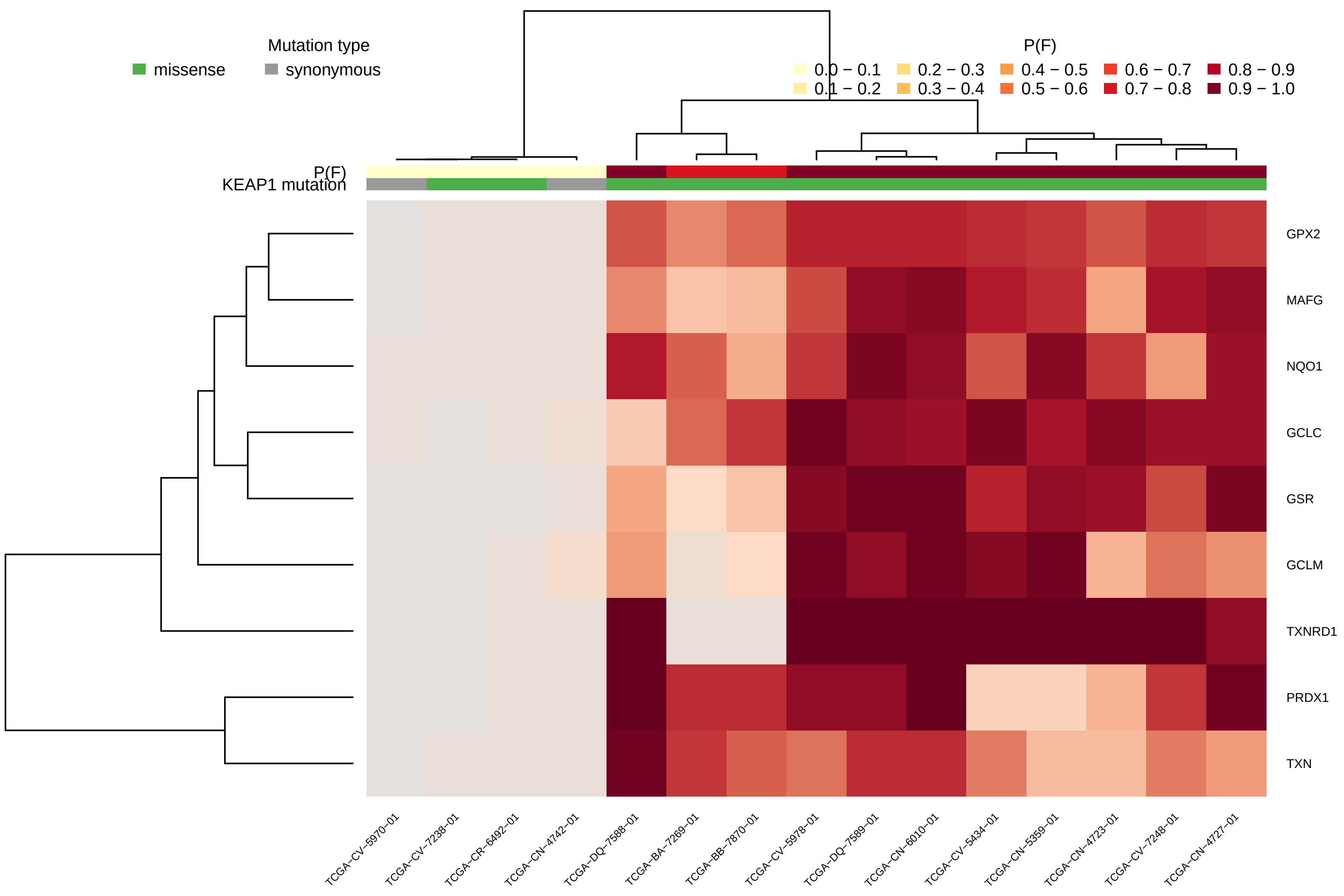

Mutation type
missense
synonymous
P(F)
0.0 − 0.1
0.1 − 0.2
0.2 − 0.3
0.3 − 0.4
0.4 − 0.5
0.5 − 0.6
0.6 − 0.7
0.7 − 0.8
0.8 − 0.9
0.9 − 1.0
P(F)
KEAP1 mutation
GPX2
MAFG
NQO1
GCLC
GSR
GCLM
TXNRD1
PRDX1
TXN
TCGA−CV−5970−01
TCGA−CV−7238−01
TCGA−CR−6492−01
TCGA−CN−4742−01
TCGA−DQ−7588−01
TCGA−BA−7269−01
TCGA−BB−7870−01
TCGA−CV−5978−01
TCGA−DQ−7589−01
TCGA−CN−6010−01
TCGA−CV−5434−01
TCGA−CN−5359−01
TCGA−CN−4723−01
TCGA−CV−7248−01
TCGA−CN−4727−01

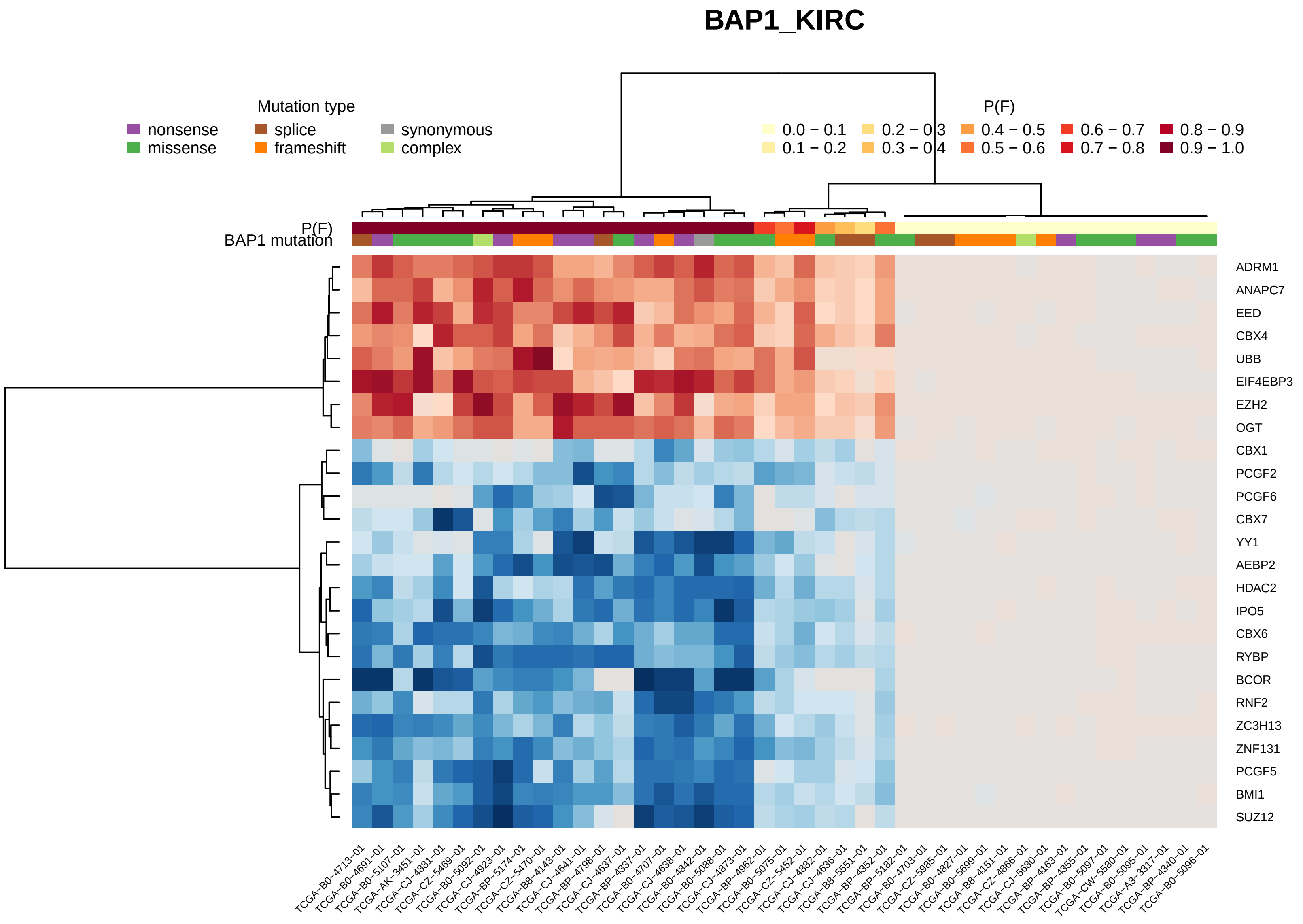
BAP1_KIRC
Mutation type
nonsense
missense
splice
frameshift
synonymous
complex
P(F)
0.0 − 0.1
0.1 − 0.2
0.2 − 0.3
0.3 − 0.4
0.4 − 0.5
0.5 − 0.6
0.6 − 0.7
0.7 − 0.8
0.8 − 0.9
0.9 − 1.0
P(F)
BAP1 mutation
ADRM1
ANAPC7
EED
CBX4
UBB
EIF4EBP3
EZH2
OGT
CBX1
PCGF2
PCGF6
CBX7
YY1
AEBP2
HDAC2
IPO5
CBX6
RYBP
BCOR
RNF2
ZC3H13
ZNF131
PCGF5
BMI1
SUZ12
TCGA−B0−4713−01
TCGA−B0−4691−01
TCGA−B0−5107−01
TCGA−AK−3451−01
TCGA−CJ−4881−01
TCGA−CZ−5469−01
TCGA−B0−5092−01
TCGA−CJ−4923−01
TCGA−BP−5174−01
TCGA−CZ−5470−01
TCGA−B8−4143−01
TCGA−CJ−4641−01
TCGA−BP−4798−01
TCGA−CJ−4637−01
TCGA−BP−4337−01
TCGA−B0−4707−01
TCGA−CJ−4638−01
TCGA−B0−4842−01
TCGA−B0−5088−01
TCGA−CJ−4873−01
TCGA−BP−4962−01
TCGA−B0−5075−01
TCGA−CZ−5452−01
TCGA−CJ−4882−01
TCGA−CJ−4636−01
TCGA−B8−5551−01
TCGA−BP−4352−01
TCGA−BP−5182−01
TCGA−B0−4703−01
TCGA−CZ−5985−01
TCGA−B0−4827−01
TCGA−B0−5699−01
TCGA−B8−4151−01
TCGA−CZ−4866−01
TCGA−CJ−5680−01
TCGA−BP−4163−01
TCGA−BP−4355−01
TCGA−B0−5097−01
TCGA−CW−5580−01
TCGA−B0−5095−01
TCGA−A3−3317−01
TCGA−BP−4340−01
TCGA−B0−5096−01

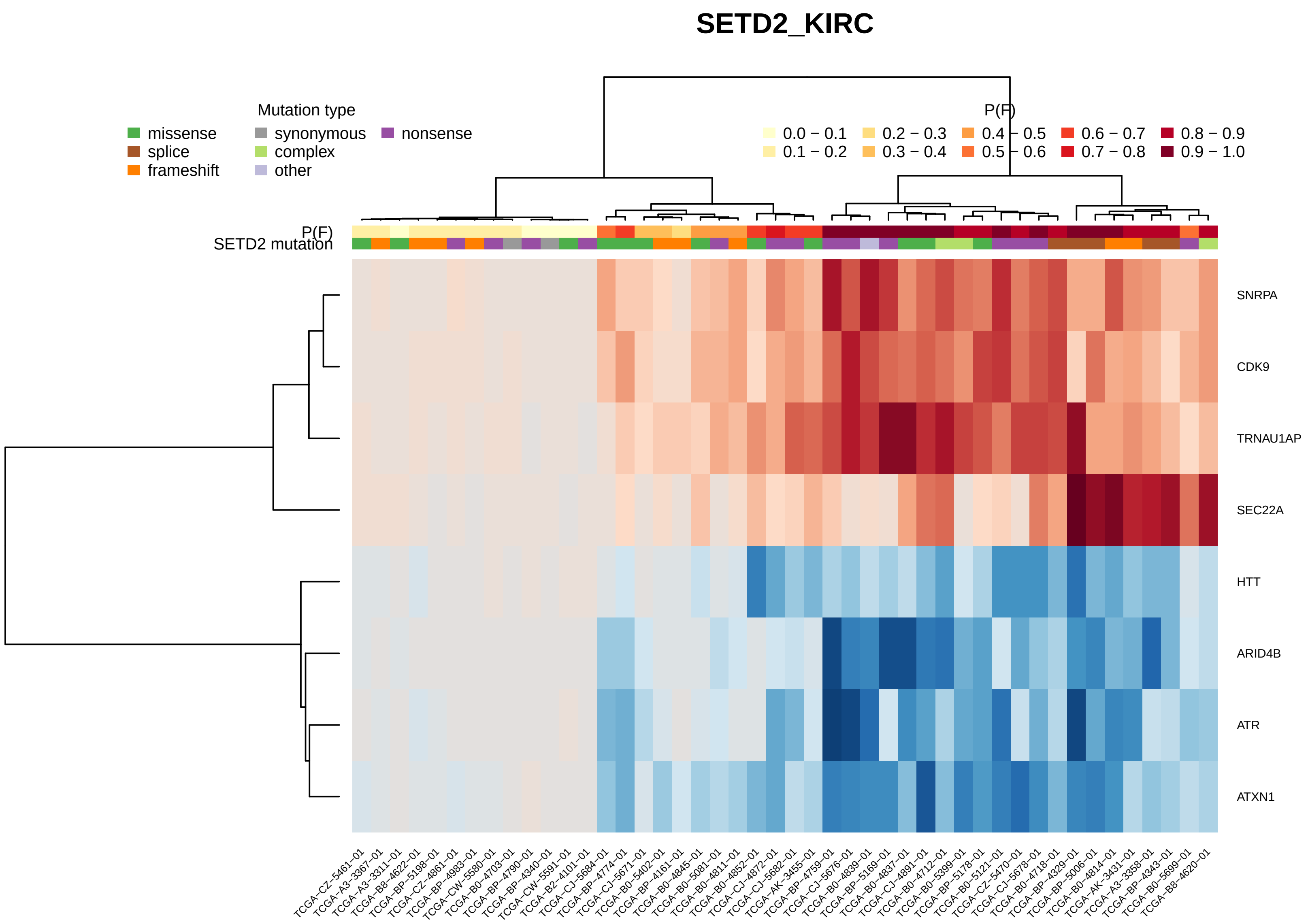

SETD2_KIRC
Mutation type
missense
splice
frameshift
synonymous
complex
other
nonsense
P(F)
0.0 − 0.1
0.1 − 0.2
0.2 − 0.3
0.3 − 0.4
0.4 − 0.5
0.5 − 0.6
0.6 − 0.7
0.7 − 0.8
0.8 − 0.9
0.9 − 1.0
P(F)
SETD2 mutation
SNRPA
CDK9
TRNAU1AP
SEC22A
HTT
ARID4B
ATR
ATXN1
TCGA−CZ−5461−01
TCGA−A3−3367−01
TCGA−A3−3311−01
TCGA−B8−4622−01
TCGA−BP−5198−01
TCGA−CZ−4861−01
TCGA−BP−4983−01
TCGA−CW−5580−01
TCGA−B0−4703−01
TCGA−BP−4790−01
TCGA−BP−4340−01
TCGA−CW−5591−01
TCGA−B2−4101−01
TCGA−CJ−5684−01
TCGA−BP−4774−01
TCGA−CJ−5671−01
TCGA−B0−5402−01
TCGA−BP−4161−01
TCGA−B0−4845−01
TCGA−B0−5081−01
TCGA−B0−4811−01
TCGA−B0−4852−01
TCGA−CJ−4872−01
TCGA−CJ−5682−01
TCGA−AK−3455−01
TCGA−BP−4759−01
TCGA−CJ−5676−01
TCGA−B0−4839−01
TCGA−BP−5169−01
TCGA−B0−4837−01
TCGA−CJ−4891−01
TCGA−B0−4712−01
TCGA−B0−5399−01
TCGA−BP−5178−01
TCGA−B0−5121−01
TCGA−CZ−5470−01
TCGA−CJ−5678−01
TCGA−B0−4718−01
TCGA−BP−4329−01
TCGA−BP−5006−01
TCGA−B0−4814−01
TCGA−AK−3431−01
TCGA−A3−3358−01
TCGA−BP−4343−01
TCGA−B0−5699−01
TCGA−B8−4620−01

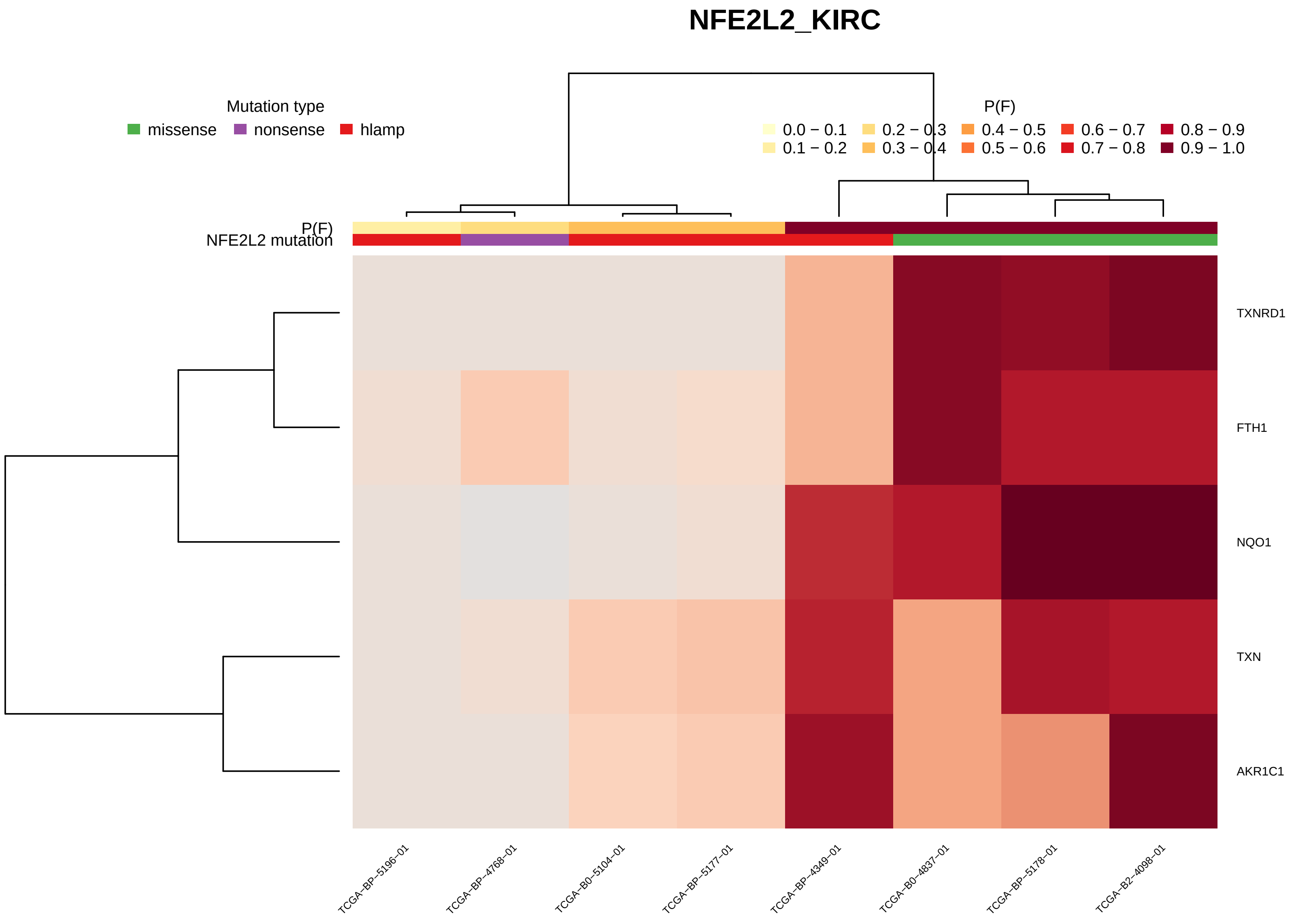
NFE2L2_KIRC
Mutation type
missense
nonsense
hlamp
P(F)
0.0 − 0.1
0.1 − 0.2
0.2 − 0.3
0.3 − 0.4
0.4 − 0.5
0.5 − 0.6
0.6 − 0.7
0.7 − 0.8
0.8 − 0.9
0.9 − 1.0
P(F)
NFE2L2 mutation
TXNRD1
FTH1
NQO1
TXN
AKR1C1
TCGA−BP−5196−01
TCGA−BP−4768−01
TCGA−B0−5104−01
TCGA−BP−5177−01
TCGA−BP−4349−01
TCGA−B0−4837−01
TCGA−BP−5178−01
TCGA−B2−4098−01

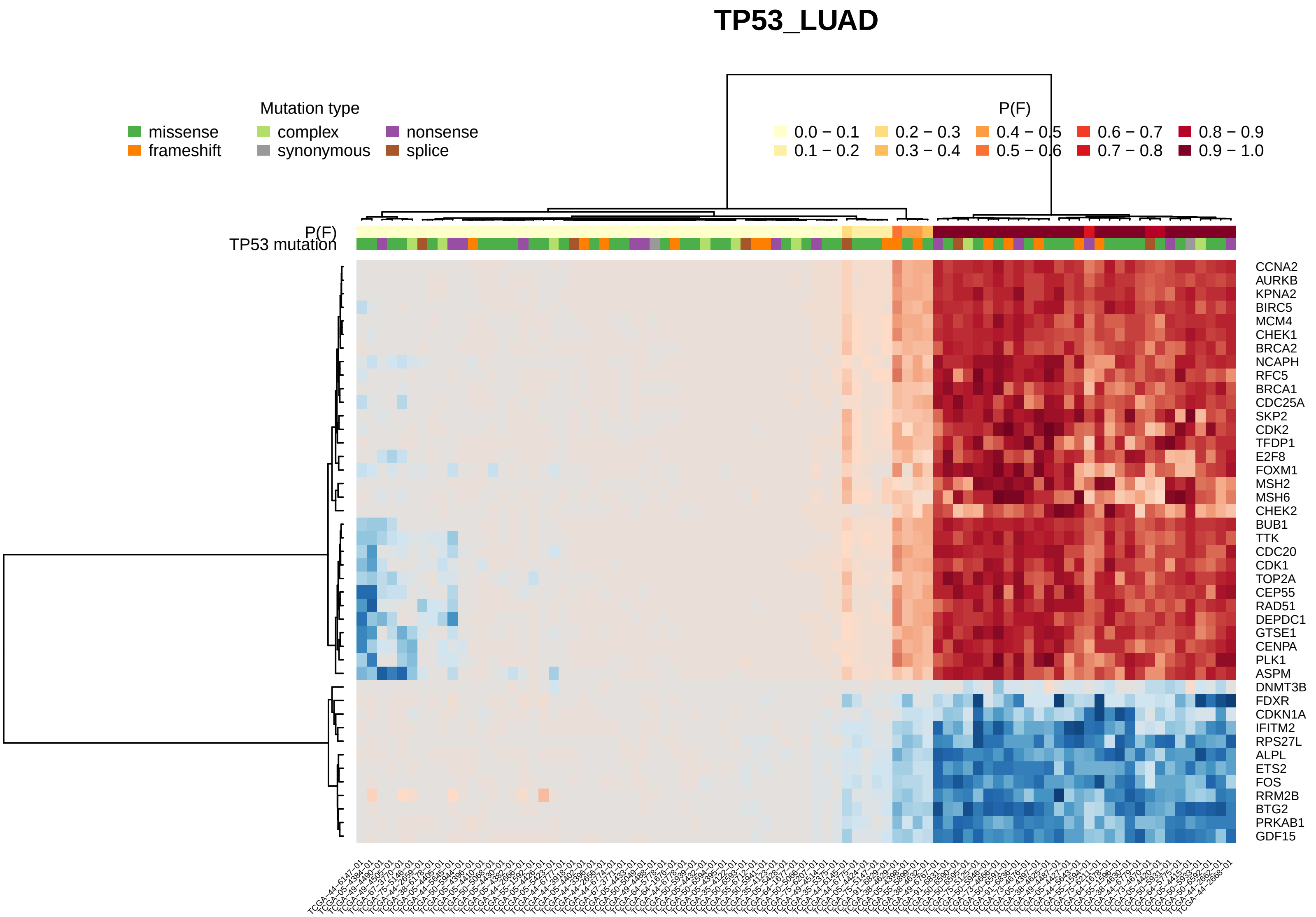
TP53_LUAD
Mutation type
missense
complex
nonsense
frameshift
synonymous
splice
P(F)
0.0 − 0.1
0.1 − 0.2
0.2 − 0.3
0.3 − 0.4
0.4 − 0.5
0.5 − 0.6
0.6 − 0.7
0.7 − 0.8
0.8 − 0.9
0.9 − 1.0
P(F)
TP53 mutation
CCNA2
AURKB
KPNA2
BIRC5
MCM4
CHEK1
BRCA2
NCAPH
RFC5
BRCA1
CDC25A
SKP2
CDK2
TFDP1
E2F8
FOXM1
MSH2
MSH6
CHEK2
BUB1
TTK
CDC20
CDK1
TOP2A
CEP55
RAD51
DEPDC1
GTSE1
CENPA
PLK1
ASPM
DNMT3B
FDXR
CDKN1A
IFITM2
RPS27L
ALPL
ETS2
FOS
RRM2B
BTG2
PRKAB1
GDF15

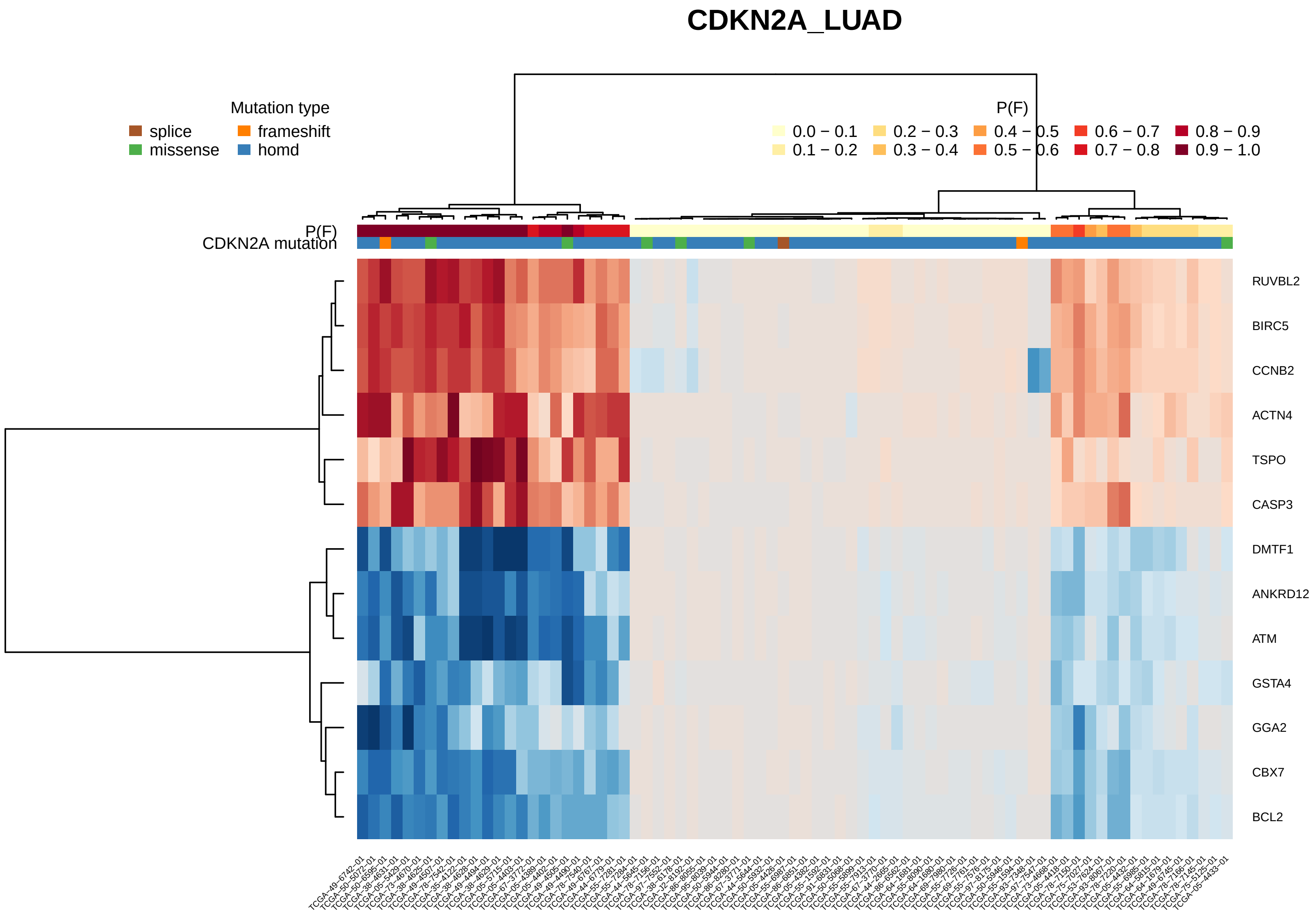
CDKN2A_LUAD
Mutation type
splice
frameshift
missense
homd
P(F)
0.0 − 0.1
0.1 − 0.2
0.2 − 0.3
0.3 − 0.4
0.4 − 0.5
0.5 − 0.6
0.6 − 0.7
0.7 − 0.8
0.8 − 0.9
0.9 − 1.0
P(F)
CDKN2A mutation
RUVBL2
BIRC5
CCNB2
ACTN4
TSPO
CASP3
DMTF1
ANKRD12
ATM
GSTA4
GGA2
CBX7
BCL2

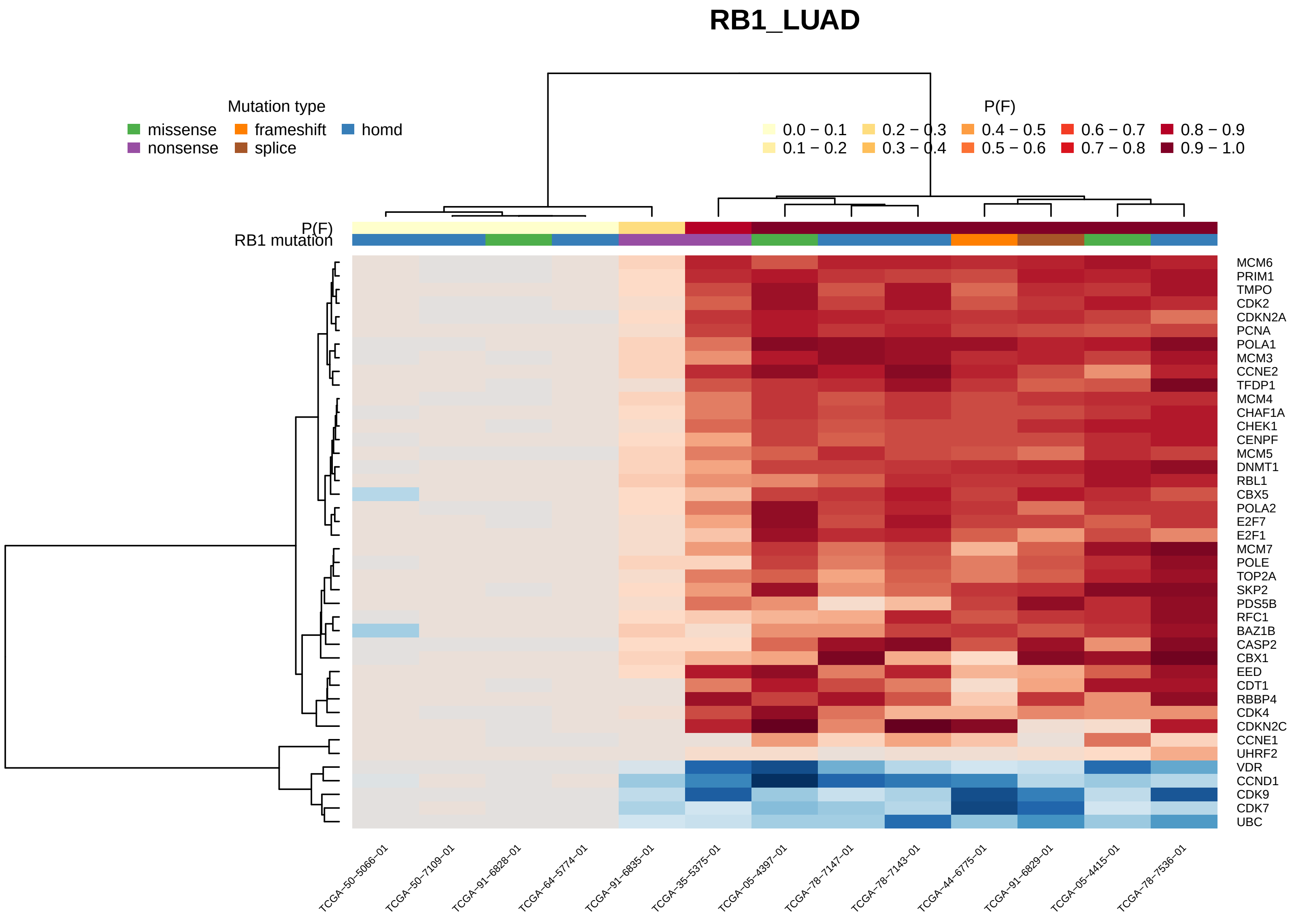
RB1_LUAD
Mutation type
missense
frameshift
homd
nonsense
splice
P(F)
0.0 − 0.1
0.1 − 0.2
0.2 − 0.3
0.3 − 0.4
0.4 − 0.5
0.5 − 0.6
0.6 − 0.7
0.7 − 0.8
0.8 − 0.9
0.9 − 1.0
P(F)
RB1 mutation
MCM6
PRIM1
TMPO
CDK2
CDKN2A
PCNA
POLA1
MCM3
CCNE2
TFDP1
MCM4
CHAF1A
CHEK1
CENPF
MCM5
DNMT1
RBL1
CBX5
POLA2
E2F7
E2F1
MCM7
POLE
TOP2A
SKP2
PDS5B
RFC1
BAZ1B
CASP2
CBX1
EED
CDT1
RBBP4
CDK4
CDKN2C
CCNE1
UHRF2
VDR
CCND1
CDK9
CDK7
UBC
TCGA−50−5066−01
TCGA−50−7109−01
TCGA−91−6828−01
TCGA−64−5774−01
TCGA−91−6835−01
TCGA−35−5375−01
TCGA−05−4397−01
TCGA−78−7147−01
TCGA−78−7143−01
TCGA−44−6775−01
TCGA−91−6829−01
TCGA−05−4415−01
TCGA−78−7536−01

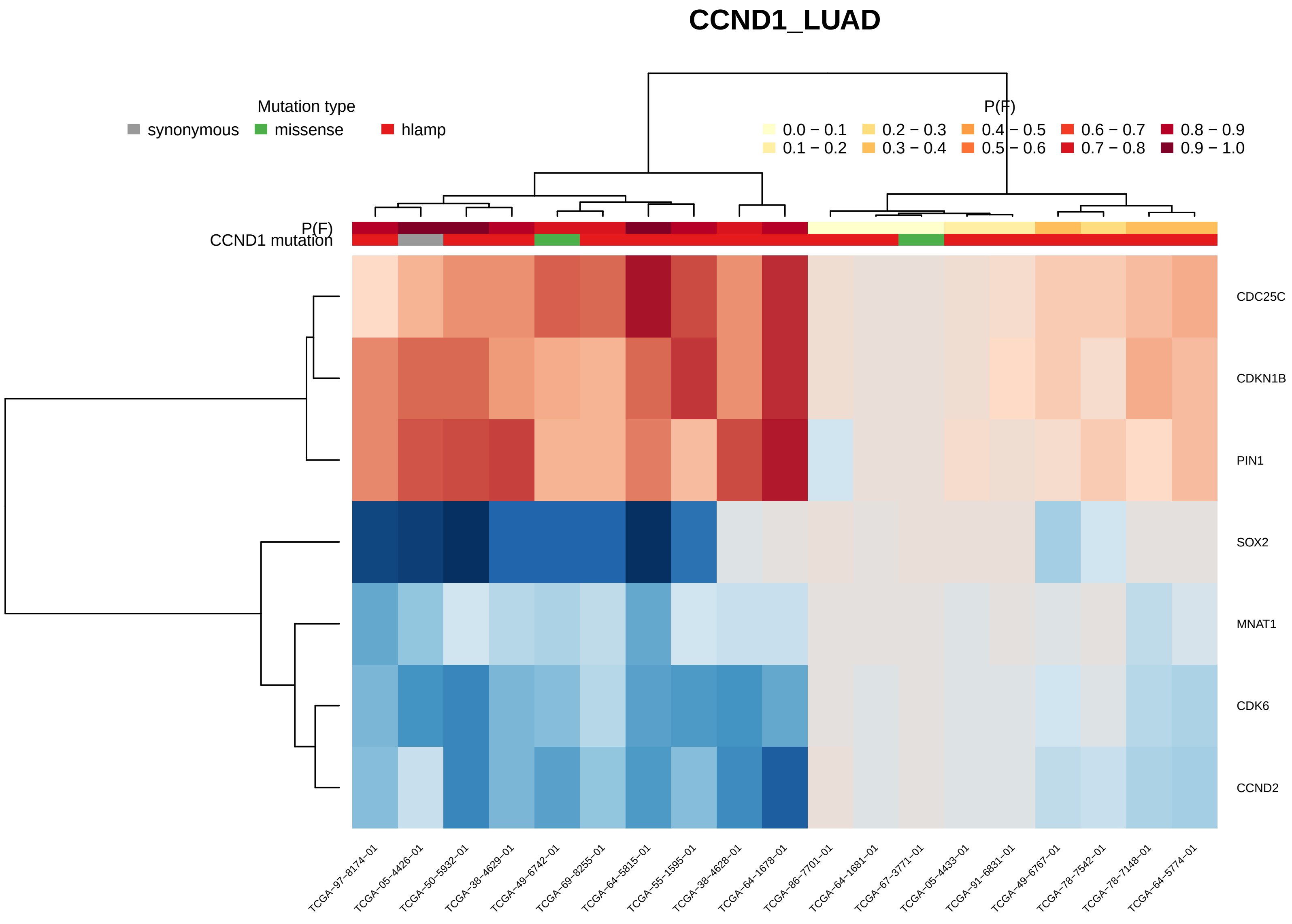

CCND1_LUAD
Mutation type
synonymous
missense
hlamp
P(F)
0.0 − 0.1
0.1 − 0.2
0.2 − 0.3
0.3 − 0.4
0.4 − 0.5
0.5 − 0.6
0.6 − 0.7
0.7 − 0.8
0.8 − 0.9
0.9 − 1.0
P(F)
CCND1 mutation
CDC25C
CDKN1B
PIN1
SOX2
MNAT1
CDK6
CCND2
TCGA−97−8174−01
TCGA−05−4426−01
TCGA−50−5932−01
TCGA−38−4629−01
TCGA−49−6742−01
TCGA−69−8255−01
TCGA−64−5815−01
TCGA−55−1595−01
TCGA−38−4628−01
TCGA−64−1678−01
TCGA−86−7701−01
TCGA−64−1681−01
TCGA−67−3771−01
TCGA−05−4433−01
TCGA−91−6831−01
TCGA−49−6767−01
TCGA−78−7542−01
TCGA−78−7148−01
TCGA−64−5774−01

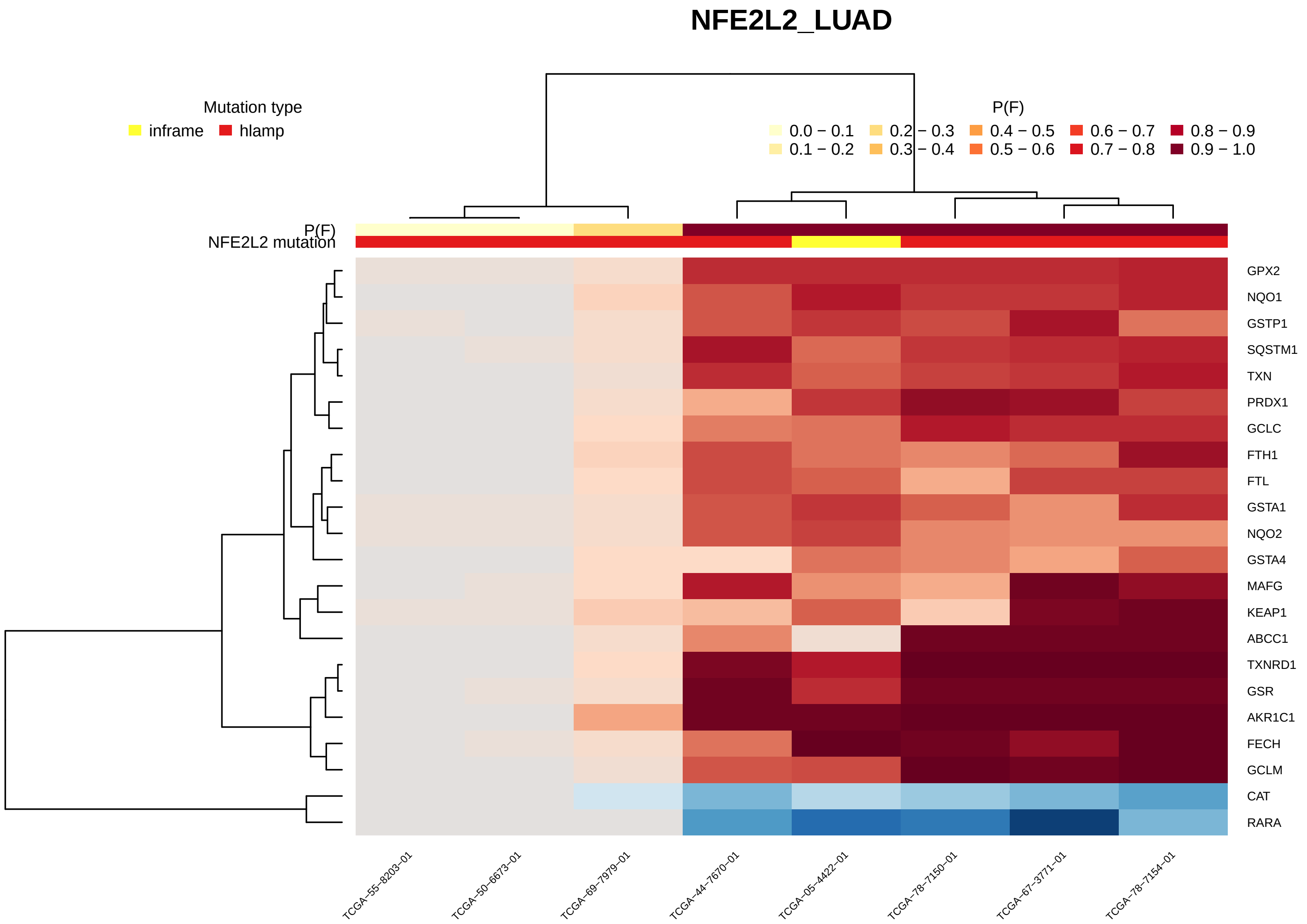

NFE2L2_LUAD
Mutation type
inframe
hlamp
P(F)
0.0 − 0.1
0.1 − 0.2
0.2 − 0.3
0.3 − 0.4
0.4 − 0.5
0.5 − 0.6
0.6 − 0.7
0.7 − 0.8
0.8 − 0.9
0.9 − 1.0
P(F)
NFE2L2 mutation
GPX2
NQO1
GSTP1
SQSTM1
TXN
PRDX1
GCLC
FTH1
FTL
GSTA1
NQO2
GSTA4
MAFG
KEAP1
ABCC1
TXNRD1
GSR
AKR1C1
FECH
GCLM
CAT
RARA
TCGA−55−8203−01
TCGA−50−6673−01
TCGA−69−7979−01
TCGA−44−7670−01
TCGA−05−4422−01
TCGA−78−7150−01
TCGA−67−3771−01
TCGA−78−7154−01

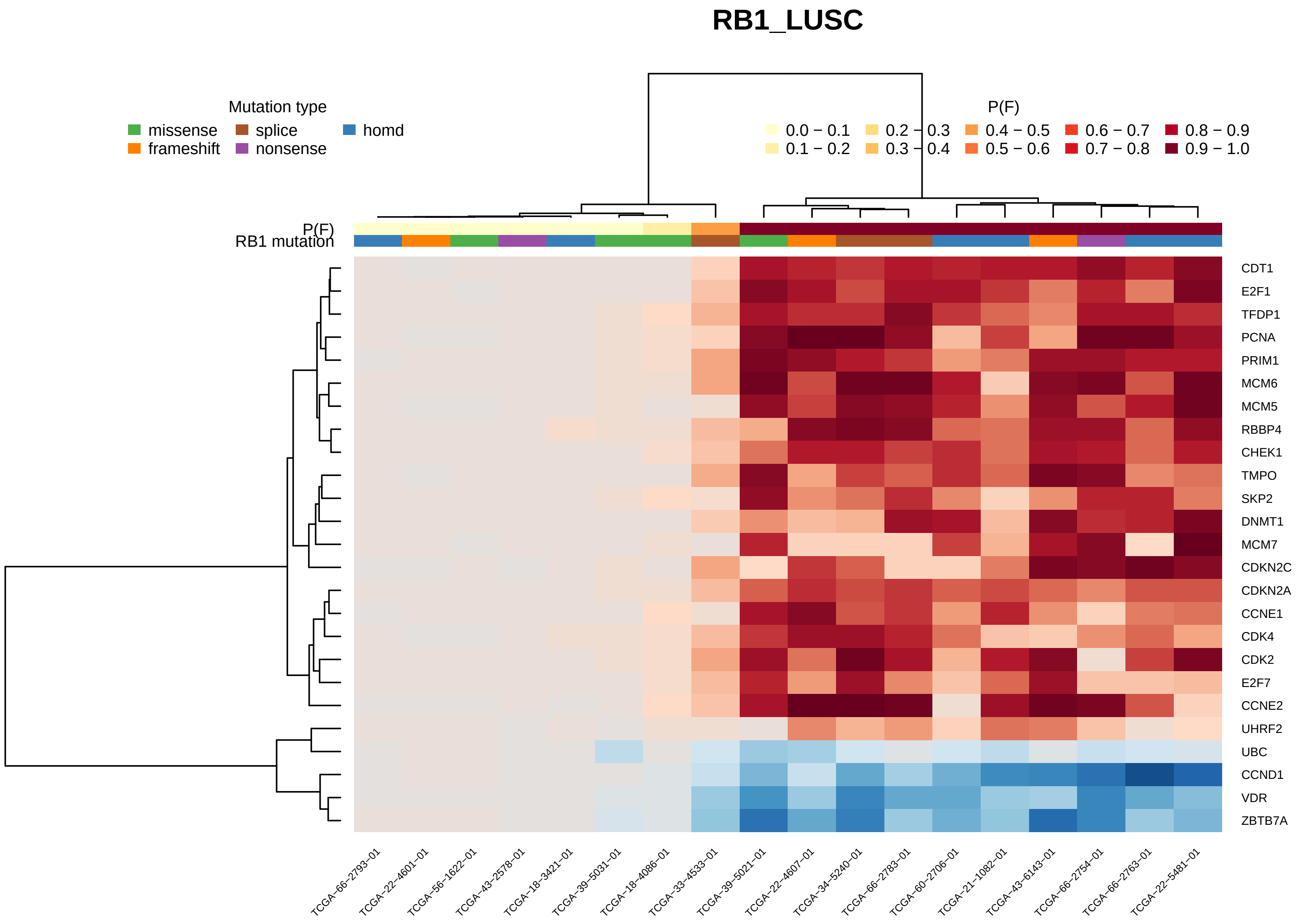

RB1_LUSC
Mutation type
missense
splice
homd
frameshift
nonsense
P(F)
0.0 − 0.1
0.1 − 0.2
0.2 − 0.3
0.3 − 0.4
0.4 − 0.5
0.5 − 0.6
0.6 − 0.7
0.7 − 0.8
0.8 − 0.9
0.9 − 1.0
P(F)
RB1 mutation
CDT1
E2F1
TFDP1
PCNA
PRIM1
MCM6
MCM5
RBBP4
CHEK1
TMPO
SKP2
DNMT1
MCM7
CDKN2C
CDKN2A
CCNE1
CDK4
CDK2
E2F7
CCNE2
UHRF2
UBC
CCND1
VDR
ZBTB7A
TCGA−66−2793−01
TCGA−22−4601−01
TCGA−56−1622−01
TCGA−43−2578−01
TCGA−18−3421−01
TCGA−39−5031−01
TCGA−18−4086−01
TCGA−33−4533−01
TCGA−39−5021−01
TCGA−22−4607−01
TCGA−34−5240−01
TCGA−66−2783−01
TCGA−60−2706−01
TCGA−21−1082−01
TCGA−43−6143−01
TCGA−66−2754−01
TCGA−66−2763−01
TCGA−22−5481−01

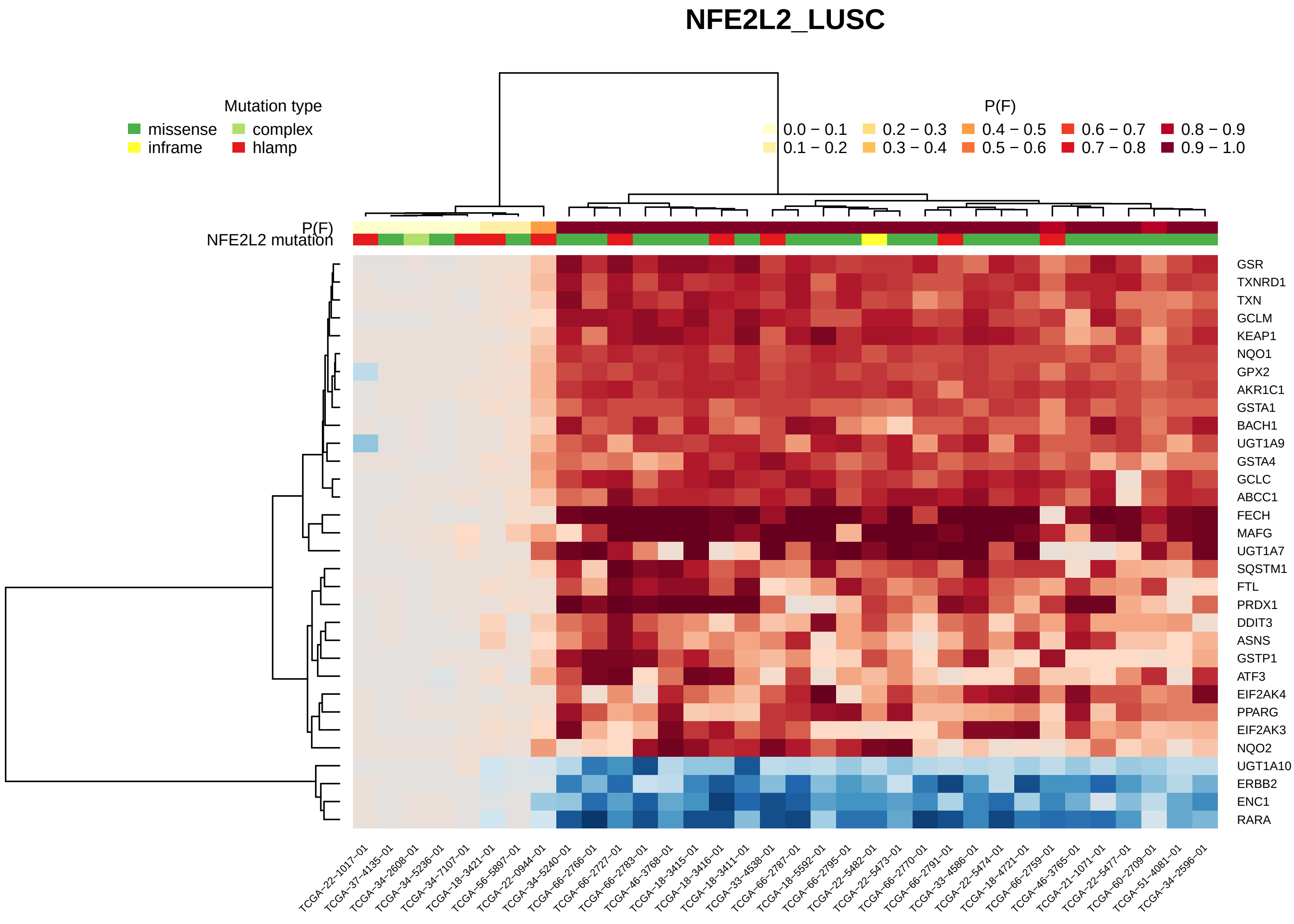
NFE2L2_LUSC
Mutation type
missense
complex
inframe
hlamp
P(F)
0.0 − 0.1
0.1 − 0.2
0.2 − 0.3
0.3 − 0.4
0.4 − 0.5
0.5 − 0.6
0.6 − 0.7
0.7 − 0.8
0.8 − 0.9
0.9 − 1.0
P(F)
NFE2L2 mutation
GSR
TXNRD1
TXN
GCLM
KEAP1
NQO1
GPX2
AKR1C1
GSTA1
BACH1
UGT1A9
GSTA4
GCLC
ABCC1
FECH
MAFG
UGT1A7
SQSTM1
FTL
PRDX1
DDIT3
ASNS
GSTP1
ATF3
EIF2AK4
PPARG
EIF2AK3
NQO2
UGT1A10
ERBB2
ENC1
RARA
TCGA−22−1017−01
TCGA−37−4135−01
TCGA−34−2608−01
TCGA−34−5236−01
TCGA−34−7107−01
TCGA−18−3421−01
TCGA−56−5897−01
TCGA−22−0944−01
TCGA−34−5240−01
TCGA−66−2766−01
TCGA−66−2727−01
TCGA−66−2783−01
TCGA−46−3768−01
TCGA−18−3415−01
TCGA−18−3416−01
TCGA−18−3411−01
TCGA−33−4538−01
TCGA−66−2787−01
TCGA−18−5592−01
TCGA−66−2795−01
TCGA−22−5482−01
TCGA−22−5473−01
TCGA−66−2770−01
TCGA−66−2791−01
TCGA−33−4586−01
TCGA−22−5474−01
TCGA−18−4721−01
TCGA−66−2759−01
TCGA−46−3765−01
TCGA−21−1071−01
TCGA−22−5477−01
TCGA−60−2709−01
TCGA−51−4081−01
TCGA−34−2596−01

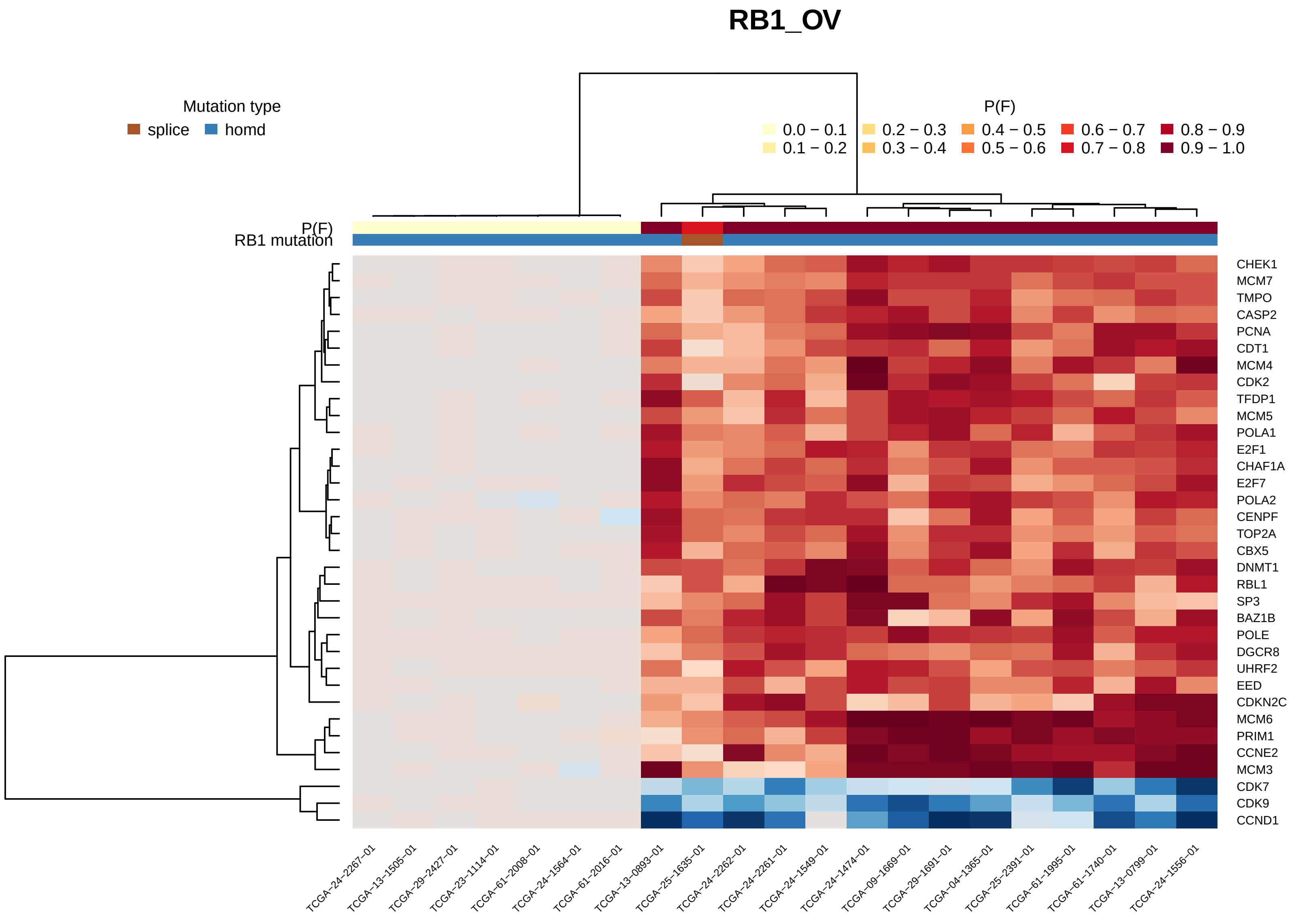

RB1_OV
Mutation type
splice
homd
P(F)
0.0 − 0.1
0.1 − 0.2
0.2 − 0.3
0.3 − 0.4
0.4 − 0.5
0.5 − 0.6
0.6 − 0.7
0.7 − 0.8
0.8 − 0.9
0.9 − 1.0
P(F)
RB1 mutation
CHEK1
MCM7
TMPO
CASP2
PCNA
CDT1
MCM4
CDK2
TFDP1
MCM5
POLA1
E2F1
CHAF1A
E2F7
POLA2
CENPF
TOP2A
CBX5
DNMT1
RBL1
SP3
BAZ1B
POLE
DGCR8
UHRF2
EED
CDKN2C
MCM6
PRIM1
CCNE2
MCM3
CDK7
CDK9
CCND1
TCGA−24−2267−01
TCGA−13−1505−01
TCGA−29−2427−01
TCGA−23−1114−01
TCGA−61−2008−01
TCGA−24−1564−01
TCGA−61−2016−01
TCGA−13−0893−01
TCGA−25−1635−01
TCGA−24−2262−01
TCGA−24−2261−01
TCGA−24−1549−01
TCGA−24−1474−01
TCGA−09−1669−01
TCGA−29−1691−01
TCGA−04−1365−01
TCGA−25−2391−01
TCGA−61−1995−01
TCGA−61−1740−01
TCGA−13−0799−01
TCGA−24−1556−01

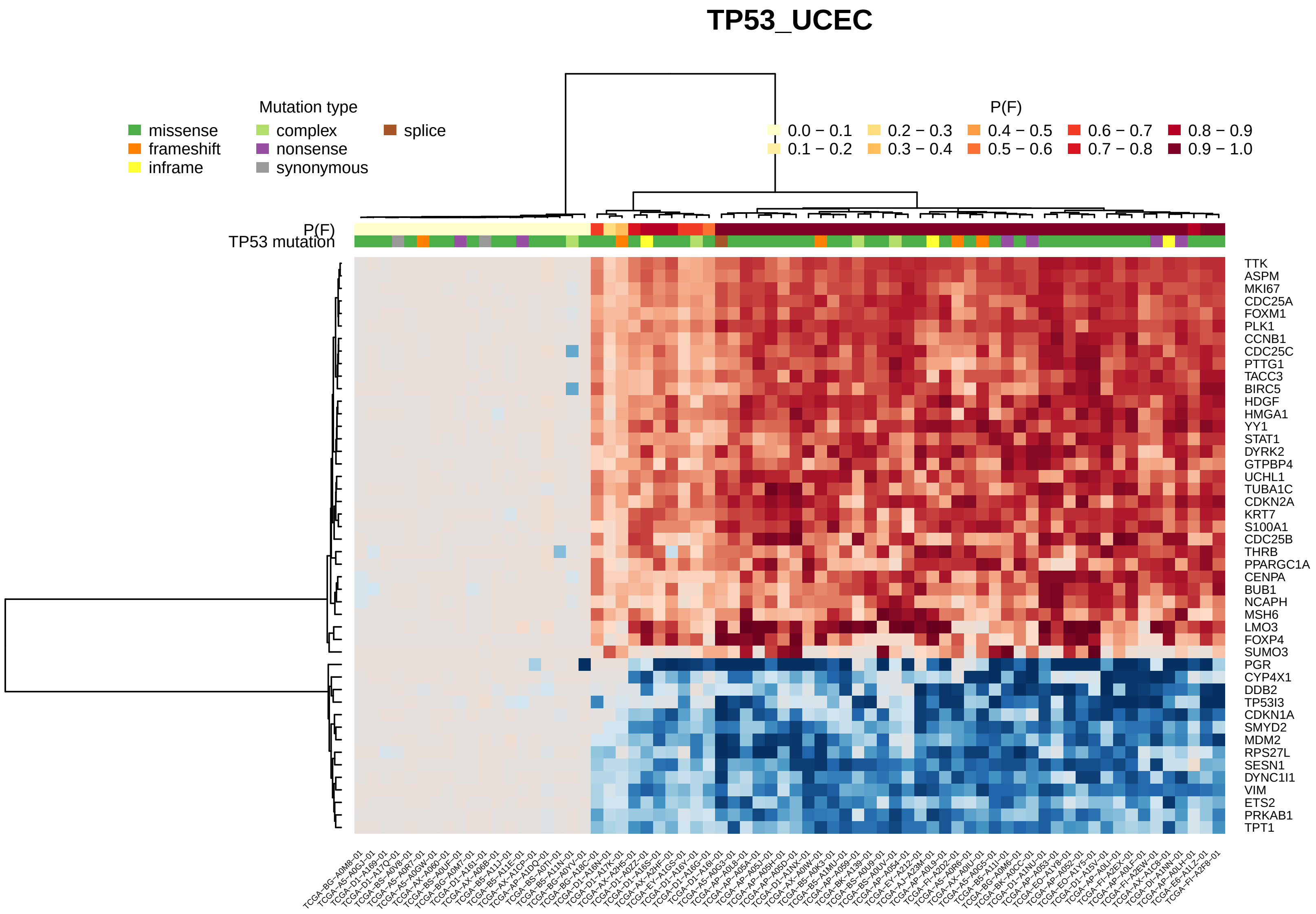
TP53_UCEC
Mutation type
missense
frameshift
inframe
complex
nonsense
synonymous
splice
P(F)
0.0 − 0.1
0.1 − 0.2
0.2 − 0.3
0.3 − 0.4
0.4 − 0.5
0.5 − 0.6
0.6 − 0.7
0.7 − 0.8
0.8 − 0.9
0.9 − 1.0
P(F)
TP53 mutation
TTK
ASPM
MKI67
CDC25A
FOXM1
PLK1
CCNB1
CDC25C
PTTG1
TACC3
BIRC5
HDGF
HMGA1
YY1
STAT1
DYRK2
GTPBP4
UCHL1
TUBA1C
CDKN2A
KRT7
S100A1
CDC25B
THRB
PPARGC1A
CENPA
BUB1
NCAPH
MSH6
LMO3
FOXP4
SUMO3
PGR
CYP4X1
DDB2
TP53I3
CDKN1A
SMYD2
MDM2
RPS27L
SESN1
DYNC1I1
VIM
ETS2
PRKAB1
TPT1

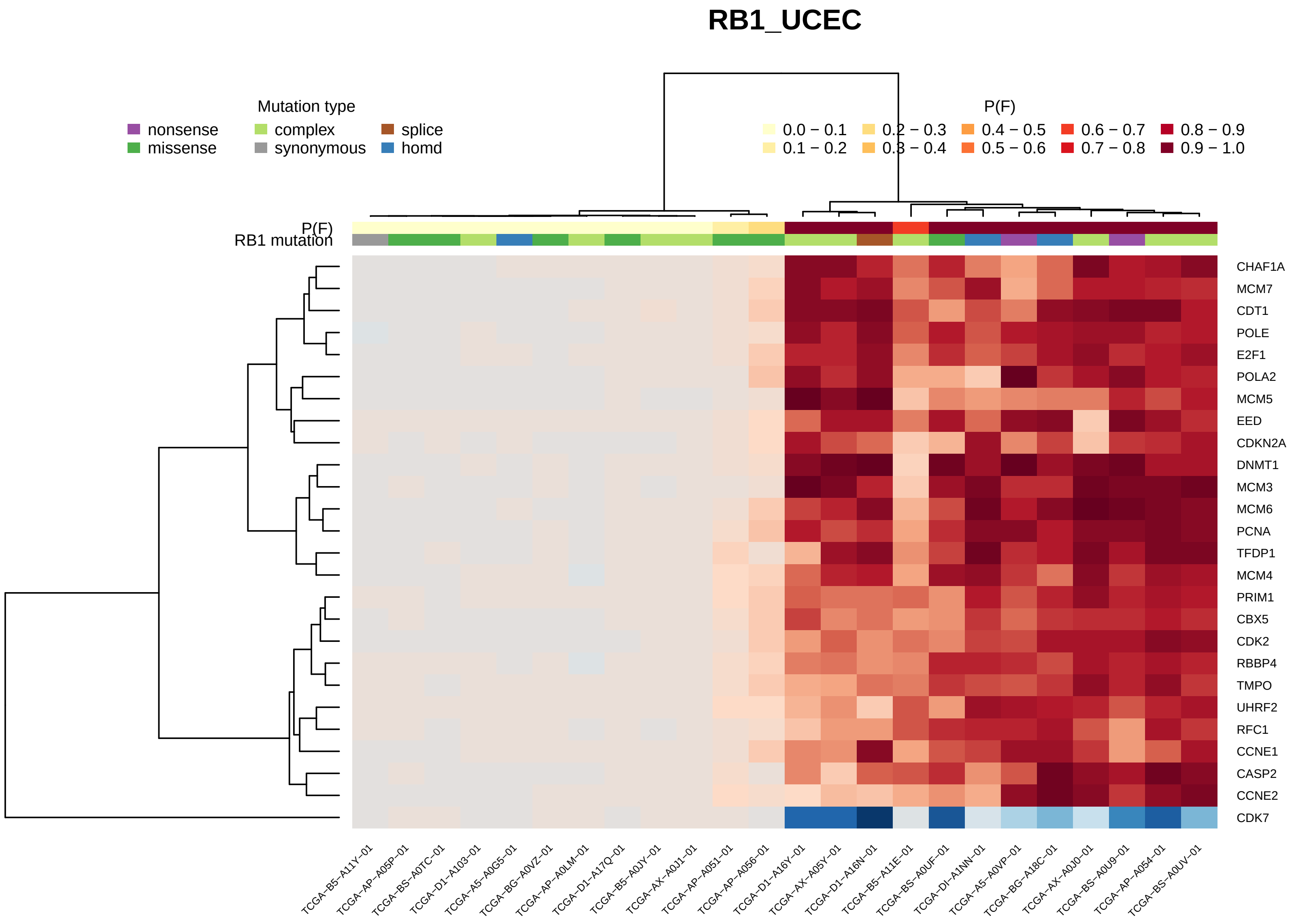

RB1_UCEC
Mutation type
nonsense
missense
complex
synonymous
splice
homd
P(F)
0.0 − 0.1
0.1 − 0.2
0.2 − 0.3
0.3 − 0.4
0.4 − 0.5
0.5 − 0.6
0.6 − 0.7
0.7 − 0.8
0.8 − 0.9
0.9 − 1.0
P(F)
RB1 mutation
CHAF1A
MCM7
CDT1
POLE
E2F1
POLA2
MCM5
EED
CDKN2A
DNMT1
MCM3
MCM6
PCNA
TFDP1
MCM4
PRIM1
CBX5
CDK2
RBBP4
TMPO
UHRF2
RFC1
CCNE1
CASP2
CCNE2
CDK7
TCGA−B5−A11Y−01
TCGA−AP−A05P−01
TCGA−BS−A0TC−01
TCGA−D1−A103−01
TCGA−A5−A0G5−01
TCGA−BG−A0VZ−01
TCGA−AP−A0LM−01
TCGA−D1−A17Q−01
TCGA−B5−A0JY−01
TCGA−AX−A0J1−01
TCGA−AP−A051−01
TCGA−AP−A056−01
TCGA−D1−A16Y−01
TCGA−AX−A05Y−01
TCGA−D1−A16N−01
TCGA−B5−A11E−01
TCGA−BS−A0UF−01
TCGA−DI−A1NN−01
TCGA−A5−A0VP−01
TCGA−BG−A18C−01
TCGA−AX−A0J0−01
TCGA−BS−A0U9−01
TCGA−AP−A054−01
TCGA−BS−A0UV−01

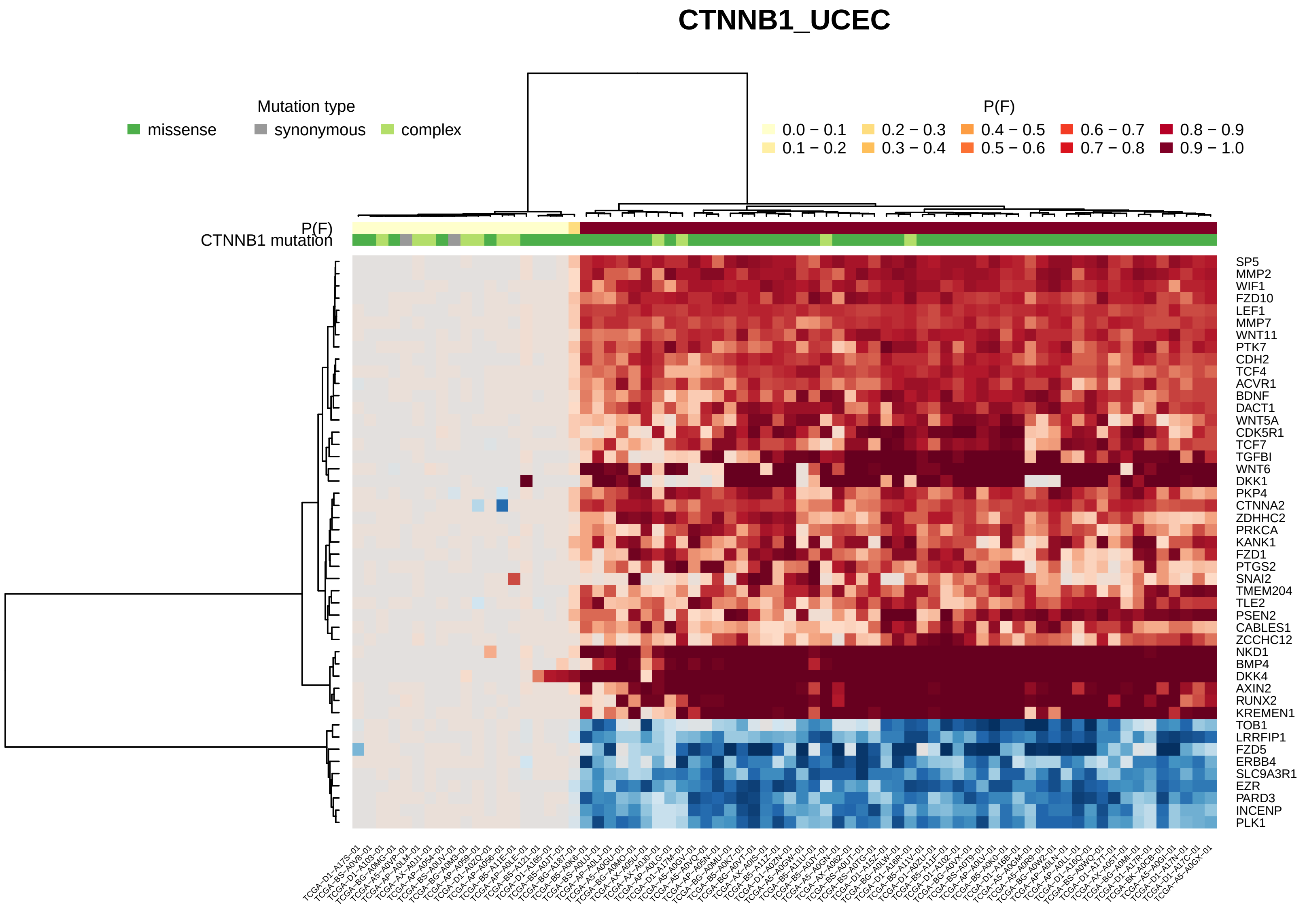
CTNNB1_UCEC
Mutation type
missense
synonymous
complex
P(F)
0.0 − 0.1
0.1 − 0.2
0.2 − 0.3
0.3 − 0.4
0.4 − 0.5
0.5 − 0.6
0.6 − 0.7
0.7 − 0.8
0.8 − 0.9
0.9 − 1.0
P(F)
CTNNB1 mutation
SP5
MMP2
WIF1
FZD10
LEF1
MMP7
WNT11
PTK7
CDH2
TCF4
ACVR1
BDNF
DACT1
WNT5A
CDK5R1
TCF7
TGFBI
WNT6
DKK1
PKP4
CTNNA2
ZDHHC2
PRKCA
KANK1
FZD1
PTGS2
SNAI2
TMEM204
TLE2
PSEN2
CABLES1
ZCCHC12
NKD1
BMP4
DKK4
AXIN2
RUNX2
KREMEN1
TOB1
LRRFIP1
FZD5
ERBB4
SLC9A3R1
EZR
PARD3
INCENP
PLK1

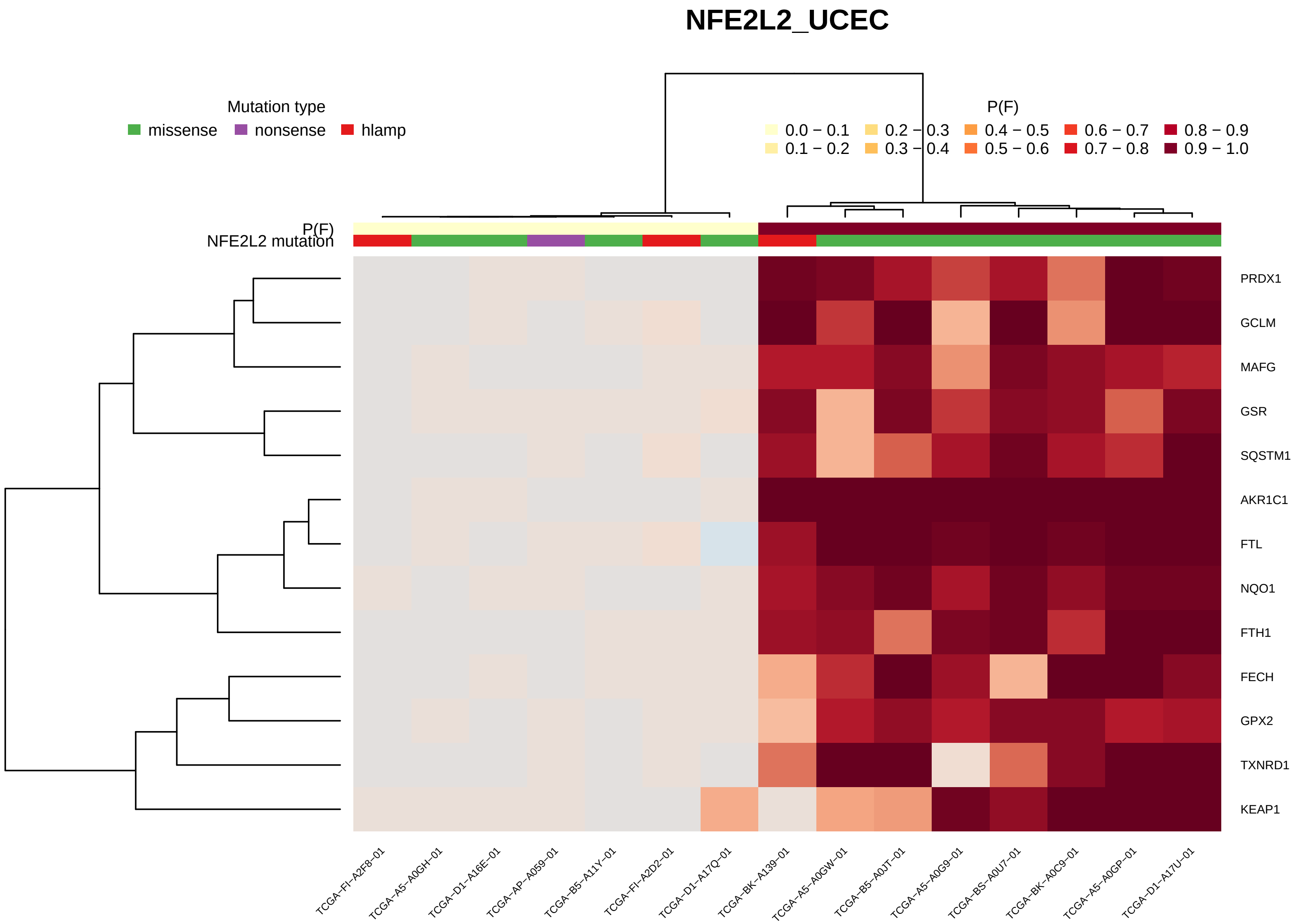

NFE2L2_UCEC
Mutation type
missense
nonsense
hlamp
P(F)
0.0 − 0.1
0.1 − 0.2
0.2 − 0.3
0.3 − 0.4
0.4 − 0.5
0.5 − 0.6
0.6 − 0.7
0.7 − 0.8
0.8 − 0.9
0.9 − 1.0
P(F)
NFE2L2 mutation
PRDX1
GCLM
MAFG
GSR
SQSTM1
AKR1C1
FTL
NQO1
FTH1
FECH
GPX2
TXNRD1
KEAP1
TCGA−FI−A2F8−01
TCGA−A5−A0GH−01
TCGA−D1−A16E−01
TCGA−AP−A059−01
TCGA−B5−A11Y−01
TCGA−FI−A2D2−01
TCGA−D1−A17Q−01
TCGA−BK−A139−01
TCGA−A5−A0GW−01
TCGA−B5−A0JT−01
TCGA−A5−A0G9−01
TCGA−BS−A0U7−01
TCGA−BK−A0C9−01
TCGA−A5−A0GP−01
TCGA−D1−A17U−01

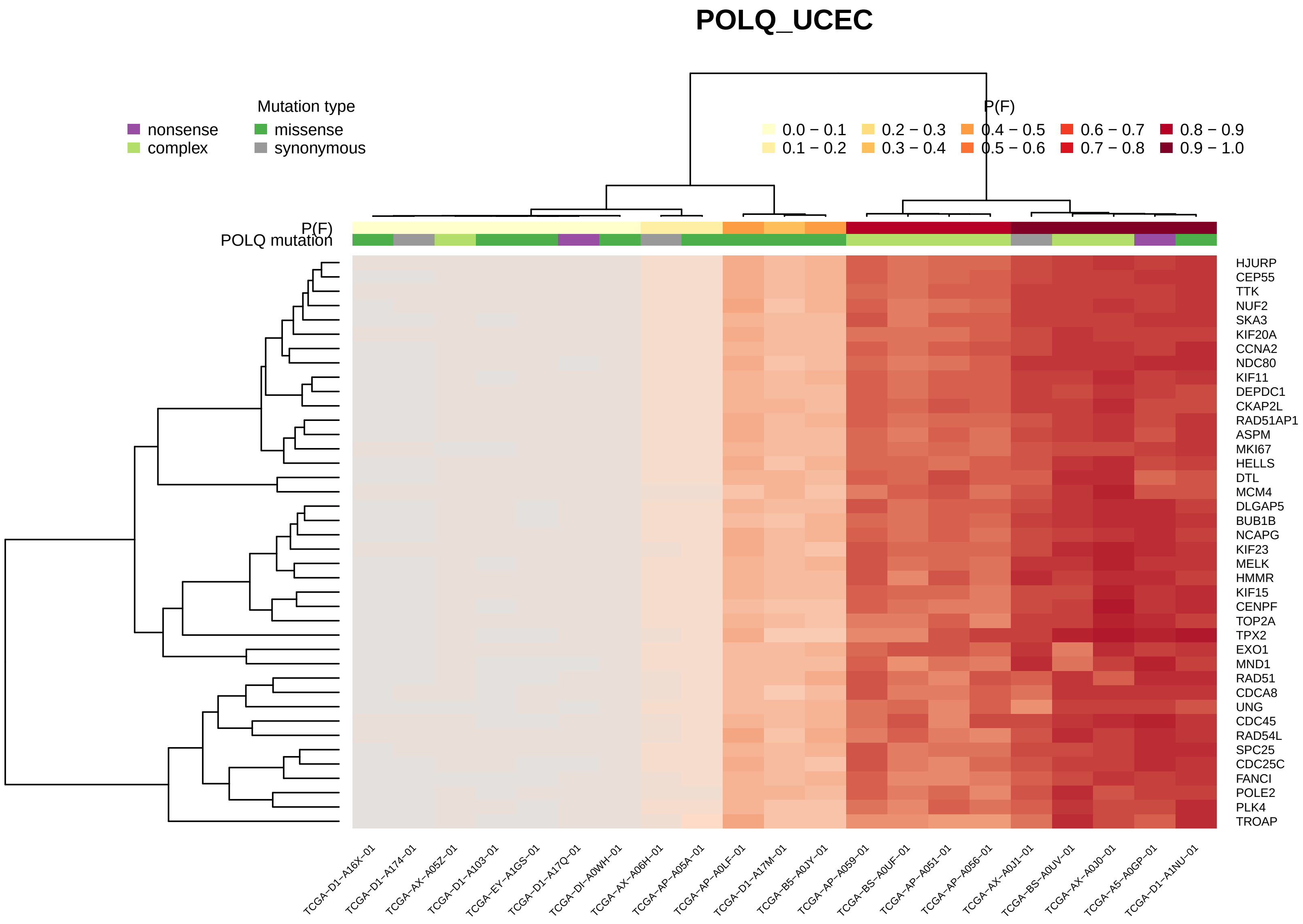
POLQ_UCEC
Mutation type
nonsense
complex
missense
synonymous
P(F)
0.0 − 0.1
0.1 − 0.2
0.2 − 0.3
0.3 − 0.4
0.4 − 0.5
0.5 − 0.6
0.6 − 0.7
0.7 − 0.8
0.8 − 0.9
0.9 − 1.0
P(F)
POLQ mutation
HJURP
CEP55
TTK
NUF2
SKA3
KIF20A
CCNA2
NDC80
KIF11
DEPDC1
CKAP2L
RAD51AP1
ASPM
MKI67
HELLS
DTL
MCM4
DLGAP5
BUB1B
NCAPG
KIF23
MELK
HMMR
KIF15
CENPF
TOP2A
TPX2
EXO1
MND1
RAD51
CDCA8
UNG
CDC45
RAD54L
SPC25
CDC25C
FANCI
POLE2
PLK4
TROAP
TCGA−D1−A16X−01
TCGA−D1−A174−01
TCGA−AX−A05Z−01
TCGA−D1−A103−01
TCGA−EY−A1GS−01
TCGA−D1−A17Q−01
TCGA−DI−A0WH−01
TCGA−AX−A06H−01
TCGA−AP−A05A−01
TCGA−AP−A0LF−01
TCGA−D1−A17M−01
TCGA−B5−A0JY−01
TCGA−AP−A059−01
TCGA−BS−A0UF−01
TCGA−AP−A051−01
TCGA−AP−A056−01
TCGA−AX−A0J1−01
TCGA−BS−A0UV−01
TCGA−AX−A0J0−01
TCGA−A5−A0GP−01
TCGA−D1−A1NU−01

# CHEK2_UCEC

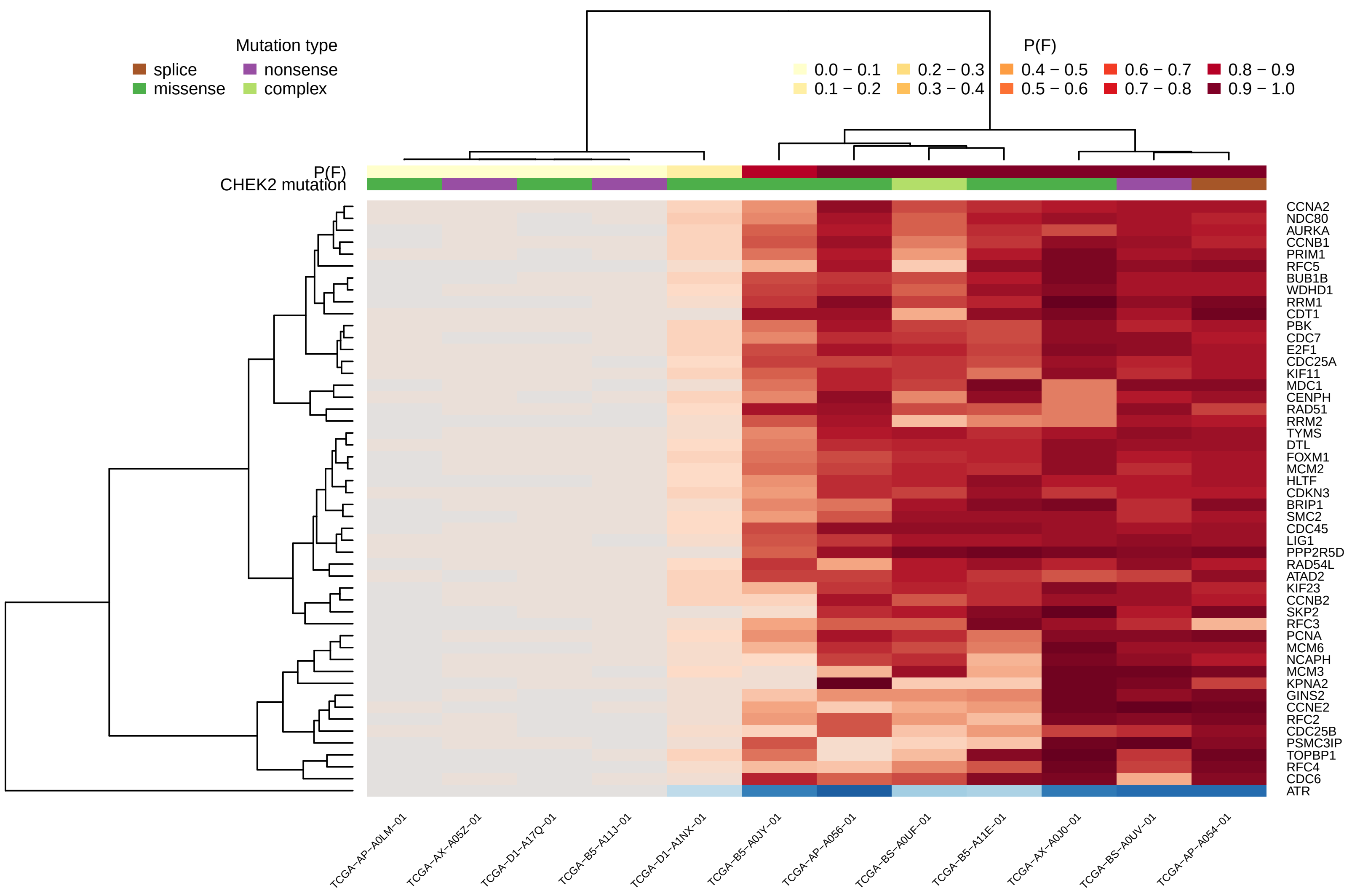

Mutation type
splice
nonsense
missense
complex
P(F)
0.0 − 0.1
0.1 − 0.2
0.2 − 0.3
0.3 − 0.4
0.4 − 0.5
0.5 − 0.6
0.6 − 0.7
0.7 − 0.8
0.8 − 0.9
0.9 − 1.0
P(F)
CHEK2 mutation
CCNA2
NDC80
AURKA
CCNB1
PRIM1
RFC5
BUB1B
WDHD1
RRM1
CDT1
PBK
CDC7
E2F1
CDC25A
KIF11
MDC1
CENPH
RAD51
RRM2
TYMS
DTL
FOXM1
MCM2
HLTF
CDKN3
BRIP1
SMC2
CDC45
LIG1
PPP2R5D
RAD54L
ATAD2
KIF23
CCNB2
SKP2
RFC3
PCNA
MCM6
NCAPH
MCM3
KPNA2
GINS2
CCNE2
RFC2
CDC25B
PSMC3IP
TOPBP1
RFC4
CDC6
ATR
TCGA−AP−A0LM−01
TCGA−AX−A05Z−01
TCGA−D1−A17Q−01
TCGA−B5−A11J−01
TCGA−D1−A1NX−01
TCGA−B5−A0JY−01
TCGA−AP−A056−01
TCGA−BS−A0UF−01
TCGA−B5−A11E−01
TCGA−AX−A0J0−01
TCGA−BS−A0UV−01
TCGA−AP−A054−01

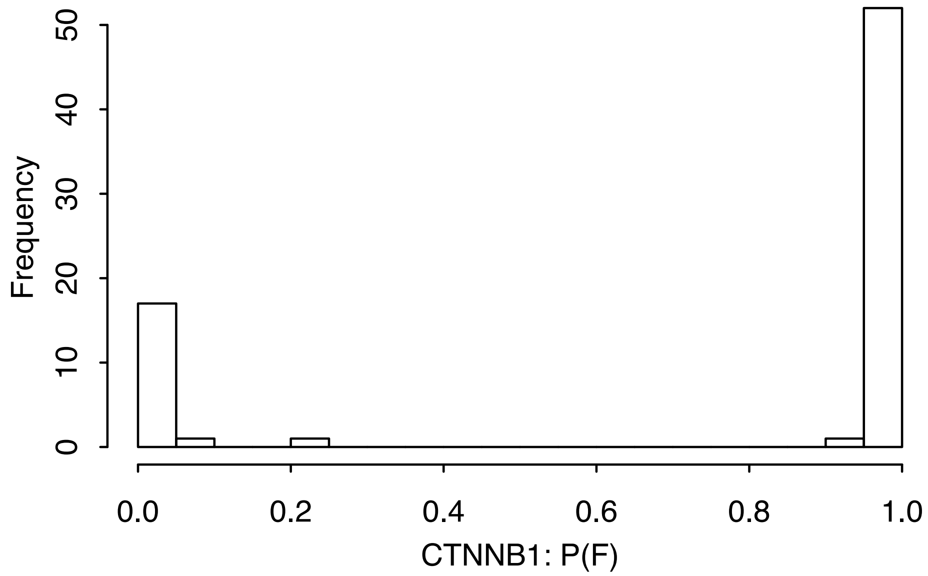


Supplementary Fig. 30: *CTNNB1* mutation $P(F)$ distribution in UCEC. The posterior showed bimodal distributions. We used a threshold of 0.5 to separate high probability mutations from low probability mutations. However, a threshold of 0.2 to 0.8 almost did not change the results.

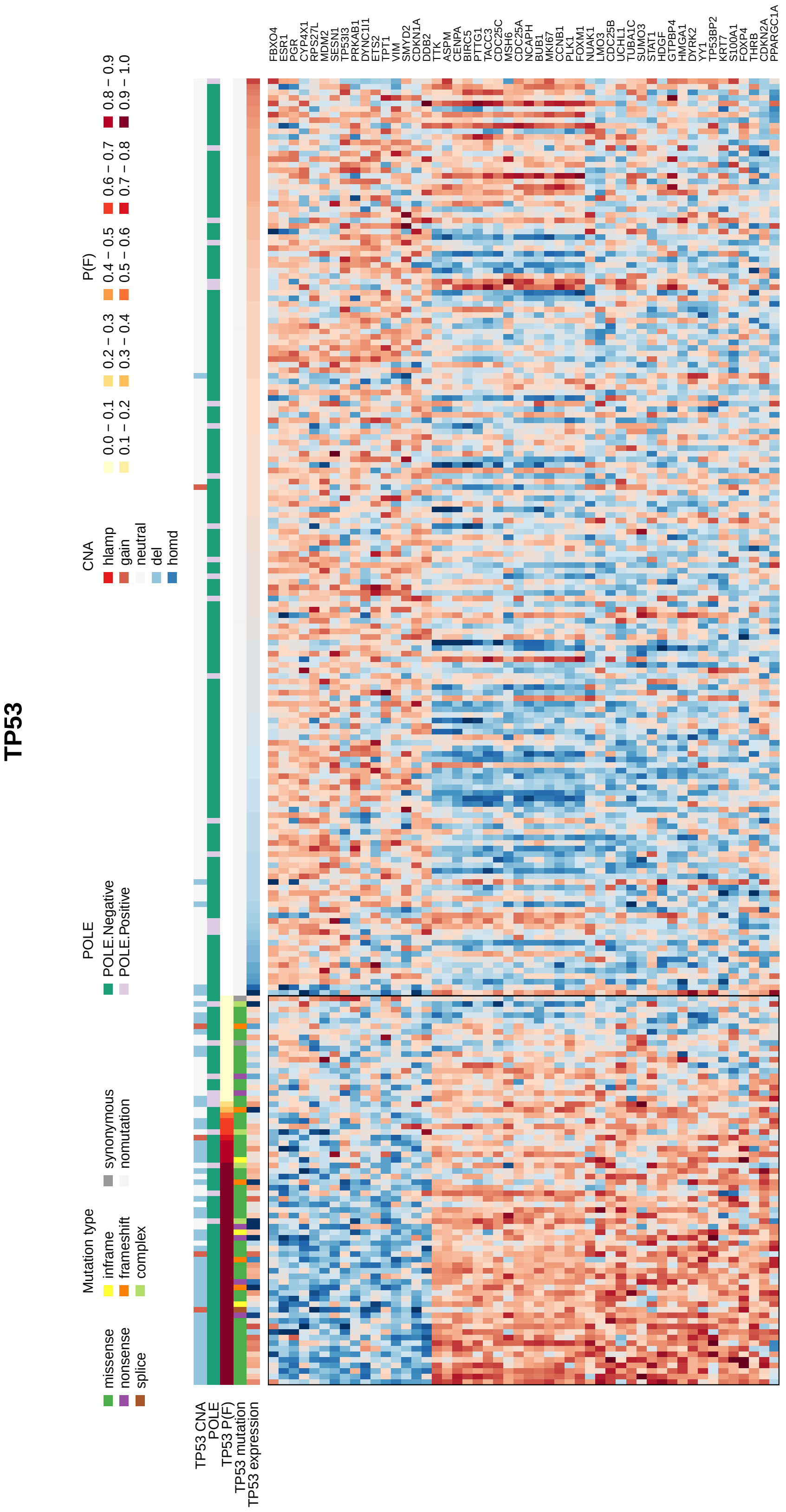


Supplementary Fig. 31: Heatmap shows the expression of genes connected to *TP53* in endometrial cancer. Red represents high expression and blue represents low expression. Here columns represent patients and rows represent genes. Both high probability *TP53* mutations and low probability *TP53* mutations consisted of mostly missense mutations. One patient had *TP53* mutations, but the copy number status was not available.

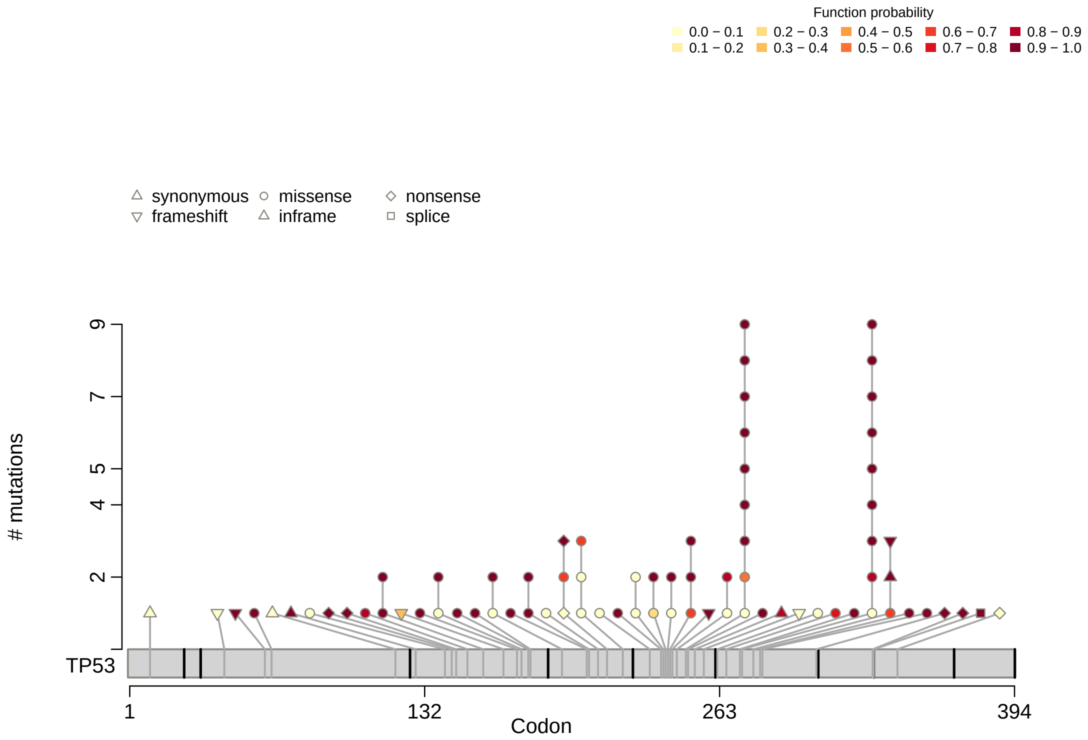


Supplementary Fig. 32: *TP53* mutations and their probabilities $P(F)$ in UCEC. We can see that the high probability mutations can not be simply separated from low probability mutations by mutation sites or types of mutations.

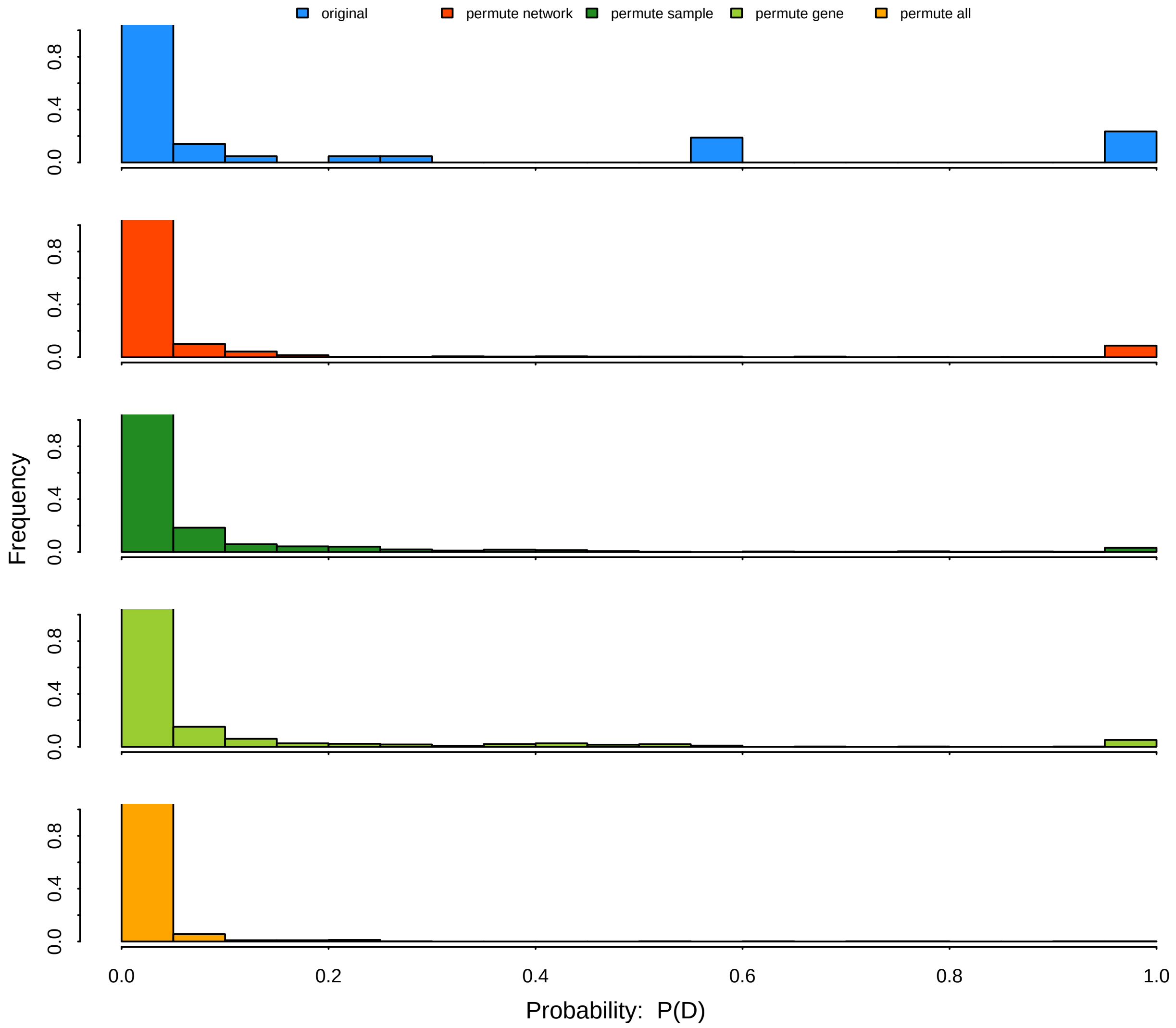


Supplementary Fig. 33: $P(D)$ histograms from the LAML dataset and permuted versions of the LAML dataset. Because very few genes were predicted to have high probabilities $P(D)$, the y-axises of the histograms were limited to 1.

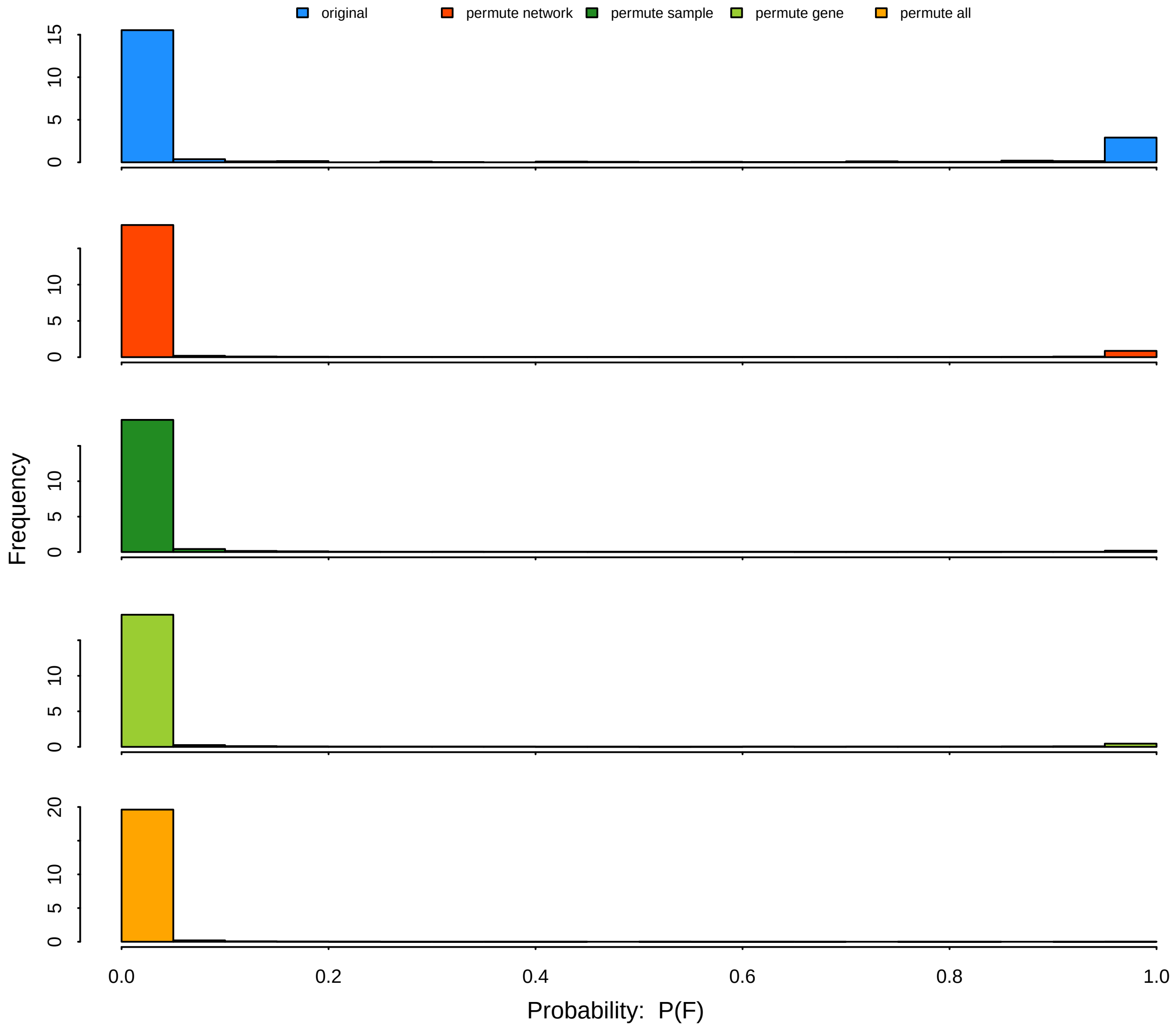


Supplementary Fig. 34: $P(F)$ histograms from the LAML dataset and permuted versions of the LAML dataset

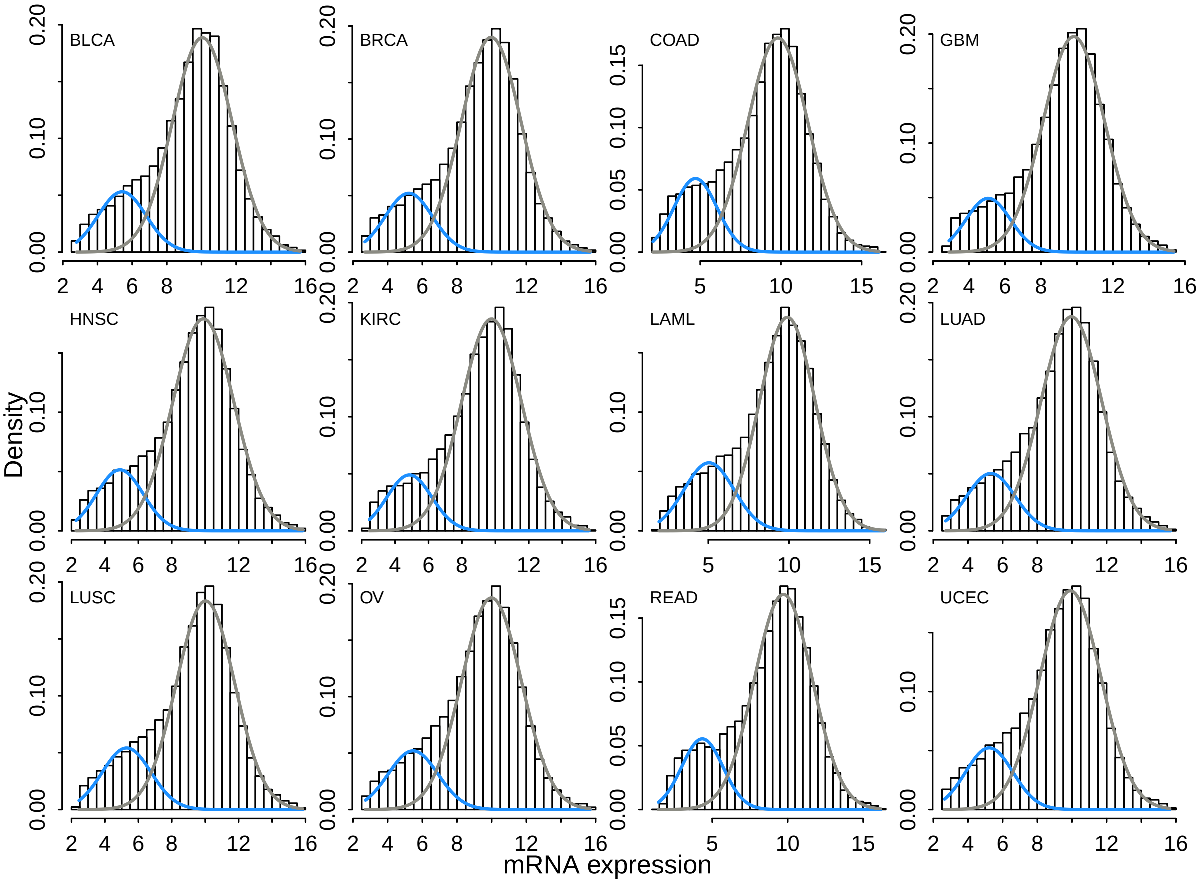


Supplementary Fig. 35: Detect highly-expressed genes based on mixture-of-Gaussian distributions. For each plot, the left blue curve is the Gaussian fit to the “lowly-expressed” genes, and the right grey curve is the Gaussian fit to the “highly-expressed” genes.

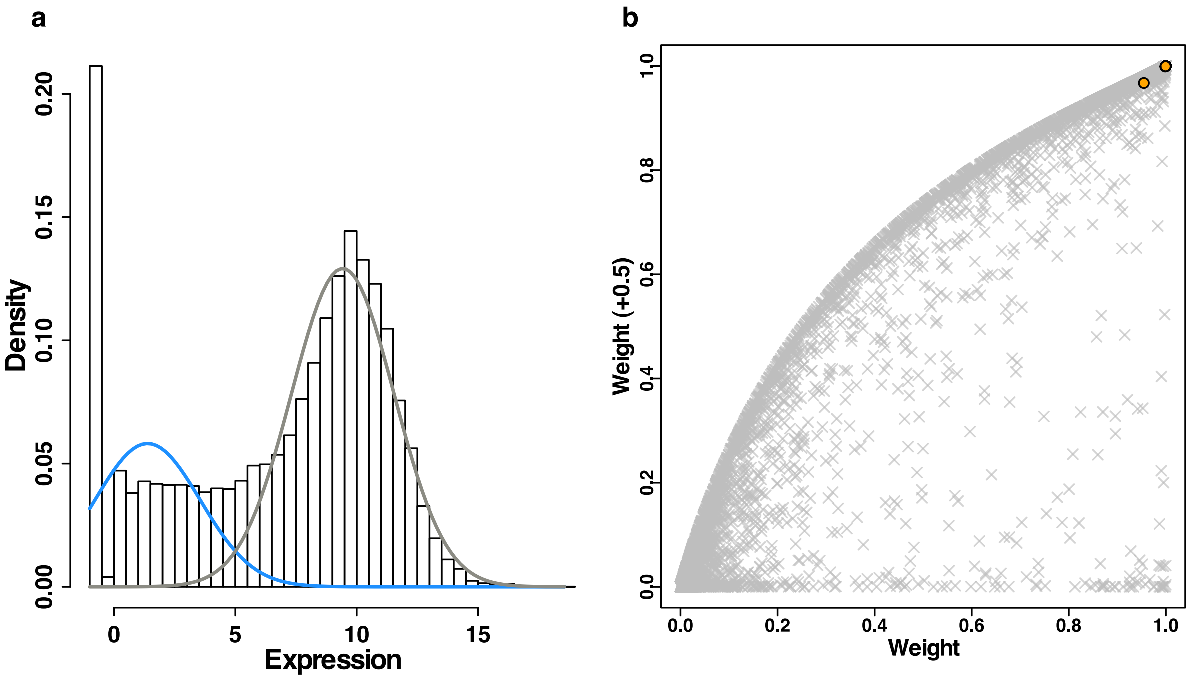


Supplementary Fig. 36: Comparing two RNASeq gene expression normalization methods for detecting highly-expressed genes. (a) A small number of 0.5 was added to the RSEM abundance estimation values before $\log_2$-transformation. Then the $90^{th}$ percentile of the expression of a gene across patients was used to represent the expression of that gene. Many lowly expressed genes were estimated to have exactly the same expression value. A mixture Gaussian distribution might not fit the data very well. (b) The weights computed from both normalization approaches (adding 0.5 before $\log_2$ transformation and assigned NA to genes with 0 RSEM values) were highly correlated (Pearson correlation coefficient: 0.984, Spearman correlation coefficient: 0.995).

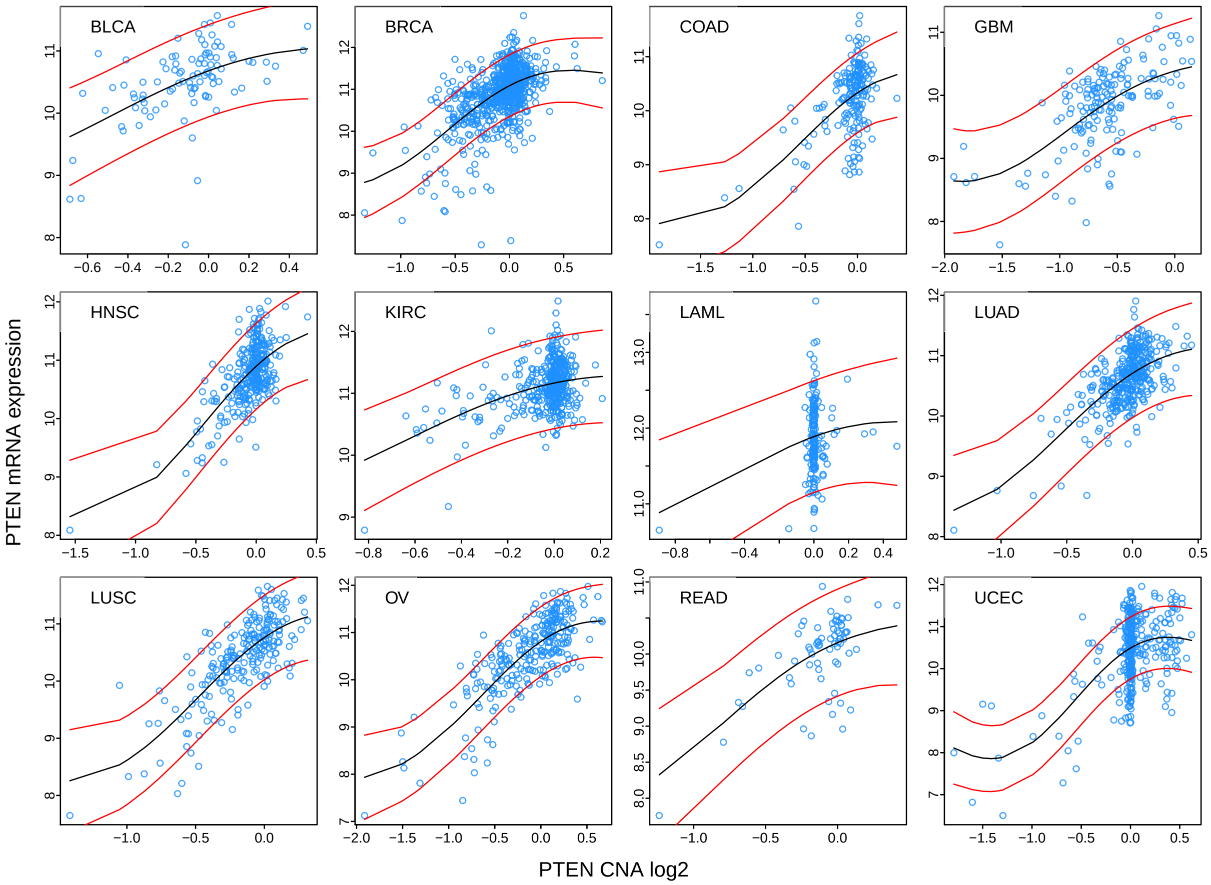


Supplementary Fig. 37: Scatter plots of *PTEN* copy number alterations and expression across 12 cancer types. The black curves are the regression curves estimated by Gaussian process regression, and the region between the two red curves for each plot is the 95% confidence interval.

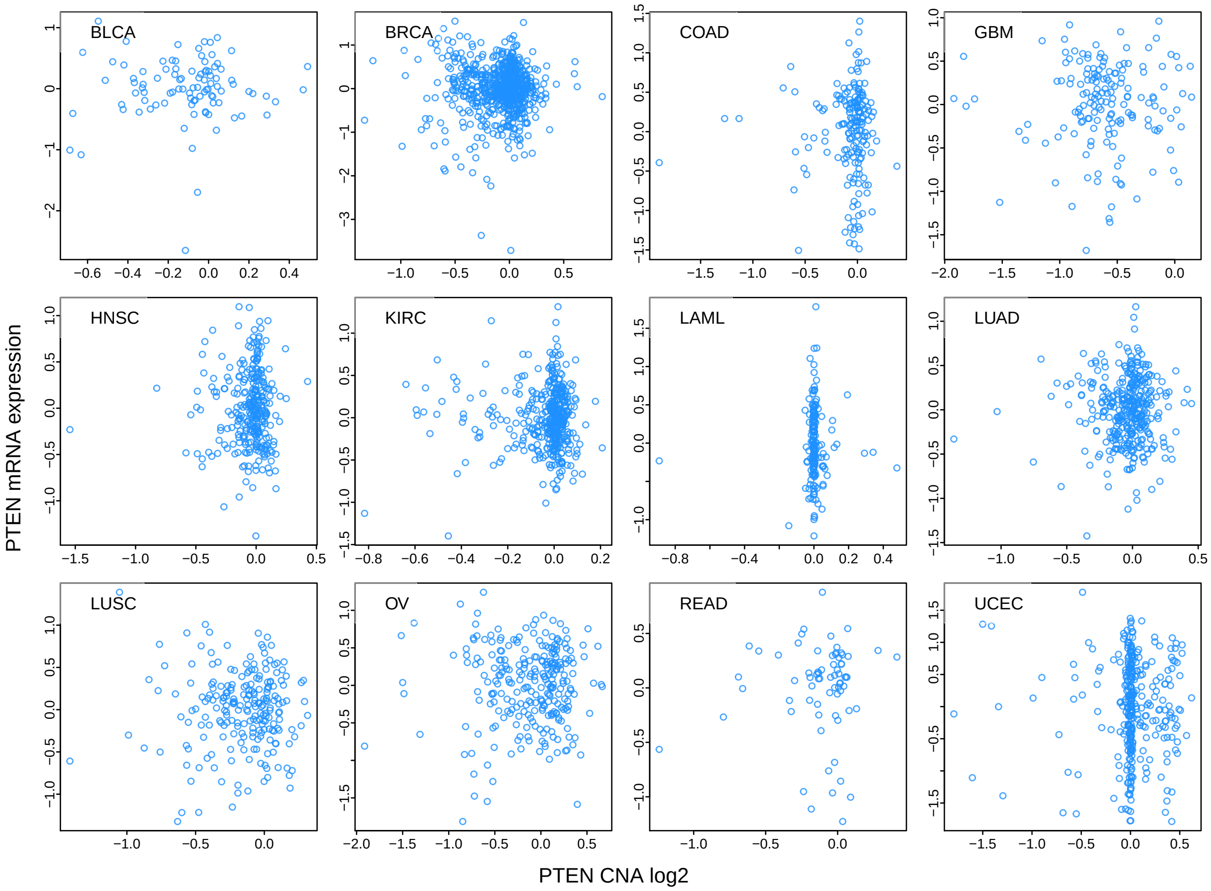


Supplementary Fig. 38: Scatter plots of *PTEN* copy number alterations and expression after cis-effect removing. The cis-effects of copy number alterations on gene expression have been removed by subtracting the regression values from the original expression values.

# Supplementary Tables

Supplementary Table 1: Representative methods predicting mutations impacting gene expression

| Method | Probabilistic generative models | Patient-specific | Predict dysregulated genes | CNAs | Mutations | Pathways | Reference |
|---|---|---|---|---|---|---|---|
| CONEXIC | Yes | No | Partial (no probability output) | Yes | No | No | 2 |
| EPoC | No | No | Partial (no probability output) | Yes | No | No | 3 |
| MOCA | No | No | Partial (no probability output) | No | Yes | No | 4 |
| DriverNet | No | No | Partial (no probability output) | Yes | Yes | Yes | 5 |
| PARADIGM | Yes | No | No (output pathway activities) | Yes | Yes | Yes | 6 |
| **xseq** | Yes | Yes | Yes | Yes | Yes | Yes | This paper |

Some methods are specifically designed to predict driver copy number alterations (CNAs), and others aim at predicting driver point mutations and indels (Mutations). Some methods predict driver genes and their target genes *de-novo* without using pathway information. Only `xseq` predicts individual mutations of a gene impacting expression, and outputs the posterior probability of a gene in a patient been up-regulated or down-regulated.

Supplementary Table 2: Description of the random variables and the conditional distributions in `xseq`

| Name | Description | Range |
|---|---|---|
| $D_g$ | Whether mutations in gene $g$ across patients impacting expression | Binary (0 means not impacting expression, and 1 means impacting expression) |
| $F_{g,m}$ | Whether gene $g$'s mutation specifically in patient $m$ impacting expression | Binary (0 means not impacting expression, and 1 means impacting expression) |
| $G_{g,m,n}$ | The $n$th gene connected to $g$ in patient $m$ is up-regulated, neutral, or down-regulated | Ternary ($\mathcal{L}$, $\mathcal{N}$ and $\mathcal{G}$ represent expression down-regulation, neutral and up-regulation, respectively) |
| $H_{g,n}$ | The $n$th gene connected to $g$ is up-regulated or down-regulated across the patients harbouring gene $g$ mutations which correlated with connected gene dysregulation | Binary ($\mathcal{L}$ and $\mathcal{G}$ mean down- and up-regulation of expression, respectively) |
| $Y_{g,m,n}$ | The expression of the $n$th gene connected to $g$ in patient $m$ | Real (observed) |
| $\theta_{D=1} \stackrel{\text{def}}{=} P(D=1)$ | Probability of gene $g$'s mutations impacting expression across patients | Real |
| $\theta_{F=1\|D=0} \stackrel{\text{def}}{=} P(F=1\|D=0)$ | Probability of gene $g$'s mutation impacting expression in patient $m$ given that gene $g$'s mutations do not impact expression across patients | Real |
| $\theta_{F=1\|D=1} \stackrel{\text{def}}{=} P(F=1\|D=1)$ | Probability of gene $g$'s mutation impacting expression in patient $m$ given that gene $g$'s mutations impact expression across patients | Real |
| $\theta_{G=\mathcal{L}\|F=0} \stackrel{\text{def}}{=} P(G=\mathcal{L}\|F=0)$ | Probability of a gene connected to $g$ is down-regulated in patient $m$ given that $g$'s mutation in $m$ do not impact expression | Real |
| $\theta_{G=\mathcal{N}\|F=0} \stackrel{\text{def}}{=} P(G=\mathcal{N}\|F=0)$ | Probability of a gene connected to $g$ is neutral in patient $m$ given that $g$'s mutation in $m$ do not impact expression | Real |
| $\theta_{G=\mathcal{L}\|F=1,H=0} \stackrel{\text{def}}{=} P(G=\mathcal{L}\|F=1,H=0)$ | Probability of a gene connected to $g$ is down-regulated in patient $m$ given that $g$'s mutation in $m$ impact expression and this gene is generally down-regulated when mutations in $g$ impact expression | Real |
| $\theta_{G=\mathcal{N}\|F=1,H=0} \stackrel{\text{def}}{=} P(G=\mathcal{N}\|F=1,H=0)$ | Probability of a gene connected to $g$ is neutral in patient $m$ given that $g$'s mutation in $m$ impact expression and this gene is generally down-regulated when mutations in $g$ impact expression | Real |
| $\theta_{G=\mathcal{L}\|F=1,H=1} \stackrel{\text{def}}{=} P(G=\mathcal{L}\|F=1,H=1)$ | Probability of a gene connected to $g$ is down-regulated in patient $m$ given that $g$'s mutation in $m$ impact expression and this gene is generally up-regulated when mutations in $g$ impact expression | Real |
| $\theta_{G=\mathcal{N}\|F=1,H=1} \stackrel{\text{def}}{=} P(G=\mathcal{N}\|F=1,H=1)$ | Probability of a gene connected to $g$ is neutral in patient $m$ given that $g$'s mutation in $m$ impacts expression and this gene is generally up-regulated when mutations in $g$ impact expression | Real |
| $p(Y=y\|G=\mathcal{L})$ | The likelihood of observing expression of $y$ in a gene given that this gene is down-regulated | Real |
| $p(Y=y\|G=\mathcal{N})$ | The likelihood of observing expression of $y$ in a gene given that this gene is neutral | Real |
| $p(Y=y\|G=\mathcal{G})$ | The likelihood of observing expression of $y$ in a gene given that this gene is up-regulated | Real |

When describing the conditional probability tables, $g$ refers to a mutated gene in general.

Supplementary Table 3: The validation rates across tumour types from repeated cross-validation experiments

| | READ | BLCA | GBM | COAD | OV | LAML | LUAD | LUSC | UCEC | HNSC | KIRC | BRCA |
|---|---|---|---|---|---|---|---|---|---|---|---|---|
| Median validation rates (only *bona fide* genes) | 0.00 | 0.50 | 0.50 | 0.37 | 0.58 | 0.67 | 0.40 | 0.67 | 0.67 | 0.67 | 0.67 | 0.73 |
| # predicted *bona fide* genes from the original analysis | 2.00 | 5.00 | 8.00 | 5.00 | 2.00 | 5.00 | 15.00 | 4.00 | 13.00 | 16.00 | 6.00 | 19.00 |
| Median # predicted *bona fide* genes from cross-validation | 0.50 | 2.00 | 3.00 | 3.00 | 2.00 | 3.00 | 4.50 | 3.00 | 8.00 | 5.00 | 3.50 | 12.00 |
| Validation rates (all genes) | 0.00 | 0.50 | 0.50 | 0.33 | 0.58 | 0.67 | 0.21 | 0.37 | 0.48 | 0.47 | 0.58 | 0.65 |
| # predicted genes from the original analysis | 2.00 | 8.00 | 8.00 | 12.00 | 3.00 | 5.00 | 25.00 | 12.00 | 30.00 | 29.00 | 8.00 | 54.00 |
| Median # predicted genes from cross-validation | 0.50 | 2.50 | 3.00 | 4.00 | 2.00 | 3.00 | 9.00 | 5.00 | 13.50 | 8.50 | 4.00 | 25.50 |

We also compare the cross-validation predictions with the predicted genes from the original analysis.

Supplementary Table 4: The validation rates from 10-repeated cross-validation experiments after filtering out rarely mutated genes

| | READ | BLCA | GBM | COAD | OV | LAML | LUAD | LUSC | UCEC | HNSC | KIRC | BRCA |
|---|---|---|---|---|---|---|---|---|---|---|---|---|
| Median validation rates (only *bona fide* genes) | 0.00 | 0.50 | 0.67 | 0.33 | 0.75 | 0.88 | 0.58 | 0.83 | 0.69 | 0.75 | 0.67 | 0.75 |
| # predicted *bona fide* genes from the original analysis | 2.00 | 5.00 | 8.00 | 5.00 | 2.00 | 5.00 | 15.00 | 4.00 | 13.00 | 16.00 | 6.00 | 19.00 |
| Median # predicted *bona fide* genes from cross-validation | 0.50 | 2.00 | 2.50 | 3.00 | 2.00 | 3.00 | 3.00 | 2.00 | 7.50 | 4.00 | 3.00 | 12.00 |
| Validation rates (all genes) | 0.00 | 0.50 | 0.67 | 0.50 | 0.58 | 0.88 | 0.40 | 0.67 | 0.60 | 0.68 | 0.67 | 0.71 |
| # predicted genes from the original analysis | 2.00 | 8.00 | 8.00 | 12.00 | 3.00 | 5.00 | 25.00 | 12.00 | 30.00 | 29.00 | 8.00 | 54.00 |
| Median # predicted genes from cross-validation | 0.50 | 2.00 | 2.50 | 3.50 | 2.00 | 3.00 | 5.00 | 3.00 | 10.00 | 5.50 | 3.00 | 23.50 |

In summering the results, we only keep the gene which have at least 5 mutations in both the discovery and validation datasets in repeated cross-validation analysis. We also compare the cross-validation predictions with the predicted genes from the original analysis.

Supplementary Table 5: The genes were predicted to have high probability in both TCGA and METABRIC (MT) breast cancer data

| Gene | Type | P(D)-TCGA | P(D)-MT | #CNAs-TCGA | #CNAs-MT | Frequency-TCGA | Frequency-MT |
|---|---|---|---|---|---|---|---|
| MYC | HLAMP | 1.00 | 1.00 | 193 | 71 | 0.22 | 0.07 |
| ERBB2 | HLAMP | 1.00 | 1.00 | 114 | 118 | 0.13 | 0.12 |
| CCNE1 | HLAMP | 1.00 | 1.00 | 33 | 13 | 0.04 | 0.01 |
| RB1 | HOMD | 1.00 | 1.00 | 26 | 16 | 0.03 | 0.02 |
| CCND1 | HLAMP | 1.00 | 1.00 | 145 | 100 | 0.16 | 0.10 |
| CBX8 | HLAMP | 1.00 | 1.00 | 57 | 16 | 0.06 | 0.02 |
| CDKN2A | HOMD | 1.00 | 1.00 | 33 | 16 | 0.04 | 0.02 |
| E2F3 | HLAMP | 1.00 | 1.00 | 21 | 4 | 0.02 | 0.00 |
| PTEN | HOMD | 1.00 | 1.00 | 32 | 16 | 0.04 | 0.02 |
| KRAS | HLAMP | 1.00 | 0.99 | 19 | 7 | 0.02 | 0.01 |
| ING1 | HLAMP | 1.00 | 0.98 | 24 | 3 | 0.03 | 0.00 |
| WNK1 | HLAMP | 1.00 | 0.98 | 36 | 11 | 0.04 | 0.01 |
| SMARCA2 | HLAMP | 1.00 | 0.98 | 22 | 5 | 0.03 | 0.01 |
| PPP2R2A | HOMD | 1.00 | 1.00 | 49 | 34 | 0.06 | 0.03 |

Here only copy number alteration were reported. The copy number calls for METABRIC data were from a hidden Markov model based approach HMM-dosage[7,8], instead of GISTIC[9] used for the TCGA data. HMM-dosage is more conservative to call copy number homozygous deletions and amplifications based on the mutation frequency. The sensitivity issue of HMM-dosage partially explains why some genes were not predicted to have high probability in the METABRIC data simply because of lacking copy number calls.

## Supplementary Table 6: Comparison with CONEXIC

| | # input genes | # predicted genes | # *bona fide* driver genes | # direct partners of *bona fide* driver genes |
|---|---|---|---|---|
| CONEXIC | 3044 | 313 | 21 | 46/292 |
| `xseq` | 3044 | 40 | 13 | 13/27 |
| CONEXIC | 131 | 72 | 27 | 12/45 |
| `xseq` | 131(3044) | 20 | 11 | 5/9 |

Comparison with CONEXIC in predicting genes whose copy number alterations (homozygous deletions and amplifications) influence gene expression in the TCGA breast cancer datasets.

## Supplementary Table 7: Different versions of `xseq` predicted high probability genes

| | # `xseq` predicted genes ($P(D) \geq 0.8$) | # *bona fide* driver genes | # direct partners of *bona fide* driver genes (of the novel `xseq` predictions) |
|---|---|---|---|
| `xseq` | 150 | 60/150 | 29/89 |
| `xseq-simple` | 106 | 46/106 | 19/59 |

Different versions of `xseq` predicted high probability genes and their overlap with *bona fide* driver genes and direct connected partners of *bona fide* driver genes. Here gene *ACVR2A* was predicted by both `xseq` and MuSiC but was not in the *bona fide* cancer gene list. Therefore *ACVR2A* was considered as neither a *bona fide* gene nor a novel gene.

Supplementary Table 8: The genes harbouring mutations whose `xseq` probabilities showed bimodal distributions.

| | Gene | Tumour | # P(F)>=0.5 | # P(F)<0.5 | FDR |
|---|---|---|---|---|---|
| 1 | RB1 | BLCA | 12 | 9 | 0.00 |
| 2 | NFE2L2 | BLCA | 7 | 3 | 0.20 |
| 3 | GATA3 | BRCA | 31 | 46 | 0.00 |
| 4 | TP53 | BRCA | 163 | 87 | 0.00 |
| 5 | CDKN2A | BRCA | 20 | 12 | 0.00 |
| 6 | RB1 | BRCA | 30 | 10 | 0.00 |
| 7 | CCND1 | BRCA | 42 | 93 | 0.00 |
| 8 | PTEN | BRCA | 27 | 32 | 0.00 |
| 9 | CDH1 | BRCA | 29 | 27 | 0.00 |
| 10 | MAP3K1 | BRCA | 19 | 39 | 0.00 |
| 11 | BRCA1 | BRCA | 5 | 8 | 0.04 |
| 12 | KRAS | BRCA | 14 | 8 | 0.13 |
| 13 | AKT1 | BRCA | 14 | 15 | 0.13 |
| 14 | MAP2K4 | BRCA | 16 | 15 | 0.17 |
| 15 | ACVR2A | COAD | 17 | 6 | 0.19 |
| 16 | TP53 | GBM | 29 | 20 | 0.00 |
| 17 | EGFR | GBM | 25 | 58 | 0.00 |
| 18 | RB1 | GBM | 7 | 6 | 0.01 |
| 19 | NFE2L2 | HNSC | 25 | 8 | 0.00 |
| 20 | NOTCH1 | HNSC | 29 | 32 | 0.00 |
| 21 | CCND1 | HNSC | 20 | 59 | 0.00 |
| 22 | RB1 | HNSC | 10 | 5 | 0.06 |
| 23 | KEAP1 | HNSC | 11 | 4 | 0.12 |
| 24 | BAP1 | KIRC | 24 | 19 | 0.00 |
| 25 | SETD2 | KIRC | 27 | 19 | 0.00 |
| 26 | NFE2L2 | KIRC | 4 | 4 | 0.01 |
| 27 | TP53 | LUAD | 31 | 56 | 0.00 |
| 28 | CDKN2A | LUAD | 29 | 48 | 0.00 |
| 29 | RB1 | LUAD | 8 | 5 | 0.01 |
| 30 | CCND1 | LUAD | 10 | 9 | 0.07 |
| 31 | NFE2L2 | LUAD | 5 | 3 | 0.18 |
| 32 | RB1 | LUSC | 10 | 8 | 0.00 |
| 33 | NFE2L2 | LUSC | 26 | 8 | 0.09 |
| 34 | RB1 | OV | 14 | 7 | 0.00 |
| 35 | TP53 | UCEC | 49 | 21 | 0.00 |
| 36 | RB1 | UCEC | 12 | 12 | 0.00 |
| 37 | CTNNB1 | UCEC | 53 | 19 | 0.00 |
| 38 | NFE2L2 | UCEC | 8 | 7 | 0.00 |
| 39 | POLQ | UCEC | 9 | 12 | 0.00 |
| 40 | CHEK2 | UCEC | 7 | 5 | 0.01 |

These genes were predicted to be significantly mutated by MuSiC analysis. The dip-test was used to test for unimodality of `xseq` $P(F)$ distribution of a gene in a specific tumour type, and the FDR was the adjusted dip-test p-values based on the Benjamini & Hochberg procedule.

# 1 Supplementary Discussions

## 1.1 Additional 17 genes with cis-effect loss-of-function mutations

Three novel predicted genes (*AMOT, AMOTL1*, and *ITCH*) encode proteins of the Hippo signalling pathway, involved in restraining cell division and promoting apoptosis. *AMOT* and *AMOTL1* are the angiomotin family proteins. The *AMOT* family proteins physically interact with and inhibit *YAP/TAZ*, a transcription co-activator which plays a crucial role in organ size control by promoting cell proliferation and inhibiting apoptosis[10]. In addition, the *AMOT* family proteins can activate *LATS2*, a protein kinase which belongs to the *LATS* tumor suppressor family[11]. Note that *AMOT* encodes two proteins isoforms, and *AMOT*-p80 positively regulates MARK signalling pathway[12]. *ITCH* is an anti-proliferation protein[13]. *ITCH* ubiquitinates c-FLIP, an inhibitor of tumour suppressor gene caspase-8, and induces its proteasomal degradation[13].

Three genes (*CTNND1, CELSR2*, and *PARD3*) encode adhesion proteins, which play roles in metastasis. *CTNND1* (cadherin-associated Src substrate) deletions in mice lead to tumor development[14]. It has been shown to be down-regulated in several cancer types[15]. Both *CTNND1* and *CELSR2* (cadherin, EGF LAG seven-pass G-type receptor 2) are part of the Wnt signaling pathway. It has been shown that *CELSR2* was down-regulated in breast cancer cell lines and tumors. It is postulated that *CELSR2* is a receptor and involved in cell adhesion and receptor-ligand interactions[16]. *PARD3* (Par-3 Family Cell Polarity Regulator) is in the Signalling by TGF-beta Receptor Complex(R) pathway. This gene is deleted in both cell lines and primary tumors from squamous carcinomas and glioblastomas[17], and reconstituting *PARD3* expression restores tight conjunction and retards contact-dependent proliferation[17].

Five genes (*MED23, RB1CC1, YLPM1, EIF2C1*, and *MAZ*) encode proteins that regulate transcription. *MED23* encodes a protein that is a subunit of the Mediator complex, a co-activator involved in the regulated transcription of nearly all RNA polymerase II-dependent genes. *MED23* is down-regulated in esophageal squamous cell carcinoma[18], and its expression is negatively correlated with tumour size and clinical stages. Moreover, *MED23* up-regulation significantly inhibits cell growth, and down-regulation promotes tumorigenecity in vitro and in vivo[18]. *RB1CC1* is recurrently mutated in endometrial cancer and high-grade serous ovarian cancer. *RB1CC1* encodes a protein that regulates *RB1* expression[19], and positively regulates transforming growth gactor-$\beta$ signaling[20]. *YLPM1* plays roles in telomerase activity during differentiation of embryonic stem cells. Genomic copy number deletions of *YLPML* are associated with poor prognosis in colorectal cancer[21]. *EIF2C1* (*AGO1*, Argonaute RISC Catalytic Component) plays a role in RNA interference, and when over-expressed, slows down cell cycle, decreases cellular motility, and promotes apoptosis in neuroblastoma cell lines[22]. However, the functions of this gene may be cell type dependent because knockdown of *EIF2C1* by siRNA also inhibites cell cycle progression in prostate cell lines[23]. *MYC*-accociated zinc finger protein (*MAZ*) is a transcription factor, which

suppresses the expression of c-myc oncogene in COS cells and actives the expression of tissue specific genes[24]. This protein is a growth suppressor and suppresses the G1 phase of the cell cycle[25]. Down-regulation of *MAZ* results in reduced expression and promoter methylation of the tumour suppressor gene *MLH1*[26]. In contrast to many tumour suppressor genes, this gene is amplified and up-regulated in many cancer types.

*SEC63, ATF6*, and *UBQLN1* are involved in protein processing in the endoplasmic reticulum. *SEC63* encodes a protein that is associated with ribosome-free *SEC61* complex. Down-regulation of *SEC63* correlates significantly with decreased apoptosis and increased proliferation following exposure to diethylnitrosamine[27]. *ATF6* (Activating transcription factor 6) regulates apoptosis in its active form[28]. As circumventing apoptosis is a hallmark of cancer, the gene is likely to be a novel tumour suppressor gene. The roles of *UBQLN1* in cancer have been identified very recently[29]. Loss of *UBQLN1* causes increased cell migration and invasion, actin cytoskeleton reorganization, and induction of epithelial-tomesenchymal transition[29]. Loss of *UBQLN1* results in a significant decrease of E-cadherin gene expression. It is worth noticing that *UBQLN1* and *RIC8A* (below) are in the same protein complex.

*RIC8A* is a non-receptor guanine nucleotide exchange factor, and regulates mitotic cell division and spindle alignment in asymmetric cell division in model organisms and mammalian cells. *RIC8A* regulates the G-protein Regulatory-G$\alpha$i Signaling Complex, which inhibits the production of ATP derived cAMP[30]. *RIC8A* stabilizes G$\alpha$ subunits by binding to them and, in the absence of *RIC8A*, G$\alpha$ subunits are not capable of binding to GTP[31]. A nonsense mutation of *RIC8A* was observed in an aggressive breast cancer cell line and this mutation correlated with *RIC8A* lower expression[32], consistent with our predictions.

*ATP11C* (ATPase, Class VI, Type 11C) is less studied. It has recently been shown that a splice-site mutation in *ATP11C* disrupted B-cell development and antibody production, and resulted in hepatocellular carcinoma[33]. As this gene is located on the X-chromosome, it may play a role in inherited B cell deficiency and hepatocellular carcinoma in men. Very recently, this gene was found to play a role in apoptosis[34]; mutation in *ATP11C* can prevent cells from being phagocytosed by macrophages during apoptosis.

*MAGEA4* (Melanoma Antigen Family A, 4) shows both oncogene and tumour suppressor gene activities[35,36]. *MAGEA4* protein binds to gankyrin, an oncogene over-expressed in hepatocellular carcinomas, and partially suppresses both anchorage-independent growth in vitro, and tumour formation in athymic nude mice with stable gankyrin over-expression[37]. *MAGEA4* also regulates apoptosis in both lung squamous cancer cell lines and kidney cell lines[35].

## 1.2 Additional 26 novel genes with trans-effect mutations

Six genes play roles in cell proliferation regulation – *VEGFA, VEGFC, IRS1, INSR, E2F3*, and *BCL2L1* (Supplementary Data 5). *VEGFA* (vascular endothelial growth factor A) and *VEGFC* (vascular endothelial growth factor C) has many roles in cancer, in angiogenesis, vascular permeability, and in immune cells presented in the tumour environment. *IRS1* (nsulin Receptor Substrate 1) deficiency promotes apoptosis and inhibits the growth of breast cancer cells. However, it has also been demonstrated that suppression of *IRS1* promotes mammary tumour metastasis. Insulin receptor (*INSR*) play a key role in glucose homeostasis regulation, whose under degenerate conditions may cases diabetes and cancer. The E2F transcription factors regulate various biological processes, e.g., cell cycle regulation, DNA repair, apoptosis, centrosome duplication and differentiation[38]. Specifically, *E2F3* down-regulation resultes in cell death and delays/blocks in cytokinesis[38], consistent with *E2F3* amplification in various tumour types. *BCL2L1* (*BCL2*-Like 1) is in chromosome 20 and amplified in various tumours. It has been shown that of the 3 genes in a amplicon over expressed in human embryonic stem cells, only over expression of *BCL2L1* provides control cells with growth characteristics similar to *BCL2L1* copy number amplified cells[39]. In addition, inhibition of *BCL2L1* protein expression results in strong reduction the growth rate of human embryonic stem cells[39].

Twelve genes encode proteins that play roles in apoptotic process (*PSMD1, PSMD13, ERN1, MCL1, PRKCQ, TJP1, MELK, MNDA, NET1, PHB, SULF1*, and *BCL2L1*). *PSMD1* and *PSMD13* are members of the 26S proteasome non-ATPase regulatory subunit complex, and inhibition of the 26S proteasome induces apoptosis, and limits growth of human pancreatic cancer[40]. Suppression of *ERN1* leads to reduced tumour growth through inhibition of angiogenesis and proliferation[41]. Myeloid cell leukemia 1 (*MCL1*) is amplified and over-expressed in many tumour types. Moreover, over expression of *MCL1* is the resistance factor for several *BCL2* family inhibitors[42]. It has been shown that *MCL1* inhibitor has sufficient potency to kill a variety of cancer cells[42]. *PRKCQ* (Protein Kinase C, Theta) is required for the growth of triple negative breast cancer cells[43]. Even over-expression of *PRKCQ* is sufficient to drive oncogenic, growth factor-independent growth, survival and migration[43]. Down-regulation of *TJP1* (tight junction protein 1) by its direct regulator miR-105 destroys vascular endothelial barriers to promote metastasis in cancer cells[44]. *MELK* is an oncogenic kinase essential for mitotic progression in basal-like breast cancer cells[45]. *NET1* is a RhoA guanine exchange factor. *NET1* up-regulation in gastric cancer drives the invasive phenotype[46]. *PHB* acts as a tumour suppressor and AR co repressor in prostate cancer[47]. *SULF1* has been shown to promote tumorigenicity in ovarian cancer[48].

Fifteen genes play roles in transcription regulation (*ZNF217, SMARCA2, CBX8, MAF1, BZW1, ZBTB7B, EEF1A1, MSL3, PHB, MNDA, E2F3, PCGF3, ERN1, PRKCQ*, and *INSR*). *ZNF217* (Zinc Finger Protein 217) is a candidate breast cancer driver gene, and *ZNF217*-transduced cultures produce immortalized cells[49]. *SMARCA2* encodes the core catalytic unit of the SWI/SNF ATP-dependent chromatin remodelling complex. *CBX8* (chromodomain-containing protein 8) is

an essential component of the polycomb repressive complexes 1, which directly regulates numerous target gene expression involved in cell-fate decisions[50]. *MAF1* homolog (S. cerevisiae) is a down stream effector of the PTEN/PI3K signalling pathway and it plays a key role in this pathway to regulate lipid metabolism and oncogenesis[51]. *BZW1* has been shown to promote growth of salivary muocepodermoid carcinoma[52]. *ZBTB7B* up-regulation contributes to breast cancer development[53]. It may be a cell-cycle modulator by regulating *CDK2* and *E2F4*[54]. *EEF1A1* is a substrate of the Type I TGF-$\beta$ receptor[55], and mutations of Ser300 affect TGF-$\beta$ dependency in inhibition of protein synthesis and cell proliferation[55].

# 2 Supplemental Methods

## 2.1 Inference and learning in `xseq`

Pearl's belief propagation algorithm (polytree algorithm) was first proposed by Judea Pearl in 1982[56] to solve inference problems for tree structure Bayesian networks (There is only one undirected path between any two nodes. In addition, a child node has only one parent). It was then extend to do inference for polytrees[57] (a child node can have multiple 'parents'). Notice that `xseq-simple` is a tree, while `xseq` is not a tree or a polytree, e.g., for the `xseq` model in Fig. 1(a), there is a undirected loop $D \rightarrow F_1 \rightarrow G_{11} \rightarrow H_1 \rightarrow G_{21} \rightarrow F_2 \rightarrow D$. Therefore, generally speaking inference in `xseq` is difficult, i.e., the time and memory complexity is exponential in the number of mutations in $g$. However, if $H$ is given, `xseq` is equivalent to a polytree because we can "break" $H$ to convert the toy `xseq` model in Supplementary Fig. 1(a) to a polytree as given in Supplementary Fig. 1(b).

In addition to simplify the inference problem, there are practical reasons to let $H$ as observed variables. First, from pathway information, we may know that gene $g$ up-regulates gene $a$. The directionality of gene regulation information can be added to the `xseq` model as a observed node $H$. Then we can use `xseq` to search the mutations in gene $g$ that correlate with gene $a$ up-regulation. Secondly, if we observe that gene $g$ up-regulates gene $a$ in a discovery dataset, given a validation dataset, we also want to inspect whether the mutations in gene $g$ up-regulate gene $a$.

The belief propagation algorithm computes the posterior marginal probabilities $P(D_g)$, $P(F_{m,n})$ and $P(G_{g,m,n})$ in two phases of message passing: 1) A leaf node (An observed gene expression value node $Y_{g,m,n}$) sends to its parent $G_{g,m,n}$ a message, namely a probability distribution $P(Y_{g,m,n} \mid G_{g,m,n})$ over parent $G_{g,m,n}$. After receiving all the messages from its children, a node can construct and send messages to its parents. Again, a message sent to a parent is a distribution over the parent, e.g., the message $G_{g,m,n}$ sent to $F_{g,m}$ is a distribution over $F_{g,m}$. The detailed definitions of these messages are described below. This process is repeated until the roots of the tree - the nodes without any parents receive all the messages from their children. 2) A root node can update its posterior distribution by normalizing the product of all the incoming messages and its own prior distribution. Then a root node can construct and send messages down to its children. A top-down message is also a distribution of the parent, e.g., the message $D_g$ sent to $F_{g,m}$ is the posterior distribution $P(D_g)$ divided by the message $F_{g,m}$ sent to $D_g$ before. This process is again repeated until the leaf nodes have received all the messages from their parents. After receiving all the incoming messages from its neighbours (both parents and children), a node can update its belief – the posterior probabilities.

### 2.1.1 Details of the message passing steps in xseq

To simplify our description of the inference algorithm, we use a simple example with just one mutated gene, and $H$ is given. In addition, this gene is mutated in $M$ patients and this gene is connected to $N$ genes. Formally, at the first phase, message collection phase, the bottom-up messages (with superscript $(-)$) consist of (in sequential order):

$$m^{(-)}_{Y_{m,n}\to G_{m,n}} = p(Y_{m,n} \mid G_{m,n}) \tag{1}$$

$$m^{(-)}_{G_{m,n}\to F_m} = \sum_{G_{m,n}} \theta_{G_{m,n}|F_m,H_n} \mathrm{bel}^{(-)}_{G_{m,n}} \tag{2}$$

$$m^{(-)}_{F_m\to D} = \sum_{F_m} \theta_{F_m|D} \mathrm{bel}^{(-)}_{F_m} \tag{3}$$

where $\mathrm{bel}^{(-)}_{G_{m,n}}$ and $\mathrm{bel}^{(-)}_{F_m}$ are the bottom-up belief states of $G_{m,n}$ and $F_m$, respectively. The bottom-up belief of a node is updated after the node receives all the bottom-up messages from its children, for example,

$$\mathrm{bel}^{(-)}_{G_{m,n}} \propto m^{(-)}_{Y_{m,n}\to G_{m,n}} \tag{4}$$

$$\mathrm{bel}^{(-)}_{F_m} \propto \prod_{n=1}^{N} m^{(-)}_{G_{m,n}\to F_m} \tag{5}$$

Once the root $D$ receives all the incoming messages, it can update its belief by normalizing the product of these messages:

$$\mathrm{bel}_D = P(D|Y) \propto \theta_D \prod_m m^{(-)}_{F_m\to D} \tag{6}$$

Then $D$ sends top-down messages to its children (with superscript (+)):

$$m^{(+)}_{D\to F_m} \propto \frac{\mathrm{bel}_D}{m^{(-)}_{F_m\to D}} \tag{7}$$

$$m^{(+)}_{F_m\to G_{m,n}} \propto \frac{\mathrm{bel}_{F_m}}{m^{(-)}_{G_{m,n}\to F_m}} \tag{8}$$

Here the 'division' operator for distributions is only defined for two distributions of the same set of random variables, and it is defined as element-wise divisions. The marginal posterior of $F_m$ and

$G_{m,n}$ can be updated by

$$\mathrm{bel}_{F_m} = P(F_m|Y) \propto \sum_D \theta_{F_m|D} * m^{(+)}_{D\to F_m} \prod_n m^{(-)}_{G_{m,n}\to F_m} \tag{9}$$

$$\mathrm{bel}_{G_{m,n}} = P(G_{m,n}|Y) \propto \sum_{F_m} \theta_{G_{m,n}|F_m,H_n} * m^{(+)}_{F_m\to G_{m,n}} m^{(-)}_{Y_{m,n}\to G_{m,n}} \tag{10}$$

### 2.1.2 Details of parameter learning

Assume that we have the mutation data for $T$ genes, and gene $g$ is mutated in $M_g$ patients, and gene $g$ connects to $N_g$ genes. We first introduce some variables to make the presentation easier. We define $\theta_{D_i} = \theta_{D=i}$, similarly, $\theta_{F_j|D_i} = \theta_{F=j|D=i}$, and $\theta_{G_k|F_j,H_l} = \theta_{G=k|F=j,H=l}$. Notice that we assume these parameters are the same for different genes. This assumption is necessary otherwise we do not have enough data to robustly estimate model parameters. Then we define hidden variable $Z^g_{D_i} = 1$ if and only if $D_g = i$. Similarly, indicator variable $Z^{g,m}_{F_j|D_i} = 1$ if and only if $F_{g,m} = j$ and $D_g = i$. Indicator variable $Z^{g,m,n}_{G_k|F_j,H_l} = 1$ if and only if $G_{g,m,n} = k$, $F_{g,m} = j$ and $H_{g,n} = l$. Finally, we use $D_g = 1$ to denote that the mutations in $g$ impact expression, and $D_g = 0$ means not; $F_{g,m} = 1$ means that the mutation in gene $g$ of patient $m$ impacting expression, and $F_{g,m}$ means not; $H_{g,n} = \mathcal{L}$ means that the $n$th gene connected to gene $g$ is down-regulated across patients, and $H_{g,n} = \mathcal{G}$ means that the $n$th gene connected to gene $g$ is up-regulated across patients. Similarly, $G_{g,m,n} \in \mathcal{L}, \mathcal{N}, \mathcal{G}$ , where $\mathcal{L}, \mathcal{N}, \mathcal{G}$ represents down-regulation, neutral and up-regulation of expression, respectively.

For a give dataset $\mathcal{D}$ consisting of mutations, expression and gene interaction networks, the complete log-likelihood can be computed by

$$\log p(\mathcal{D} \mid \theta) = \sum_{g=1}^{T}\sum_{m=1}^{M_g}\sum_{n=1}^{N_{m,g}}\sum_{i=0}^{1} \log\left(\theta_{D_i}^{Z^g_{D_i}}\right) \sum_{j=0}^{1} \log\left(\theta_{F_j|D_i}^{Z^{g,m}_{F_j|D_i}}\right) \sum_{k\in\mathcal{L},\mathcal{N},\mathcal{G}} \left(\log\left(\theta_{G_k|F_j,H_l}^{Z^{g,m,n}_{G_k|F_j,H_l}}\right) + \log(p(Y_{g,m,n} \mid G_{g,m,n}))\right) \tag{11}$$

We then take the expectation of the complete log-likelihood function to get

$$Q(\theta, \theta^{(old)}) = \mathbb{E}\left(\sum_{g=1}^{T}\sum_{m=1}^{M_g}\sum_{n=1}^{N_{m,g}}\sum_{i=0}^{1} \log\left(\theta_{D_i}^{Z^g_{D_i}}\right) \sum_{j=0}^{1} \log\left(\theta_{F_j|D_i}^{Z^{g,m}_{F_j|D_i}}\right) \sum_{k=\in\mathcal{L},\mathcal{N},\mathcal{G}} \left(\log\left(\theta_{G_k|F_j,H_l}^{Z^{g,m,n}_{G_k|F_j,H_l}}\right) + \log(p(Y_{g,m,n} \mid G_{g,m,n}))\right)\right)$$

$$\propto \sum_{i=0}^{1} \hat{N}_{Z_{D_i}} \log(\theta_{D_i}) \sum_{j=0}^{1} \hat{N}_{Z_{F_j|D_i}} \log(\theta_{F_j|D_i}) \sum_{k\in\mathcal{L},\mathcal{N},\mathcal{G}} \hat{N}_{Z_{G_k|F_j}} \log(\theta_{G_k|F_j,H_l}) \tag{12}$$

where

$$\hat{N}_{Z_{D_i}} = \sum_{g=1}^{T} P(D_g = i|\mathcal{D}) \tag{13}$$

$$\hat{N}_{Z_{F_j|D_i}} = \sum_{g=1}^{T} \sum_{m=1}^{M_g} P(F_{g,m} = j, D_g = i|\mathcal{D}) \tag{14}$$

$$\hat{N}_{Z_{G_k|F_j,H_l}} = \sum_{g=1}^{T} \sum_{m=1}^{M_g} \sum_{n=1}^{N_{m,g}} P(G_{g,m,n} = k, F_{g,m} = j, H_{g,n} = l|\mathcal{D}) \tag{15}$$

These terms can be computed by using the belief propagation algorithm introduced in the previous section. The complete data log-likelihood expectation function in Equation 12 can be maximized by solving a constraint optimization problem to get the maximization steps:

$$\hat{\theta}_{D_i} = \frac{\hat{N}_{Z_{D_i}}}{\sum_i \hat{N}_{Z_{D_i}}} \tag{16}$$

$$\hat{\theta}_{F_j|D_i} = \frac{\hat{N}_{Z_{F_j|D_i}}}{\sum_j \hat{N}_{Z_{F_j|D_i}}} \tag{17}$$

$$\hat{\theta}_{G_k|F_j=0} = \frac{\sum_l \hat{N}_{Z_{G_k|F_j=0,H_l}}}{\sum_k \sum_l \hat{N}_{Z_{G_k|F_j=0,H_l}}} \tag{18}$$

$$\hat{\theta}_{G_k|F_j=1,H_l} = \frac{\hat{N}_{Z_{G_k|F_j=1,H_l}}}{\sum_k \hat{N}_{Z_{G_k|F_j=1,H_l}}} \tag{19}$$

Seudocounts can be added to the above maximization steps to do maximum a posteriori estimation of parameters.

### 2.1.3 Adding constraints in learning `xseq` parameters

Parameter learning is a challenge problem in using Bayesian networks to solve real world problems. When the training data are incomplete or the Bayesian networks have multiple hidden nodes, parameter learning is extremely difficult, and learning algorithms can easily get trapped into local maxima. One solution to mitigate these local maximum problems is to add prior distributions for parameters and use maximum a posterior estimation of parameters. However, we still need to set the hyper-parameters of the prior distributions. These parameters may be data dependent and difficult to set. Here in addition to selecting good hyper-parameters for the prior distributions, we also constrain the parameter space in learning `xseq` conditional distributions. More specifically, in

trans analysis, we require the parameters of the `xseq-simple` model:

$$\theta_{G=\mathcal{L}|F} = \theta_{G=\mathcal{G}|F} \tag{20}$$

These constraints indicate that the genes connected to a mutated gene $g$ are equally likely to be up-regulated or down-regulated, given the $F$ status of the mutation in $g$ of a patient. For `xseq` model, we require the flowing constraints in analyzing the TCGA data:

$$\theta_{G=\mathcal{L}|F=1,H=\mathcal{L}} = \theta_{G=\mathcal{G}|F=1,H=\mathcal{G}} \tag{21}$$

$$\theta_{G=\mathcal{G}|F=1,H=\mathcal{L}} = \theta_{G=\mathcal{L}|F=1,H=\mathcal{G}} \tag{22}$$

### 2.1.4 Incorporating network connection weight in `xseq` inference

Our inference graph is a directed weighted graph. We can easily incorporate the weight between two genes/proteins into `xseq` inference. As before, we consider a simple case with just one mutated gene $g$. The posterior marginal of $D$ can be written as:

$$\begin{aligned}
&\sum_{F,G} P(D, F_1, \ldots, F_M, H_1, \ldots, H_N, G_{1,1}, \ldots, G_{1,N}, \ldots, \\
&G_{M,1}, \ldots, G_{M,N}, y_{1,1}, \ldots, y_{1,N}, \ldots, y_{M,1}, \ldots, y_{M,N}) \\
=&\sum_{F,G} \theta_D \prod_{m=1}^{M} \theta_{F_m|D} \prod_{n=1}^{N} \theta_{G_{m,n}|F_m=1,H_n}^{\mathbb{I}(F_m=1)} \theta_{G_{m,n}|F_m=0}^{\mathbb{I}(F_m=0)} p(Y_{m,n} \mid G_{m,n})
\end{aligned} \tag{23}$$

where $\mathbb{I}(\cdot)$ is the indicator function, and $\mathbb{I}(x) = 1$ when $x$ = TRUE, otherwise $\mathbb{I}(x) = 0$. The posterior marginal $P(D)$ of observing expression $y_{m,n}, m \in 1, \ldots, M, n \in 1, \ldots, N$ are determined by each factor (or "bucket"), e.g., $p(y_{m,n} \mid G_{m,n})$. Each factor can be considered as a feature, and the features which most relevant should play a larger role in computing the posterior marginal probabilities. Assuming the connection strength between gene $g$ and the $n$th gene connected to $g$ is $w_{g,n}$, then we can replace the factor $p(y_{m,n} \mid G_{m,n})$ by $p(y_{m,n} \mid G_{m,n})^{w_{g,n}}$ in Supplementary Equation 23.

Let's see how the weight influences predictions. When $w_{g,n} = 0$, the factor $p(y_{m,n} \mid G_{m,n})^{w_{g,n}}$ has no influence on the posterior marginal. When $0 < w_{g,n} < 1$, the relative values among $p(y_{m,n} \mid G_{m,n} = \mathcal{G})$, $p(y_{m,n} \mid G_{m,n} = \mathcal{N})$, and $p(y_{m,n} \mid G_{m,n} = \mathcal{L})$ will decrease after weighting, for example,

$$\frac{p(y_{m,n} \mid G_{m,n} = \mathcal{L})}{\sum_k p(y_{m,n} \mid G_{m,n} = k)} \leq \left( \frac{p(y_{m,n} \mid G_{m,n} = \mathcal{L})}{\sum_k p(y_{m,n} \mid G_{m,n} = k)} \right)^{w_{g,n}} \leq 1 \tag{24}$$

As the posterior marginal $P(D)$ should be normalized so they sum to 1: $\sum P(D) = 1$, the posterior marginal is only sensitive to the relative magnitude among $p(y_{m,n} \mid G_{m,n} \in \{\mathcal{L}, \mathcal{N}, \mathcal{G}\})$. That means,

the more discriminative of $p(y_{m,n} \mid G_{m,n} \in \{\mathcal{L}, \mathcal{N}, \mathcal{G}\})$, the more important of the $n$th gene in predicting the posterior marginal $P(D)$. Therefore, a small weight for $w_{g,n}$ means that the $n$th gene is less important.

## 2.2 Details of simulation study

We used `xseq` (Supplementary Fig. 2) to generate synthetic data. The number of mutated genes $T$ was fixed to 300, and the number of patients was fixed to 200. The number of accumulated mutations for gene $g$ was sampled from a shifted geometric distributions: $P(M_g = m) = 0.1^{m-15} \times 0.9$ with $m \geq 15$. The number of genes connected to $g$ was also sampled from a shifted geometric distribution: $P(N_g = n) = 0.1^{n-10} \times 0.9$ with $n \geq 10$. The hyper-parameters of the beta distribution for $\theta_D$ were set to $(70, 30)$, which means that around 30% of the genes accumulated mutations impacting expression. The hype-parameters of the beta distribution for $\theta_{F|D=0}$ were set to $(105, 5)$, and $(10, 100)$ for $\theta_{F|D=1}$. The Dirichlet hyper-parameters for $\theta_{G|F=0}$ were set to $(10, 180, 10)$. The gene expression data were sampled from Gaussian distributions. The variances of the Gaussian distributions were sampled from an inverse-gamma distribution: $\mathrm{IG}(\sigma^2 \mid \nu, \sigma_0^2)$. We fixed the hyper-parameter $\nu = 3$, $\sigma^2 = 0.12$.

We first analyzed the influence of the degree of gene regulation (up- or down-regulation) correlated with mutations. For high degree of gene regulation correlated with mutations, we set the Dirichlet parameters to $(144, 55, 1)$ for $\theta_{G|F=1,H=\mathcal{L}}$. For $\theta_{G|F=1,H=\mathcal{G}}$, the Dirichlet parameters were set to $(1, 55, 144)$ (Supplementary Fig. 3, column 1, here the $H$ was set to the true values). For moderate gene regulation correlated with mutations, we set the Dirichlet parameters to $(94, 105, 1)$ for $\theta_{G|F=1,H=\mathcal{L}}$. For $\theta_{G|F=1,H=\mathcal{G}}$, the Dirichlet parameters were set to $(1, 105, 94)$ (Supplementary Fig. 3, column 2). Finally, for low level gene regulation correlated with mutations, we set the Dirichlet parameters to $(44, 155, 1)$ for $\theta_{G|F=1,H=\mathcal{L}}$. For $\theta_{G|F=1,H=\mathcal{G}}$, the Dirichlet parameters were set to $(1, 155, 44)$ (Supplementary Fig. 3, column 3). As expected, as we decreased the degree of gene regulation correlated with mutations, the `xseq` prediction performance declined, especially in recovering the specific mutation variables $F$ (the ROC curves in Supplementary Fig. 3)

We then analyzed the influence of the discrimination of expression of down-regulation, neutral, and up-regulation on predictions. For high discriminative down-regulation, neutral and up-regulation expression distributions, we set the mean parameters of Gaussian distributions to (-2, 0, 2) (Supplementary Fig. 3, the first row). For moderate discriminative down-regulation, neutral and up-regulation expression distributions, we set the mean parameters of Gaussian distributions to (-1, 0, 1) (Supplementary Fig. 3, the second row). For poor discriminative down-regulation, neutral and up-regulation, we set the mean parameters of Gaussian distributions to (-0.5, 0, 0.5) (Supplementary Fig. 3, the third row). The decreasing in the discrimination of down-regulation, neutral and up-regulation expression distributions mostly declined the performance of recovering the gene regulation variables $G$.

We next used the xseq-simple model to analyze the simulation datasets (Supplementary Fig. 4). Generally speaking, the xseq-simple performed quite well. Compared to xseq predictions given the true values of $H$ variables, we could see the drop in performance, especially for the most challenging case (Supplementary Fig. 3 and Supplementary Fig. 4, bottom-right ROC curves). Because xseq-simple does not model the $H$ variables, it substituted the two conditional distributions $\theta_{G=\mathcal{L}|F=1,H=\mathcal{L}}$ and $\theta_{G=\mathcal{L}|F=1,H=\mathcal{G}}$ with a single conditional distribution $\theta_{G=\mathcal{L}|F=1}$. We could see this phenomenon from the parameter trace plots during EM algorithm iterations (Supplementary Fig. 5).

For completeness, we also did a simulation analysis of the cis-effects of somatic mutations (Supplementary Fig. 6). It is rare for a gene to be up-regulated across patients in the presence of loss-of-function mutations (i.e., $H = \mathcal{G}$). Since these events are used to estimate the parameters $\theta_{G|F=1,H=\mathcal{G}}$, we may not have enough events to robustly estimate these parameters. By contrast, the xseq-simple model (which does not have the $H$ variable) is sufficient for modelling the cis-effects of mutations. Therefore, we sampled data from the xseq-simple model for cis-analysis (using only the most challenging hyper-parameter regime). Compared to trans-analysis, it took longer for xseq to converge (more than 100 iterations), likely due to only one connected gene available for inference. Overall, results were similar to those obtained for trans simulations.

## 2.3 Downloading the Pan-Cancer datasets

We obtained the somatic mutation calls from the Synapse platform (syn1729383[58]). Affymetrix SNP6.0 copy number log2 values, GISTIC copy number calls, and RNASeq gene expression data were downloaded from syn300013[59].

## 2.4 Mutation probabilities in genes show bimodal distributions

Here we analyzed the mutations in the 127 significantly mutated genes predicted by MuSiC[60] to detect the genes whose xseq mutation probabilities $P(F)$ show bimodal distributions in at least one tumour type. For the mutations of a significantly mutated gene in a tumour type, the dip-test[61] was used to test for unimodality of the xseq mutation probability distribution ($P(F)$). The genes with FDR $\leq 0.2$ (Benjamini-Hochberg procedure[62]) were shown in Supplementary Table 8. Finally, for the genes given in Supplementary Table 8, we drew heatmaps as given in Supplementary Fig. 29 to show the heterogeneity in gene expression profiles.

## 2.5 Methods comparison

### 2.5.1 Comparison with CONEXIC

We ran CONEXIC algorithm on the TCGA breast cancer copy number and RNASeq expression data. We first used the default parameter setting and initialized the clustering using K-means with $K = 50$ clusters. The candidate driver CNAs were the focal copy number deletions and amplifications from the Pan-Cancer analysis[63]. Because of the large number of candidate driver genes (focal copy number alterations in 2891/3044 genes which have Entrez gene symbols), CONEXIC generated 1385 modules, with median module size of 6 and modulator number of 3. These modules selected uniquely 313 modulators, and 21/313 genes were putative cancer driver genes (Supplementary Table 6). Another 46/292 genes were directly connected partners of the *bona fide* cancer driver genes.

To compare with CONEXIC, we re-ran `xseq` on the TCGA breast cancer data using only DNA copy number and RNASeq expression data. This analysis generated 40 high-probability genes ($P(D) \geq 0.8$), and 13/40 genes were *bona fide* cancer driver genes (Supplementary Table 6). Another 13/27 genes were directly connected partners of the *bona fide* cancer driver genes. Ten genes overlapped with CONEXIC predictions: *BYSL, CALML3, CALML5, CCNE1, EGFR, ERBB2, EXOSC4, KPNA2, MRPS28*, and *PPP2R2A*.

We next used the 131 'target' genes of 140 focal copy number alterations predicted by GISTIC[63] as the candidate driver genes, and first initialized CONEXIC by single module analysis. The single module analysis generated 62 modules. We then ran CONEXIC ModuleNetwork with this initial clustering. This analysis generated 1136 modules, with median module size of 7 and modulator number of 2. These modules selected uniquely 72 modulators, and 27/72 genes were *bona fide* cancer driver genes (Supplementary Table 6). Another 12/45 genes were directly connected partners of the *bona fide* cancer driver genes. Sixteen of the 72 genes overlapped with `xseq` predictions: *CBX8, CCND1, CCNE1, CDKN2A, CYC1, E2F3, EGFR, ERBB2, KDM5A, MCL1, MRPS28, NEDD9, PPP2R2A, PTEN, SMARCA2*, and *STK11*.

Compared to CONEXIC, `xseq` is more specific while less sensitive. As can be seen from Supplementary Table 6, 26/40 genes predicted by `xseq` were either *bona fide* driver genes or directly connected parters of *bona fide* driver genes, while 67/313 CONEXIC predicted gene were either *bona fide* driver genes or directly connected partners of *bona fide* driver genes (p-value $\leq 0.01$). As an unsupervised method, CONEXIC can be powerful in prediction cancer driver modulators and modules when initialized properly, e.g., 39/72 genes were predicted as putative cancer genes when the 131 GISTIC focal copy number targets were used as inputs. For `xseq`, it needs 'negative' examples to learn model parameters, so we simply counted the overlap between the 40 predicted gene and the 131 GISTIC targets of focal copy number alterations, and found

16/20 genes were *bona fide* cancer genes. In addition, both methods uniquely predicted some known driver genes of breast cancer, e.g., CONEXIC predicted *ZNF703*, and `xseq` predicted *MYC* suggest these methods can compensate each other. Compared to CONEXIC, `xseq` has the advantages of predicting 'modulators' that mostly accumulate SNVs and indels, e.g., *TP53, CDH1*, and *GATA3* in breast cancer. Finally, `xseq` has the unique feature of estimating the impacts of individual mutations on gene expression.

### 2.5.2 Comparison with `xseq-simple`

Lastly we compared `xseq` to `xseq-simple`. Application of `xseq-simple` to the Pan-Cancer datasets predicted a strict subset of 106 genes (Supplementary Table 7), relative to the 150 genes predicted by `xseq`. An example gene predicted only by `xseq` is *CCNE1* in OV cancer as can seen from Supplementary Fig. 11 and Supplementary Fig. 12. The `xseq-simple` model only picked the *CCNE1* amplifications in 5/30 patients correlated with extreme expression dysregulation ($P(F) \geq 0.5$, Supplementary Fig. 18(b-c)). By considering the direction of gene regulation, `xseq` picked 19/30 amplification in *CCNE1* ($P(F) \geq 0.5$). In addition, considering the direction of gene-regulation does not sacrifice specificity, e.g., 89/149 predicted genes were either putative cancer genes or directly connected parters of putative cancer genes for the `xseq` model, compared to 65/105 `xseq-simple` predicted genes were either putative cancer genes or directly connected partners of putative cancer genes (Supplementary Table 7, p-value = 0.80).

# References


1. Smyth, G. K. Limma: linear models for microarray data. In *Bioinformatics and computational biology solutions using R and Bioconductor*, 397–420 (Springer, 2005).

2. Akavia, U. *et al.* An integrated approach to uncover drivers of cancer. *Cell* **143**, 1005–1017 (2010).

3. Jörnsten, R. *et al.* Network modeling of the transcriptional effects of copy number aberrations in glioblastoma. *Mol. Syst. Biol.* **7** (2011). doi: 10.1038/msb.2011.17.

4. Masica, D. L. & Karchin, R. Correlation of somatic mutation and expression identifies genes important in human glioblastoma progression and survival. *Cancer Res.* **71**, 4550–4561 (2011).

5. Bashashati, A. *et al.* DriverNet: uncovering the impact of somatic driver mutations on transcriptional networks in cancer. *Genome Biol.* **13** (2012). doi: 10.1186/gb-2012-13-12-r124.

6. Vaske, C. J. *et al.* Inference of patient-specific pathway activities from multi-dimensional cancer genomics data using PARADIGM. *Bioinformatics* **26**, i237–i245 (2010).

7. Curtis, C. *et al.* The genomic and transcriptomic architecture of 2,000 breast tumours reveals novel subgroups. *Nature* **486**, 346–352 (2012).

8. Ha, G. & Shah, S. Distinguishing somatic and germline copy number events in cancer patient dna hybridized to whole-genome snp genotyping arrays. In *Array Comparative Genomic Hybridization*, vol. 973, 355–372 (Humana Press, 2013).

9. Mermel, C. H. *et al.* GISTIC2.0 facilitates sensitive and confident localization of the targets of focal somatic copy-number alteration in human cancers. *Genome Biol.* **12** (2011). doi: 10.1186/gb-2011-12-4-r41.

10. Zhao, B. *et al.* Angiomotin is a novel Hippo pathway component that inhibits YAP oncoprotein. *Genes Dev.* **25**, 51–63 (2011).

11. Paramasivam, M., Sarkeshik, A., Yates, J. R., Fernandes, M. J. & McCollum, D. Angiomotin family proteins are novel activators of the LATS2 kinase tumor suppressor. *Mol. Biol. Cell* **22**, 3725–3733 (2011).

12. Yi, C. *et al.* A tight junction-associated Merlin-angiomotin complex mediates Merlin's regulation of mitogenic signaling and tumor suppressive functions. *Cancer cell* **19**, 527–540 (2011).

13. Chang, L. *et al.* The E3 ubiquitin ligase itch couples JNK activation to TNFalpha-induced cell death by inducing c-FLIP(L) turnover. *Cell* **124**, 601–613 (2006).

14. Stairs, D. B. *et al.* Deletion of p120-catenin results in a tumor microenvironment with inflammation and cancer that establishes it as a tumor suppressor gene. *Cancer cell* **19**, 470–483 (2011).

15. Thoreson, M. A. & Reynolds, A. B. Altered expression of the catenin p120 in human cancer: implications for tumor progression. *Differentiation* **70**, 583–589 (2002).

16. Usui, T. *et al.* Flamingo, a seven-pass transmembrane cadherin, regulates planar cell polarity under the control of frizzled. *Cell* **98**, 585–595 (1999).

17. Rothenberg, S. M. *et al.* A genome-wide screen for microdeletions reveals disruption of polarity complex genes in diverse human cancers. *Cancer Res.* **70**, 2158–2164 (2010).

18. Shi, J., Han, Q., Zhao, H., Zhong, C. & Yao, F. Downregulation of med23 promoted the tumorigenecity of esophageal squamous cell carcinoma. *Mol. Carcinog.* **53**, 833–840 (2014).

19. Ikebuchi, K. *et al.* RB1CC1 activates the promoter and expression of RB1 in human cancer. *Int. J. Cancer* **125**, 861–867 (2009).

20. Koinuma, D. *et al.* RB1CC1 protein positively regulates transforming growth factor-$\beta$ signaling through the modulation of Arkadia E3 ubiquitin ligase activity. *J. Biol. Chem.* **286**, 32502–32512 (2011).

21. Poulogiannis, G. *et al.* Prognostic relevance of DNA copy number changes in colorectal cancer. *J. Pathol.* **220**, 338–347 (2010).

22. Parisi, C. *et al.* Ago1 and Ago2 differentially affect cell proliferation, motility and apoptosis when overexpressed in SH-SY5Y neuroblastoma cells. *FEBS Lett.* **585**, 2965–2971 (2011).

23. Huang, V. *et al.* Ago1 interacts with RNA polymerase ii and binds to the promoters of actively transcribed genes in human cancer cells. *PLoS Genet.* **9** (2013). dii: 10.1371/journal.pgen.1003821.

24. Izzo, M. W., Strachan, G. D., Stubbs, M. C. & Hall, D. J. Transcriptional repression from the c-myc P2 promoter by the zinc finger protein ZF87/MAZ. *J. Biol. Chem.* **274**, 19498–19506 (1999).

25. Stubbs, M. C. *et al.* The ZF87/MAZ transcription factor functions as a growth suppressor in fibroblasts. *Biochem. Cell Biol.* **78**, 477–485 (2000).

26. Baxter, E. L. *Investigating the association between BRAFV600E and methylation in sporadic colon cancer*. Ph.D. thesis, The University of Edinburgh (2012).

27. Casper, M. *et al.* Hepatocellular carcinoma as extracolonic manifestation of lynch syndrome indicates SEC63 as potential target gene in hepatocarcinogenesis. *Scand. J. Gastroenterol.* **48**, 344–351 (2013).

28. Morishima, N., Nakanishi, K. & Nakano, A. Activating transcription factor-6 (ATF6) mediates apoptosis with reduction of myeloid cell leukemia sequence 1 (Mcl-1) protein via induction of ww domain binding protein 1. *J. Biol. Chem.* **286**, 35227–35235 (2011).

29. Shah, P. *et al.* Ubiquilin1 represses migration and epithelial-to-mesenchymal transition of human non-small cell lung cancer cells. *Oncogene* **34**, 1709–1717 (2015).

30. Oner, S. S. *et al.* Regulation of the G-protein regulatory-G$\alpha$i signaling complex by nonreceptor guanine nucleotide exchange factors. *J. Biol. Chem.* **288**, 3003–3015 (2013).

31. Chan, P., Thomas, C. J., Sprang, S. R. & Tall, G. G. Molecular chaperoning function of Ric-8 is to fold nascent heterotrimeric G protein $\alpha$ subunits. *Proc. Natl Acad. Sci. USA* **110**, 3794–3799 (2013).

32. Muggerud, A. A. *et al.* Data integration from two microarray platforms identifies bi-allelic genetic inactivation of RIC8A in a breast cancer cell line. *BMC med. genomics* **2** (2009). doi: 10.1186/1755-8794-2-26.

33. Yabas, M. *et al.* ATP11C is critical for the internalization of phosphatidylserine and differentiation of B lymphocytes. *Nat. Immunol.* **12**, 441–449 (2011).

34. Segawa, K. *et al.* Caspase-mediated cleavage of phospholipid flippase for apoptotic phosphatidylserine exposure. *Science* **344**, 1164–1168 (2014).

35. Peikert, T., Specks, U., Farver, C., Erzurum, S. C. & Comhair, S. A. Melanoma antigen A4 is expressed in non–small cell lung cancers and promotes apoptosis. *Cancer Res.* **66**, 4693–4700 (2006).

36. Bhan, S., Chuang, A., Negi, S. S., Glazer, C. A. & Califano, J. A. MAGEA4 induces growth in normal oral keratinocytes by inhibiting growth arrest and apoptosis. *Oncol. Rep.* **28**, 1498–1502 (2012).

37. Nagao, T. *et al.* MAGE-A4 interacts with the liver oncoprotein gankyrin and suppresses its tumorigenic activity. *J. Biol. Chem.* **278**, 10668–10674 (2003).

38. Lee, M.-Y., Moreno, C. S. & Saavedra, H. I. E2f activators signal and maintain centrosome amplification in breast cancer cells. *Mol. Cell. Biol.* **34**, 2581–2599 (2014).

39. Avery, S. *et al.* Bcl-xl mediates the strong selective advantage of a 20q11. 21 amplification commonly found in human embryonic stem cell cultures. *Stem cell reports* **1**, 379–386 (2013).

40. Shah, S. A. *et al.* 26s proteasome inhibition induces apoptosis and limits growth of human pancreatic cancer. *J. Cell. Biochem.* **82**, 110–122 (2001).

41. Minchenko, O. H. *et al.* Molecular mechanisms of ERN1-mediated angiogenesis. *Int. J. Physiol. Pathophys.* **5**, 1–22 (2014).

42. Leverson, J. *et al.* Potent and selective small-molecule MCL-1 inhibitors demonstrate on-target cancer cell killing activity as single agents and in combination with ABT-263 (navitoclax). *Cell Death Differ.* **6** (2015). doi: 10.1038/cddis.2014.561.

43. Irie, H. Y., Halstead-Nussloch, G. & Ito, K. Abstract p4-05-10: Prkcq, a novel protein kinase c preferentially expressed in triple negative breast cancer, drives oncogenic growth, survival and migration. *Cancer Res.* **75**, P4–05 (2015).

44. Zhou, W. *et al.* Cancer-secreted mir-105 destroys vascular endothelial barriers to promote metastasis. *Cancer cell* **25**, 501–515 (2014).

45. Wang, Y. *et al.* Melk is an oncogenic kinase essential for mitotic progression in basal-like breast cancer cells. *Elife* **3** (2014). doi: 10.7554/eLife.01763.

46. Bennett, G., Sadlier, D., Doran, P. P., MacMathuna, P. & Murray, D. W. A functional and transcriptomic analysis of net1 bioactivity in gastric cancer. *BMC cancer* **11** (2011). doi: 10.1186/1471-2407-11-50.

47. Fletcher, C. E. *et al.* Androgen-regulated processing of the oncomir mir-27a, which targets prohibitin in prostate cancer. *Hum. Mol. Gen.* **21**, 3112–3127 (2012).

48. He, X., Khurana, A., Roy, D., Kaufmann, S. & Shridhar, V. Loss of hsulf-1 expression enhances tumorigenicity by inhibiting bim expression in ovarian cancer. *Int. J. Cancer* **135**, 1783–1789 (2014).

49. Nonet, G. H. *et al.* The znf217 gene amplified in breast cancers promotes immortalization of human mammary epithelial cells. *Cancer Res.* **61**, 1250–1254 (2001).

50. Dietrich, N. *et al.* Bypass of senescence by the polycomb group protein cbx8 through direct binding to the ink4a-arf locus. *EMBO J.* **26**, 1637–1648 (2007).

51. Palian, B. M. *et al.* Maf1 is a novel target of pten and pi3k signaling that negatively regulates oncogenesis and lipid metabolism. *PLoS Genet.* **10**, e1004789 (2014).

52. Li, S. *et al.* Bzw1, a novel proliferation regulator that promotes growth of salivary muocepodermoid carcinoma. *Cancer Lett.* **284**, 86–94 (2009).

53. Qu, H. *et al.* Zbtb7 overexpression contributes to malignancy in breast cancer. *Cancer Invest.* **28**, 672–678 (2010).

54. Yang, X. *et al.* Zbtb7 suppresses the expression of cdk2 and e2f4 in liver cancer cells: implications for the role of zbtb7 in cell cycle regulation. *Mol Med Rep.* **5**, 1475–1480 (2012).

55. Lin, K. W., Yakymovych, I., Jia, M., Yakymovych, M. & Souchelnytskyi, S. Phosphorylation of eef1a1 at ser300 by t$\beta$r-i results in inhibition of mrna translation. *Curr. Biol.* **20**, 1615–1625 (2010).

56. Pearl, J. Reverend bayes on inference engines: A distributed hierarchical approach. In *Proceedings of the second national conference on artificial intelligence*, 133–136 (AAAI Press, 1982).

57. Kim, J. H. & Pearl, J. A computational model for causal and diagnostic reasoning in inference systems. In *Proceedings of the Eighth international joint conference on Artificial intelligence-Volume 1*, 190–193 (Morgan Kaufmann Publishers Inc., 1983).

58. Kandoth, C. syn1729383 (2013). URL `https://www.synapse.org/#!Synapse:syn1729383`.

59. Ellrott, K. syn300013 (2013). URL `https://www.synapse.org/#!Synapse:syn300013`.

60. Kandoth, C. *et al.* Mutational landscape and significance across 12 major cancer types. *Nature* **502**, 333–339 (2013).

61. Hartigan, P. Algorithm as 217: Computation of the dip statistic to test for unimodality. *J. R. Stat. Soc. Ser. C Appl. Stat.* **34**, 320–325 (1985).

62. Benjamini, Y. & Hochberg, Y. Controlling the false discovery rate: a practical and powerful approach to multiple testing. *J. R. Stat. Soc. Ser. B Stat. Methodol.* **57**, 289–300 (1995).

63. Zack, T. I. *et al.* Pan-cancer patterns of somatic copy number alteration. *Nat. Genet.* **45**, 1134–1140 (2013).
